# Supplementary material for: Chromosome-scale genome assembly reveals how repeat elements shape non-coding RNA landscapes active during newt limb regeneration
Source: Cell Genom. 2025 Jan 27;5(2):100761. doi: 10.1016/j.xgen.2025.100761 (PMC11872487; doi:10.1016/j.xgen.2025.100761)
Supplement: Document S1. Figures S1–S26 [file mmc1.pdf]

Cell Genomics, Volume *5*

Supplemental information

Chromosome-scale genome assembly reveals how repeat elements shape non-coding RNA landscapes active during newt limb regeneration

Thomas Brown, Ketan Mishra, Ahmed Elewa, Svetlana Iarovenko, Elaiyaraja Subramanian, Alberto Joven Araus, Andreas Petzold, Bastian Fromm, Marc R. Friedländer, Lennart Rikk, Miyuki Suzuki, Ken-ichi T. Suzuki, Toshinori Hayashi, Atsushi Toyoda, Catarina R. Oliveira, Ekaterina Osipova, Nicholas D. Leigh, Maximina H. Yun, and András Simon

This file includes:
Figure S1 - 26

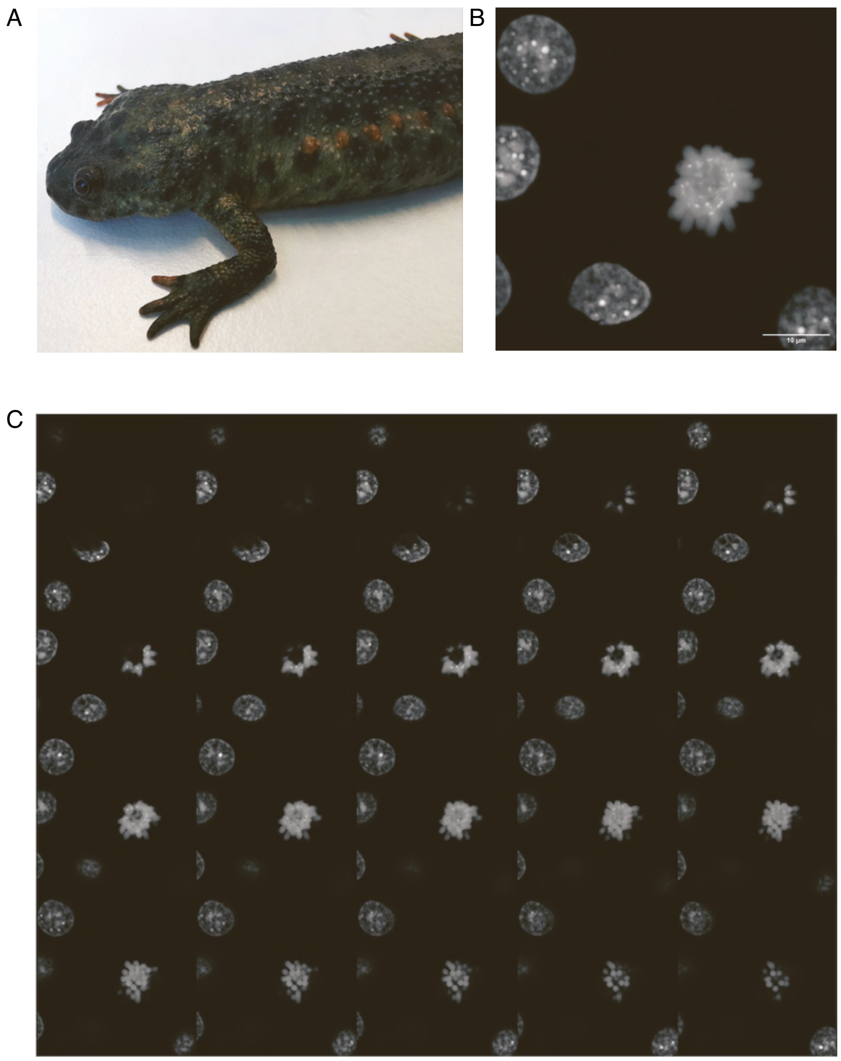

Figure S1. Tissue collection for genome sequencing, related to Figure 1.
(A) Adult female Iberian Ribbed newt used in the present study for tissue collection and subsequent genome sequencing, Hi-C, as well as brain and spleen Iso-Seq. (B) Maximum intensity projection of a Z-stack confocal image of chondrocytes found in the tail vertebra in interphase and metaphase. The cells show both the expected size of the nucleus for a diploid animal (10µm-diameter) and the correct number of chromosomes (2n=24). (C) Individual Z-planes corresponding to Figure S1b.

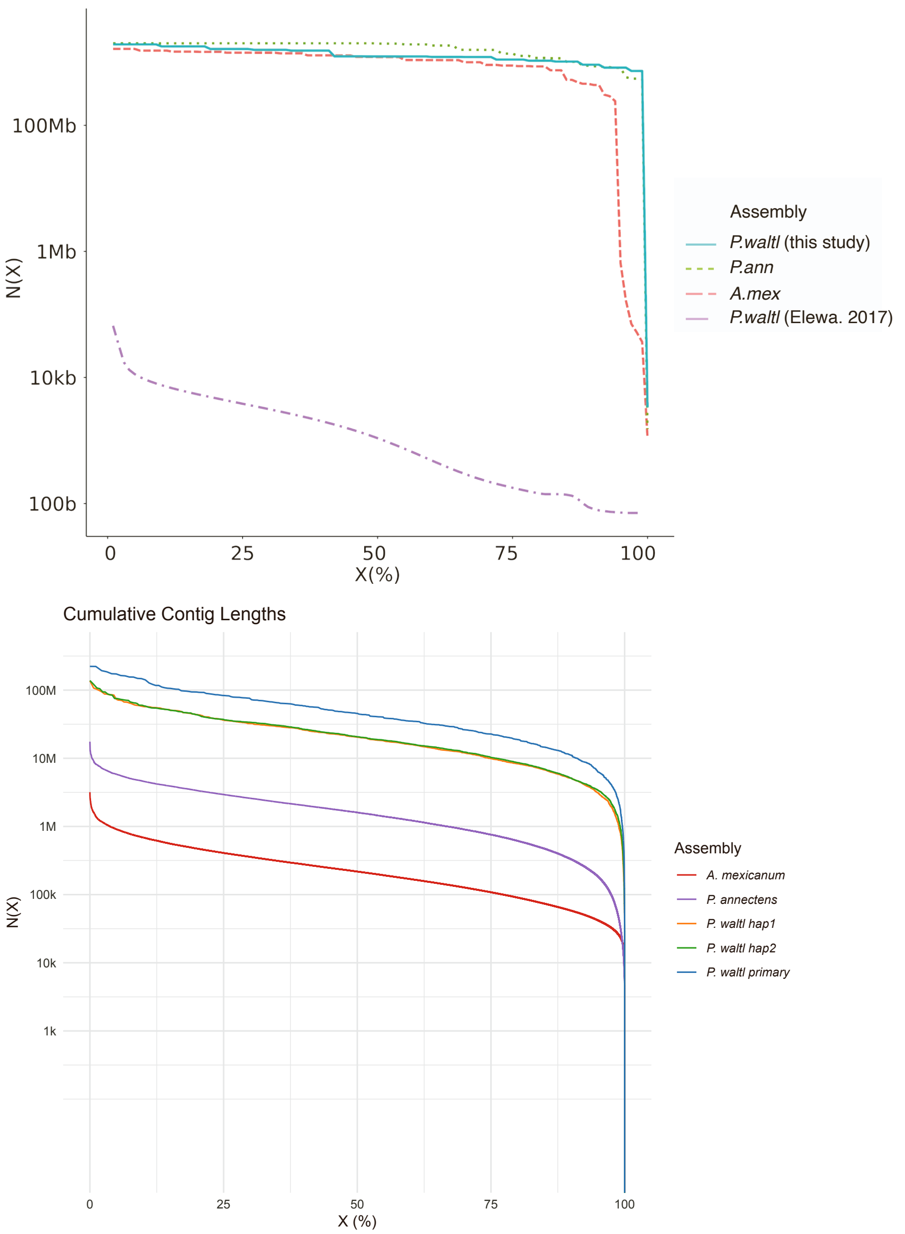

Figure S2. Scaffold plots of contig statistics, related to Figure 1.

Scaffold N(X) plot showing which % of each assembled genome (X) is contained within pieces at least N(X) bp in size. Shown are contig statistics from (top) *Pleurodeles waltl* (this study and [S1]), *Protopterus annectens* [S2], *Ambystoma mexicanum* [S3]. Contig statistics from (bottom) *P. waltl* (Primary, Haploid 1 and Haploid 2), *P. annectens* [S2], *A. mexicanum* [S3].

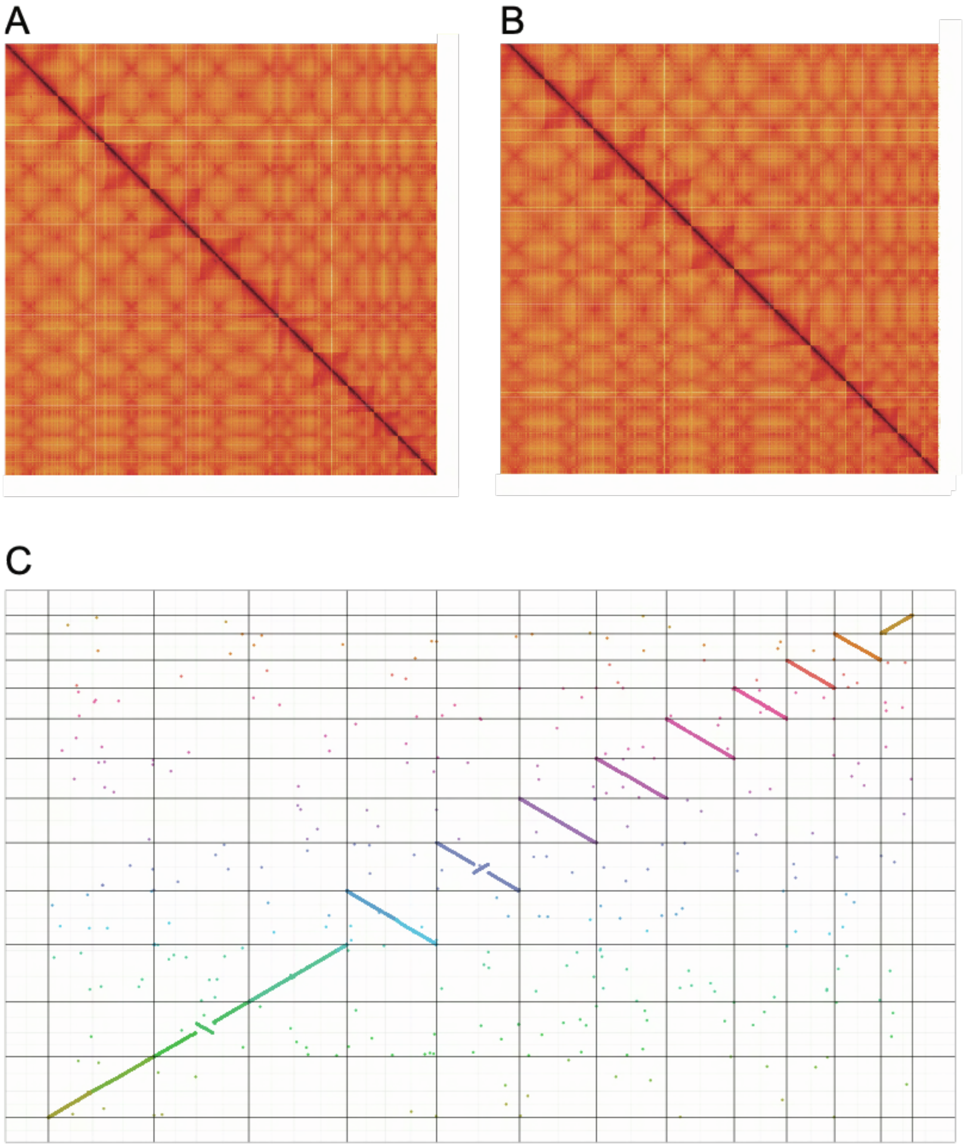

Figure S3. Assembled haplotype HiC contact maps and macrosyntenic relationships, related to Figure 1.

HiC contact maps (A, B) of the two assembled haplotypes and Oxford plot (C) of macrosyntenic relationships between the two assembled haplotypes of *P. waltl.*

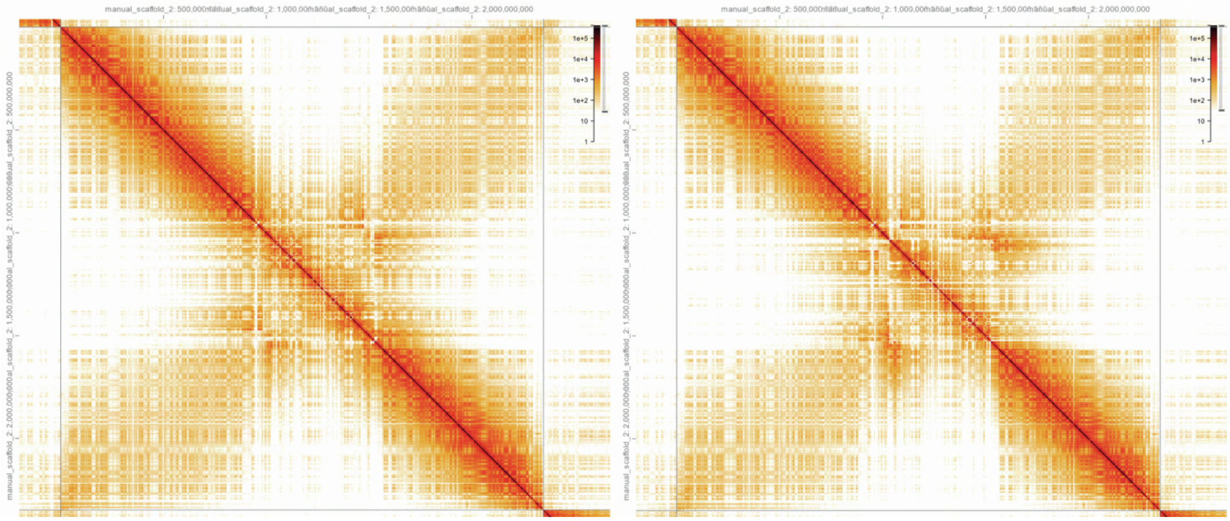

Figure S4. 500Mb inversion of the central region of chromosome 2, related to Figure 1.

Hi-C interaction heatmap of contact data for chromosome 2 (left) and corresponding heatmap after inverted central 500Mb region (right).

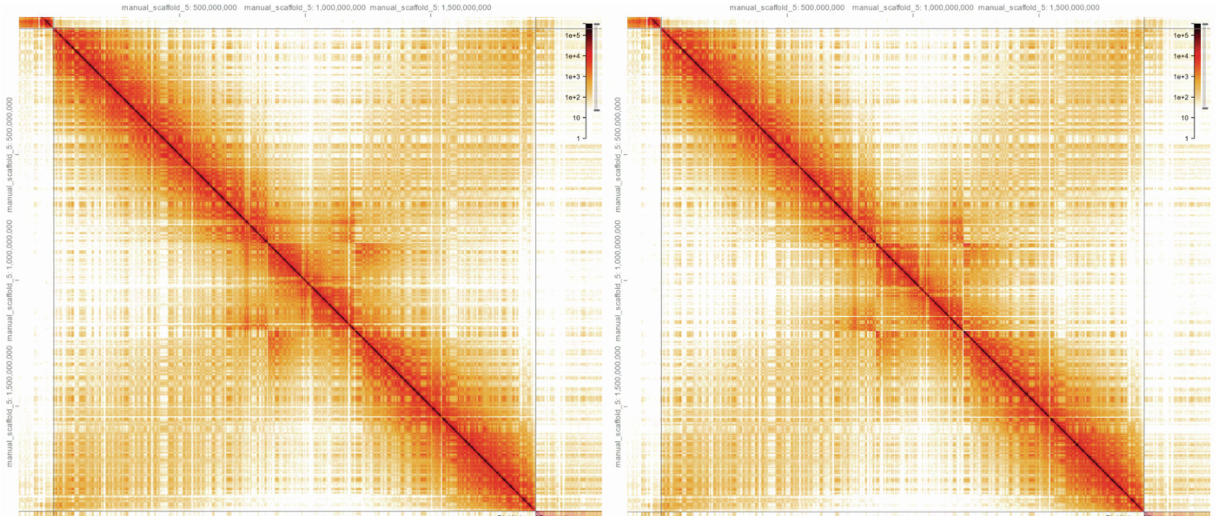

Figure S5. 350Mb inversion of the central region of chromosome 5, related to Figure 1.

Hi-C interaction heatmap of contact data for chromosome 5 (left) and corresponding heatmap after inverted central 350Mb region (right).

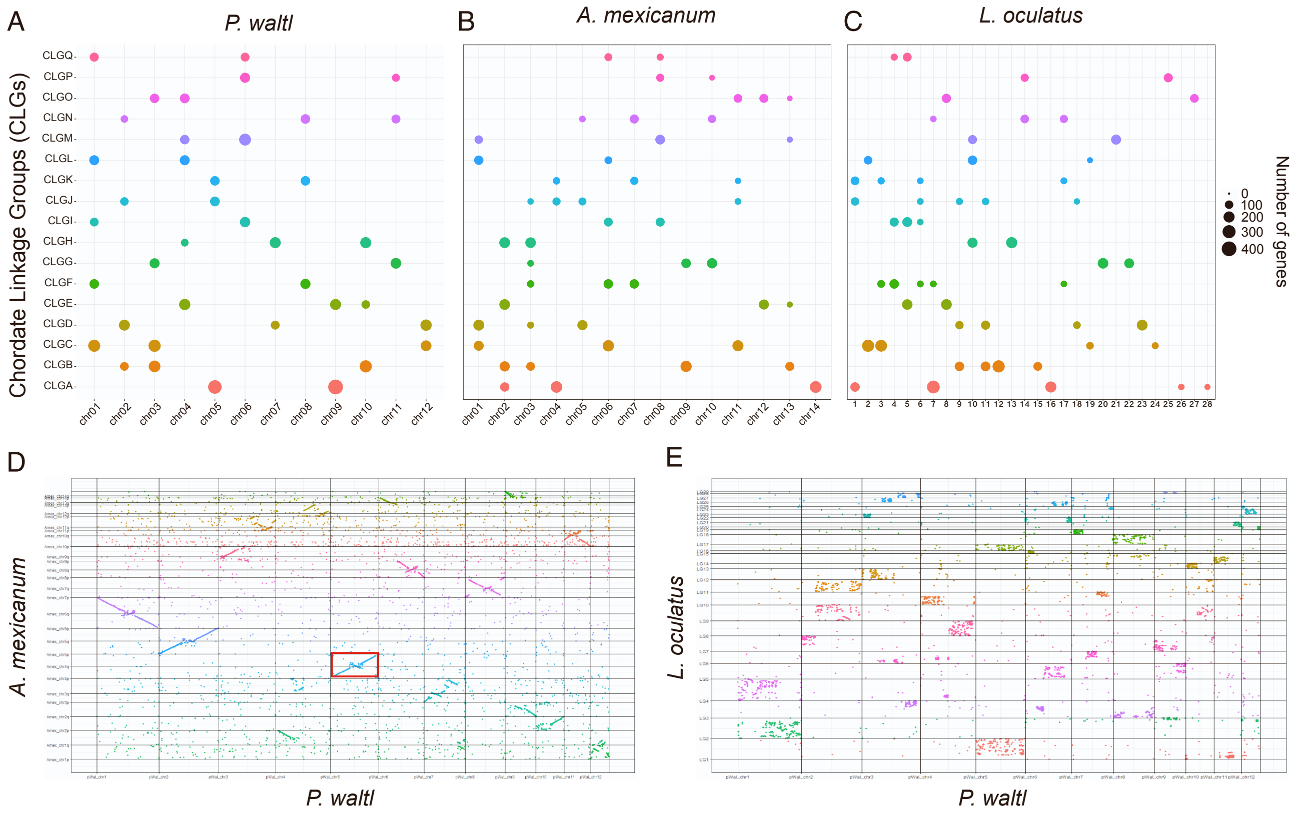

Figure S6. Comparative synteny plots, related to Figure 2.

Synteny Blocks represents regions of the newt, axolotl and gar genomes identified as containing co-localised blocks of genes with the Chordate Linkage Groups (CLGs) in newt (A), axolotl (B) and gar. The size of each circle corresponds to the number of genes in each identified syntenic block. Circles are coloured by CLG and individual numbers are also available in Table S2-6. Comparative macrosynteny of the *P. waltl* genome**.** (D) Oxford plot of macrosyntenic relationships between *P. waltl* and *A. mexicanum* chromosomes. Coloured dots indicate relative chromosomal arrangement of newt-axolotl orthologues. Inversion of *P. waltl* chromosome 5 is indicated by the red rectangle. (E) Oxford plot depicting macrosyntenic relationships between *P. waltl* and *L. oculatus* orthologues. LG: linkage group, chr: chromosome.

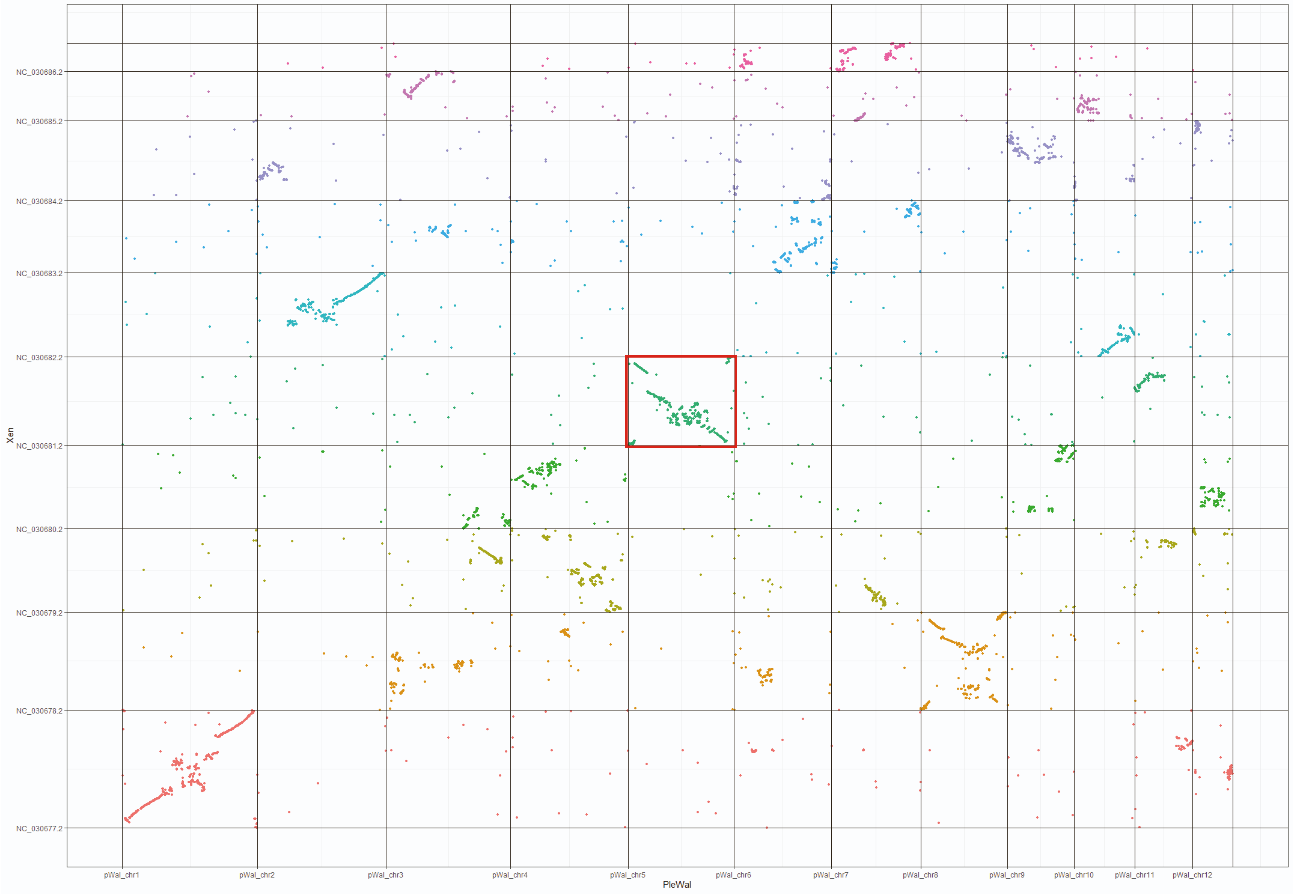

Figure S7. Macrosyntenic relationships between *P. waltl* and *X. tropicalis*, related to Figure 2.
Oxford plot of macrosyntenic relationships between *P. waltl* and *X. tropicalis* chromosomes based on 13,736 one-to-one orthologues. Coloured dots indicate relative chromosomal arrangement of newt-frog orthologues. Inversion of *P. waltl* chromosome 5 is indicated by the red rectangle.

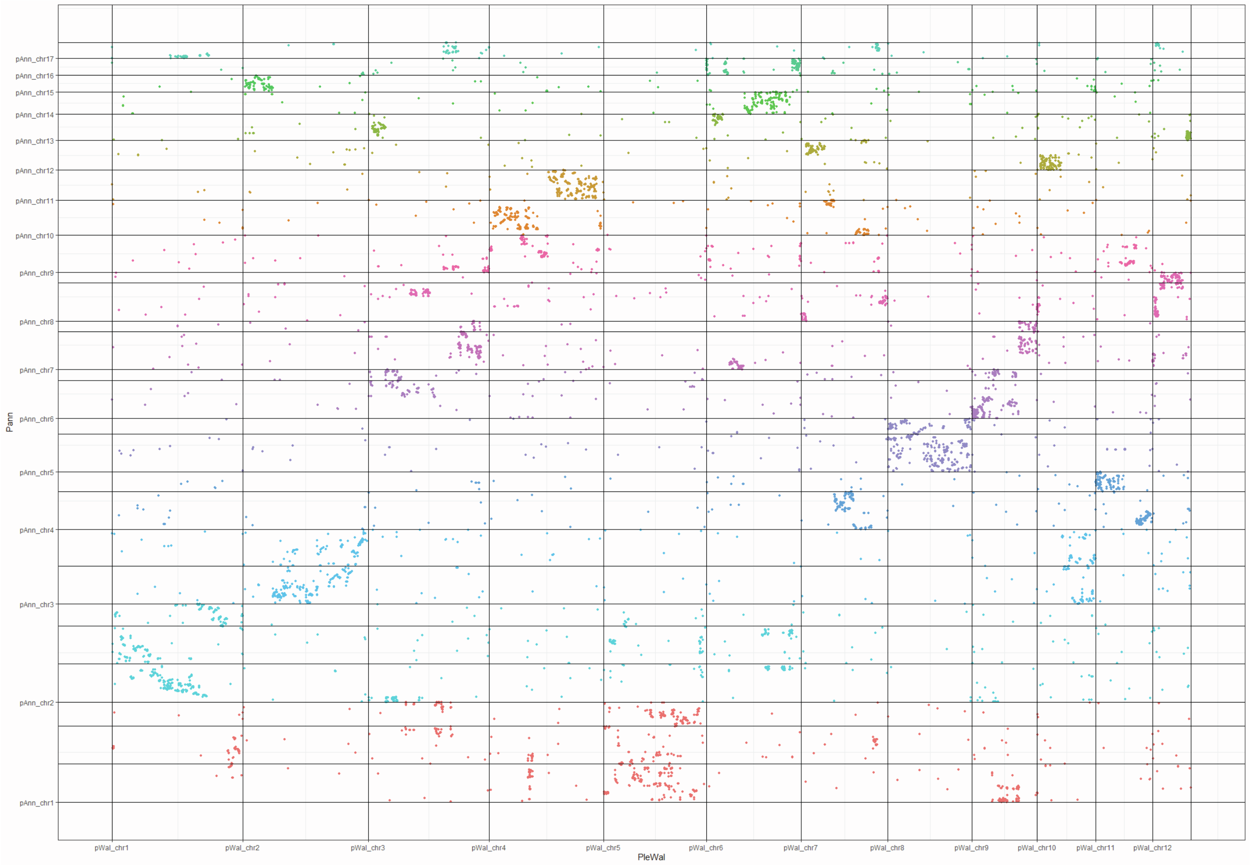

Figure S8. Macrosyntenic relationships between *P. waltl* and *P. annectens*, related to Figure 2. Oxford plot of macrosyntenic relationships between *P. waltl* and *P. annectens* chromosomes based on 13,423 one-to-one orthologues. Coloured dots indicate relative chromosomal arrangement of newt-lungfish orthologues.

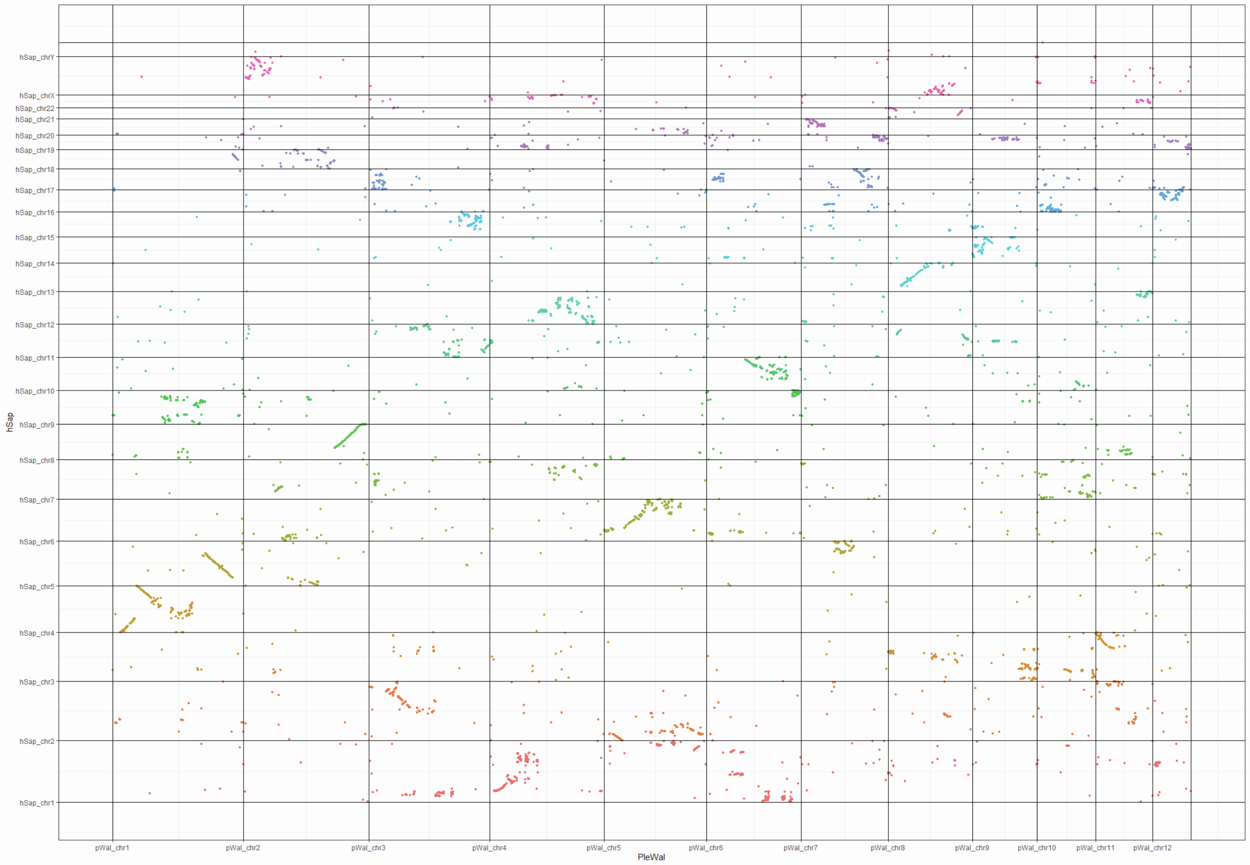

Figure S9. Macrosyntenic relationships between *P. waltl* and *H. sapiens*, related to Figure 2.

Oxford plot of macrosyntenic relationships between *P. waltl* and *H. sapiens* chromosomes based on 13,127 one-to-one orthologues. Coloured dots indicate relative chromosomal arrangement of newt-human orthologues.

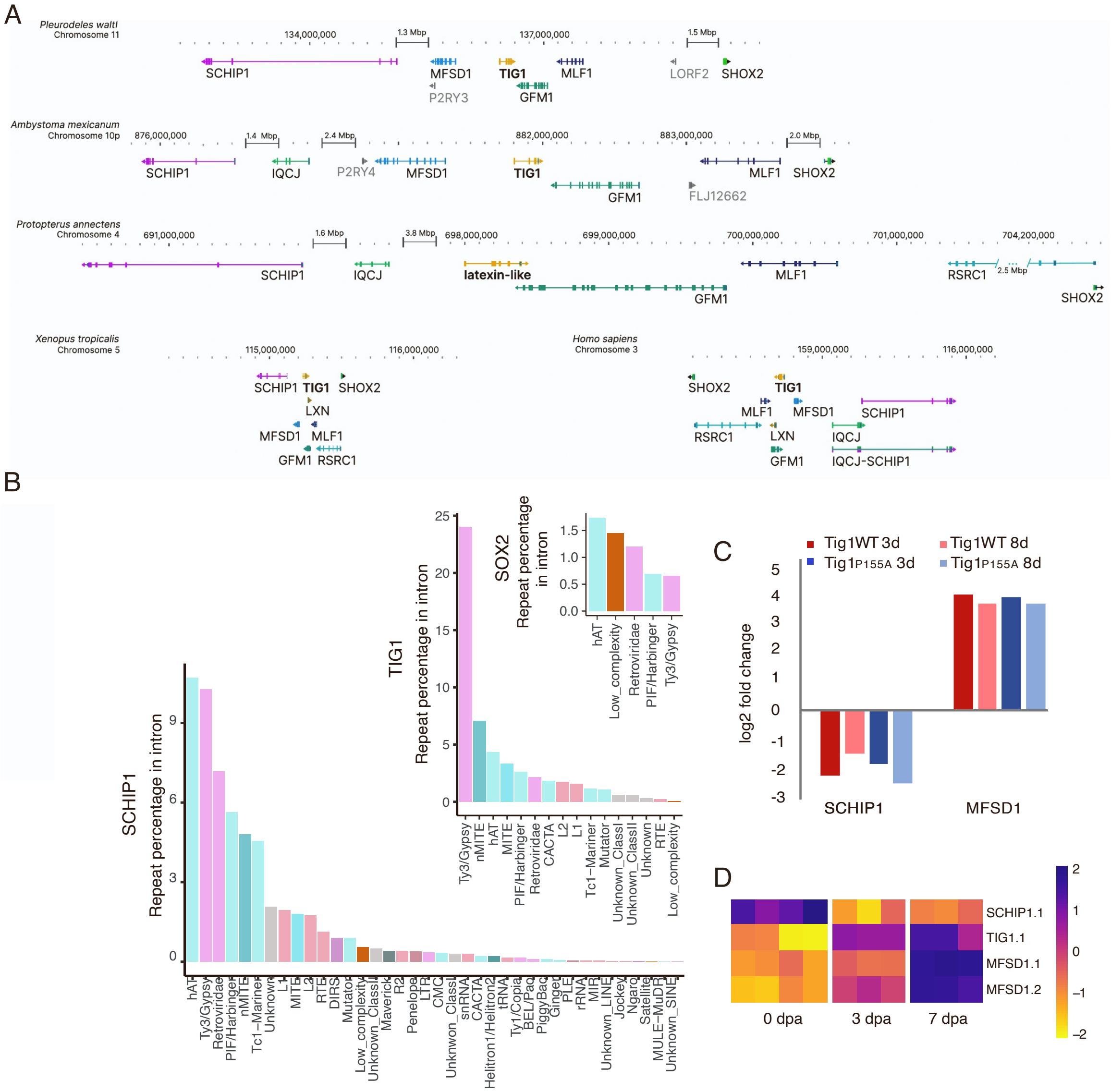

Figure S10. Microsynteny conservation of the *Tig1* locus, related to Figure 2.
(A) Analysis of genomic location and structure of genes associated with the *Tig1* locus in the indicated vertebrate species. Genes are indicated in colour. Arrowheads indicate reading frame direction. Perpendicular bars within genes represent exons. Note the increase in intron size for all genes associated with the *Tig1* locus in *P. waltl* compared to *X. tropicalis* and *H. sapiens*. Variation in intron length among species with giant genomes is indicative of independent intron expansion. (B) Relative contribution of repeat elements to intron expansion for the indicated genes. (C) *Tig1* overexpression affects expression of nearby genes in *A. mexicanum*. Bars represent ratios of gene expression in *Tig1* or *Tig1P155A* overexpressing cells versus control cells (analysis based on dataset [S4]). (D) Heatmap depicting differences in gene expression of *Tig1* and its genomic neighbours during *P. waltl* limb regeneration.

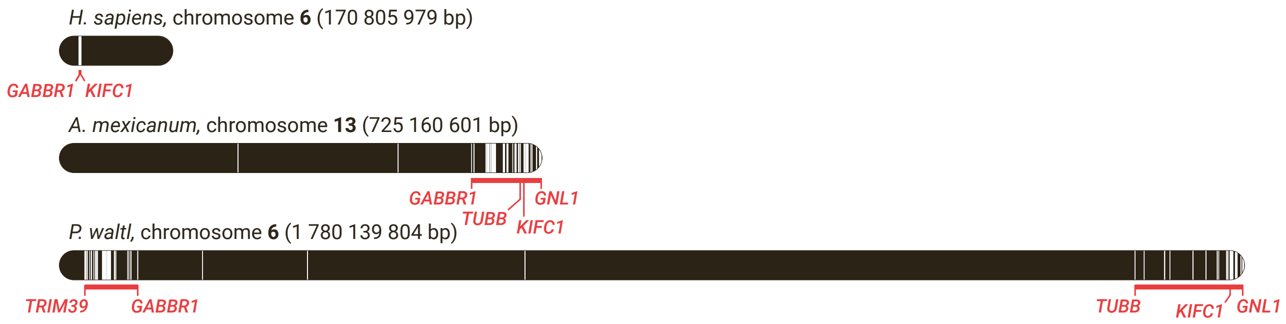

Figure S11. Comparative analysis of the Major Histocompatibility Complex (MHC) locus, related to Figure 2.

The *P. waltl* Major Histocompatibility Complex (MHC) locus shows a bimodal distribution. White lines represent location of MHC locus genes in *H. sapiens*, *A. mexicanum*, and *P. waltl* chromosomes. Red underlines highlight regions of higher density of MHC genes. The *P. waltl* MHC locus demonstrates a bimodal distribution with a gap (i.e., black region) representing a dearth of MHC genes.

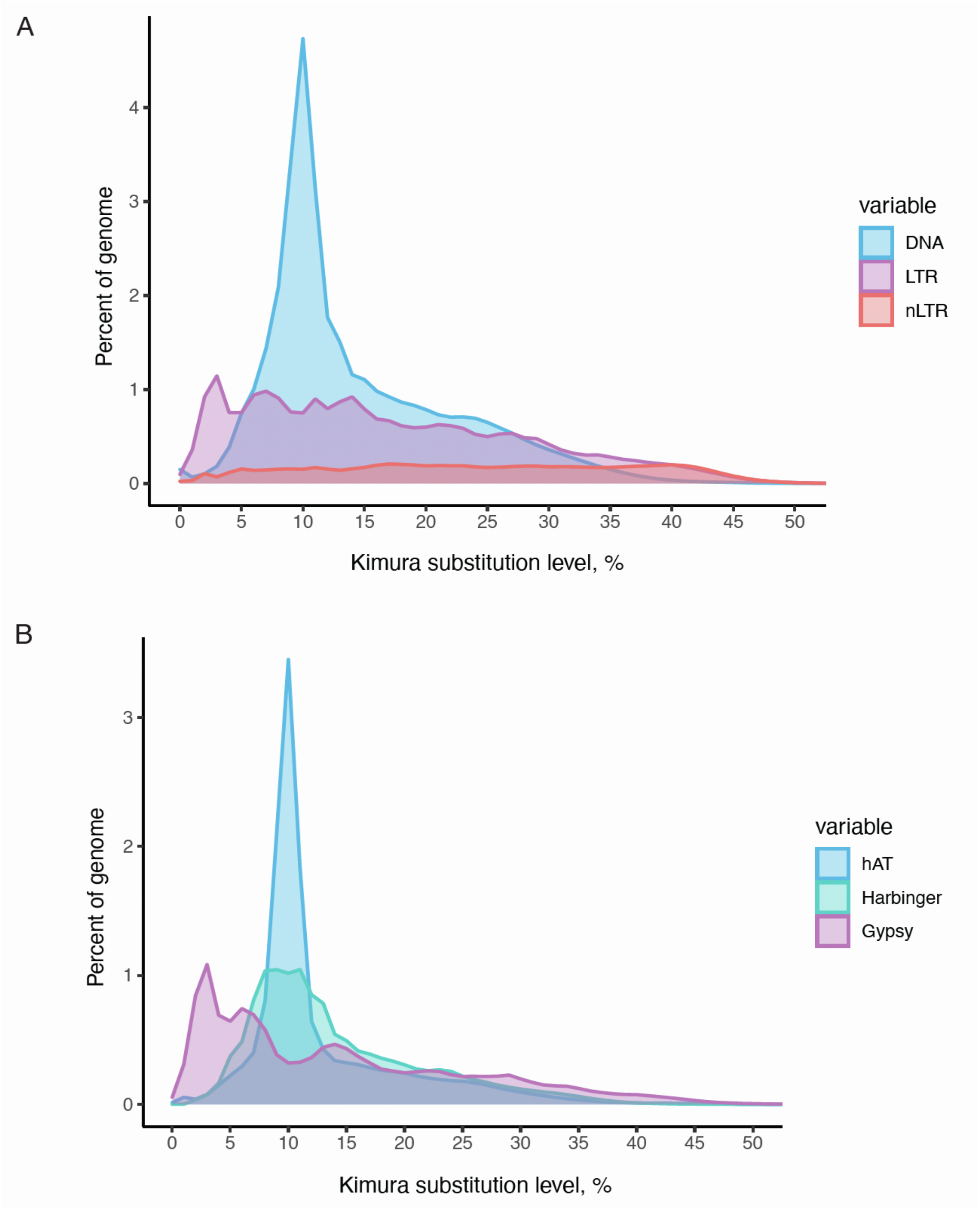

Figure S12. Kimura analysis of transposable element expansion, related to Figure 3.
Expansion history of transposable elements based on the Kimura substitution level for each copy of the indicated repetitive element against its consensus sequence: (A) for the indicated repeat types; (B) for the top contributor transposable elements families.

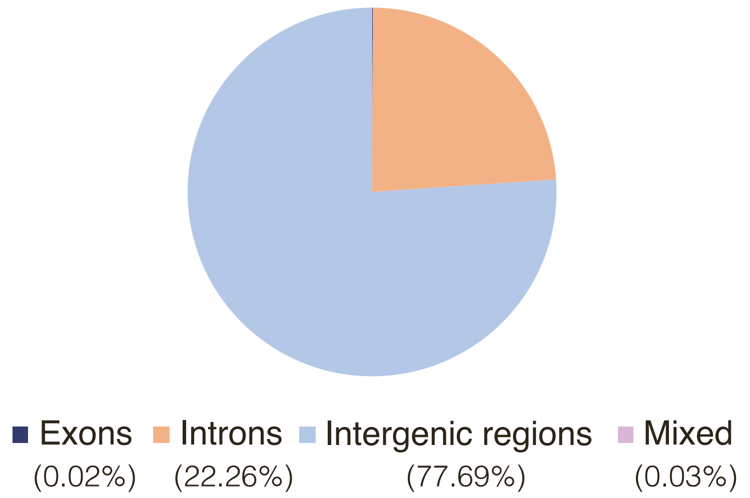

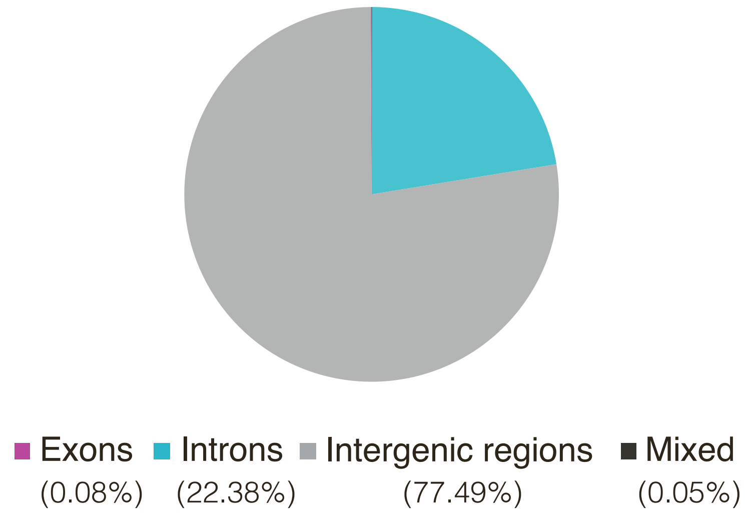

Figure S13. Genomic distribution of repeat elements, related to Figure 3.

Distribution of hAT elements (top) and all repeats (bottom) within exons, introns and intergenic regions of the *P. waltl* genome, expressed as % contribution to each genomic component.

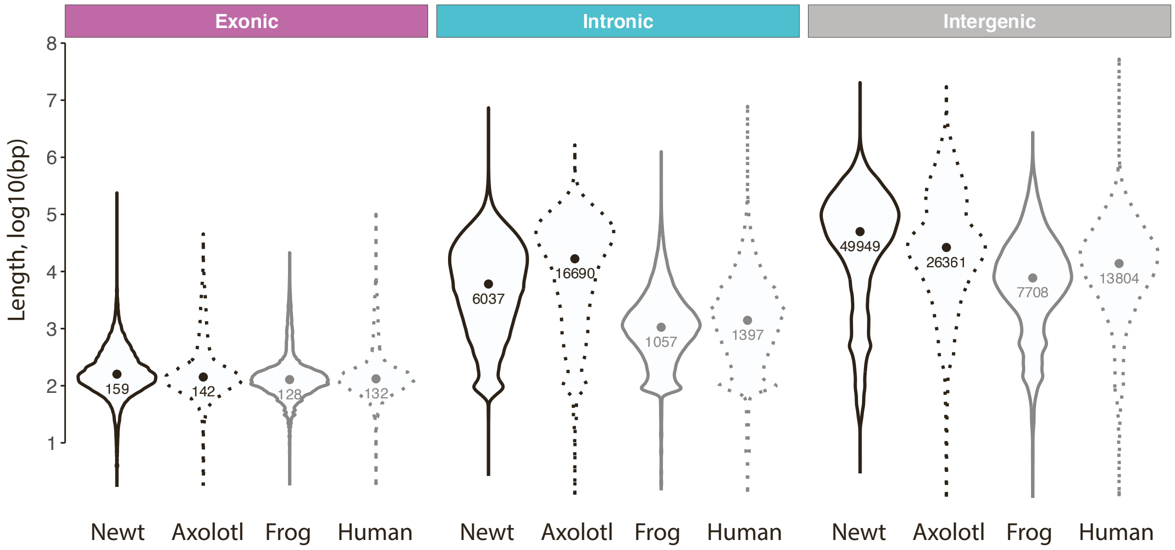

Figure S14. Comparison of genomic region sizes, related to Figure 3.

Analysis of exon/intron/intergenic region size in *P. waltl* compared to axolotl, frog, and human. Exon dimensions (median: 159bp; mean: 277bp; maximum: 200Kb), intron dimensions (median: 6Kb; mean: 17.8Kb; maximum: 4.8Mb) and intergenic regions (median: 50Kb; mean: 116Kb; maximum: 11.8 Mb).

Violin plots for exon (left, in kb), intronic region (middle, in kb) and intergenic (right, in bp) size. Coloured circles indicate the median of each distribution.

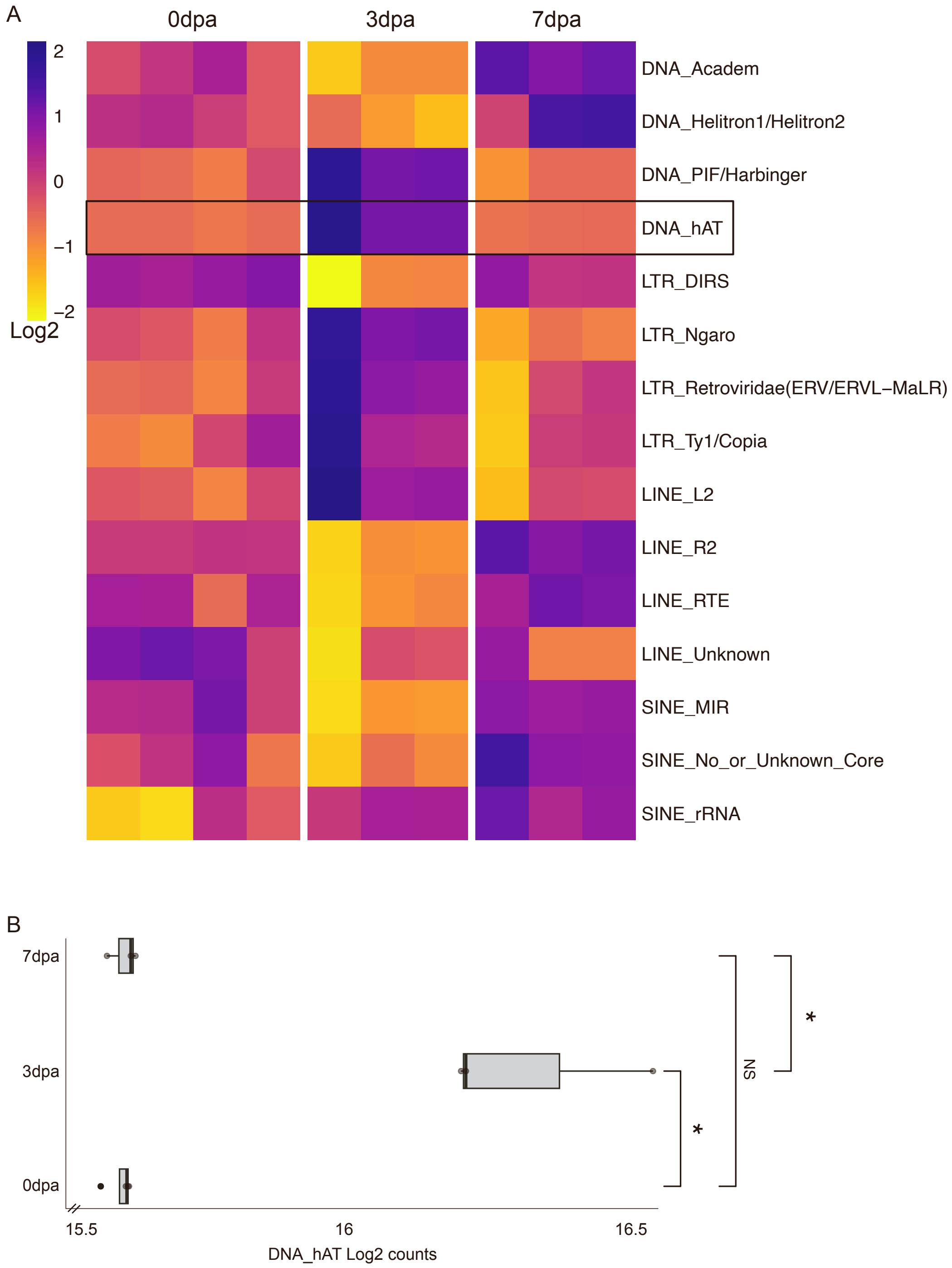

Figure S15. Repeat element expression during limb regeneration, related to Figure 3.

(A) Expression of repeat elements across regenerating *P. waltl* limb samples (RNA-seq from [S1]). Only showing repeat element with a significant change in expression. Boxed element indicates (B). (B) DNA hAT elements show a significant upregulation on 3dpa (n =3), whereas 7dpa (n=3) levels resumes to 0dpa (n=4). * = $p < 0.05$, Anova followed by pairwise t-test. dpa = days post amputation.

A

	>2000bp			500-2000bp			<500bp		
	type	number	percent	type	number	percent	type	number	percent
blastema	Ac	1	0,08	Ac	12	0,37	Ac	78	0,91
	Charlie	5	0,38	Charlie	363	11,21	Charlie	1027	12,01
	nMITE	1273	96,66	nMITE	2331	71,99	nMITE	2281	26,67
	Tip100	38	2,89	Tip100	4	0,12	Tip100	67	0,78
				MITE	460	14,21	MITE	4414	51,60
				Unknown	68	2,10	Blackjack	73	0,85
							Unknown	614	7,18

	>2000bp			500-2000bp			<500bp		
	type	number	percent	type	number	percent	type	number	percent
brain	Ac	1	0,27				Ac	14	0,24
	Charlie	1	0,27	Charlie	222	12,07	Charlie	702	11,97
	nMITE	355	96,99	nMITE	1281	69,62	nMITE	1482	25,27
	Tip100	9	2,46	Tip100	2	0,11	Tip100	43	0,73
				MITE	281	15,27	MITE	3069	52,33
				Unknown	54	2,93	Blackjack	69	1,18
							Unknown	486	8,29

	>2000bp			500-2000bp			<500bp		
	type	number	percent	type	number	percent	type	number	percent
spleen	Ac	1	0,25	Ac	2	0,10	Ac	13	0,22
	Charlie	2	0,50	Charlie	250	12,96	Charlie	788	13,20
	nMITE	383	96,23	nMITE	1334	69,16	nMITE	1554	26,03
	Tip100	12	3,02	Tip100	4	0,21	Tip100	42	0,70
				MITE	292	15,14	MITE	3056	51,20
				Unknown	47	2,44	Blackjack	75	1,26
							Unknown	441	7,39

B

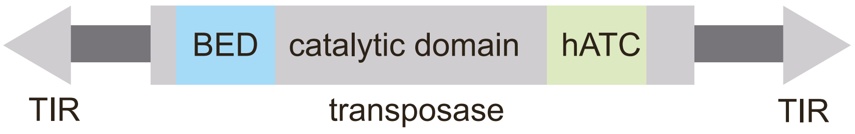

Figure S16. Expression and structure of hAT elements, related to Figure 3.

(A) Expression of hAT elements in Iso-seq-derived transcriptomes from limb blastema, brain and spleen categorised by element length (bp). (B) Schematic diagram representing the structure of a standard hAT, indicating key domains and transposition sites. BED: Zinc Finger BED domain; TIR: Terminal Inverted Repeat; hATC: C terminal dimerization domain.

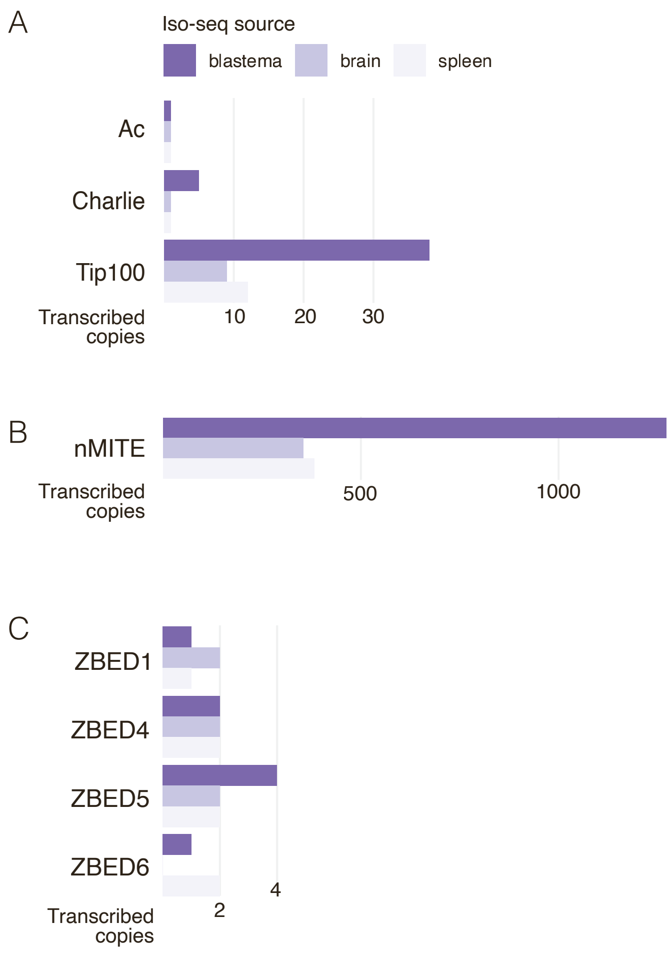

Figure S17. Transcribed copies of repeat elements, related to Figure 3.

Number of transcribed copies of repeat elements >2000 bp for (A, B) the indicated hAT or (C) domesticated hAT categories based on PacBio Iso-seq data for limb blastema, brain and spleen.

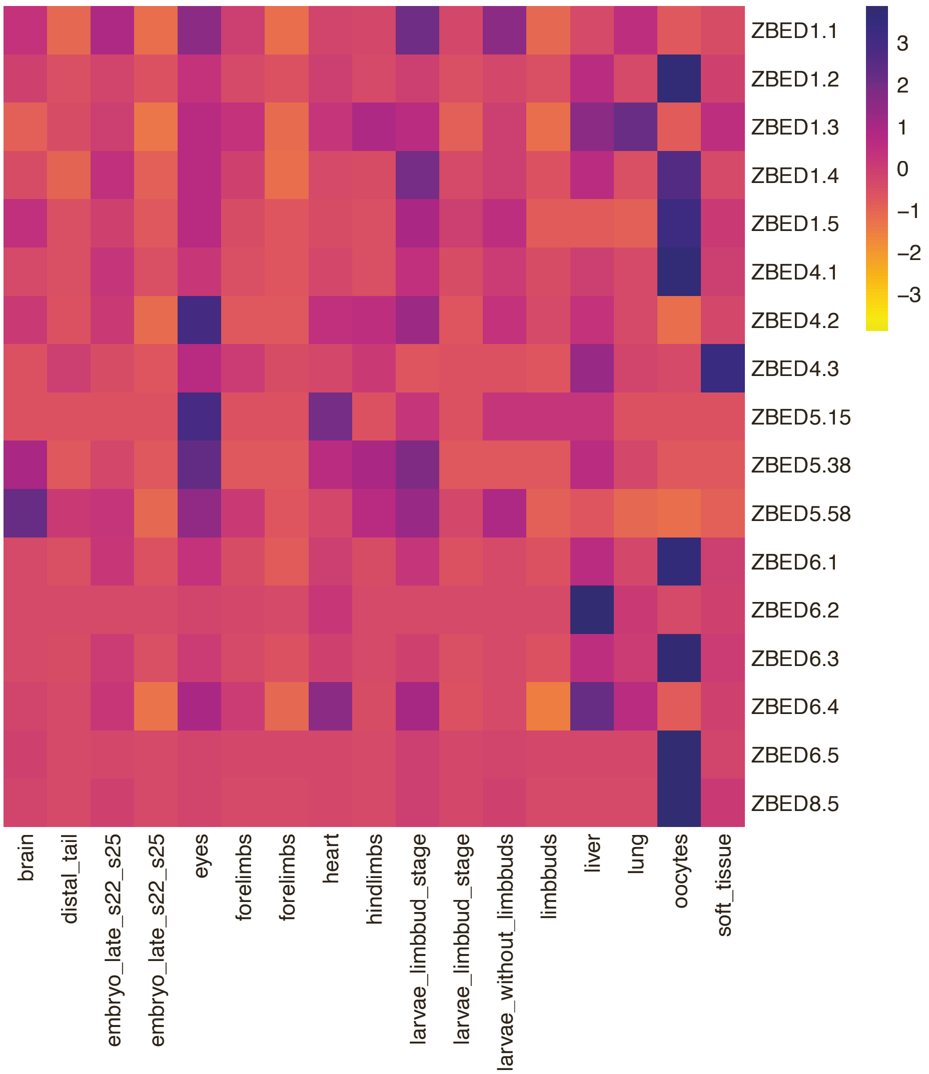

Figure S18. Expression of hATs across various tissues, related to Figure 3.

RNA-seq quantification of domesticated hATs and relevant genes differentially expressed across the indicated *P. waltl* tissues.

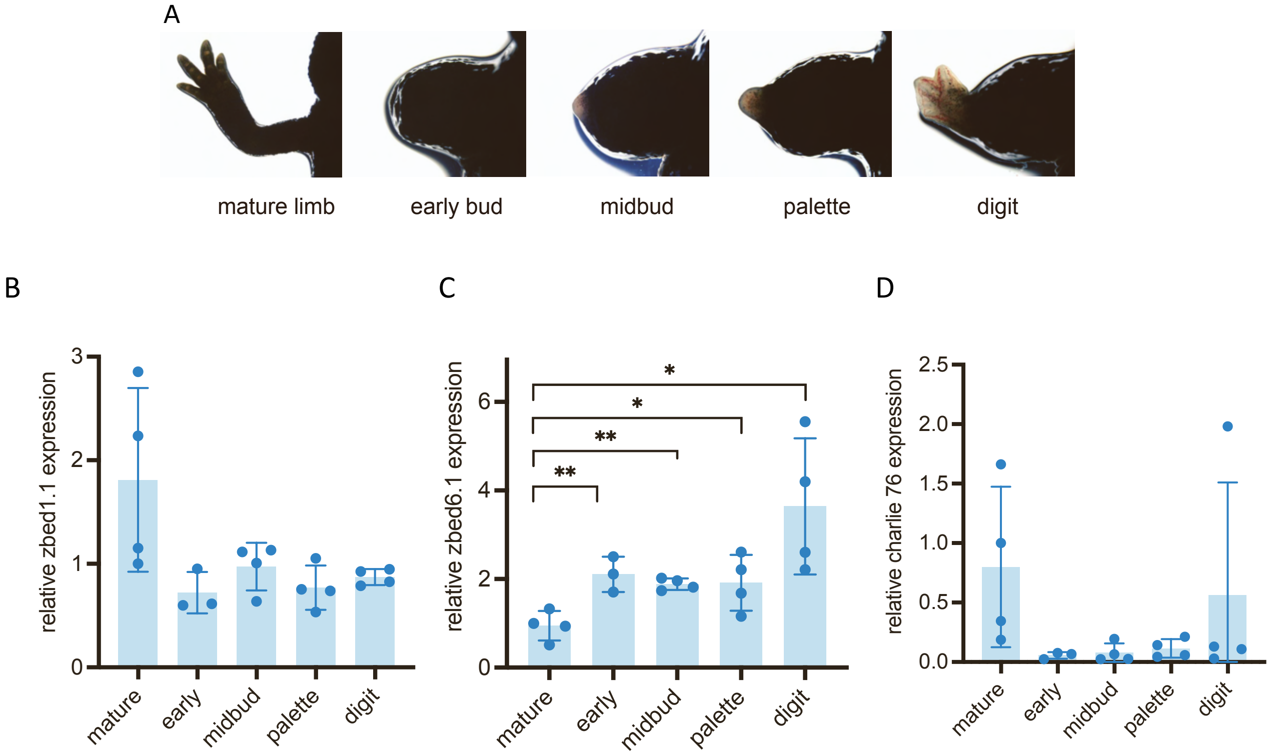

Figure S19. Relative expression of classical and domesticated hAT genes during *P. waltl* limb regeneration, related to Figure 3.

(A) Representative images of selected stages during limb regeneration. (B) qRT-PCR quantification of gene expression for the indicated domesticated (*Zbed1* (B), *Zbed6* (C)) and classical hAT (Charlie 76 (D)) relative to *Ef1a*. **p<0.001 (Welch ANOVA test followed by t-test individual comparison). Error bars represent SD.

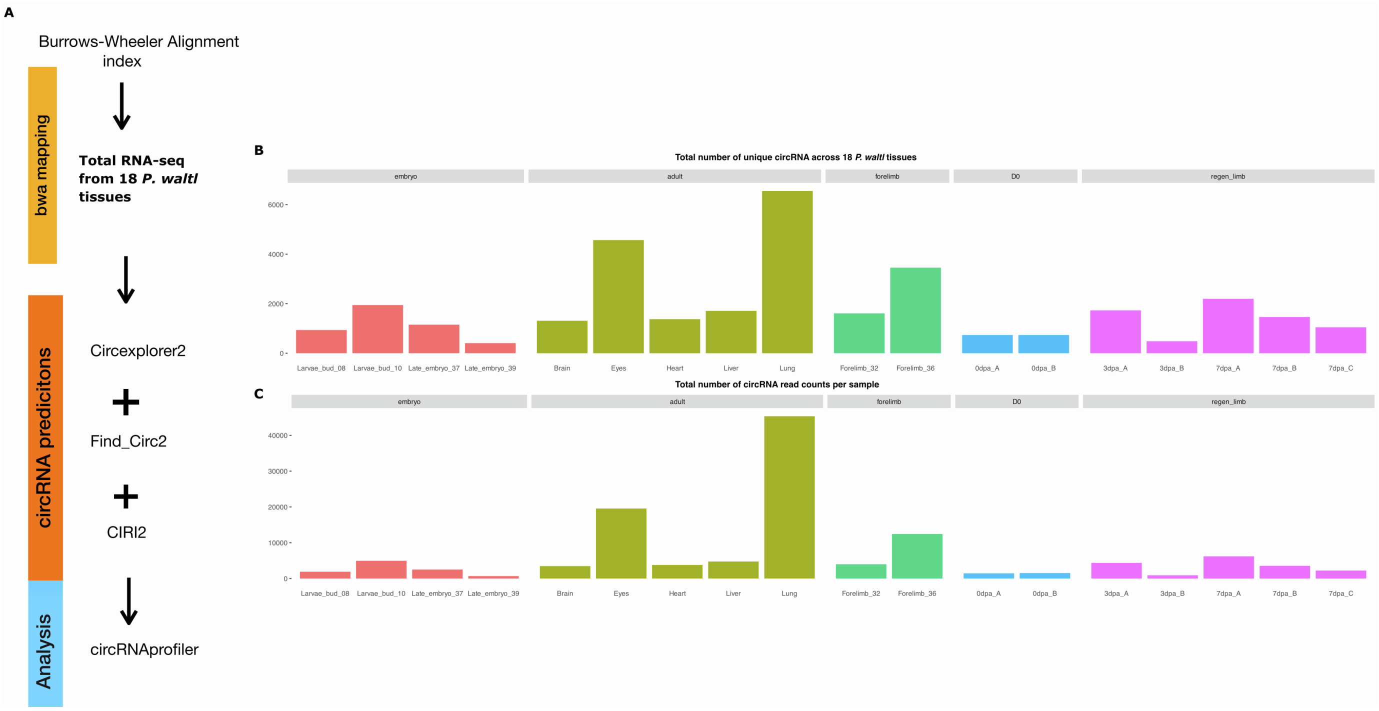

Figure S20. CircRNA detection pipeline and summary statistics, related to Figure 4.

Pipeline for detection and analysis of circRNAs (A). CircRNA summary statistics including (B) number of unique circRNAs across samples [S1] and (C) total number of circRNA counts.

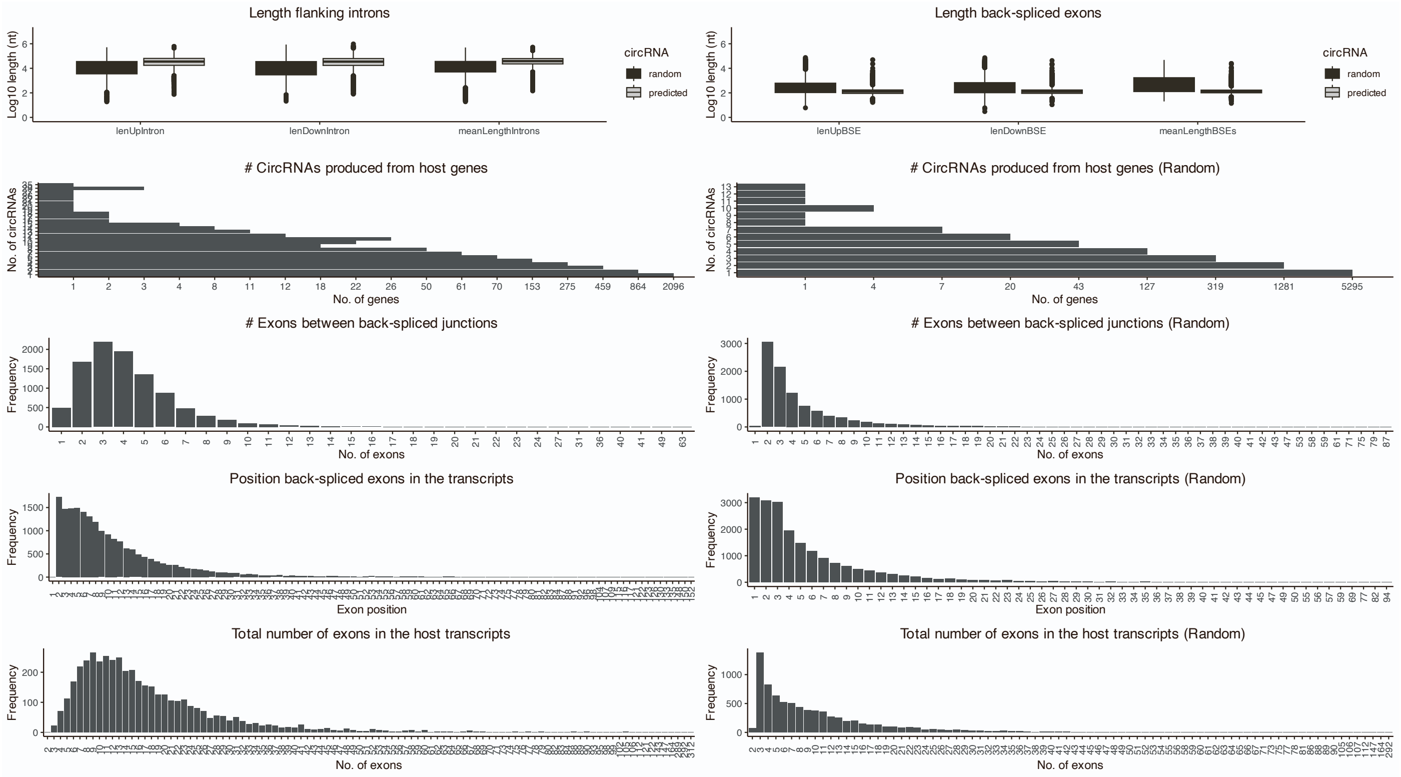

Figure S21. Features of circRNAs, related to Figure 4.

Features of predicted circRNAs (light grey in top graphs, left in bottom four panels) compared to randomly permutated back-spliced junctions (dark grey in top graphs, right in bottom four panels), n = 9799.

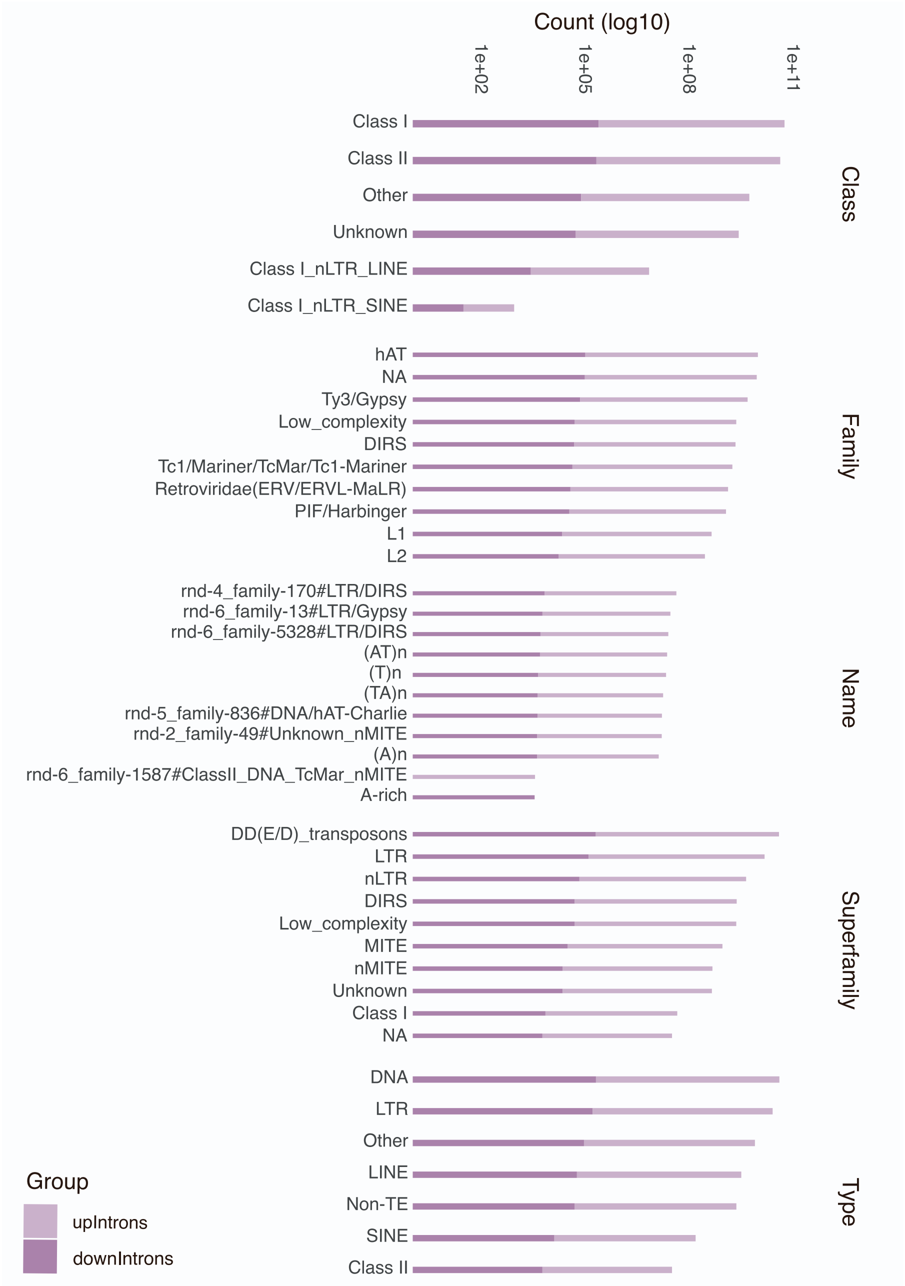

Figure S22. Repeat elements in circRNA flanking introns, related to Figure 4.

Stacked bar plot of repeat elements embedded in down- and up-stream flanking introns of circRNAs. Top ten repeat elements are shown further classified under Class, Family, Name, Superfamily or Type.

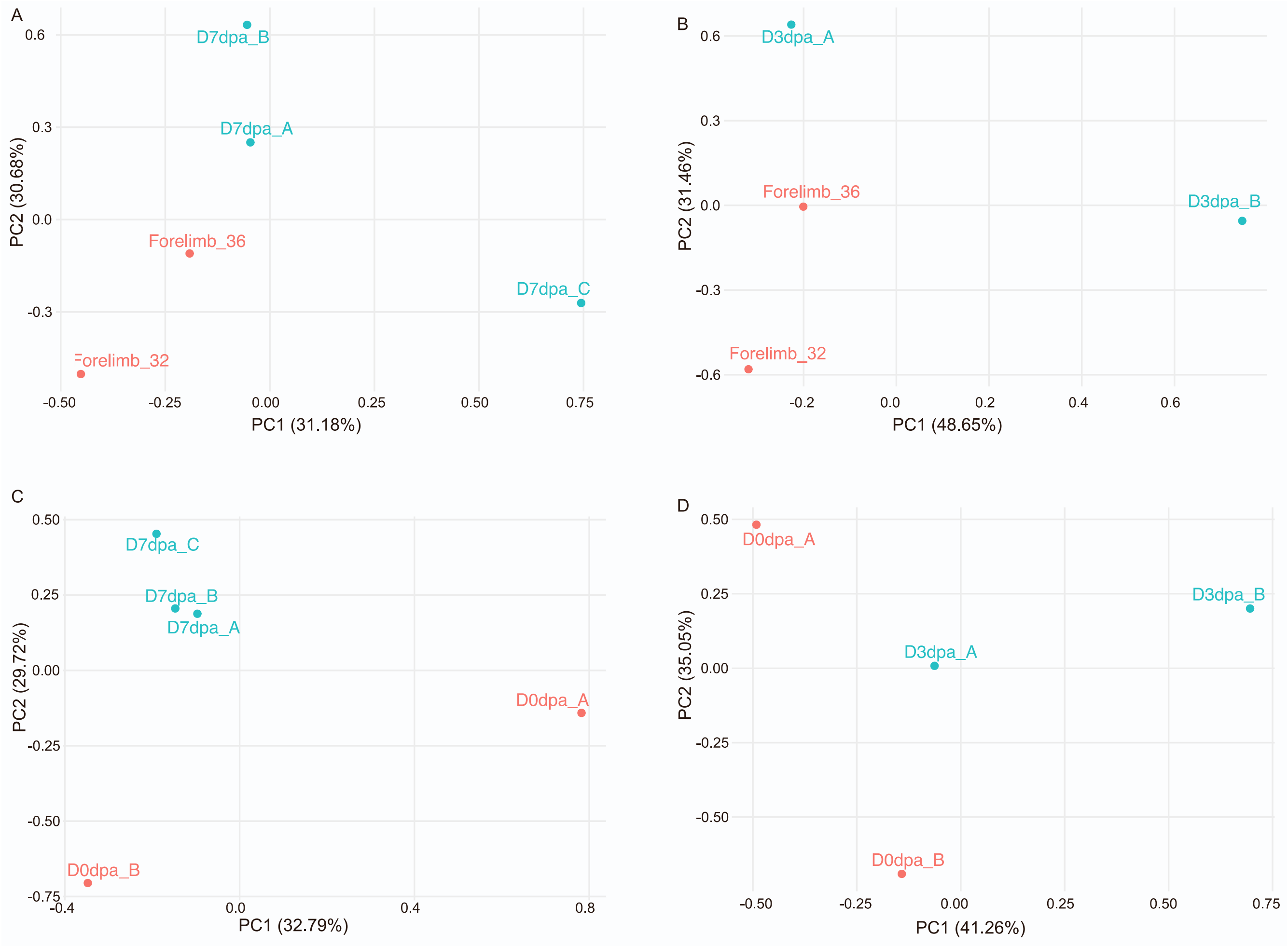

Figure S23. Principal component analysis plot based on circRNA expression across adult limb tissue groups, related to Figure 4.

(A) Forelimb and D7, (B) Forelimb and D3, (C) D0 and D7 and (D) D0 and D3. Forelimb (uninjured limb, n=2); D0 limb stump tissue (n=2); regenerating limb (n= 2 of 3dpa and n = 3 of 7dpa). The naming of the samples in the figures is based on [S1].

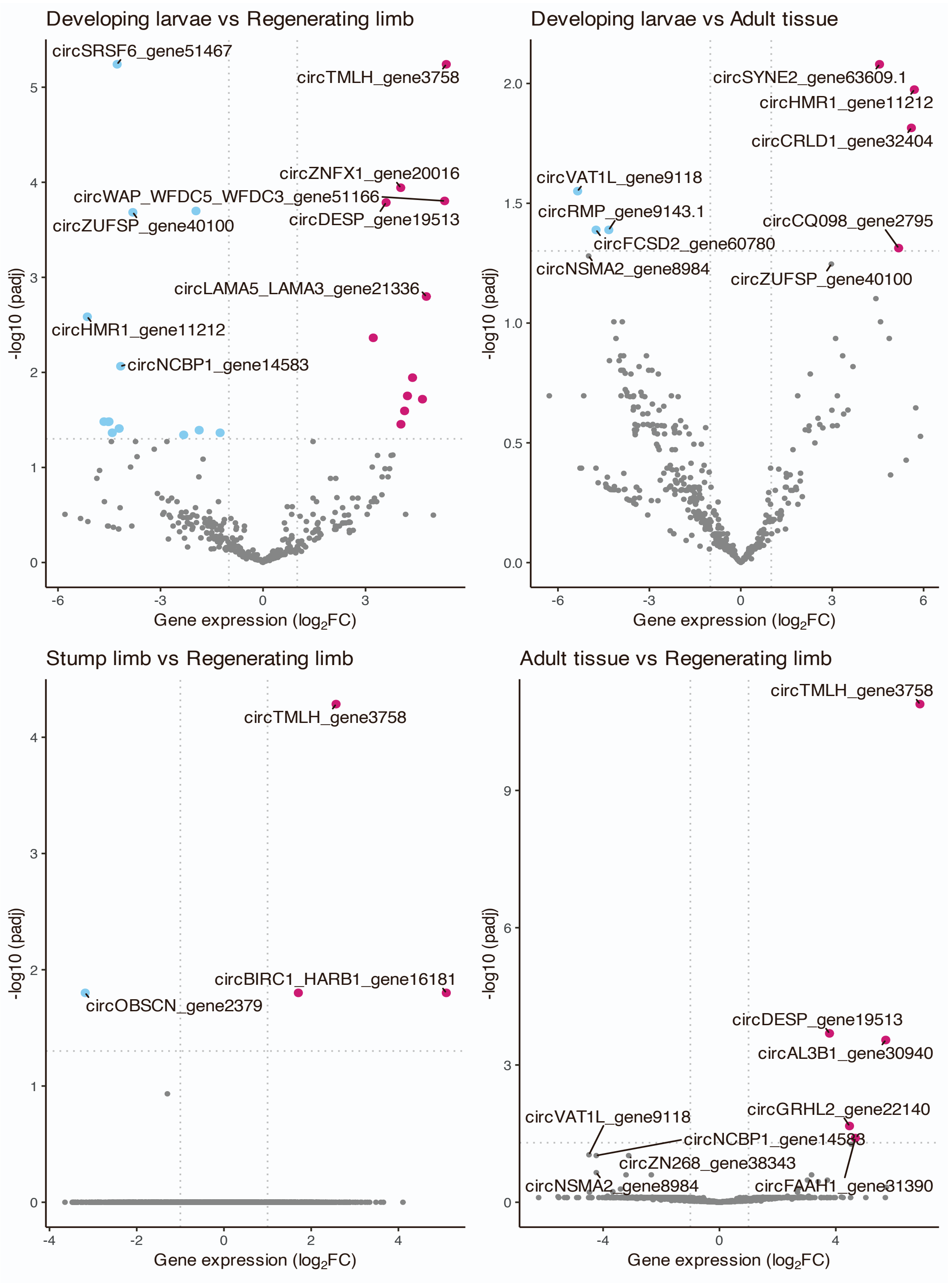

Figure S24. Volcano plots of pairwise circRNA differential expression, related to Figure 4.

Volcano plots depicting differential circRNAs expression detected pairwise between various tissue groups, labelled with full gene id. Developing larvae versus Regenerating limb volcano plot corresponds to Figure 4e.

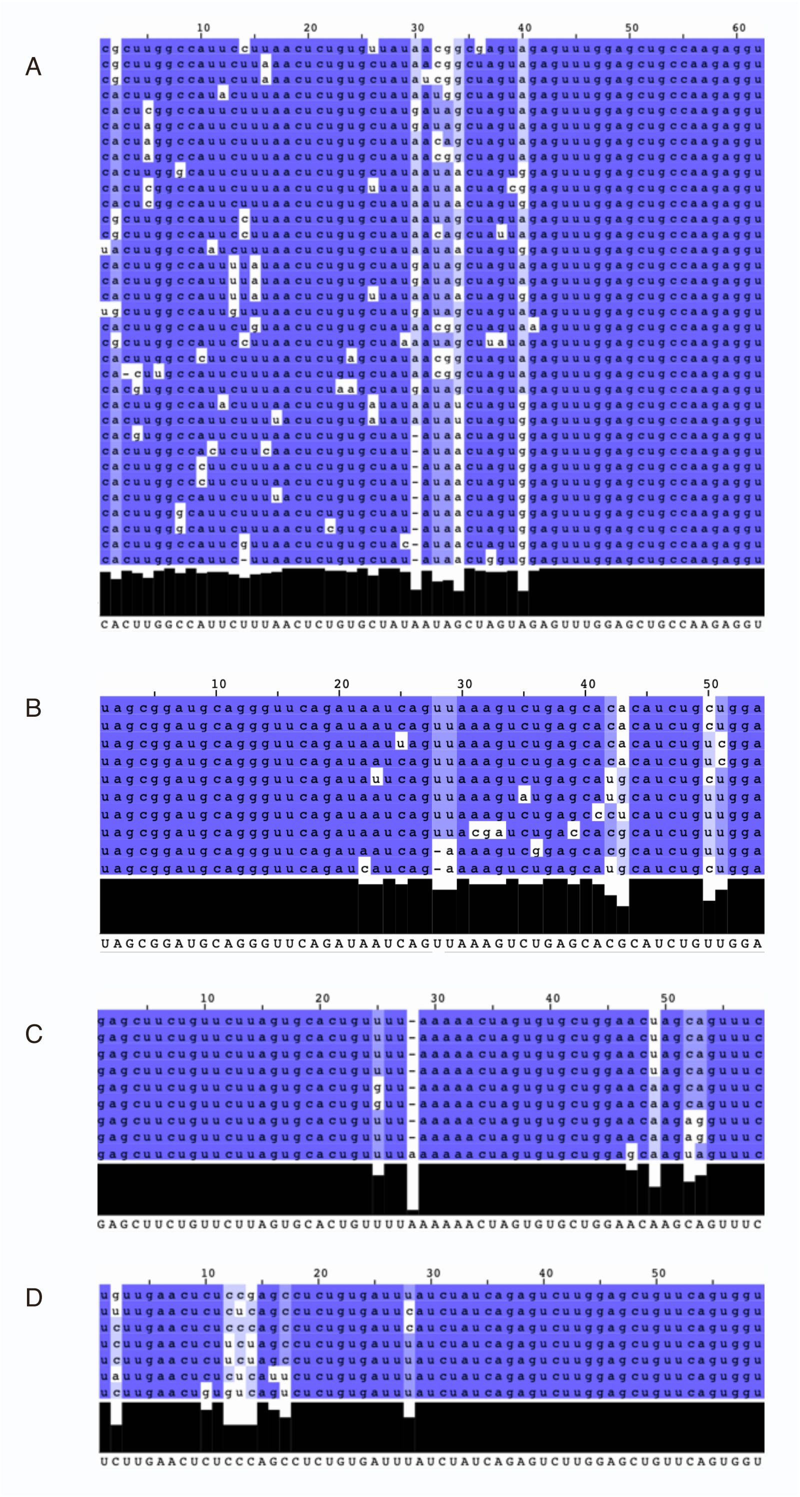

Figure S25. Multiple sequence alignment of pwa-miR precursors, related to Figure 6.

(A) Group 1 consists of 34 identical miRNAs, (B) Group 2 consists of ten, (C) Group 3 has nine and, (D) Group 4 contains seven identical mature miRNA copies.

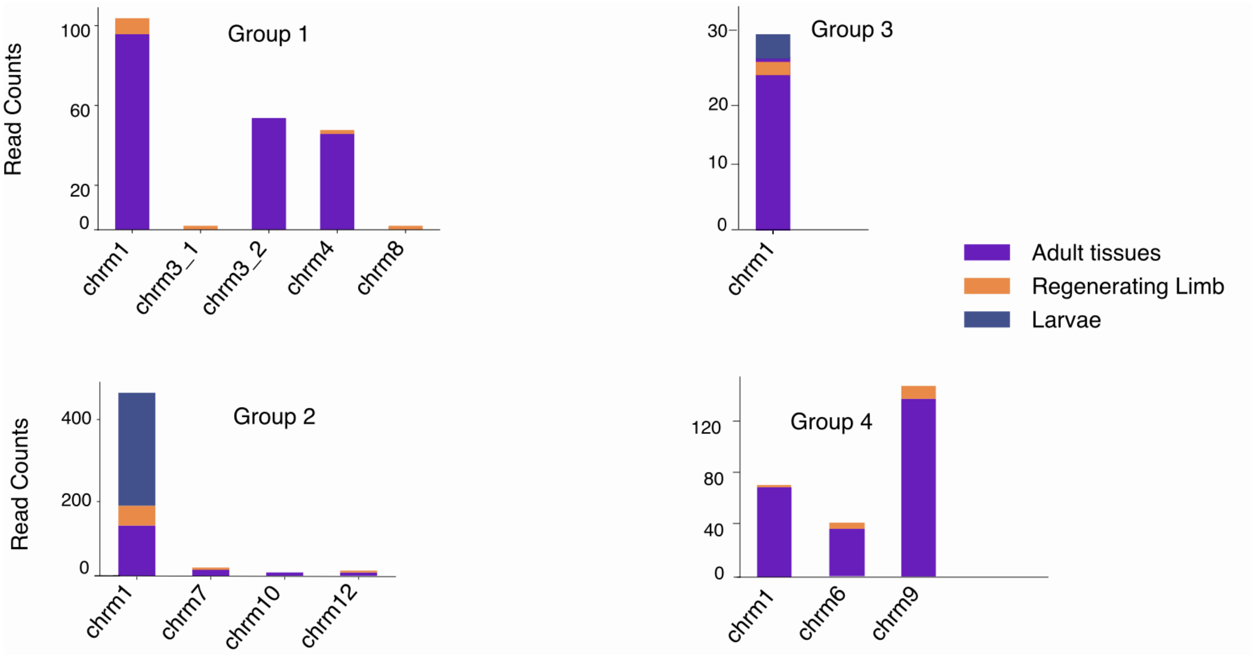

Figure S26. Reads uniquely mapped to pwa-miRs across tissues, related to Figure 6.
Stacked bar plot showing uniquely mapped reads of the four pwa-miR groups across tissues. Different copies of the same pwa-miR groups are distinguished by chromosome location.

References

S1. Elewa, A., Wang, H., Talavera-López, C., Joven, A., Brito, G., Kumar, A., Hameed, L.S., Penrad-Mobayed, M., Yao, Z., Zamani, N., et al. (2017). Reading and editing the Pleurodeles waltl genome reveals novel features of tetrapod regeneration. Nature Communications 8, 2286. 10.1038/s41467-017-01964-9.

S2. Wang, K., Wang, J., Zhu, C., Yang, L., Ren, Y., Ruan, J., Fan, G., Hu, J., Xu, W., Bi, X., et al. (2021). African lungfish genome sheds light on the vertebrate water-to-land transition. Cell 184, 1362-1376.e1318. 10.1016/j.cell.2021.01.047.

S3. Schloissnig, S., Kawaguchi, A., Nowoshilow, S., Falcon, F., Otsuki, L., Tardivo, P., Timoshevskaya, N., Keinath, M.C., Smith, J.J., Voss, S.R., and Tanaka, E.M. (2021). The giant axolotl genome uncovers the evolution, scaling, and transcriptional control of complex gene loci. Proceedings of the National Academy of Sciences *118*, e2017176118. 10.1073/pnas.2017176118.

S4. Oliveira, C.R., Knapp, D., Elewa, A., Gerber, T., Gonzalez Malagon, S.G., Gates, P.B., Walters, H.E., Petzold, A., Arce, H., Cordoba, R.C., et al. (2022). Tig1 regulates proximo-distal identity during salamander limb regeneration. Nature Communications *13*, 1141. 10.1038/s41467-022-28755-1.
