# Supplementary figures and images for: Chromosome-scale genome assembly reveals how repeat elements shape non-coding RNA landscapes active during newt limb regeneration
Source: Cell Genom. 2025 Jan 27;5(2):100761. doi: 10.1016/j.xgen.2025.100761 (PMC11872487; doi:10.1016/j.xgen.2025.100761)

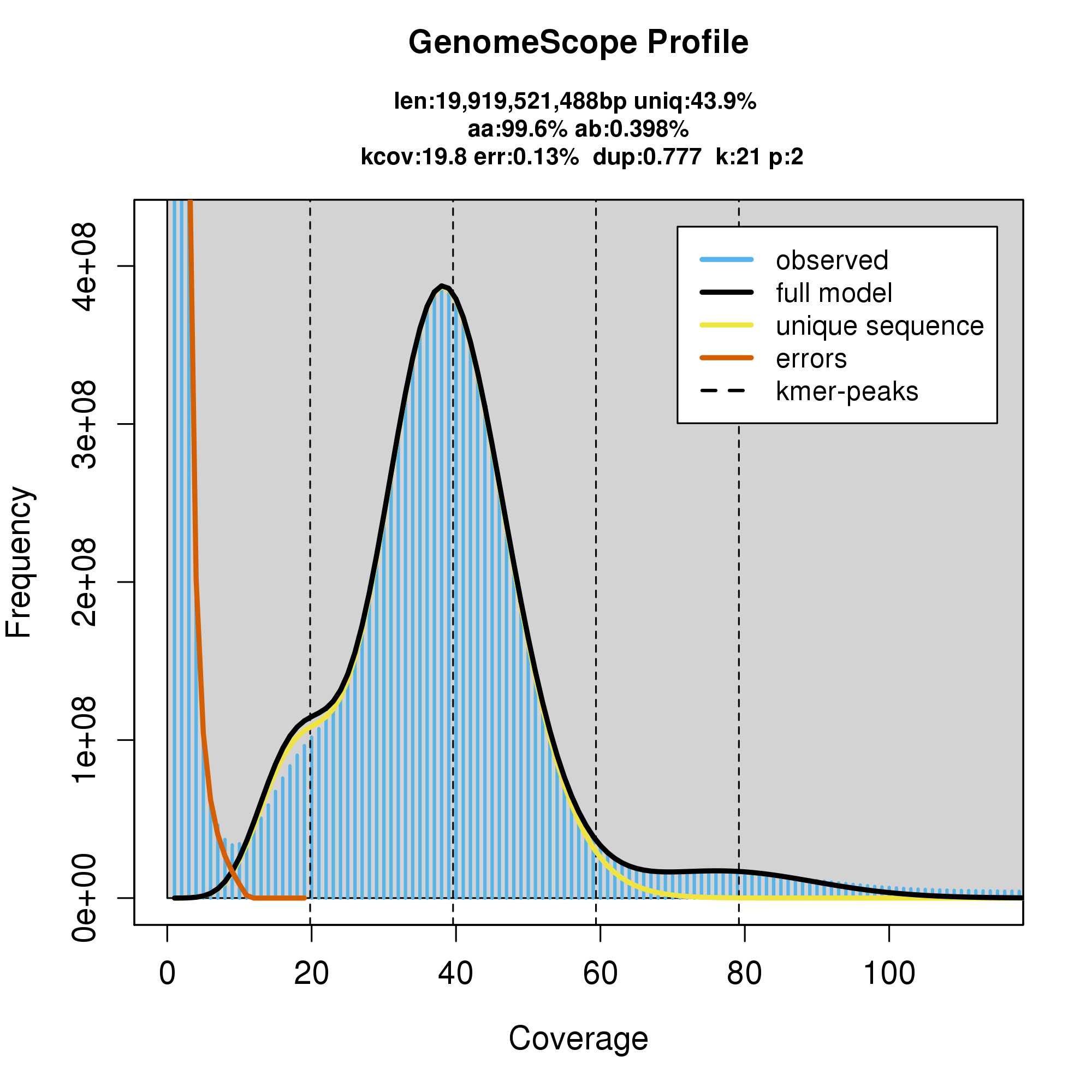

Supplement: Data S1. GenomeScope profile (k-mer-based statistical analysis) of the P. waltl genome assembly, related to Figure 1 (ab) indicates level of heterozygosity; (aa) indicates level of homozygosity [file mmc17.zip › Suppl data 1 GenomeScope_ccs.png]
