# Supplementary material for: Chromosome-scale genome assembly reveals how repeat elements shape non-coding RNA landscapes active during newt limb regeneration
Source: Cell Genom. 2025 Jan 27;5(2):100761. doi: 10.1016/j.xgen.2025.100761 (PMC11872487; doi:10.1016/j.xgen.2025.100761)
Supplement: Data S7. An unfiltered summary of miRDeep2 output on P. waltl miRNA predictions, related to Figure 5 and Table S9 [file mmc23.zip › xgen_00100761_Suppl data 7 miRDeep2_summary.html]

miRDeep2


| **miRDeep2** | . |

## Parameters used

|  |  |
| --- | --- |
| miRDeep2 version | 2.0.1.3 |
  
| Program call | /proj/snic2021-23-675/private/frozen\_scaffolds/nya/mirdeep2\_install/mirdeep2-0.1.3/bin/miRDeep2.pl all\_like\_split.fasta aPleWal.scaffolded.splitChromosomes.masked.fa reads\_vs\_genome\_split.arf newt\_MAT3.fasta output\_xenfasta newt\_PRE3.fasta |||  |  |
| --- | --- |
| Reads | all\_like\_split.fasta |
| Genome | aPleWal.scaffolded.splitChromosomes.masked.fa |
| Mappings | reads\_vs\_genome\_split.arf |
| Reference mature miRNAs | newt\_MAT3.fasta |
| Other mature miRNAs | output\_xenfasta |

  
  

**Survey of miRDeep2 performance for score cut-offs -10 to 10**


|  | novel miRNAs | | | known miRBase miRNAs | | |  |  |
| --- | --- | --- | --- | --- | --- | --- | --- | --- |
 miRDeep2 scorefor details on how the log-odds score is calculated, see Friedlander et al., Nature Biotechnology, 2008. | predicted by miRDeep2novel miRNA hairpins are here defined by not having any of the reference mature miRNAs mapping perfectly (full length, no mismatches). The numbers show how many novel miRNA hairpins have a score equal to or exceeding the cut-off. | estimated false positivesnumber of false positive miRNA hairpins predicted at this cut-off, as estimated by the miRDeep2 controls (see Friedlander et al., Nature Biotechnology, 2008). Mean and standard deviation is estimated from 100 rounds of permuted controls. | estimated true positivesthe number of true positive miRNA hairpins is estimated as t = total novel miRNAs - false positive novel miRNAs. The percentage of the predicted novel miRNAs that is estimated to be true positives is calculated as p = t / total novel miRNAs. The number of false positives is estimated from 100 rounds of permuted controls. In each of the 100 rounds, t and p are calculated, generating mean and standard deviation of t and p. The variable p can be used as an estimation of miRDeep2 positive predictive value at the score cut-off. | in speciesnumber of reference mature miRNAs for that species given as input to miRDeep2. | in datanumber of reference mature miRNAs for that species that map perfectly (full length, no mismatches) to one or more of precursor candidates that have been excised from the genome by miRDeep2. | detected by miRDeep2number of reference mature miRNAs for that species that map perfectly (full length, no mismatches) to one or more of predicted miRNA hairpins that have a score equal to or exceeding the cut-off. The percentage of reference mature miRNAs in data that is detected by miRDeep2 is calculated as s = reference mature miRNAs detected / reference mature miRNAs in data. s can be used as an estimation of miRDeep2 sensitivity at the score cut-off. | estimated signal-to-noisefor the given score cut-off, the signal-to-noise ratio is estimated as r = total miRNA hairpins reported / mean estimated false positive miRNA hairpins over 100 rounds of permuted controls. | excision gearingthis is the minimum read stack height required for excising a potential miRNA precursor from the genome in this analysis. || 10 522 22 ± 5 500 ± 5 (96 ± 1%) 284 185 145 (78%) 25.9 6 | | | | | | | | |
| 9 534 23 ± 5 511 ± 5 (96 ± 1%) 284 185 145 (78%) 25.8 6 | | | | | | | | |
| 8 552 23 ± 5 529 ± 5 (96 ± 1%) 284 185 146 (79%) 26 6 | | | | | | | | |
| 7 574 24 ± 5 550 ± 5 (96 ± 1%) 284 185 148 (80%) 26.1 6 | | | | | | | | |
| 6 592 25 ± 5 567 ± 5 (96 ± 1%) 284 185 148 (80%) 26 6 | | | | | | | | |
| 5 617 27 ± 5 590 ± 5 (96 ± 1%) 284 185 168 (91%) 25.8 6 | | | | | | | | |
| 4 640 29 ± 6 611 ± 6 (95 ± 1%) 284 185 177 (96%) 24.8 6 | | | | | | | | |
| 3 654 44 ± 6 610 ± 6 (93 ± 1%) 284 185 178 (96%) 17.7 6 | | | | | | | | |
| 2 762 60 ± 7 702 ± 7 (92 ± 1%) 284 185 178 (96%) 14.7 6 | | | | | | | | |
| 1 1275 118 ± 11 1157 ± 11 (91 ± 1%) 284 185 179 (97%) 11.3 6 | | | | | | | | |
| 0 1506 384 ± 17 1122 ± 17 (75 ± 1%) 284 185 179 (97%) 4.3 6 | | | | | | | | |
| -1 1648 760 ± 23 888 ± 23 (54 ± 1%) 284 185 179 (97%) 2.4 6 | | | | | | | | |
| -2 1745 1048 ± 29 697 ± 29 (40 ± 2%) 284 185 180 (97%) 1.8 6 | | | | | | | | |
| -3 1954 1323 ± 31 631 ± 31 (32 ± 2%) 284 185 180 (97%) 1.6 6 | | | | | | | | |
| -4 2198 1554 ± 34 644 ± 34 (29 ± 2%) 284 185 180 (97%) 1.5 6 | | | | | | | | |
| -5 2417 1790 ± 33 627 ± 33 (26 ± 1%) 284 185 180 (97%) 1.4 6 | | | | | | | | |
| -6 2558 2023 ± 34 535 ± 34 (21 ± 1%) 284 185 180 (97%) 1.4 6 | | | | | | | | |
| -7 2689 2180 ± 34 509 ± 34 (19 ± 1%) 284 185 180 (97%) 1.3 6 | | | | | | | | |
| -8 2829 2293 ± 35 536 ± 35 (19 ± 1%) 284 185 180 (97%) 1.3 6 | | | | | | | | |
| -9 2948 2375 ± 36 573 ± 36 (19 ± 1%) 284 185 180 (97%) 1.3 6 | | | | | | | | |
| -10 3046 2438 ± 36 608 ± 36 (20 ± 1%) 284 185 180 (97%) 1.3 6 | | | | | | | | |

  
  
  
  
  

## novel miRNAs predicted by miRDeep2

  


provisional idthis is a provisional miRNA name assigned by miRDeep2. The first part of the id designates the chromosome or genome contig on which the miRNA gene is located. The second part is a running number that is added to avoid identical ids. The running number is incremented by one for each potential miRNA precursor that is excised from the genome. Clicking this field will display a pdf of the structure, read signature and score breakdown of the reported miRNA. | miRDeep2 scorethe log-odds score assigned to the hairpin by miRDeep2 | estimated probability that the miRNA candidate is a true positivethe estimated probability that a predicted novel miRNA with a score of this or higher is a true positive. To see exactly how this probability is estimated, mouse over the 'novel miRNAs, true positives' in the table at the top of the webpage. | rfam alertthis field indicates if the predicted miRNA hairpin has sequence similarity to reference rRNAs or tRNAs. Warnings in this field should overrule the estimated probability that a reported miRNA is a true positive (previous field). | total read countthis is the sum of read counts for the predicted mature, loop and star miRNAs. | mature read countthis is the number of reads that map to the predicted miRNA hairpin and are contained in the sequence covered by the predicted mature miRNA, including 2 nts upstream and 5 nts downstream. | loop read countthis is the number of reads that map to the predicted miRNA hairpin and are contained in the sequence covered by the predicted miRNA loop, including 2 nts upstream and 5 nts downstream. | star read countthis is the number of reads that map to the predicted miRNA hairpin and are contained in the sequence covered by the predicted star miRNA, including 2 nts upstream and 5 nts downstream. | significant randfold p-valuethis field indicates if the estimated randfold p-value of the excised potential miRNA hairpin is equal to or lower than 0.05 (see Bonnet et al., Bioinformatics, 2004). | miRBase miRNAthis field displays the ids of any reference mature miRNAs for the species that map perfectly (full length, no mismatches) to the reported miRNA hairpin. If this is the case, the reported miRNA hairpin is assigned as a known miRNA. If not, it is assigned as a novel miRNA. If more than one reference mature miRNA maps to the miRNA hairpin, then only the id of the reference miRBase miRNA that matches the predicted mature sequence is output. | example miRBase miRNA with the same seedthis field displays the ids of any reference mature miRNAs from related species that have a seed sequence identical to that of the reported mature miRNA. The seed is here defined as nucleotides 2-8 from the 5' end of the mature miRNA. If more than one reference mature miRNA have identical seed, then only the id of the miRNA that occurs last in the input file of reference mature miRNAs from related species is displayed. | UCSC browserif a species name was input to miRDeep2, then clicking this field will initiate a UCSC blat search of the consensus precursor sequence against the reference genome. | NCBI blastnclicking this field will initiate a NCBI blastn search of the consensus precursor sequence against the nr/nt database (non-redundant collection of all NCBI nucleotide sequences). | consensus mature sequencethis is the consensus mature miRNA sequence as inferred from the deep sequencing reads. | consensus star sequencethis is the consensus star miRNA sequence as inferred from the deep sequencing reads. | consensus precursor sequencethis is the consensus precursor miRNA sequence as inferred from the deep sequencing reads. Note that this is the inferred Drosha hairpin product, and therefore does not include substantial flanking genomic sequence as does most miRBase precursors. | precursor coordinateThe given precursor coordinates refer do absolute position in the mapped reference sequence || manual\_scaffold\_12\_38982 | 1.1e+5 | 0.96 ± 0.01 |  | 228483 | 228333 | 0 | 150 | no |  | xtr-miR-367 |  | blast | cauugcacucgucccggccgga | agguugggagagcuguaaucugc | agguugggagagcuguaaucugcgugacucuguuggauggcauugcacucgucccggccgga | manual\_scaffold\_12:56490459..56490521:- |
| manual\_scaffold\_4b\_16189 | 1.1e+5 | 0.96 ± 0.01 |  | 223532 | 223426 | 0 | 106 | yes |  |  |  | blast | acccuguagaaucgaauuugugu | acaaauucgucucuggggggu | acccuguagaaucgaauuuguguggauauuuaagagccacaaauucgucucuggggggu | manual\_scaffold\_4b:44227893..44227952:- |
| manual\_scaffold\_5\_18073 | 5.0e+4 | 0.96 ± 0.01 |  | 98496 | 79281 | 17821 | 1394 | yes |  |  |  | blast | agacgaucaaacuugacuaucua | uggaugguuuagugagguc | uggaugguuuagugagguccuuggaucggccccgccggggucggcaacggcccuggcggagcacugaaaagacgaucaaacuugacuaucua | manual\_scaffold\_5:565330601..565330693:+ |
| manual\_scaffold\_10\_33990 | 4.1e+4 | 0.96 ± 0.01 |  | 80629 | 76544 | 5 | 4080 | yes |  |  |  | blast | uugacaucaucauacuugggau | cccaagcaggcugaugucaggc | uugacaucaucauacuugggauguaggacacaaagucccaagcaggcugaugucaggc | manual\_scaffold\_10:580187476..580187534:+ |
| manual\_scaffold\_1a\_1799 | 2.9e+4 | 0.96 ± 0.01 |  | 58059 | 57968 | 0 | 91 | yes |  |  |  | blast | caucgcguuaaccggaagucu | gacuucugggugacacgacgug | caucgcguuaaccggaagucucaguuauauguaugggacuucugggugacacgacgug | manual\_scaffold\_1a:1137601234..1137601292:- |
| manual\_scaffold\_3a\_7249 | 2.5e+4 | 0.96 ± 0.01 |  | 50468 | 50329 | 0 | 139 | no |  | xtr-miR-375 |  | blast | uuuguucguucggcucgcguu | acgucgagccaaacgugcaaugc | acgucgagccaaacgugcaaugccugacgcaacguuuuguucguucggcucgcguu | manual\_scaffold\_3a:44981683..44981739:+ |
| manual\_scaffold\_10\_34114 | 1.7e+4 | 0.96 ± 0.01 |  | 33780 | 33746 | 0 | 34 | no |  |  |  | blast | ccaggccugagauuuucccucu | agggcgaucacagucuugagu | ccaggccugagauuuucccucuguauugcucaaauggagggcgaucacagucuugagu | manual\_scaffold\_10:744621588..744621646:+ |
| manual\_scaffold\_2a\_4984 | 1.4e+4 | 0.96 ± 0.01 |  | 28954 | 7137 | 0 | 21817 | yes |  |  |  | blast | cguguauuugacaagcugagcu | ugucaguuugucaaauac | cguguauuugacaagcugagcuugacacucaaauugguagagugucaguuugucaaauac | manual\_scaffold\_2a:98511596..98511656:- |
| manual\_scaffold\_10\_35580 | 1.4e+4 | 0.96 ± 0.01 |  | 28506 | 27801 | 2 | 703 | yes |  |  |  | blast | ugacaucauguguacggcugcu | uggucguauguaugacgucauu | uggucguauguaugacgucauuuacuugggguuuuuggagugacaucauguguacggcugcu | manual\_scaffold\_10:923703005..923703067:- |
| manual\_scaffold\_8\_30053 | 1.3e+4 | 0.96 ± 0.01 |  | 26760 | 26232 | 4 | 524 | no |  | xtr-miR-16a |  | blast | uagcagcacguaaugguuugu | caggccauacuguacugccu | uagcagcacguaaugguuugugggguguuauauaaaagcacaggccauacuguacugccu | manual\_scaffold\_8:420347172..420347232:- |
| manual\_scaffold\_3b\_10211 | 1.3e+4 | 0.96 ± 0.01 |  | 25837 | 25644 | 0 | 193 | yes |  |  |  | blast | ucugagcacgcaucuguugga | uagcagaugcaggguucagau | uagcagaugcaggguucagauaaucagaaacgucugagcacgcaucuguugga | manual\_scaffold\_3b:237254032..237254085:+ |
| manual\_scaffold\_2a\_5682 | 1.3e+4 | 0.96 ± 0.01 |  | 25651 | 25644 | 0 | 7 | yes |  |  |  | blast | ucugagcacgcaucuguugga | uaguggaugcaggguucagauaau | uaguggaugcaggguucagauaaucaguuaaacgucugagcacgcaucuguugga | manual\_scaffold\_2a:1117158963..1117159018:- |
| manual\_scaffold\_6\_22654 | 1.2e+4 | 0.96 ± 0.01 |  | 24887 | 24715 | 1 | 171 | yes |  |  |  | blast | acuuggacucucuuagacucu | aguauuggagcugucuuguggu | acuuggacucucuuagacucugugacauucuaaucagaguauuggagcugucuuguggu | manual\_scaffold\_6:1763450795..1763450854:+ |
| manual\_scaffold\_9\_33245 | 1.0e+4 | 0.96 ± 0.01 |  | 19732 | 19720 | 0 | 12 | yes |  |  |  | blast | gacagucaggaguuggucuggc | cagaucagcuuuuggcugugc | gacagucaggaguuggucuggcgugaucccccaaucucagaucagcuuuuggcugugc | manual\_scaffold\_9:1182338199..1182338257:- |
| manual\_scaffold\_11\_35850 | 8.3e+3 | 0.96 ± 0.01 |  | 16424 | 8327 | 0 | 8097 | yes |  |  |  | blast | caccugaaauuaugucugaaau | uucagucauuguuucuggugga | caccugaaauuaugucugaaaugaggaauuagaaaauuucagucauuguuucuggugga | manual\_scaffold\_11:7292395..7292454:+ |
| manual\_scaffold\_10\_35114 | 6.5e+3 | 0.96 ± 0.01 |  | 12801 | 12416 | 0 | 385 | yes |  |  |  | blast | ucgccgucacauuaguccucacu | uguggacuugagagagggcaau | uguggacuugagagagggcaauguucuuuucaucgccgucacauuaguccucacu | manual\_scaffold\_10:470039431..470039486:- |
| manual\_scaffold\_3a\_8506 | 6.0e+3 | 0.96 ± 0.01 |  | 11951 | 5974 | 0 | 5977 | yes |  | xtr-miR-449c-5p |  | blast | aggcaguguaguuagcugauugu | aaucacuaacuguacugccauc | aggcaguguaguuagcugauugugugcuccuguuuggcaaucacuaacuguacugccauc | manual\_scaffold\_3a:794602836..794602896:+ |
| manual\_scaffold\_1b\_2223 | 6.0e+3 | 0.96 ± 0.01 |  | 11946 | 11865 | 0 | 81 | yes |  |  |  | blast | ucuugaacucucuuagccucu | aagucuuagagcuguucaguggu | ucuugaacucucuuagccucugugauuuaucuaucaaagucuuagagcuguucaguggu | manual\_scaffold\_1b:482803255..482803314:+ |
| manual\_scaffold\_10\_35135 | 4.9e+3 | 0.96 ± 0.01 |  | 9680 | 8153 | 0 | 1527 | yes |  |  |  | blast | ugaugaaaucaaccuauuggccugu | aggauagguugauuucauugac | ugaugaaaucaaccuauuggccuguuugaagauucaccacaggauagguugauuucauugac | manual\_scaffold\_10:495126268..495126330:- |
| manual\_scaffold\_4b\_16656 | 4.6e+3 | 0.96 ± 0.01 |  | 9210 | 9017 | 0 | 193 | yes |  |  |  | blast | ucugagcaugcaucuguugga | uagcagaugcaggguucagau | uagcagaugcaggguucagauaaucaguaaauucugagcaugcaucuguugga | manual\_scaffold\_4b:181431107..181431160:- |
| manual\_scaffold\_7\_26916 | 4.6e+3 | 0.96 ± 0.01 |  | 9198 | 6888 | 0 | 2310 | yes |  |  |  | blast | uccgggguacucagcacgaaga | uucguugugaguagcucggguc | uccgggguacucagcacgaagaauuaaacuucguugugaguagcucggguc | manual\_scaffold\_7:357537946..357537997:- |
| manual\_scaffold\_1b\_3786 | 4.5e+3 | 0.96 ± 0.01 |  | 9020 | 9017 | 0 | 3 | yes |  |  |  | blast | ucugagcaugcaucuguugga | uagcggaugcaagguucagauaauc | uagcggaugcaagguucagauaaucaguuaaauucugagcaugcaucuguugga | manual\_scaffold\_1b:1209455301..1209455355:- |
| manual\_scaffold\_6\_21989 | 4.5e+3 | 0.96 ± 0.01 |  | 9018 | 9017 | 0 | 1 | yes |  |  |  | blast | ucugagcaugcaucuguugga | uaguggauguagggcucagauaa | uaguggauguagggcucagauaaucaguaaauucugagcaugcaucuguugga | manual\_scaffold\_6:1205611562..1205611615:+ |
| manual\_scaffold\_8\_30202 | 4.5e+3 | 0.96 ± 0.01 |  | 9000 | 6690 | 0 | 2310 | yes |  |  |  | blast | uccgggguacucagcacgaagg | uucguugugaguagcucggguc | uccgggguacucagcacgaaggauuaaucuucguugugaguagcucggguc | manual\_scaffold\_8:674641012..674641063:- |
| manual\_scaffold\_2a\_5373 | 4.5e+3 | 0.96 ± 0.01 |  | 8904 | 8903 | 0 | 1 | yes |  |  |  | blast | auugaggcuaucuguguagc | cuacccaaguaacccauuuac | auugaggcuaucuguguagccugcuccauagaacaggcuacccaaguaacccauuuac | manual\_scaffold\_2a:602533930..602533988:- |
| manual\_scaffold\_7\_25943 | 4.4e+3 | 0.96 ± 0.01 |  | 8661 | 8535 | 0 | 126 | yes |  |  |  | blast | agcauucuggggcauuucagga | cugagacaccccagaaugauuu | cugagacaccccagaaugauuuuccucaugcaaagcauucuggggcauuucagga | manual\_scaffold\_7:1299541443..1299541498:+ |
| manual\_scaffold\_5\_18287 | 4.2e+3 | 0.96 ± 0.01 |  | 8416 | 8168 | 17 | 231 | yes |  |  |  | blast | caggcugguuagaugguuguc | gcaggcaucuucccagccuaca | gcaggcaucuucccagccuacauguggaauacuggaccugcaggcugguuagaugguuguc | manual\_scaffold\_5:885455807..885455868:+ |
| manual\_scaffold\_7\_28050 | 4.2e+3 | 0.96 ± 0.01 |  | 8366 | 4416 | 1 | 3949 | yes |  |  |  | blast | aagcccuuaccccaaaaagcgu | cuuuuugcggucugggcuu | cuuuuugcggucugggcuuucugggucuaugaaauccuaaaaucagaaagcccuuaccccaaaaagcgu | manual\_scaffold\_7:1460505479..1460505548:- |
| manual\_scaffold\_5\_17998 | 4.2e+3 | 0.96 ± 0.01 |  | 8361 | 7305 | 0 | 1056 | yes |  |  |  | blast | uccugcuacuuugaucuuucuc | gaagaucauuguaguggaaga | uccugcuacuuugaucuuucucuuuuucuacucuuugagaagaucauuguaguggaaga | manual\_scaffold\_5:473290323..473290382:+ |
| manual\_scaffold\_11\_37441 | 3.9e+3 | 0.96 ± 0.01 |  | 7675 | 7671 | 0 | 4 | no |  | xtr-miR-146b |  | blast | agagaacuaucucaaugaacugu | aguucaagaaauuaguucc | agagaacuaucucaaugaacuguccaaauggaaauugcaguucaagaaauuaguucc | manual\_scaffold\_11:743174670..743174727:- |
| manual\_scaffold\_3a\_8360 | 3.8e+3 | 0.96 ± 0.01 |  | 7577 | 7514 | 0 | 63 | yes |  |  |  | blast | ucugagcaugcaucuguugga | uaacggauacagcguucagau | uaacggauacagcguucagauaaucaguaaagucugagcaugcaucuguugga | manual\_scaffold\_3a:624517558..624517611:+ |
| manual\_scaffold\_8\_30277 | 3.5e+3 | 0.96 ± 0.01 |  | 7056 | 6722 | 0 | 334 | yes |  |  |  | blast | gagcuucuguucuuagugcacu | ugugcuggaacaagcaguuuc | gagcuucuguucuuagugcacuguguuaaaaacuagugugcuggaacaagcaguuuc | manual\_scaffold\_8:788138786..788138843:- |
| manual\_scaffold\_10\_33800 | 3.5e+3 | 0.96 ± 0.01 |  | 7056 | 6722 | 0 | 334 | yes |  |  |  | blast | gagcuucuguucuuagugcacu | ugugcuggaacaagcaguuuc | gagcuucuguucuuagugcacuguguuaaaaacuagugugcuggaacaagcaguuuc | manual\_scaffold\_10:298320511..298320568:+ |
| manual\_scaffold\_10\_35730 | 3.5e+3 | 0.96 ± 0.01 |  | 6964 | 6724 | 0 | 240 | yes |  |  |  | blast | gagcuucuguucuuagugcacu | ugugcuggaacaagagguuuc | gagcuucuguucuuagugcacuguuuuaaaaacuagugugcuggaacaagagguuuc | manual\_scaffold\_10:997016111..997016168:- |
| manual\_scaffold\_10\_35732 | 3.5e+3 | 0.96 ± 0.01 |  | 6964 | 6724 | 0 | 240 | yes |  |  |  | blast | gagcuucuguucuuagugcacu | ugugcuggaacaagagguuuc | gagcuucuguucuuagugcacuguuuuaaaaacuagugugcuggaacaagagguuuc | manual\_scaffold\_10:997022090..997022147:- |
| manual\_scaffold\_8\_29453 | 3.4e+3 | 0.96 ± 0.01 |  | 6770 | 6724 | 0 | 46 | yes |  |  |  | blast | gagcuucuguucuuagugcacu | ugugcuggagcaaguaguuuc | gagcuucuguucuuagugcacuguuuuaaaaaacuagugugcuggagcaaguaguuuc | manual\_scaffold\_8:1307258926..1307258984:+ |
| manual\_scaffold\_5\_19606 | 3.4e+3 | 0.96 ± 0.01 |  | 6744 | 6724 | 0 | 20 | yes |  |  |  | blast | gagcuucuguucuuagugcacu | ugugcuggaacuagcaguuuc | gagcuucuguucuuagugcacuguuuuaaaaacuagugugcuggaacuagcaguuuc | manual\_scaffold\_5:602378359..602378416:- |
| manual\_scaffold\_4b\_16986 | 3.4e+3 | 0.96 ± 0.01 |  | 6744 | 6724 | 0 | 20 | yes |  |  |  | blast | gagcuucuguucuuagugcacu | ugugcuggaacuagcaguuuc | gagcuucuguucuuagugcacuguuuuaaaaacuagugugcuggaacuagcaguuuc | manual\_scaffold\_4b:631866799..631866856:- |
| manual\_scaffold\_6\_23338 | 3.4e+3 | 0.96 ± 0.01 |  | 6744 | 6724 | 0 | 20 | yes |  |  |  | blast | gagcuucuguucuuagugcacu | ugugcuggaacuagcaguuuc | gagcuucuguucuuagugcacuguuuuaaaaacuagugugcuggaacuagcaguuuc | manual\_scaffold\_6:315363029..315363086:- |
| manual\_scaffold\_2b\_6001 | 3.4e+3 | 0.96 ± 0.01 |  | 6744 | 6724 | 0 | 20 | yes |  |  |  | blast | gagcuucuguucuuagugcacu | ugugcuggaacuagcaguuuc | gagcuucuguucuuagugcacuguuuuaaaaacuagugugcuggaacuagcaguuuc | manual\_scaffold\_2b:113208361..113208418:+ |
| manual\_scaffold\_7\_26652 | 3.4e+3 | 0.96 ± 0.01 |  | 6718 | 6690 | 0 | 28 | yes |  |  |  | blast | uccgggguacucagcacgaagg | uucguucugaguagcucggguc | uccgggguacucagcacgaaggauuaauauucguucugaguagcucggguc | manual\_scaffold\_7:121644550..121644601:- |
| manual\_scaffold\_8\_30782 | 3.4e+3 | 0.96 ± 0.01 |  | 6707 | 6690 | 0 | 17 | yes |  |  |  | blast | uccgggguacucagcacgaagg | uucguggugaguagcucggguc | uccgggguacucagcacgaaggauuaauauucguggugaguagcucggguc | manual\_scaffold\_8:1476552051..1476552102:- |
| manual\_scaffold\_2a\_5808 | 3.4e+3 | 0.96 ± 0.01 |  | 6707 | 6690 | 0 | 17 | yes |  |  |  | blast | uccgggguacucagcacgaagg | uucguggugaguagcucggguc | uccgggguacucagcacgaaggauuaauauucguggugaguagcucggguc | manual\_scaffold\_2a:1337951649..1337951700:- |
| manual\_scaffold\_8\_29071 | 3.4e+3 | 0.96 ± 0.01 |  | 6693 | 6690 | 0 | 3 | no |  |  |  | blast | uccgggguacucagcacgaagg | uugguugugaguagcucggguc | uccgggguacucagcacgaaggauuaauauugguugugaguagcucggguc | manual\_scaffold\_8:720475300..720475351:+ |
| manual\_scaffold\_8\_30840 | 3.3e+3 | 0.96 ± 0.01 |  | 6532 | 6531 | 0 | 1 | no |  |  |  | blast | uccgucuucucugaguugaguu | cucaucccagguggaggcgugga | cucaucccagguggaggcguggagcagauuauaagagucuccgucuucucugaguugaguu | manual\_scaffold\_8:1577929449..1577929510:- |
| manual\_scaffold\_1a\_1830 | 3.2e+3 | 0.96 ± 0.01 |  | 6334 | 6247 | 35 | 52 | yes |  |  |  | blast | uuuccugcagugugacauccu | ugauuucacagagcaggaagc | ugauuucacagagcaggaagcagugggauccauuguuugaaauaauguuuccugcagugugacauccu | manual\_scaffold\_1a:1200706433..1200706501:- |
| manual\_scaffold\_1a\_1177 | 3.0e+3 | 0.96 ± 0.01 |  | 6058 | 6056 | 0 | 2 | yes |  |  |  | blast | uucuagaaguuguuggauacu | ggacccaacuucuucuagauuu | uucuagaaguuguuggauacuaggaguucuaucacuggacccaacuucuucuagauuu | manual\_scaffold\_1a:379572746..379572804:- |
| manual\_scaffold\_3a\_9051 | 3.0e+3 | 0.96 ± 0.01 |  | 5886 | 5191 | 1 | 694 | yes |  | xtr-miR-2184 |  | blast | aacaguaagaauuaaugugcug | gcacauuaucuccuacuguuaa | aacaguaagaauuaaugugcugugaaauaaaaauuaucagcacauuaucuccuacuguuaa | manual\_scaffold\_3a:314918099..314918160:- |
| manual\_scaffold\_10\_33441 | 2.9e+3 | 0.96 ± 0.01 |  | 5761 | 5737 | 0 | 24 | yes |  | xtr-miR-17-5p |  | blast | gaaagugcuguucuguugggac | gcccaaacaccagcacuuccu | gcccaaacaccagcacuuccuuuguuuucacccauaagaaagugcuguucuguugggac | manual\_scaffold\_10:48079129..48079188:+ |
| manual\_scaffold\_5\_19197 | 2.8e+3 | 0.96 ± 0.01 |  | 5628 | 5551 | 0 | 77 | yes |  |  |  | blast | ucugagcccgcaucuguugga | caaacagaugcaagguucagau | caaacagaugcaagguucagauaauaaguaaagucugagcccgcaucuguugga | manual\_scaffold\_5:81178445..81178499:- |
| manual\_scaffold\_4b\_15158 | 2.2e+3 | 0.96 ± 0.01 |  | 4403 | 4345 | 0 | 58 | yes |  |  |  | blast | ucgagaacaacugccaacuacu | uggguugagaguuguucucgga | ucgagaacaacugccaacuacuggucugguguccuggguugagaguuguucucgga | manual\_scaffold\_4b:279834292..279834348:+ |
| manual\_scaffold\_5\_19529 | 2.2e+3 | 0.96 ± 0.01 |  | 4357 | 3750 | 0 | 607 | yes |  |  |  | blast | ugaggcuucccacuucaaggcu | uccuggaaacuggaagccuaga | ugaggcuucccacuucaaggcuguguaaugacucauccuggaaacuggaagccuaga | manual\_scaffold\_5:500580880..500580937:- |
| manual\_scaffold\_10\_34656 | 2.0e+3 | 0.96 ± 0.01 |  | 3957 | 3792 | 12 | 153 | yes |  |  |  | blast | ccccugcuccgccacuaucga | gauggugucgaaggcagcggaca | gauggugucgaaggcagcggacagauuuaauaaaauuacugccccugcuccgccacuaucga | manual\_scaffold\_10:105946058..105946120:- |
| manual\_scaffold\_6\_23983 | 2.0e+3 | 0.96 ± 0.01 |  | 3947 | 3901 | 0 | 46 | yes |  |  |  | blast | gaaaucaagguuggguugagccu | gcgacccacacuugguuucua | gaaaucaagguuggguugagccuauucagcuacauacaggcgacccacacuugguuucua | manual\_scaffold\_6:1059939344..1059939404:- |
| manual\_scaffold\_2b\_6678 | 1.9e+3 | 0.96 ± 0.01 |  | 3846 | 3843 | 0 | 3 | yes |  |  |  | blast | acaccugcacugauaucccccu | ggugguauccugcauaggugu | ggugguauccugcauaggugugggguauuuacagaaauuaccacaccugcacugauaucccccu | manual\_scaffold\_2b:218847661..218847725:- |
| manual\_scaffold\_3a\_8508 | 1.8e+3 | 0.96 ± 0.01 |  | 3695 | 1943 | 0 | 1752 | yes |  |  |  | blast | aaucacuaacuacacagccagg | aggcaguguaguuagcugauuug | aggcaguguaguuagcugauuugacacugguaaauaaucacuaacuacacagccagg | manual\_scaffold\_3a:794605547..794605604:+ |
| manual\_scaffold\_5\_18975 | 1.8e+3 | 0.96 ± 0.01 |  | 3689 | 3394 | 0 | 295 | yes |  |  |  | blast | ucccggcugauuucugcaaau | ugcagaaaucugccgggacacc | ugcagaaaucugccgggacaccugcugaaaaaccgggacugucccggcugauuucugcaaau | manual\_scaffold\_5:1790273222..1790273284:+ |
| manual\_scaffold\_2b\_6305 | 1.8e+3 | 0.96 ± 0.01 |  | 3622 | 2498 | 0 | 1124 | yes |  |  |  | blast | ucugagcacacaucuguuaga | uagcggaugcaggguucagau | uagcggaugcaggguucagauaaucaauuaaauucugagcacacaucuguuaga | manual\_scaffold\_2b:551663241..551663295:+ |
| manual\_scaffold\_2a\_5161 | 1.8e+3 | 0.96 ± 0.01 |  | 3622 | 3587 | 0 | 35 | yes |  |  |  | blast | cccucauuguacaugcugugua | cacagcgcaugcaauguggac | cccucauuguacaugcuguguagauuucuauacauacacagcgcaugcaauguggac | manual\_scaffold\_2a:342477405..342477462:- |
| manual\_scaffold\_2b\_6769 | 1.5e+3 | 0.96 ± 0.01 |  | 2987 | 2976 | 0 | 11 | yes |  |  |  | blast | cuggcacugcucuuaugcuguu | cagcaugagagcacggcuuaga | cuggcacugcucuuaugcuguuuaguguuauaggcagcaugagagcacggcuuaga | manual\_scaffold\_2b:310557568..310557624:- |
| manual\_scaffold\_4b\_14874 | 1.5e+3 | 0.96 ± 0.01 |  | 2986 | 2936 | 0 | 50 | yes |  |  |  | blast | ugaguuggugcugccuggagga | ugccuggccucuccaacccu | ugccuggccucuccaacccucagcgccauuguggcaacugaguuggugcugccuggagga | manual\_scaffold\_4b:78821514..78821574:+ |
| manual\_scaffold\_1b\_3296 | 1.2e+3 | 0.96 ± 0.01 |  | 2370 | 2310 | 0 | 60 | yes |  |  |  | blast | uucguugugaguagcucggguc | uccgggguacucagcaugaag | uccgggguacucagcaugaaggauuaaacuucguugugaguagcucggguc | manual\_scaffold\_1b:535075669..535075720:- |
| manual\_scaffold\_1a\_1309 | 1.1e+3 | 0.96 ± 0.01 |  | 2322 | 2315 | 0 | 7 | yes |  |  |  | blast | uucguugugaguagcucggguc | uccgggguacucagcaaaaa | uccgggguacucagcaaaaaggauuaauauucguugugaguagcucggguc | manual\_scaffold\_1a:547708545..547708596:- |
| manual\_scaffold\_3b\_11833 | 1.1e+3 | 0.96 ± 0.01 |  | 2322 | 2315 | 0 | 7 | yes |  |  |  | blast | uucguugugaguagcucggguc | uccgggguacucagcaaaaa | uccgggguacucagcaaaaaggauuaauauucguugugaguagcucggguc | manual\_scaffold\_3b:789111169..789111220:- |
| manual\_scaffold\_6\_24341 | 1.1e+3 | 0.96 ± 0.01 |  | 2318 | 2315 | 0 | 3 | yes |  |  |  | blast | uucguugugaguagcucggguc | uccgggguacucagcaaaa | uccgggguacucagcaaaaggauuaauauucguugugaguagcucggguc | manual\_scaffold\_6:1570493090..1570493140:- |
| manual\_scaffold\_3b\_10103 | 1.1e+3 | 0.96 ± 0.01 |  | 2229 | 2220 | 0 | 9 | yes |  |  |  | blast | auguagguccaucugcugccu | gacagcagggugaccagcauga | gacagcagggugaccagcaugaggaucaugcuaaaggcuuauguagguccaucugcugccu | manual\_scaffold\_3b:124224766..124224827:+ |
| manual\_scaffold\_9\_31894 | 1.1e+3 | 0.96 ± 0.01 |  | 2174 | 1727 | 0 | 447 | yes |  |  |  | blast | agauguacgaaaggauuuuaccc | gaaaaagcuuucguacauauggc | agauguacgaaaggauuuuacccauucugugucuaugggaaaaagcuuucguacauauggc | manual\_scaffold\_9:944696243..944696304:+ |
| manual\_scaffold\_12\_39310 | 1.0e+3 | 0.96 ± 0.01 |  | 2101 | 2063 | 0 | 38 | yes |  |  |  | blast | ucgcaaucauguuaguccucacc | ugaggacuagcgagagggcaaugu | ugaggacuagcgagagggcaauguacucuucaucgcaaucauguuaguccucacc | manual\_scaffold\_12:388589987..388590042:- |
| manual\_scaffold\_4a\_14793 | 1.0e+3 | 0.96 ± 0.01 |  | 2067 | 2047 | 0 | 20 | yes |  |  |  | blast | ugaguuggugcugccuggaggu | ugccaggccucuccaacuaucagu | ugccaggccucuccaacuaucaguguuauuuaaaugacugaguuggugcugccuggaggu | manual\_scaffold\_4a:885333497..885333557:- |
| manual\_scaffold\_9\_33125 | 1.0e+3 | 0.96 ± 0.01 |  | 2065 | 2048 | 0 | 17 | yes |  |  |  | blast | ugaguuggugcugccuggaggu | cgccuggcugcuccaaucaucagu | cgccuggcugcuccaaucaucagugcuauuacagcauucugaguuggugcugccuggaggu | manual\_scaffold\_9:1066927126..1066927187:- |
| manual\_scaffold\_5\_18618 | 1.0e+3 | 0.96 ± 0.01 |  | 2064 | 2047 | 0 | 17 | yes |  |  |  | blast | ugaguuggugcugccuggaggu | cgccuggcugcuccaaucaucagu | cgccuggcugcuccaaucaucagugcuauuacagcauacugaguuggugcugccuggaggu | manual\_scaffold\_5:1332938990..1332939051:+ |
| manual\_scaffold\_5\_19519 | 1.0e+3 | 0.96 ± 0.01 |  | 2065 | 2048 | 0 | 17 | no |  |  |  | blast | ugaguuggugcugccuggaggu | cgccuggcugcuccaaucaucagu | cgccuggcugcuccaaucaucagugcuauuacagcauucugaguuggugcugccuggaggu | manual\_scaffold\_5:488555246..488555307:- |
| manual\_scaffold\_5\_20375 | 1.0e+3 | 0.96 ± 0.01 |  | 2009 | 1999 | 0 | 10 | no |  |  |  | blast | ugacuugguggugucaggaggu | caccugauuacuccaacgcacc | caccugauuacuccaacgcaccgugguuuuuaucggugacuugguggugucaggaggu | manual\_scaffold\_5:1613707712..1613707770:- |
| manual\_scaffold\_8\_29492 | 9.7e+2 | 0.96 ± 0.01 |  | 1911 | 1788 | 122 | 1 | yes |  | xtr-miR-155 |  | blast | uuaaugcuguccaugacugggguu | ccccaccaggauagcauugccac | uuaaugcuguccaugacugggguugaauccuaggaagaccccaccaggauagcauugccac | manual\_scaffold\_8:1355500936..1355500997:+ |
| manual\_scaffold\_4b\_17274 | 9.7e+2 | 0.96 ± 0.01 |  | 1902 | 1785 | 0 | 117 | yes |  |  |  | blast | aagaggagugcuaggaaggaga | ugcuuucugggauucuuguugu | ugcuuucugggauucuuguuguuugauugcaacaucaagaggagugcuaggaaggaga | manual\_scaffold\_4b:997519703..997519761:- |
| manual\_scaffold\_10\_33440 | 9.6e+2 | 0.96 ± 0.01 |  | 1892 | 1853 | 0 | 39 | no |  | xtr-miR-428a |  | blast | aaagugcuucuaguuugggc | aaugaacuagggcucucuucu | aaugaacuagggcucucuucucguuuugaaacugagaaagugcuucuaguuugggc | manual\_scaffold\_10:48075750..48075806:+ |
| manual\_scaffold\_3a\_9557 | 8.8e+2 | 0.96 ± 0.01 |  | 1728 | 1703 | 0 | 25 | yes |  |  |  | blast | ugggacaccugccaagacuucu | aaguauugugagguguccuggga | ugggacaccugccaagacuucuuagagaagucaggaaguauugugagguguccuggga | manual\_scaffold\_3a:536292642..536292700:- |
| manual\_scaffold\_11\_37754 | 8.6e+2 | 0.96 ± 0.01 |  | 1712 | 1642 | 0 | 70 | no |  |  |  | blast | ucucggagaggacagcaugagc | guaugcuguacuggacgagcu | guaugcuguacuggacgagcuggaggauuucacugauaaucucggagaggacagcaugagc | manual\_scaffold\_11:1015134717..1015134778:- |
| manual\_scaffold\_1b\_3230 | 8.4e+2 | 0.96 ± 0.01 |  | 1650 | 1124 | 0 | 526 | yes |  |  |  | blast | uagcggaugcaggguucagau | uaugagcaugcaucuguugga | uagcggaugcaggguucagauaaucaguuaaaguaugagcaugcaucuguugga | manual\_scaffold\_1b:413785869..413785923:- |
| manual\_scaffold\_6\_22348 | 7.7e+2 | 0.96 ± 0.01 |  | 1518 | 1517 | 0 | 1 | yes |  |  |  | blast | cagggcuacucgcucucauccu | ggaguggaggacuaggccu | cagggcuacucgcucucauccuguaagaucucaggaguggaggacuaggccu | manual\_scaffold\_6:1562057657..1562057709:+ |
| manual\_scaffold\_4b\_15461 | 7.0e+2 | 0.96 ± 0.01 |  | 1373 | 1371 | 0 | 2 | yes |  |  |  | blast | gauguaccaaaggauuuuacc | gaaaacucuuucguacauaug | gauguaccaaaggauuuuacccauucugugucuaugggaaaacucuuucguacauaug | manual\_scaffold\_4b:590318127..590318185:+ |
| manual\_scaffold\_6\_20987 | 6.8e+2 | 0.96 ± 0.01 |  | 1345 | 1343 | 0 | 2 | yes |  |  |  | blast | gauguaccaaaggauuuuacc | gaaaacucuuucguacauaug | gauguaccaaaggauuuuacccuuucugugucuaugggaaaacucuuucguacauaug | manual\_scaffold\_6:234521113..234521171:+ |
| manual\_scaffold\_10\_33353 | 6.7e+2 | 0.96 ± 0.01 |  | 1333 | 1067 | 0 | 266 | yes |  |  |  | blast | uucacuacuagcagaacucgg | ggaauuccgcuaguucugaacu | ggaauuccgcuaguucugaacuauuuacauuuggaaaaaguucacuacuagcagaacucgg | manual\_scaffold\_10:21092015..21092076:+ |
| manual\_scaffold\_4a\_14700 | 6.5e+2 | 0.96 ± 0.01 |  | 1279 | 787 | 0 | 492 | yes |  |  |  | blast | caauguguccccagugcagua | gugcauuguaguugcauugc | gugcauuguaguugcauugcaugucaccuuggucugugcaauguguccccagugcagua | manual\_scaffold\_4a:783248853..783248912:- |
| manual\_scaffold\_4b\_15802 | 6.5e+2 | 0.96 ± 0.01 |  | 1279 | 1278 | 0 | 1 | yes |  |  |  | blast | cccaucaacuguauuacaagu | uuguaauauggcggauggaa | cccaucaacuguauuacaagugccauuagcugaaauacacuuguaauauggcggauggaa | manual\_scaffold\_4b:1004803049..1004803109:+ |
| manual\_scaffold\_12\_38988 | 6.0e+2 | 0.96 ± 0.01 |  | 1181 | 1124 | 0 | 57 | yes |  |  |  | blast | uagcggaugcaggguucagau | ucugagcccucaucuguugga | uagcggaugcaggguucagauaaucaguuaaagucugagcccucaucuguugga | manual\_scaffold\_12:61379129..61379183:- |
| manual\_scaffold\_1a\_1797 | 5.9e+2 | 0.96 ± 0.01 |  | 1169 | 1098 | 0 | 71 | yes |  |  |  | blast | acagcgcagcaagauucucagu | ugaguucaugcuacgcugagu | acagcgcagcaagauucucaguguuuacauggccaugcugaguucaugcuacgcugagu | manual\_scaffold\_1a:1136672163..1136672222:- |
| manual\_scaffold\_7\_26094 | 5.8e+2 | 0.96 ± 0.01 |  | 1148 | 1146 | 0 | 2 | yes |  |  |  | blast | ucuaggcgguacugucaugcugc | gcaugagaguagugccaggauu | ucuaggcgguacugucaugcugcugauauuaaccagcaugagaguagugccaggauu | manual\_scaffold\_7:1363948930..1363948987:+ |
| manual\_scaffold\_3b\_10761 | 5.8e+2 | 0.96 ± 0.01 |  | 1144 | 1124 | 0 | 20 | yes |  |  |  | blast | uagcggaugcaggguucagau | ucugagcacacaucugucgga | uagcggaugcaggguucagauaaucaguuaaagucugagcacacaucugucgga | manual\_scaffold\_3b:857805393..857805447:+ |
| manual\_scaffold\_12\_37863 | 5.7e+2 | 0.96 ± 0.01 |  | 1133 | 1113 | 0 | 20 | yes |  |  |  | blast | uagcggaugcaggguucagau | ucugagcacacaucugucgga | uagcggaugcaggguucagauaauuaguuaaagucugagcacacaucugucgga | manual\_scaffold\_12:43293477..43293531:+ |
| manual\_scaffold\_1b\_3654 | 5.7e+2 | 0.96 ± 0.01 |  | 1132 | 1124 | 0 | 8 | yes |  |  |  | blast | uagcggaugcaggguucagau | ucugagcacacaucugcugga | uagcggaugcaggguucagauaaucaguuaaagucugagcacacaucugcugga | manual\_scaffold\_1b:1032625428..1032625482:- |
| manual\_scaffold\_9\_30985 | 5.7e+2 | 0.96 ± 0.01 |  | 1132 | 1124 | 0 | 8 | yes |  |  |  | blast | uagcggaugcaggguucagau | ucugagcacacaucugcugga | uagcggaugcaggguucagauaaucaguuaaagucugagcacacaucugcugga | manual\_scaffold\_9:138304427..138304481:+ |
| manual\_scaffold\_2b\_6089 | 5.7e+2 | 0.96 ± 0.01 |  | 1130 | 1124 | 0 | 6 | yes |  |  |  | blast | uagcggaugcaggguucagau | ucugaccacgcaucuguugga | uagcggaugcaggguucagauaaucaguuacgaucugaccacgcaucuguugga | manual\_scaffold\_2b:231677495..231677549:+ |
| manual\_scaffold\_5\_18215 | 5.7e+2 | 0.96 ± 0.01 |  | 1138 | 1120 | 0 | 18 | no |  |  |  | blast | uagcggaugcaggguucagau | ucggagcacgcaucuguugga | uagcggaugcaggguucagauaaucagaaaagucggagcacgcaucuguugga | manual\_scaffold\_5:752771514..752771567:+ |
| manual\_scaffold\_11\_35911 | 5.7e+2 | 0.96 ± 0.01 |  | 1118 | 1100 | 0 | 18 | yes |  |  |  | blast | uguagaccacaagaugaacgacu | ucauucaucugguggucuguau | ucauucaucugguggucuguaugucacuaguacaucauguagaccacaagaugaacgacu | manual\_scaffold\_11:56777600..56777660:+ |
| manual\_scaffold\_4b\_17043 | 5.2e+2 | 0.96 ± 0.01 |  | 1034 | 945 | 0 | 89 | yes |  |  |  | blast | ucgcggucacguuaguccucacu | ugaggacuugcgagagugcaa | ugaggacuugcgagagugcaauguacucuucaucgcggucacguuaguccucacu | manual\_scaffold\_4b:710990505..710990560:- |
| manual\_scaffold\_1b\_2200 | 5.1e+2 | 0.96 ± 0.01 |  | 1019 | 883 | 0 | 136 | yes |  |  |  | blast | gagucuuggagcuguucaguggu | uauugaacucucucauucucu | uauugaacucucucauucucugugauuuaucuaucagagucuuggagcuguucaguggu | manual\_scaffold\_1b:447986363..447986422:+ |
| manual\_scaffold\_9\_30925 | 5.0e+2 | 0.96 ± 0.01 |  | 990 | 711 | 0 | 279 | yes |  |  |  | blast | uccuuucuggaguuugacugcu | aaauccaacuccaguuaggau | aaauccaacuccaguuaggauguaauuaaaaacaauccuuucuggaguuugacugcu | manual\_scaffold\_9:71007848..71007905:+ |
| manual\_scaffold\_3a\_7660 | 4.8e+2 | 0.96 ± 0.01 |  | 951 | 883 | 0 | 68 | yes |  |  |  | blast | gagucuuggagcuguucaguggu | ucuugaacucuucuagccu | ucuugaacucuucuagccucugugauuuaucuaucagagucuuggagcuguucaguggu | manual\_scaffold\_3a:341464227..341464286:+ |
| manual\_scaffold\_3b\_11193 | 4.8e+2 | 0.96 ± 0.01 |  | 949 | 883 | 0 | 66 | yes |  |  |  | blast | gagucuuggagcuguucaguggu | uguugaacucuccgagccu | uguugaacucuccgagccucugugauuuaucuaucagagucuuggagcuguucaguggu | manual\_scaffold\_3b:165207895..165207954:- |
| manual\_scaffold\_3b\_12022 | 4.8e+2 | 0.96 ± 0.01 |  | 950 | 946 | 0 | 4 | yes |  |  |  | blast | uauguucguaacauguagugcc | caaacaugcuacgagcaguaaau | caaacaugcuacgagcaguaaauguaaccuuguaauuauguucguaacauguagugcc | manual\_scaffold\_3b:999858530..999858588:- |
| manual\_scaffold\_4b\_16865 | 4.8e+2 | 0.96 ± 0.01 |  | 952 | 883 | 0 | 69 | no |  |  |  | blast | gagucuuggagcuguucaguggu | ucuugaacucucccagccucu | ucuugaacucucccagccucugugauucaucuaucagagucuuggagcuguucaguggu | manual\_scaffold\_4b:473826028..473826087:- |
| manual\_scaffold\_7\_25576 | 4.8e+2 | 0.96 ± 0.01 |  | 951 | 883 | 0 | 68 | no |  |  |  | blast | gagucuuggagcuguucaguggu | ucuugaacucuucuagccu | ucuugaacucuucuagccucugugauuuaucuaucagagucuuggagcuguucaguggu | manual\_scaffold\_7:952681164..952681223:+ |
| manual\_scaffold\_10\_35354 | 4.7e+2 | 0.96 ± 0.01 |  | 938 | 919 | 0 | 19 | yes |  |  |  | blast | uagcggaugcaggguucagau | ucugagcaugcaucugcugga | uagcggaugcaggguucagauauucaguuaaagucugagcaugcaucugcugga | manual\_scaffold\_10:681618712..681618766:- |
| manual\_scaffold\_4a\_13093 | 4.6e+2 | 0.96 ± 0.01 |  | 921 | 855 | 0 | 66 | yes |  |  |  | blast | cgccuggcuucaucauaacucugu | ugaguugaugacgcugggaggu | cgccuggcuucaucauaacucugugcuauuaaagcuuucugaguugaugacgcugggaggu | manual\_scaffold\_4a:498941820..498941881:+ |
| manual\_scaffold\_5\_17707 | 4.6e+2 | 0.96 ± 0.01 |  | 921 | 855 | 0 | 66 | yes |  |  |  | blast | cgccuggcuucaucauaacucugu | ugaguugaugacgcugggaggu | cgccuggcuucaucauaacucugugcuaauaaagcuuugaguugaugacgcugggaggu | manual\_scaffold\_5:186048543..186048602:+ |
| manual\_scaffold\_7\_27183 | 4.6e+2 | 0.96 ± 0.01 |  | 918 | 744 | 0 | 174 | yes |  |  |  | blast | cauuugaauaaaaugggagcc | guucccauuguguucaaugau | cauuugaauaaaaugggagccaugguauaagaaugagguucccauuguguucaaugau | manual\_scaffold\_7:650039337..650039395:- |
| manual\_scaffold\_10\_34819 | 4.5e+2 | 0.96 ± 0.01 |  | 906 | 797 | 0 | 109 | no |  |  |  | blast | ugaguuggugacgccgggaggu | cgccuggcuccaucauaacucugu | cgccuggcuccaucauaacucugugcuauaagcuuucugaguuggugacgccgggaggu | manual\_scaffold\_10:182621490..182621549:- |
| manual\_scaffold\_8\_28970 | 4.5e+2 | 0.96 ± 0.01 |  | 899 | 883 | 0 | 16 | no |  |  |  | blast | gagucuuggagcuguucaguggu | ucuugaacugugucagucucu | ucuugaacugugucagucucugugauuuaucuaucagagucuuggagcuguucaguggu | manual\_scaffold\_8:559359592..559359651:+ |
| manual\_scaffold\_5\_18646 | 4.5e+2 | 0.96 ± 0.01 |  | 892 | 720 | 0 | 172 | yes |  |  |  | blast | auacugcaucaggaacugauu | aucaguuccuaaugcauuaccu | auacugcaucaggaacugauuggaucacuuuuaauugccaucaguuccuaaugcauuaccu | manual\_scaffold\_5:1366828336..1366828397:+ |
| manual\_scaffold\_3b\_11663 | 4.5e+2 | 0.96 ± 0.01 |  | 894 | 883 | 0 | 11 | no |  |  |  | blast | gagucuuggagcuguucaguggu | uuuugaacucucucagccucu | uuuugaacucucucagccucugugauucaucuaucagagucuuggagcuguucaguggu | manual\_scaffold\_3b:579008348..579008407:- |
| manual\_scaffold\_8\_30365 | 4.3e+2 | 0.96 ± 0.01 |  | 850 | 844 | 0 | 6 | yes |  |  |  | blast | ugaggcauccugcauccaaccu | guagugcagugugccucaagggu | ugaggcauccugcauccaaccuguuguuuuaucggguagugcagugugccucaagggu | manual\_scaffold\_8:913969147..913969205:- |
| manual\_scaffold\_10\_34802 | 4.1e+2 | 0.96 ± 0.01 |  | 809 | 470 | 1 | 338 | yes |  | xtr-miR-33a |  | blast | gugcauuguaguugcauugc | caaugcgucugcagugcaaua | gugcauuguaguugcauugcaugguucauacgagguguccaaugcgucugcagugcaaua | manual\_scaffold\_10:155174021..155174081:- |
| manual\_scaffold\_5\_18932 | 4.1e+2 | 0.96 ± 0.01 |  | 823 | 797 | 0 | 26 | no |  |  |  | blast | ugaguuggugacgccgggaggu | uuccuggcuuaaucauaacucc | uuccuggcuuaaucauaacuccgugcucugauagcuuucugaguuggugacgccgggaggu | manual\_scaffold\_5:1725747864..1725747925:+ |
| manual\_scaffold\_1a\_324 | 4.1e+2 | 0.96 ± 0.01 |  | 814 | 785 | 0 | 29 | yes |  |  |  | blast | cacuuggccacucuuagacucugc | agaguuggagcugccaggaggu | cacuuggccacucuuagacucugcgcuguuaaggcuuguagaguuggagcugccaggaggu | manual\_scaffold\_1a:385578017..385578078:+ |
| manual\_scaffold\_2b\_6538 | 4.1e+2 | 0.96 ± 0.01 |  | 819 | 797 | 0 | 22 | no |  |  |  | blast | ugaguuggugacgccgggaggu | caccuggauucaucauaacucugu | caccuggauucaucauaacucugugcuauuaaagcuuucugaguuggugacgccgggaggu | manual\_scaffold\_2b:796469775..796469836:+ |
| manual\_scaffold\_4a\_12348 | 4.1e+2 | 0.96 ± 0.01 |  | 808 | 797 | 0 | 11 | yes |  |  |  | blast | ugaguuggugacgccgggaggu | cgccuggcuucaucauuacucugu | cgccuggcuucaucauuacucugugcuauaauagcuuucugaguuggugacgccgggaggu | manual\_scaffold\_4a:52705663..52705724:+ |
| manual\_scaffold\_3a\_9076 | 4.0e+2 | 0.96 ± 0.01 |  | 794 | 725 | 0 | 69 | yes |  |  |  | blast | gaugaauccgugcugagcugg | agcgcugcauggauuuuucaac | gaugaauccgugcugagcuggcgaugcguggauuuccccagcgcugcauggauuuuucaac | manual\_scaffold\_3a:332160511..332160572:- |
| manual\_scaffold\_7\_26771 | 4.0e+2 | 0.96 ± 0.01 |  | 788 | 754 | 0 | 34 | yes |  |  |  | blast | aaugccaaucgguuuugcuugu | aagugagaccaauuggcauu | aaugccaaucgguuuugcuuguauauaaaggcaagugagaccaauuggcauu | manual\_scaffold\_7:194891170..194891222:- |
| manual\_scaffold\_4a\_12967 | 4.0e+2 | 0.96 ± 0.01 |  | 785 | 768 | 0 | 17 | yes |  |  |  | blast | cgcuuggacucucucaggcucu | agucugggagcugucuagugga | cgcuuggacucucucaggcucugugauuuaucuaucggagucugggagcugucuagugga | manual\_scaffold\_4a:281275650..281275710:+ |
| manual\_scaffold\_1b\_3516 | 4.0e+2 | 0.96 ± 0.01 |  | 802 | 796 | 0 | 6 | no |  |  |  | blast | ugccaaauucaguacugugaau | cauaguauucucuuuggacaca | cauaguauucucuuuggacacaaaguauuaagaugugccaaauucaguacugugaau | manual\_scaffold\_1b:841585813..841585870:- |
| manual\_scaffold\_7\_25385 | 3.8e+2 | 0.96 ± 0.01 |  | 755 | 736 | 0 | 19 | yes |  |  |  | blast | uagcggaugcaggguucagau | ucugagcaugcaucugcugga | uagcggaugcaggguucagaucaucagaaaagucugagcaugcaucugcugga | manual\_scaffold\_7:737462553..737462606:+ |
| manual\_scaffold\_9\_32623 | 3.7e+2 | 0.96 ± 0.01 |  | 736 | 264 | 0 | 472 | yes |  |  |  | blast | cacugucauguuaguccucacc | ugaggacuugcgagacggca | ugaggacuugcgagacggcaauuuacucuucaucacugucauguuaguccucacc | manual\_scaffold\_9:494730572..494730627:- |
| manual\_scaffold\_7\_26297 | 3.7e+2 | 0.96 ± 0.01 |  | 735 | 708 | 0 | 27 | yes |  |  |  | blast | ucgcgaucacguuaguccucacu | ugaggacuugcgagagggcaaugu | ugaggacuugcgagagggcaauguacucuucaucgcgaucacguuaguccucacu | manual\_scaffold\_7:1468268513..1468268568:+ |
| manual\_scaffold\_5\_18812 | 3.7e+2 | 0.96 ± 0.01 |  | 727 | 721 | 0 | 6 | yes |  |  |  | blast | cagagagguucagugauugugu | acaguaccuggacgcucuuucc | cagagagguucagugauuguguaguuggcauucacaauccacacaguaccuggacgcucuuucc | manual\_scaffold\_5:1550459621..1550459685:+ |
| manual\_scaffold\_7\_25687 | 3.6e+2 | 0.96 ± 0.01 |  | 719 | 526 | 0 | 193 | yes |  |  |  | blast | uaugagcaugcaucuguugga | uagcagaugcaggguucagau | uagcagaugcaggguucagauaaucaguaaaguaugagcaugcaucuguugga | manual\_scaffold\_7:1043113449..1043113502:+ |
| manual\_scaffold\_4b\_15488 | 3.4e+2 | 0.96 ± 0.01 |  | 680 | 670 | 0 | 10 | yes |  |  |  | blast | uccaaucacugcuccuauguugc | agcauaaagcagugcuaggauu | uccaaucacugcuccuauguugcauauaauacuaagcagcauaaagcagugcuaggauu | manual\_scaffold\_4b:621198529..621198588:+ |
| manual\_scaffold\_7\_26308 | 3.4e+2 | 0.96 ± 0.01 |  | 684 | 652 | 0 | 32 | no |  |  |  | blast | ugaguuggugcugccuggagg | caccuggccgcucuaaucauc | caccuggccgcucuaaucaucagugcuauuacggcauacugaguuggugcugccuggagg | manual\_scaffold\_7:1474991108..1474991168:+ |
| manual\_scaffold\_1b\_3475 | 3.4e+2 | 0.96 ± 0.01 |  | 666 | 664 | 0 | 2 | yes |  |  |  | blast | uuggcagugcucuuaugcuguu | caucaugagagcagugcuuuua | uuggcagugcucuuaugcuguuuacuuauauagacaucaugagagcagugcuuuua | manual\_scaffold\_1b:786187673..786187729:- |
| manual\_scaffold\_5\_18642 | 3.3e+2 | 0.96 ± 0.01 |  | 659 | 651 | 3 | 5 | yes |  |  |  | blast | aaaucucugcaggcaacuguga | ucacaauugccuggagaguuuc | aaaucucugcaggcaacugugaugucauugcagcaaucucacaauugccuggagaguuuc | manual\_scaffold\_5:1366769850..1366769910:+ |
| manual\_scaffold\_7\_27796 | 3.3e+2 | 0.96 ± 0.01 |  | 654 | 590 | 0 | 64 | yes |  |  |  | blast | cuuuuccgguggaugcucuagc | uaggaguaucugucagaaaaac | uaggaguaucugucagaaaaacaaguuaauuucaauauugcuuuuccgguggaugcucuagc | manual\_scaffold\_7:1254282833..1254282895:- |
| manual\_scaffold\_12\_38315 | 3.0e+2 | 0.96 ± 0.01 |  | 605 | 320 | 0 | 285 | yes |  |  |  | blast | ucugagcacguaucuguugga | uaacggaugcaggguucagau | uaacggaugcaggguucagauaauacuuaaugucugagcacguaucuguugga | manual\_scaffold\_12:489072811..489072864:+ |
| manual\_scaffold\_11\_36016 | 2.9e+2 | 0.96 ± 0.01 |  | 581 | 266 | 0 | 315 | yes |  |  |  | blast | cuuuucuuagggaccuggau | uccacgucccuuuaggaagagcu | cuuuucuuagggaccuggauaaauaauugguuaaaaagcauauccacgucccuuuaggaagagcu | manual\_scaffold\_11:179039314..179039379:+ |
| manual\_scaffold\_4a\_13100 | 2.9e+2 | 0.96 ± 0.01 |  | 579 | 550 | 0 | 29 | yes |  |  |  | blast | ugagagcagcaccuggauuagu | aaauccaggcacuucuuucaug | aaauccaggcacuucuuucaugcucguuaacggcaugagagcagcaccuggauuagu | manual\_scaffold\_4a:501950400..501950457:+ |
| manual\_scaffold\_5\_17648 | 2.7e+2 | 0.96 ± 0.01 |  | 532 | 527 | 0 | 5 | yes |  |  |  | blast | aagcccaaccuggcaugaagcug | cuucuggccaagcuggcuuag | aagcccaaccuggcaugaagcugacacaguuaaauuagucacuucuggccaagcuggcuuag | manual\_scaffold\_5:127540352..127540414:+ |
| manual\_scaffold\_9\_31564 | 2.7e+2 | 0.96 ± 0.01 |  | 533 | 417 | 0 | 116 | yes |  |  |  | blast | aaggaaucuucacagcugucc | auuggcgauaaaagauuccgac | aaggaaucuucacagcuguccagugaggccauacuggauuggcgauaaaagauuccgac | manual\_scaffold\_9:768598029..768598088:+ |
| manual\_scaffold\_6\_23836 | 2.6e+2 | 0.96 ± 0.01 |  | 527 | 524 | 0 | 3 | yes |  |  |  | blast | uuauuuagauuucgcggacgaga | caucugcugaaaucuaacuacc | caucugcugaaaucuaacuaccaauagaaaacaauguuauuuagauuucgcggacgaga | manual\_scaffold\_6:894827132..894827191:- |
| manual\_scaffold\_9\_31562 | 2.6e+2 | 0.96 ± 0.01 |  | 533 | 417 | 0 | 116 | no |  |  |  | blast | aaggaaucuucacagcugucc | auuggcgauaaaagauuccgac | aaggaaucuucacagcuguccagugaggccauacuggauuggcgauaaaagauuccgac | manual\_scaffold\_9:768594093..768594152:+ |
| manual\_scaffold\_4b\_15841 | 2.5e+2 | 0.96 ± 0.01 |  | 491 | 471 | 0 | 20 | yes |  |  |  | blast | ccaagcacugcuuuuaugcug | agcauaagaggcgugcuaggau | ccaagcacugcuuuuaugcugcauauaauaguaaacagcauaagaggcgugcuaggau | manual\_scaffold\_4b:1040070693..1040070751:+ |
| manual\_scaffold\_4b\_16918 | 2.5e+2 | 0.96 ± 0.01 |  | 492 | 491 | 0 | 1 | yes |  |  |  | blast | uaaauugagacugccaggaggu | cgccuggccaucucauuauu | cgccuggccaucucauuauucagugccaaaaagguuuacuaaauugagacugccaggaggu | manual\_scaffold\_4b:559936922..559936983:- |
| manual\_scaffold\_4b\_16920 | 2.5e+2 | 0.96 ± 0.01 |  | 492 | 491 | 0 | 1 | yes |  |  |  | blast | uaaauugagacugccaggaggu | cgccuggccaucucauuauu | cgccuggccaucucauuauucagugccaaaaagguuuacuaaauugagacugccaggaggu | manual\_scaffold\_4b:559947529..559947590:- |
| manual\_scaffold\_1a\_150 | 2.4e+2 | 0.96 ± 0.01 |  | 483 | 330 | 0 | 153 | yes |  |  |  | blast | aguuuggagcugccaagaggu | cacuuggccacucuucaacucu | cacuuggccacucuucaacucugugcuauauaacuaguggaguuuggagcugccaagaggu | manual\_scaffold\_1a:149392926..149392987:+ |
| manual\_scaffold\_4b\_16916 | 2.4e+2 | 0.96 ± 0.01 |  | 492 | 491 | 0 | 1 | no |  |  |  | blast | uaaauugagacugccaggaggu | cgccuggccaucucauuauu | cgccuggccaucucauuauucagugccaaaaagguuuacuaaauugagacugccaggaggu | manual\_scaffold\_4b:559926315..559926376:- |
| manual\_scaffold\_8\_28635 | 2.3e+2 | 0.96 ± 0.01 |  | 478 | 474 | 0 | 4 | no |  |  |  | blast | aagcuaauaauaggggucaga | gggccucucuuguuaugcuuugu | gggccucucuuguuaugcuuuguucuuguaggaagcuaauaauaggggucaga | manual\_scaffold\_8:161741644..161741697:+ |
| manual\_scaffold\_9\_31205 | 2.3e+2 | 0.96 ± 0.01 |  | 457 | 386 | 0 | 71 | yes |  |  |  | blast | uugccgcccugucaguucccauu | uggaaacuccuaggguugcagu | uggaaacuccuaggguugcaguguguccuucauugccgcccugucaguucccauu | manual\_scaffold\_9:325872292..325872347:+ |
| manual\_scaffold\_11\_36050 | 2.3e+2 | 0.96 ± 0.01 |  | 456 | 455 | 0 | 1 | yes |  |  |  | blast | uugaggcauucuccuacccuuccu | gaggguggggauugccauagu | uugaggcauucuccuacccuuccucugucuucuuugaggaggguggggauugccauagu | manual\_scaffold\_11:218486159..218486218:+ |
| manual\_scaffold\_1b\_2964 | 2.3e+2 | 0.96 ± 0.01 |  | 454 | 261 | 0 | 193 | yes |  |  |  | blast | gucuaggcaugcaucuguugga | uagcagaugcaggguucagau | uagcagaugcaggguucagauaaucagaaaagucuaggcaugcaucuguugga | manual\_scaffold\_1b:117001332..117001385:- |
| manual\_scaffold\_4b\_17346 | 2.3e+2 | 0.96 ± 0.01 |  | 463 | 462 | 0 | 1 | no |  |  |  | blast | cuuggcuccaaugauaucuga | agguugauuugcgaggcc | agguugauuugcgaggccaauuaugugccacugcuugugaauuacuuggcuccaaugauaucuga | manual\_scaffold\_4b:1070176008..1070176073:- |
| manual\_scaffold\_1b\_2372 | 2.3e+2 | 0.96 ± 0.01 |  | 454 | 203 | 0 | 251 | yes |  |  |  | blast | cgcuuagacucucucagucucu | agucuuggagcugucuaguggu | cgcuuagacucucucagucucugugauuuaucuaucagagucuuggagcugucuaguggu | manual\_scaffold\_1b:697534309..697534369:+ |
| manual\_scaffold\_9\_31522 | 2.3e+2 | 0.96 ± 0.01 |  | 450 | 330 | 0 | 120 | yes |  |  |  | blast | aguuuggagcugccaagaggu | uacuuggccaaucuuuaacucu | uacuuggccaaucuuuaacucugugcuauaauaacuaguggaguuuggagcugccaagaggu | manual\_scaffold\_9:733455215..733455277:+ |
| manual\_scaffold\_3a\_7849 | 2.2e+2 | 0.96 ± 0.01 |  | 438 | 433 | 0 | 5 | yes |  |  |  | blast | uaguagagucuugcuggugugg | caugccagcaagacuccuccu | caugccagcaagacuccuccugcuguauuaugaaccguaguagagucuugcuggugugg | manual\_scaffold\_3a:408704184..408704243:+ |
| manual\_scaffold\_12\_38468 | 2.2e+2 | 0.96 ± 0.01 |  | 431 | 348 | 0 | 83 | yes |  |  |  | blast | cgccaggccacuccaaaaaucu | gauuuggaggugcccggaggu | cgccaggccacuccaaaaaucugcgcuauaacagcauucagauuuggaggugcccggaggu | manual\_scaffold\_12:596798874..596798935:+ |
| manual\_scaffold\_5\_19567 | 2.1e+2 | 0.96 ± 0.01 |  | 425 | 421 | 0 | 4 | yes |  |  |  | blast | ugggcaucuuugaauuauuucu | aaaucauucagagauggcuacc | ugggcaucuuugaauuauuucucuuaagacaucugaaaaucauucagagauggcuacc | manual\_scaffold\_5:549039147..549039205:- |
| manual\_scaffold\_1b\_2915 | 2.1e+2 | 0.96 ± 0.01 |  | 424 | 415 | 0 | 9 | yes |  |  |  | blast | uuccuggccucuccaaucuuca | gaauugguguugccuggaggu | uuccuggccucuccaaucuucagugcuauuacggcaucugaauugguguugccuggaggu | manual\_scaffold\_1b:46702904..46702964:- |
| manual\_scaffold\_7\_25182 | 2.1e+2 | 0.96 ± 0.01 |  | 431 | 348 | 0 | 83 | no |  |  |  | blast | cgccaggccacuccaaaaaucu | gauuuggaggugcccggaggu | cgccaggccacuccaaaaaucugcgcuaugauagcauucagauuuggaggugcccggaggu | manual\_scaffold\_7:576451420..576451481:+ |
| manual\_scaffold\_3b\_12194 | 2.1e+2 | 0.96 ± 0.01 |  | 414 | 373 | 0 | 41 | yes |  |  |  | blast | uaguuagagcagcaccuggaau | auccagaugcuucuuucaugcu | auccagaugcuucuuucaugcuaguuaacagcuaguuagagcagcaccuggaau | manual\_scaffold\_3b:1162675579..1162675633:- |
| manual\_scaffold\_8\_30146 | 2.1e+2 | 0.96 ± 0.01 |  | 412 | 407 | 0 | 5 | yes |  |  |  | blast | aagugcuuucuguugagcuuu | agcuuaaacuuaggcacuug | agcuuaaacuuaggcacuugcuuuguuuagaucuguaagugcuuucuguugagcuuu | manual\_scaffold\_8:570703227..570703284:- |
| manual\_scaffold\_7\_27285 | 1.9e+2 | 0.96 ± 0.01 |  | 390 | 331 | 0 | 59 | yes |  |  |  | blast | aguuuggagcugccaagaggu | cacuaggccauucuuuaacucu | cacuaggccauucuuuaacucugugcuauaacagcuaguagaguuuggagcugccaagaggu | manual\_scaffold\_7:785243988..785244050:- |
| manual\_scaffold\_6\_21974 | 1.9e+2 | 0.96 ± 0.01 |  | 390 | 331 | 0 | 59 | yes |  |  |  | blast | aguuuggagcugccaagaggu | cacuaggccauucuuuaacucu | cacuaggccauucuuuaacucugugcuauaacggcuaguagaguuuggagcugccaagaggu | manual\_scaffold\_6:1188552686..1188552748:+ |
| manual\_scaffold\_12\_37978 | 1.9e+2 | 0.96 ± 0.01 |  | 385 | 226 | 3 | 156 | yes |  |  |  | blast | ugcuggauucuguccccuucc | guggguacugaugccugcacc | guggguacugaugccugcaccaccuggacaucuccuugggugcuggauucuguccccuucc | manual\_scaffold\_12:81952253..81952314:+ |
| manual\_scaffold\_5\_17701 | 1.9e+2 | 0.96 ± 0.01 |  | 383 | 369 | 0 | 14 | yes |  |  |  | blast | cuaccaaaugacagcgagagcu | cucucgcuaucauuugguaga | cucucgcuaucauuugguagaaguuugaaguucuaccaaaugacagcgagagcu | manual\_scaffold\_5:174011970..174012024:+ |
| manual\_scaffold\_8\_28770 | 1.9e+2 | 0.96 ± 0.01 |  | 385 | 368 | 0 | 17 | yes |  |  |  | blast | aguuuggagcugccaagaggu | cgcuuggccauucuuaaacucu | cgcuuggccauucuuaaacucugugcuauaacggcuaguagaguuuggagcugccaagaggu | manual\_scaffold\_8:279194873..279194935:+ |
| manual\_scaffold\_2b\_6567 | 1.9e+2 | 0.96 ± 0.01 |  | 390 | 331 | 0 | 59 | no |  |  |  | blast | aguuuggagcugccaagaggu | cacuaggccauucuuuaacucu | cacuaggccauucuuuaacucugugcuaugauagcuaguagaguuuggagcugccaagaggu | manual\_scaffold\_2b:70003159..70003221:- |
| manual\_scaffold\_4b\_17025 | 1.9e+2 | 0.96 ± 0.01 |  | 389 | 258 | 0 | 131 | no |  |  |  | blast | aacuugaacucucucaggcucu | aguuuuggagcugucuaguggu | aacuugaacucucucaggcucugugguauaucuauuggaguuuuggagcugucuaguggu | manual\_scaffold\_4b:691273088..691273148:- |
| manual\_scaffold\_6\_21458 | 1.9e+2 | 0.96 ± 0.01 |  | 380 | 309 | 0 | 71 | yes |  |  |  | blast | aguuuggagcugccaagaggu | cacuuggccauucuguaacucu | cacuuggccauucuguaacucugugcuauaacggcuaguaaaguuuggagcugccaagaggu | manual\_scaffold\_6:637518176..637518238:+ |
| manual\_scaffold\_5\_17930 | 1.9e+2 | 0.96 ± 0.01 |  | 377 | 330 | 0 | 47 | yes |  |  |  | blast | aguuuggagcugccaagaggu | cacuugggcauucuuuaacucu | cacuugggcauucuuuaacucugugcuauauaacuaguggaguuuggagcugccaagaggu | manual\_scaffold\_5:368510883..368510944:+ |
| manual\_scaffold\_2a\_5791 | 1.8e+2 | 0.96 ± 0.01 |  | 371 | 331 | 0 | 40 | yes |  |  |  | blast | aguuuggagcugccaagaggu | cacucggccauucuuuaacucu | cacucggccauucuuuaacucugugcuaugauagcuaguagaguuuggagcugccaagaggu | manual\_scaffold\_2a:1309923953..1309924015:- |
| manual\_scaffold\_2b\_6478 | 1.8e+2 | 0.96 ± 0.01 |  | 370 | 330 | 0 | 40 | yes |  |  |  | blast | aguuuggagcugccaagaggu | cacucggccauucuuuaacucu | cacucggccauucuuuaacucuguguuauaauaacuagcggaguuuggagcugccaagaggu | manual\_scaffold\_2b:721316559..721316621:+ |
| manual\_scaffold\_10\_33496 | 1.8e+2 | 0.96 ± 0.01 |  | 377 | 330 | 0 | 47 | no |  |  |  | blast | aguuuggagcugccaagaggu | cacuugggcauucuuuaacucu | cacuugggcauucuuuaacucugugcuauaauaacuaguggaguuuggagcugccaagaggu | manual\_scaffold\_10:62250756..62250818:+ |
| manual\_scaffold\_2a\_4704 | 1.8e+2 | 0.96 ± 0.01 |  | 365 | 331 | 0 | 34 | yes |  |  |  | blast | aguuuggagcugccaagaggu | cacuuggcccuucuuuaacucu | cacuuggcccuucuuuaacucugagcuauaacggcuaguagaguuuggagcugccaagaggu | manual\_scaffold\_2a:1195071462..1195071524:+ |
| manual\_scaffold\_4a\_13142 | 1.8e+2 | 0.96 ± 0.01 |  | 364 | 330 | 0 | 34 | yes |  |  |  | blast | aguuuggagcugccaagaggu | cacuuggcccuucuuuaacucu | cacuuggcccuucuuuaacucugugcuauauaacuaguggaguuuggagcugccaagaggu | manual\_scaffold\_4a:558842756..558842817:+ |
| manual\_scaffold\_8\_29068 | 1.8e+2 | 0.96 ± 0.01 |  | 370 | 330 | 0 | 40 | no |  |  |  | blast | aguuuggagcugccaagaggu | cacucggccauucuuuaacucu | cacucggccauucuuuaacucugugcuauaauaacuaguggaguuuggagcugccaagaggu | manual\_scaffold\_8:707006757..707006819:+ |
| manual\_scaffold\_4b\_16006 | 1.8e+2 | 0.96 ± 0.01 |  | 358 | 338 | 0 | 20 | yes |  |  |  | blast | ugggacacugcuccccucaccu | uucguggggugguguucuucc | ugggacacugcuccccucaccuguaacaugcaguccagguucguggggugguguucuucc | manual\_scaffold\_4b:1160594486..1160594546:+ |
| manual\_scaffold\_4a\_13140 | 1.8e+2 | 0.96 ± 0.01 |  | 364 | 330 | 0 | 34 | no |  |  |  | blast | aguuuggagcugccaagaggu | cacuuggcccuucuuuaacucu | cacuuggcccuucuuuaacucugugcuauauaacuaguggaguuuggagcugccaagaggu | manual\_scaffold\_4a:558836661..558836722:+ |
| manual\_scaffold\_8\_29737 | 1.8e+2 | 0.96 ± 0.01 |  | 361 | 348 | 0 | 13 | no |  |  |  | blast | cgccaggccacuccaaaaaucu | gauuuggaggugcccggagg | cgccaggccacuccaaaaaucugcgcuguaauagcauucagauuuggaggugcccggagg | manual\_scaffold\_8:62830292..62830352:- |
| manual\_scaffold\_1a\_364 | 1.7e+2 | 0.96 ± 0.01 |  | 353 | 331 | 0 | 22 | yes |  |  |  | blast | aguuuggagcugccaagaggu | cacguggccauucuuuaacucu | cacguggccauucuuuaacucuaagcuaugauagcuaguagaguuuggagcugccaagaggu | manual\_scaffold\_1a:414781434..414781496:+ |
| manual\_scaffold\_3a\_9077 | 1.7e+2 | 0.96 ± 0.01 |  | 349 | 345 | 0 | 4 | yes |  |  |  | blast | agcccgccgccgaccaugcuga | gcaguaguggcggcaggcauc | agcccgccgccgaccaugcugacagucgacggaagaccagcaguaguggcggcaggcauc | manual\_scaffold\_3a:332181372..332181432:- |
| manual\_scaffold\_3a\_7370 | 1.7e+2 | 0.96 ± 0.01 |  | 348 | 331 | 0 | 17 | yes |  |  |  | blast | aguuuggagcugccaagaggu | cgcuuggccauucuuaaacucu | cgcuuggccauucuuaaacucugugcuauaucggcuaguagaguuuggagcugccaagaggu | manual\_scaffold\_3a:146382269..146382331:+ |
| manual\_scaffold\_8\_29767 | 1.7e+2 | 0.96 ± 0.01 |  | 348 | 331 | 0 | 17 | yes |  |  |  | blast | aguuuggagcugccaagaggu | cacuuggccauuuuauaacucu | cacuuggccauuuuauaacucugugcuaugauagcuaguagaguuuggagcugccaagaggu | manual\_scaffold\_8:72354036..72354098:- |
| manual\_scaffold\_11\_37095 | 1.7e+2 | 0.96 ± 0.01 |  | 347 | 330 | 0 | 17 | yes |  |  |  | blast | aguuuggagcugccaagaggu | cacuuggccauuuuauaacucu | cacuuggccauuuuauaacucuguguuauaauaacuaguggaguuuggagcugccaagaggu | manual\_scaffold\_11:268081494..268081556:- |
| manual\_scaffold\_1b\_2612 | 1.7e+2 | 0.96 ± 0.01 |  | 345 | 330 | 0 | 15 | yes |  |  |  | blast | aguuuggagcugccaagaggu | cacuuggccauucuuuuacucu | cacuuggccauucuuuuacucugugcuauauaacuaguggaguuuggagcugccaagaggu | manual\_scaffold\_1b:994358709..994358770:+ |
| manual\_scaffold\_3b\_10350 | 1.7e+2 | 0.96 ± 0.01 |  | 345 | 330 | 0 | 15 | yes |  |  |  | blast | aguuuggagcugccaagaggu | cacuuggccauucuuuuacucu | cacuuggccauucuuuuacucugugauauaauaucuaguggaguuuggagcugccaagaggu | manual\_scaffold\_3b:396667615..396667677:+ |
| manual\_scaffold\_4b\_16823 | 1.7e+2 | 0.96 ± 0.01 |  | 352 | 330 | 0 | 22 | no |  |  |  | blast | aguuuggagcugccaagaggu | cacuuggccauucuuaacucu | cacuuggccauucuuaacucugugcuauauaacugguggaguuuggagcugccaagaggu | manual\_scaffold\_4b:416289418..416289478:- |
| manual\_scaffold\_7\_27777 | 1.7e+2 | 0.96 ± 0.01 |  | 344 | 331 | 0 | 13 | yes |  |  |  | blast | aguuuggagcugccaagaggu | cacuuggccauacuuuaacucu | cacuuggccauacuuuaacucugugcuauaauggcuaguagaguuuggagcugccaagaggu | manual\_scaffold\_7:1220923427..1220923489:- |
| manual\_scaffold\_1a\_1237 | 1.7e+2 | 0.96 ± 0.01 |  | 343 | 330 | 0 | 13 | yes |  |  |  | blast | aguuuggagcugccaagaggu | cacuuggccauacuuuaacucu | cacuuggccauacuuuaacucugugauauaauaucuaguggaguuuggagcugccaagaggu | manual\_scaffold\_1a:463801800..463801862:- |
| manual\_scaffold\_4b\_15053 | 1.7e+2 | 0.96 ± 0.01 |  | 350 | 342 | 0 | 8 | no |  |  |  | blast | aagugcuuucuguugagcuuu | agcuucaacuugggcaccug | agcuucaacuugggcaccugguauguuuauauuuguaagugcuuucuguugagcuuu | manual\_scaffold\_4b:175066783..175066840:+ |
| manual\_scaffold\_8\_29765 | 1.7e+2 | 0.96 ± 0.01 |  | 348 | 331 | 0 | 17 | no |  |  |  | blast | aguuuggagcugccaagaggu | cacuuggccauuuuauaacucu | cacuuggccauuuuauaacucugugcuaugauagcuaguagaguuuggagcugccaagaggu | manual\_scaffold\_8:72348423..72348485:- |
| manual\_scaffold\_1a\_313 | 1.7e+2 | 0.96 ± 0.01 |  | 339 | 186 | 0 | 153 | yes |  |  |  | blast | gaagggaccauuguggucugga | gaggccacacaggacccaucuu | gaggccacacaggacccaucuucugaugauacuuuaagaagggaccauuguggucugga | manual\_scaffold\_1a:379953823..379953882:+ |
| manual\_scaffold\_4a\_13400 | 1.7e+2 | 0.96 ± 0.01 |  | 338 | 170 | 0 | 168 | yes |  |  |  | blast | aaggaccucaugauggaguaau | uuaccccaaauagauguccuccu | aaggaccucaugauggaguaauguuuccauuucauuacauuaccccaaauagauguccuccu | manual\_scaffold\_4a:727798369..727798431:+ |
| manual\_scaffold\_9\_33077 | 1.7e+2 | 0.96 ± 0.01 |  | 337 | 331 | 0 | 6 | yes |  |  |  | blast | aguuuggagcugccaagaggu | cgcuuggccauuccuuaacucu | cgcuuggccauuccuuaacucugugcuauaacagcuauuagaguuuggagcugccaagaggu | manual\_scaffold\_9:1017661182..1017661244:- |
| manual\_scaffold\_8\_30311 | 1.7e+2 | 0.96 ± 0.01 |  | 345 | 330 | 0 | 15 | no |  |  |  | blast | aguuuggagcugccaagaggu | cacguggccauucuuuaacucu | cacguggccauucuuuaacucugugcuauauaacuaguggaguuuggagcugccaagaggu | manual\_scaffold\_8:824329478..824329539:- |
| manual\_scaffold\_6\_21438 | 1.7e+2 | 0.96 ± 0.01 |  | 337 | 331 | 0 | 6 | yes |  |  |  | blast | aguuuggagcugccaagaggu | cgcuuggccauuccuuaacucu | cgcuuggccauuccuuaacucugugcuauaauagcuaguagaguuuggagcugccaagaggu | manual\_scaffold\_6:622713698..622713760:+ |
| manual\_scaffold\_6\_22717 | 1.7e+2 | 0.96 ± 0.01 |  | 337 | 331 | 0 | 6 | yes |  |  |  | blast | aguuuggagcugccaagaggu | cgcuuggccauuccuuaacucu | cgcuuggccauuccuuaacucugugcuaaaauagcuuauagaguuuggagcugccaagaggu | manual\_scaffold\_6:37747928..37747990:- |
| manual\_scaffold\_3b\_11969 | 1.7e+2 | 0.96 ± 0.01 |  | 337 | 331 | 0 | 6 | yes |  |  |  | blast | aguuuggagcugccaagaggu | cgcuuggccauuccuuaacucu | cgcuuggccauuccuuaacucuguguuauaacggcgaguagaguuuggagcugccaagaggu | manual\_scaffold\_3b:926741033..926741095:- |
| manual\_scaffold\_2a\_5637 | 1.7e+2 | 0.96 ± 0.01 |  | 343 | 331 | 0 | 12 | no |  |  |  | blast | aguuuggagcugccaagaggu | cacuugccauucuuuaacucu | cacuugccauucuuuaacucugugcuauaacggcuaguagaguuuggagcugccaagaggu | manual\_scaffold\_2a:1019280944..1019281005:- |
| manual\_scaffold\_12\_38234 | 1.7e+2 | 0.96 ± 0.01 |  | 341 | 331 | 0 | 10 | no |  |  |  | blast | aguuuggagcugccaagaggu | ugcuuggccauuguuuaacucu | ugcuuggccauuguuuaacucugugcuaugauagcuaguagaguuuggagcugccaagaggu | manual\_scaffold\_12:359564023..359564085:+ |
| manual\_scaffold\_4a\_13198 | 1.7e+2 | 0.96 ± 0.01 |  | 342 | 330 | 0 | 12 | no |  |  |  | blast | aguuuggagcugccaagaggu | cacuuggccauucguuaacucu | cacuuggccauucguuaacucugugcuacauaacuaguggaguuuggagcugccaagaggu | manual\_scaffold\_4a:621772833..621772894:+ |
| manual\_scaffold\_4b\_15081 | 1.6e+2 | 0.96 ± 0.01 |  | 333 | 279 | 0 | 54 | yes |  |  |  | blast | ugaauaguaucagaugucaugc | aggacaucuguacacacacaug | aggacaucuguacacacacaugccuuagauacaauaagcaugaauaguaucagaugucaugc | manual\_scaffold\_4b:190918155..190918217:+ |
| manual\_scaffold\_4a\_12910 | 1.6e+2 | 0.96 ± 0.01 |  | 331 | 330 | 0 | 1 | yes |  |  |  | blast | aguuuggagcugccaagaggu | cacuugggcauucuuuaac | cacuugggcauucuuuaacuccgugcuauauaacuaguggaguuuggagcugccaagaggu | manual\_scaffold\_4a:186214300..186214361:+ |
| manual\_scaffold\_12\_39961 | 1.6e+2 | 0.96 ± 0.01 |  | 322 | 299 | 1 | 22 | yes |  |  |  | blast | cagcauaccagguagaaccacauu | auguggcucuccugauaucagc | cagcauaccagguagaaccacauuuuuguuuuuuuuaaauguggcucuccugauaucagc | manual\_scaffold\_12:694217383..694217443:- |
| manual\_scaffold\_4b\_15664 | 1.5e+2 | 0.96 ± 0.01 |  | 308 | 299 | 0 | 9 | yes |  |  |  | blast | ugacuuugguguugccuggaagu | uuccgggccccucuaaaguuc | uuccgggccccucuaaaguucagcgccauaacggcaucugacuuugguguugccuggaagu | manual\_scaffold\_4b:816044479..816044540:+ |
| manual\_scaffold\_12\_39834 | 1.5e+2 | 0.96 ± 0.01 |  | 305 | 303 | 0 | 2 | yes |  |  |  | blast | gaacuugugaucugacggacgag | ccgccugccauuuuacaaguuu | ccgccugccauuuuacaaguuucauagaauauaauggaacuugugaucugacggacgag | manual\_scaffold\_12:635099307..635099366:- |
| manual\_scaffold\_1b\_2462 | 1.5e+2 | 0.96 ± 0.01 |  | 302 | 204 | 0 | 98 | yes |  |  |  | blast | aguggugacagagaaagugcuc | uacacuuucucuggcaccuaac | aguggugacagagaaagugcucuuuaaaguuuaaauauacacuuucucuggcaccuaac | manual\_scaffold\_1b:828501101..828501160:+ |
| manual\_scaffold\_5\_18484 | 1.5e+2 | 0.96 ± 0.01 |  | 300 | 186 | 113 | 1 | yes |  |  |  | blast | cucuggcgcuacuuucuc | gaaagagucgccccuuuuugggc | cucuggcgcuacuuucucacugucacggucuguccuuggcguuuccucgccuacagcaccguggcuaccagaaagagucgccccuuuuugggc | manual\_scaffold\_5:1155049078..1155049171:+ |
| manual\_scaffold\_3b\_10085 | 1.4e+2 | 0.96 ± 0.01 |  | 293 | 279 | 0 | 14 | yes |  |  |  | blast | cacuccacaucuacguaccuuga | aagguaaauauaugugggguuc | cacuccacaucuacguaccuugaagacauauucauagucaagguaaauauaugugggguuc | manual\_scaffold\_3b:97700124..97700185:+ |
| manual\_scaffold\_10\_33822 | 1.4e+2 | 0.96 ± 0.01 |  | 290 | 224 | 0 | 66 | yes |  |  |  | blast | acaugcgugguuaaggcacaga | cggugccuuaaucacacaugug | cggugccuuaaucacacaugugcugaacaacgcacaugcgugguuaaggcacaga | manual\_scaffold\_10:328118140..328118195:+ |
| manual\_scaffold\_4b\_15973 | 1.4e+2 | 0.96 ± 0.01 |  | 287 | 207 | 0 | 80 | yes |  |  |  | blast | cacuuggacucuuucaggcucu | agcuuuggaguugucuggugau | cacuuggacucuuucaggcucugugguauaucuauuggagcuuuggaguugucuggugau | manual\_scaffold\_4b:1123687645..1123687705:+ |
| manual\_scaffold\_1a\_122 | 1.4e+2 | 0.96 ± 0.01 |  | 294 | 251 | 0 | 43 | no |  |  |  | blast | agucuuggagcugucuaguggu | ucuuggacuuucccagucucu | ucuuggacuuucccagucucugugauucaucuaucagagucuuggagcugucuaguggu | manual\_scaffold\_1a:131012642..131012701:+ |
| manual\_scaffold\_1a\_128 | 1.4e+2 | 0.96 ± 0.01 |  | 294 | 251 | 0 | 43 | no |  |  |  | blast | agucuuggagcugucuaguggu | ucuuggacuuucccagucucu | ucuuggacuuucccagucucugugauucaucuaucagagucuuggagcugucuaguggu | manual\_scaffold\_1a:131166596..131166655:+ |
| manual\_scaffold\_1a\_124 | 1.4e+2 | 0.96 ± 0.01 |  | 294 | 251 | 0 | 43 | no |  |  |  | blast | agucuuggagcugucuaguggu | ucuuggacuuucccagucucu | ucuuggacuuucccagucucugugauucaucuaucagagucuuggagcugucuaguggu | manual\_scaffold\_1a:131064281..131064340:+ |
| manual\_scaffold\_1a\_126 | 1.4e+2 | 0.96 ± 0.01 |  | 294 | 251 | 0 | 43 | no |  |  |  | blast | agucuuggagcugucuaguggu | ucuuggacuuucccagucucu | ucuuggacuuucccagucucugugauucaucuaucagagucuuggagcugucuaguggu | manual\_scaffold\_1a:131115845..131115904:+ |
| manual\_scaffold\_10\_33415 | 1.4e+2 | 0.96 ± 0.01 |  | 281 | 266 | 0 | 15 | yes |  |  |  | blast | auuuugccaccuacaacugcagc | ugcagugcaggcgguggaacau | auuuugccaccuacaacugcagcacugguugauuaagcacugcagugcaggcgguggaacau | manual\_scaffold\_10:37449416..37449478:+ |
| manual\_scaffold\_4b\_17430 | 1.4e+2 | 0.96 ± 0.01 |  | 274 | 271 | 0 | 3 | yes |  |  |  | blast | ucacucauggagacaguuagac | cugacugucucagaguaguggagc | cugacugucucagaguaguggagcagaacagucaaugcucacucauggagacaguuagac | manual\_scaffold\_4b:1160860226..1160860286:- |
| manual\_scaffold\_4a\_14394 | 1.3e+2 | 0.96 ± 0.01 |  | 268 | 162 | 0 | 106 | yes |  |  |  | blast | gaacuucuguucuuagugcacu | ugugcuggaacaaggaguuuc | gaacuucuguucuuagugcacuguuuuaaaaacuagugugcuggaacaaggaguuuc | manual\_scaffold\_4a:411468308..411468365:- |
| manual\_scaffold\_1a\_1270 | 1.3e+2 | 0.96 ± 0.01 |  | 266 | 265 | 0 | 1 | yes |  |  |  | blast | uuuugucuuucaucugcuggcu | cagcagaugaagaauaaaac | cagcagaugaagaauaaaacaauagcaagcuauuguuuugucuuucaucugcuggcu | manual\_scaffold\_1a:491349031..491349088:- |
| manual\_scaffold\_7\_25947 | 1.3e+2 | 0.96 ± 0.01 |  | 257 | 255 | 0 | 2 | yes |  |  |  | blast | uacuguggaaaugagaccuuugu | gaggucaucaauuucacuguugcc | gaggucaucaauuucacuguugccgggcuacuaaagguuacuguggaaaugagaccuuugu | manual\_scaffold\_7:1299619271..1299619332:+ |
| manual\_scaffold\_11\_37755 | 1.3e+2 | 0.96 ± 0.01 |  | 264 | 246 | 0 | 18 | no |  |  |  | blast | cugccuccccugcaaacgucca | acguuggcucugguggugau | cugccuccccugcaaacguccagugaugacguggauauggacguuggcucugguggugau | manual\_scaffold\_11:1015135179..1015135239:- |
| manual\_scaffold\_3b\_12172 | 1.2e+2 | 0.96 ± 0.01 |  | 252 | 245 | 0 | 7 | yes |  |  |  | blast | uaugagagcugcgucuggauu | auccaggcgcuucugucaugcu | auccaggcgcuucugucaugcugguuaacaguaugagagcugcgucuggauu | manual\_scaffold\_3b:1140549697..1140549749:- |
| manual\_scaffold\_1a\_118 | 1.2e+2 | 0.96 ± 0.01 |  | 259 | 251 | 0 | 8 | no |  |  |  | blast | agucuuggagcugucuaguggu | cucuuggacucucccauucucu | cucuuggacucucccauucucugugauucaucuaucagagucuuggagcugucuaguggu | manual\_scaffold\_1a:128183687..128183747:+ |
| manual\_scaffold\_5\_19821 | 1.2e+2 | 0.96 ± 0.01 |  | 256 | 173 | 0 | 83 | no |  |  |  | blast | caccaggccacuccaagaaucu | gauuuggaggugcccggaggu | caccaggccacuccaagaaucugugcuacaagagcauucagauuuggaggugcccggaggu | manual\_scaffold\_5:835698750..835698811:- |
| manual\_scaffold\_10\_34058 | 1.2e+2 | 0.96 ± 0.01 |  | 247 | 117 | 0 | 130 | yes |  |  |  | blast | cgugugcucuguguccucugcc | auagaggacacagagcacacugu | auagaggacacagagcacacuguguauguguguguuuccacgugugcucuguguccucugcc | manual\_scaffold\_10:671530913..671530975:+ |
| manual\_scaffold\_6\_23731 | 1.2e+2 | 0.96 ± 0.01 |  | 242 | 224 | 0 | 18 | yes |  |  |  | blast | acaugcgugguuaaggcacaga | ugugccuuaacuacccaugugc | ugugccuuaacuacccaugugcugaacaacgcacaugcgugguuaaggcacaga | manual\_scaffold\_6:779298736..779298790:- |
| manual\_scaffold\_1b\_2096 | 1.2e+2 | 0.96 ± 0.01 |  | 250 | 78 | 1 | 171 | no |  |  |  | blast | acuuggacacuguuauacucug | aguauuggagcugucuuguggu | acuuggacacuguuauacucugugacauucuaaucagaguauuggagcugucuuguggu | manual\_scaffold\_1b:352865332..352865391:+ |
| manual\_scaffold\_5\_20133 | 1.2e+2 | 0.96 ± 0.01 |  | 240 | 159 | 77 | 4 | yes |  |  |  | blast | uuguccuuuccagaagcug | uuucuguaaaaaugagcauc | uuguccuuuccagaagcugaccuucaacugcggcaccugcuacgauuauauuuguaggugcuucuuuugagguucaguuucuguaaaaaugagcauc | manual\_scaffold\_5:1251410008..1251410105:- |
| manual\_scaffold\_6\_23965 | 1.2e+2 | 0.96 ± 0.01 |  | 237 | 123 | 0 | 114 | yes |  |  |  | blast | uucgaauucugggacuucugguc | uacaagucccagaauucaaaga | uucgaauucugggacuucuggucaugauuuguacuggaauaaacaugacuacaagucccagaauucaaaga | manual\_scaffold\_6:1047182026..1047182097:- |
| manual\_scaffold\_3b\_10036 | 1.2e+2 | 0.96 ± 0.01 |  | 240 | 239 | 0 | 1 | yes |  |  |  | blast | ugacugaagauuccaauuguucu | auauuuggagauucaguauuc | ugacugaagauuccaauuguucuuuaauaucugacuaaaaauauuuggagauucaguauuc | manual\_scaffold\_3b:90381119..90381180:+ |
| manual\_scaffold\_6\_24190 | 1.2e+2 | 0.96 ± 0.01 |  | 238 | 237 | 0 | 1 | yes |  |  |  | blast | aaccuuguacuuguagacuuau | ugguuuaugggugcaagguagu | ugguuuaugggugcaagguagugagaacaaaaaggcaaccuuguacuuguagacuuau | manual\_scaffold\_6:1359161114..1359161172:- |
| manual\_scaffold\_9\_33027 | 1.1e+2 | 0.96 ± 0.01 |  | 233 | 203 | 0 | 30 | yes |  |  |  | blast | cgcuuagacucucucagucucu | agucuaggagcugucugguggu | cgcuuagacucucucagucucugugauuauucuaucagagucuaggagcugucugguggu | manual\_scaffold\_9:960862885..960862945:- |
| manual\_scaffold\_12\_39874 | 1.1e+2 | 0.96 ± 0.01 |  | 231 | 222 | 0 | 9 | yes |  |  |  | blast | uugaauucugggacuuguaguc | cacaaguccuagaauucaaag | uugaauucugggacuuguaguccuguuuuauaaugcaauaaacaccaccacaaguccuagaauucaaag | manual\_scaffold\_12:666555762..666555831:- |
| manual\_scaffold\_4b\_17309 | 1.1e+2 | 0.96 ± 0.01 |  | 229 | 111 | 0 | 118 | yes |  |  |  | blast | uuaucauuuuauuuuucacuggc | agacgaaagauuaaaugauaau | agacgaaagauuaaaugauaauaaaauaauuauguuaucauuuuauuuuucacuggc | manual\_scaffold\_4b:1033418112..1033418169:- |
| manual\_scaffold\_12\_38758 | 1.1e+2 | 0.96 ± 0.01 |  | 224 | 223 | 0 | 1 | yes |  |  |  | blast | uacacauacgaugaacagaaaau | caucuguucauggcauguuguguu | uacacauacgaugaacagaaaauguuacuuaaaagcaucuguucauggcauguuguguu | manual\_scaffold\_12:695003238..695003297:+ |
| manual\_scaffold\_1a\_186 | 1.0e+2 | 0.96 ± 0.01 |  | 204 | 197 | 0 | 7 | yes |  |  |  | blast | augagagaagcgucuggauugg | aucuagguacuucucuuaugcu | aucuagguacuucucuuaugcugguuaacagcaugagagaagcgucuggauugg | manual\_scaffold\_1a:199396281..199396335:+ |
| manual\_scaffold\_3b\_11861 | 1.0e+2 | 0.96 ± 0.01 |  | 211 | 69 | 0 | 142 | no |  |  |  | blast | ucuugaacucucccagccucu | gagucuuggagcuguucagu | ucuugaacucucccagccucugugauuuaacuaucagagucuuggagcuguucagu | manual\_scaffold\_3b:815450169..815450225:- |
| manual\_scaffold\_9\_32612 | 1.0e+2 | 0.96 ± 0.01 |  | 203 | 91 | 0 | 112 | yes |  |  |  | blast | cgcuuggcuucaucaauacucagu | ugaguugguggcgccgggaga | cgcuuggcuucaucaauacucaguauucuauaacuuccugaguugguggcgccgggaga | manual\_scaffold\_9:480864086..480864145:- |
| manual\_scaffold\_8\_28568 | 9.9e+1 | 0.96 ± 0.01 |  | 194 | 162 | 27 | 5 | yes |  |  |  | blast | uuguccuuuccagaagcug | guuucuguaaaaugagcauc | uuguccuuuccagaagcugagcuucgacuguggcaccugcuacguuuauauugguaagugcuucuuugagguuuaguuucuguaaaaugagcauc | manual\_scaffold\_8:75601244..75601339:+ |
| manual\_scaffold\_2a\_4953 | 9.9e+1 | 0.96 ± 0.01 |  | 193 | 185 | 0 | 8 | yes |  |  |  | blast | uaaccaauaccugacacccaa | uggguggcagguauuggucauu | uaaccaauaccugacacccaaagcuauagaaauaacuuggguggcagguauuggucauu | manual\_scaffold\_2a:60978216..60978275:- |
| manual\_scaffold\_2a\_5542 | 9.8e+1 | 0.96 ± 0.01 |  | 191 | 189 | 0 | 2 | yes |  |  |  | blast | uagagggaucgccuuaguguc | cucuagggcgaccugcucuccc | cucuagggcgaccugcucucccuuguuaccgcauugguagagggaucgccuuaguguc | manual\_scaffold\_2a:833345308..833345366:- |
| manual\_scaffold\_7\_27200 | 9.7e+1 | 0.96 ± 0.01 |  | 191 | 187 | 0 | 4 | yes |  |  |  | blast | caucggacuacaaguaccaga | ggguguuuguagugcuguguu | ggguguuuguagugcuguguucauaacaggaaaacaucggacuacaaguaccaga | manual\_scaffold\_7:672260074..672260129:- |
| manual\_scaffold\_9\_31794 | 9.6e+1 | 0.96 ± 0.01 |  | 193 | 168 | 0 | 25 | yes |  |  |  | blast | aacuccacauguacaacccuug | aagguacguagaugugggguuu | aacuccacauguacaacccuugguuauauauauaucucaaagguacguagaugugggguuu | manual\_scaffold\_9:875873596..875873657:+ |
| manual\_scaffold\_3a\_9056 | 9.1e+1 | 0.96 ± 0.01 |  | 178 | 176 | 0 | 2 | yes |  |  |  | blast | ugggucaguagcuucugguugc | uccagaagguacugguccccu | uccagaagguacugguccccucuuugguaauguugaagugggucaguagcuucugguugc | manual\_scaffold\_3a:316392639..316392699:- |
| manual\_scaffold\_8\_29909 | 9.1e+1 | 0.96 ± 0.01 |  | 187 | 176 | 0 | 11 | no |  |  |  | blast | uagagcagucuaaguugauggc | cauggacgagauuugcuucuga | uagagcagucuaaguugauggcauacacucuuggucauggacgagauuugcuucuga | manual\_scaffold\_8:259519954..259520011:- |
| manual\_scaffold\_9\_32489 | 9.1e+1 | 0.96 ± 0.01 |  | 177 | 163 | 0 | 14 | yes |  |  |  | blast | ucggcacugcuaucaagcugc | agcaugagagcagcgucuggau | ucggcacugcuaucaagcugcugaacauuaucagcagcaugagagcagcgucuggau | manual\_scaffold\_9:311397665..311397722:- |
| manual\_scaffold\_6\_21842 | 8.9e+1 | 0.96 ± 0.01 |  | 175 | 167 | 0 | 8 | yes |  |  |  | blast | ugccuggcuucaucuuaacucug | ugaguuggugacgcugggaggu | ugccuggcuucaucuuaacucugugcuauaauagcuuucugaguuggugacgcugggaggu | manual\_scaffold\_6:1050885762..1050885823:+ |
| manual\_scaffold\_8\_30488 | 8.5e+1 | 0.96 ± 0.01 |  | 167 | 106 | 0 | 61 | yes |  |  |  | blast | auauauuuuucugcucggacc | auucgagcaaaaaaugucuug | auucgagcaaaaaaugucuugugaaaauuguguguugcaauauauuuuucugcucggacc | manual\_scaffold\_8:1105153567..1105153627:- |
| manual\_scaffold\_6\_22606 | 8.4e+1 | 0.96 ± 0.01 |  | 165 | 162 | 0 | 3 | yes |  |  |  | blast | ccugugcauucugauacuugu | caagucccagaaugcaucgaaa | ccugugcauucugauacuuguaguccagcauacacuacugguaaaguaaaacuacaagucccagaaugcaucgaaa | manual\_scaffold\_6:1744211929..1744212005:+ |
| manual\_scaffold\_2b\_6207 | 8.3e+1 | 0.96 ± 0.01 |  | 164 | 157 | 0 | 7 | yes |  |  |  | blast | gucugagcaaacaucuguugga | uaguggaugcaggguucagauaau | uaguggaugcaggguucagauaaucaguuaaagucugagcaaacaucuguugga | manual\_scaffold\_2b:401967353..401967407:+ |
| manual\_scaffold\_7\_25366 | 8.1e+1 | 0.96 ± 0.01 |  | 158 | 56 | 0 | 102 | yes |  |  |  | blast | ucugaacacacaucuguugga | uagcagaugcagaauucagaaa | uagcagaugcagaauucagaaaaucaguuaaaaucugaacacacaucuguugga | manual\_scaffold\_7:720293194..720293248:+ |
| manual\_scaffold\_8\_29875 | 7.9e+1 | 0.96 ± 0.01 |  | 155 | 147 | 0 | 8 | yes |  |  |  | blast | ccagcacuguucuuaaccugc | aguauaagagcagugcuuggau | ccagcacuguucuuaaccugcuuaguauuauaugcaguauaagagcagugcuuggau | manual\_scaffold\_8:211664421..211664478:- |
| manual\_scaffold\_9\_32755 | 7.9e+1 | 0.96 ± 0.01 |  | 154 | 128 | 0 | 26 | yes |  |  |  | blast | uugcggucauguuaguccucacc | ugaggacuuucaagagggcaau | ugaggacuuucaagagggcaauguacucuucauugcggucauguuaguccucacc | manual\_scaffold\_9:692576355..692576410:- |
| manual\_scaffold\_7\_25107 | 7.9e+1 | 0.96 ± 0.01 |  | 167 | 71 | 0 | 96 | no |  |  |  | blast | ugccauuuucaugaucucaaagguc | ccugagauuuuguaaaugugaa | ugccauuuucaugaucucaaaggucgaaaguguauaguccugagauuuuguaaaugugaa | manual\_scaffold\_7:443455137..443455197:+ |
| manual\_scaffold\_2a\_3912 | 7.8e+1 | 0.96 ± 0.01 |  | 152 | 151 | 0 | 1 | yes |  |  |  | blast | ucugggacuuguauucuugcu | caacacuacaagucacagaauu | ucugggacuuguauucuugcuucuugaaauauaagagcaacacuacaagucacagaauu | manual\_scaffold\_2a:105122903..105122962:+ |
| manual\_scaffold\_7\_26666 | 7.5e+1 | 0.96 ± 0.01 |  | 147 | 143 | 0 | 4 | yes |  |  |  | blast | aaugauucucagaacuagguuc | cacuaguucccagaaugcauuuu | cacuaguucccagaaugcauuuugugaguaggaaaugauucucagaacuagguuc | manual\_scaffold\_7:127437948..127438003:- |
| manual\_scaffold\_1a\_717 | 7.3e+1 | 0.96 ± 0.01 |  | 143 | 141 | 0 | 2 | yes |  |  |  | blast | caugagaagccucuggauuggc | caguccagacgcugcucucaugcu | caguccagacgcugcucucaugcuuuuagcaauguuaagcggcaugagaagccucuggauuggc | manual\_scaffold\_1a:907557066..907557130:+ |
| manual\_scaffold\_11\_35926 | 7.3e+1 | 0.96 ± 0.01 |  | 144 | 143 | 0 | 1 | yes |  |  |  | blast | uucauuuugcuacagccugcugg | aacagcuguuucaaagugcaggu | uucauuuugcuacagccugcuggugauaguaucaauccaacagcuguuucaaagugcaggu | manual\_scaffold\_11:77332750..77332811:+ |
| manual\_scaffold\_8\_29711 | 7.2e+1 | 0.96 ± 0.01 |  | 141 | 137 | 0 | 4 | yes |  |  |  | blast | aauaucacaccugguaauuacug | guaauucugugugugguauua | aauaucacaccugguaauuacugcaguccuugguaucaguaauucugugugugguauua | manual\_scaffold\_8:54296756..54296815:- |
| manual\_scaffold\_8\_29589 | 7.1e+1 | 0.96 ± 0.01 |  | 140 | 96 | 0 | 44 | yes |  |  |  | blast | cggggacuuccuucagagcugcu | ugcuacgaagcaguccucaga | cggggacuuccuucagagcugcuuuaaaaauagcugcuacgaagcaguccucaga | manual\_scaffold\_8:1490198030..1490198085:+ |
| manual\_scaffold\_3b\_10752 | 7.1e+1 | 0.96 ± 0.01 |  | 140 | 132 | 0 | 8 | yes |  |  |  | blast | aguuuuggagcugucuaguggu | cgcuucgacucucucaggcucu | cgcuucgacucucucaggcucugugauuuuuucuaucagaguuuuggagcugucuaguggu | manual\_scaffold\_3b:850291326..850291387:+ |
| manual\_scaffold\_5\_18198 | 7.1e+1 | 0.96 ± 0.01 |  | 138 | 55 | 0 | 83 | yes |  |  |  | blast | caucuguuuguggcauguagu | auauauggcaucgaacagauuguu | auauauggcaucgaacagauuguuaauuacccuguaagcaucuguuuguggcauguagu | manual\_scaffold\_5:739900110..739900169:+ |
| manual\_scaffold\_10\_35278 | 6.9e+1 | 0.96 ± 0.01 |  | 136 | 135 | 0 | 1 | yes |  |  |  | blast | auuggcugugcacugggcuucc | uggcugaguucauagccaugagc | uggcugaguucauagccaugagcauauucguuuucaucagcauuggcugugcacugggcuucc | manual\_scaffold\_10:603559374..603559437:- |
| manual\_scaffold\_10\_35819 | 6.9e+1 | 0.96 ± 0.01 |  | 135 | 132 | 0 | 3 | yes |  |  |  | blast | ucagaauucuggaacuuguagu | uacaagucccagaauucaauga | ucagaauucuggaacuuguaguucgguuuuauaaugaauaaacaaaacuacaagucccagaauucaauga | manual\_scaffold\_10:1082687136..1082687206:- |
| manual\_scaffold\_8\_29923 | 6.9e+1 | 0.96 ± 0.01 |  | 136 | 102 | 0 | 34 | yes |  |  |  | blast | uguguucaugacuggauauacu | uaaaucucagaucguguucacgcu | uguguucaugacuggauauacuuucccuugacaugaguaaaucucagaucguguucacgcu | manual\_scaffold\_8:276431814..276431875:- |
| manual\_scaffold\_3b\_10880 | 6.8e+1 | 0.96 ± 0.01 |  | 134 | 109 | 0 | 25 | yes |  |  |  | blast | cgccuggcuccaucauaacucugu | ugaguuggugaugccgggaggu | cgccuggcuccaucauaacucugugcuauaauagcuuucugaguuggugaugccgggaggu | manual\_scaffold\_3b:995736322..995736383:+ |
| manual\_scaffold\_3a\_7338 | 6.8e+1 | 0.96 ± 0.01 |  | 131 | 129 | 0 | 2 | yes |  |  |  | blast | uugcgucaaucaauacggacu | uccguauugauugacgcaaagu | uugcgucaaucaauacggacuagacacacacacuaguccguauugauugacgcaaagu | manual\_scaffold\_3a:110087856..110087914:+ |
| manual\_scaffold\_3a\_8795 | 6.8e+1 | 0.96 ± 0.01 |  | 131 | 129 | 0 | 2 | yes |  |  |  | blast | uugcgucaaucaauacggacu | uccguauugauugacgcaaagu | uugcgucaaucaauacggacuagugugugugucuaguccguauugauugacgcaaagu | manual\_scaffold\_3a:110087853..110087911:- |
| manual\_scaffold\_1a\_1399 | 6.7e+1 | 0.96 ± 0.01 |  | 130 | 117 | 0 | 13 | yes |  |  |  | blast | cuuggcgcaucuuucaugcug | agcaugagaguagcgucagguu | cuuggcgcaucuuucaugcugguuaauauuaucagcagcaugagaguagcgucagguu | manual\_scaffold\_1a:679515629..679515687:- |
| manual\_scaffold\_5\_18836 | 6.6e+1 | 0.96 ± 0.01 |  | 132 | 131 | 0 | 1 | yes |  |  |  | blast | aguuuuggagcugucuaguggu | cacuuggacacucucaggcucu | cacuuggacacucucaggcucugugguauaucuauuggaguuuuggagcugucuaguggu | manual\_scaffold\_5:1600853789..1600853849:+ |
| manual\_scaffold\_4a\_13169 | 6.6e+1 | 0.96 ± 0.01 |  | 130 | 90 | 0 | 40 | yes |  |  |  | blast | ugacuggcauguggauauauugu | uuuguguccacgucaggcacu | uuuguguccacgucaggcacugcguaauuauaucaaacggugacuggcauguggauauauugu | manual\_scaffold\_4a:594593889..594593952:+ |
| manual\_scaffold\_3b\_12211 | 6.6e+1 | 0.96 ± 0.01 |  | 130 | 125 | 0 | 5 | yes |  |  |  | blast | agcacugcaggaaucucaacuc | guugggaauucugugugcuuu | agcacugcaggaaucucaacucguuaaacacccaaagaguugggaauucugugugcuuu | manual\_scaffold\_3b:1195885960..1195886019:- |
| manual\_scaffold\_4b\_16551 | 6.6e+1 | 0.96 ± 0.01 |  | 128 | 123 | 0 | 5 | yes |  |  |  | blast | uuugguccuacacuguuuagag | ucuaggcacuguagagccauuu | uuugguccuacacuguuuagaggcaaaaaaauugccucuaggcacuguagagccauuu | manual\_scaffold\_4b:133546170..133546228:- |
| manual\_scaffold\_4b\_16814 | 6.5e+1 | 0.96 ± 0.01 |  | 134 | 130 | 0 | 4 | no |  |  |  | blast | ccccgcgccgaugacaacaccu | agugcugucaucggugcagagcc | agugcugucaucggugcagagccgucgggccgggggcggccccgcgccgaugacaacaccu | manual\_scaffold\_4b:387496674..387496735:- |
| manual\_scaffold\_8\_29658 | 6.5e+1 | 0.96 ± 0.01 |  | 128 | 124 | 0 | 4 | yes |  |  |  | blast | ucuacccggacuaacgucauuu | augacgcuugccugguuagaguc | augacgcuugccugguuagagucaucuuuuuuuacucuacccggacuaacgucauuu | manual\_scaffold\_8:6711343..6711400:- |
| manual\_scaffold\_4a\_14202 | 6.4e+1 | 0.96 ± 0.01 |  | 126 | 116 | 0 | 10 | yes |  |  |  | blast | augucauucagaacccuuggua | ccucaggguucugacugacacc | ccucaggguucugacugacaccauggacacguauuugaugucauucagaacccuuggua | manual\_scaffold\_4a:182121742..182121801:- |
| manual\_scaffold\_6\_21738 | 6.3e+1 | 0.96 ± 0.01 |  | 122 | 119 | 0 | 3 | yes |  |  |  | blast | uucuguacccuuggcccccagu | uugggggccuaggguacagaa | uucuguacccuuggcccccaguguuaacacacugcauugggggccuaggguacagaa | manual\_scaffold\_6:932622323..932622380:+ |
| manual\_scaffold\_8\_30363 | 6.2e+1 | 0.96 ± 0.01 |  | 123 | 99 | 0 | 24 | yes |  |  |  | blast | gcgugcauuucgauugugaacu | uucacagucuuccaaugacg | gcgugcauuucgauugugaacugcauuuaguauaguucacagucuuccaaugacg | manual\_scaffold\_8:913968917..913968972:- |
| manual\_scaffold\_5\_19554 | 6.1e+1 | 0.96 ± 0.01 |  | 128 | 108 | 0 | 20 | no |  |  |  | blast | uuggcaccugaggaucuggaau | acccagaucacaggaacccaaau | acccagaucacaggaacccaaauggucaaccuagcaguuuggcaccugaggaucuggaau | manual\_scaffold\_5:534044307..534044367:- |
| manual\_scaffold\_6\_22061 | 6.1e+1 | 0.96 ± 0.01 |  | 120 | 119 | 0 | 1 | yes |  |  |  | blast | uggaacacagaaaucuucacu | cuggagauuuuugaguucc | cuggagauuuuugaguuccaaaaaaaugacuaauuuggaacacagaaaucuucacu | manual\_scaffold\_6:1253764793..1253764849:+ |
| manual\_scaffold\_8\_29388 | 6.1e+1 | 0.96 ± 0.01 |  | 128 | 99 | 0 | 29 | no |  |  |  | blast | cacuuggccacucuaacacucugc | agaguuggagcugccaggaggu | cacuuggccacucuaacacucugcacuguuaaggcuuguagaguuggagcugccaggaggu | manual\_scaffold\_8:1238267409..1238267470:+ |
| manual\_scaffold\_6\_22059 | 6.1e+1 | 0.96 ± 0.01 |  | 120 | 119 | 0 | 1 | yes |  |  |  | blast | uggaacacagaaaucuucacu | cuggagauuuuugaguucc | cuggagauuuuugaguuccaaaaaaaugacuaauuuggaacacagaaaucuucacu | manual\_scaffold\_6:1253757746..1253757802:+ |
| manual\_scaffold\_7\_27373 | 5.9e+1 | 0.96 ± 0.01 |  | 116 | 61 | 0 | 55 | yes |  |  |  | blast | cuggcagcuuugauggugugc | ugcaucaucauagaggccuggc | ugcaucaucauagaggccuggcuaugguuaagccucgccuggcagcuuugauggugugc | manual\_scaffold\_7:888811563..888811622:- |
| manual\_scaffold\_4a\_14226 | 5.9e+1 | 0.96 ± 0.01 |  | 113 | 112 | 0 | 1 | yes |  |  |  | blast | uugguauguaggauuccgguga | accggaauccuacauaccaaga | accggaauccuacauaccaagauuuguguuuuaaaugacaaaucuugguauguaggauuccgguga | manual\_scaffold\_4a:239145478..239145544:- |
| manual\_scaffold\_4b\_15853 | 5.9e+1 | 0.96 ± 0.01 |  | 115 | 90 | 0 | 25 | yes |  |  |  | blast | uacuuuacaacuuucggcacag | cgccgaaaguuguaaaguagu | cgccgaaaguuguaaaguagucuaauuaaagacuacuuuacaacuuucggcacag | manual\_scaffold\_4b:1050698266..1050698321:+ |
| manual\_scaffold\_4a\_12945 | 5.9e+1 | 0.96 ± 0.01 |  | 113 | 112 | 0 | 1 | yes |  |  |  | blast | uugguauguaggauuccgguga | accggaauccuacauaccaaga | accggaauccuacauaccaagauuugucauuuaaaacacaaaucuugguauguaggauuccgguga | manual\_scaffold\_4a:239145480..239145546:+ |
| manual\_scaffold\_5\_19901 | 5.8e+1 | 0.96 ± 0.01 |  | 112 | 111 | 0 | 1 | yes |  |  |  | blast | cuagcaaugcucuuauacuguu | agcauaagaguagugcuuggau | cuagcaaugcucuuauacuguuuaguauuauaugcagcauaagaguagugcuuggau | manual\_scaffold\_5:970426288..970426345:- |
| manual\_scaffold\_3a\_7405 | 5.6e+1 | 0.96 ± 0.01 |  | 110 | 87 | 0 | 23 | yes |  |  |  | blast | ugacugucagcagucucgcc | gcgggacugcaucagugcaaaga | gcgggacugcaucagugcaaagauuacucuuuuuugacugucagcagucucgcc | manual\_scaffold\_3a:184096004..184096058:+ |
| manual\_scaffold\_7\_26988 | 5.6e+1 | 0.96 ± 0.01 |  | 111 | 67 | 0 | 44 | yes |  |  |  | blast | uccucgucuccguguccugaau | uucaugucgcgaaggcuaga | uccucgucuccguguccugaauagaauugacaauucaugucgcgaaggcuaga | manual\_scaffold\_7:441503486..441503539:- |
| manual\_scaffold\_2a\_3959 | 5.5e+1 | 0.96 ± 0.01 |  | 117 | 113 | 0 | 4 | no |  |  |  | blast | ucuacaaguuggaacuuacaga | uuuaagguccugcaaugucagagu | uuuaagguccugcaaugucagaguguacucuuucuacuuaacucuacaaguuggaacuuacaga | manual\_scaffold\_2a:172806086..172806150:+ |
| manual\_scaffold\_3a\_8458 | 5.4e+1 | 0.96 ± 0.01 |  | 106 | 103 | 0 | 3 | yes |  |  |  | blast | acacugcacauuuugacacu | caggcagaaaaugcaguauggu | acacugcacauuuugacacugguccuagaaaugcagccaggcagaaaaugcaguauggu | manual\_scaffold\_3a:743371759..743371818:+ |
| manual\_scaffold\_11\_37355 | 5.4e+1 | 0.96 ± 0.01 |  | 113 | 79 | 0 | 34 | no |  |  |  | blast | cacucggacucucucagacucu | gaguuuuggagcugucuagu | cacucggacucucucagacucugugauauuucuaucagaguuuuggagcugucuagu | manual\_scaffold\_11:642988543..642988600:- |
| manual\_scaffold\_2a\_4023 | 5.3e+1 | 0.96 ± 0.01 |  | 104 | 102 | 0 | 2 | yes |  |  |  | blast | cugcaucguaguuaggucucaga | ugagaccucccucauugcauc | ugagaccucccucauugcaucaugucacuacuugcugcaucguaguuaggucucaga | manual\_scaffold\_2a:282180676..282180733:+ |
| manual\_scaffold\_6\_22293 | 5.1e+1 | 0.96 ± 0.01 |  | 97 | 94 | 2 | 1 | yes |  |  |  | blast | ccggacgacgcuuuccggcucc | gccggaaagcguuguccgguc | gccggaaagcguuguccggucagaacagaggcggaccggacgacgcuuuccggcucc | manual\_scaffold\_6:1488970884..1488970941:+ |
| manual\_scaffold\_7\_25770 | 5.1e+1 | 0.96 ± 0.01 |  | 107 | 104 | 0 | 3 | no |  |  |  | blast | cucagacugccuuuuccaggug | ccuggaaggguucgucuggauc | cucagacugccuuuuccagguguguuacccaguggacaaguuuucuccuggaaggguucgucuggauc | manual\_scaffold\_7:1108527879..1108527947:+ |
| manual\_scaffold\_2a\_4581 | 5.0e+1 | 0.96 ± 0.01 |  | 97 | 80 | 0 | 17 | yes |  |  |  | blast | cuuggcugagcgcugcauccu | cgaugcaucucugagccaaauc | cgaugcaucucugagccaaaucgaugcaucgccuuggcugagcgcugcauccu | manual\_scaffold\_2a:1015756186..1015756239:+ |
| manual\_scaffold\_1b\_3353 | 4.9e+1 | 0.96 ± 0.01 |  | 93 | 63 | 0 | 30 | yes |  |  |  | blast | caggucaacguacucucaccu | ugagaguacguugaccuga | caggucaacguacucucaccuccguucccaccgggggaggugagaguacguugaccuga | manual\_scaffold\_1b:607628261..607628320:- |
| manual\_scaffold\_1b\_2317 | 4.9e+1 | 0.96 ± 0.01 |  | 93 | 63 | 0 | 30 | yes |  |  |  | blast | caggucaacguacucucaccu | ugagaguacguugaccuga | caggucaacguacucucaccucccccggugggaacggaggugagaguacguugaccuga | manual\_scaffold\_1b:607628262..607628321:+ |
| manual\_scaffold\_4b\_16829 | 4.9e+1 | 0.96 ± 0.01 |  | 96 | 94 | 0 | 2 | yes |  |  |  | blast | acgggaggggccuuaugaaucacu | ugaaccauagucccuccccaca | acgggaggggccuuaugaaucacucaagguuucaucucuugugaaccauagucccuccccaca | manual\_scaffold\_4b:431869567..431869630:- |
| manual\_scaffold\_3a\_9669 | 4.8e+1 | 0.96 ± 0.01 |  | 92 | 74 | 0 | 18 | yes |  |  |  | blast | cuagcacuguacuuaugcuguu | agcaugacagcagugcuuggau | cuagcacuguacuuaugcuguuugguauuaaaaacagcaugacagcagugcuuggau | manual\_scaffold\_3a:599504507..599504564:- |
| manual\_scaffold\_7\_25926 | 4.8e+1 | 0.96 ± 0.01 |  | 93 | 67 | 0 | 26 | yes |  |  |  | blast | auccuggcacugcucauaugcu | acaugagagaagugcuuggauu | auccuggcacugcucauaugcuguguaguauuauaaacaacaugagagaagugcuuggauu | manual\_scaffold\_7:1274135536..1274135597:+ |
| manual\_scaffold\_1a\_1611 | 4.8e+1 | 0.96 ± 0.01 |  | 92 | 88 | 0 | 4 | yes |  |  |  | blast | uccugacacugcuugcuugcug | cauuagagcagcgucuugauu | uccugacacugcuugcuugcuguuuauguuaguaaacagcauuagagcagcgucuugauu | manual\_scaffold\_1a:982807317..982807377:- |
| manual\_scaffold\_12\_38338 | 4.7e+1 | 0.96 ± 0.01 |  | 99 | 95 | 0 | 4 | no |  |  |  | blast | cugucgggugucgcuguugggugc | accaaugcugcucccgaccucc | cugucgggugucgcuguugggugccguucagcacgccguccgcaccaaugcugcucccgaccucc | manual\_scaffold\_12:507951667..507951732:+ |
| manual\_scaffold\_7\_27122 | 4.6e+1 | 0.96 ± 0.01 |  | 90 | 80 | 0 | 10 | yes |  |  |  | blast | agcuuuggaguugucuggugau | cacuugaacucucucaagcucu | cacuugaacucucucaagcucugugauauuucuaucagagcuuuggaguugucuggugau | manual\_scaffold\_7:584508661..584508721:- |
| manual\_scaffold\_3a\_7669 | 4.5e+1 | 0.96 ± 0.01 |  | 86 | 67 | 0 | 19 | yes |  |  |  | blast | uggacaccgccacaccguauuau | auacggugugcggucucguuu | uggacaccgccacaccguauuauguauugcggguuguaauacggugugcggucucguuu | manual\_scaffold\_3a:346799870..346799929:+ |
| manual\_scaffold\_1b\_3503 | 4.3e+1 | 0.96 ± 0.01 |  | 84 | 53 | 0 | 31 | yes |  |  |  | blast | uuucuuugucggacuuauaguc | acuaaguccgacaaagaaauu | uuucuuugucggacuuauagucuuguauuacacaagacuaaguccgacaaagaaauu | manual\_scaffold\_1b:831805783..831805840:- |
| manual\_scaffold\_2b\_6476 | 4.3e+1 | 0.96 ± 0.01 |  | 83 | 76 | 0 | 7 | yes |  |  |  | blast | cgcacucaugaugggaccucc | aggucccugccuugagugugcu | cgcacucaugaugggaccuccgcaauaaguaacugcaauguggaggucccugccuugagugugcu | manual\_scaffold\_2b:720728455..720728520:+ |
| manual\_scaffold\_2a\_3923 | 4.2e+1 | 0.96 ± 0.01 |  | 92 | 31 | 0 | 61 | no |  |  |  | blast | caauucaagguuacaaacauc | augucuggcauccuugacuugac | caauucaagguuacaaacaucagaccuuaaaacagaugucuggcauccuugacuugac | manual\_scaffold\_2a:125025537..125025595:+ |
| manual\_scaffold\_9\_32182 | 4.2e+1 | 0.96 ± 0.01 |  | 83 | 71 | 0 | 12 | yes |  |  |  | blast | cacuuggccauucuguaacucu | aguuuggagcugccaagag | cacuuggccauucuguaacucugugcuauaauagcuaguagaguuuggagcugccaagag | manual\_scaffold\_9:1211751154..1211751214:+ |
| manual\_scaffold\_8\_29309 | 4.2e+1 | 0.96 ± 0.01 |  | 82 | 53 | 0 | 29 | yes |  |  |  | blast | gacuuugguguggccuggaagu | gaccgggccccucuaaaguuc | gaccgggccccucuaaaguucagcaccauaacggcaucugacuuugguguggccuggaagu | manual\_scaffold\_8:1114071163..1114071224:+ |
| manual\_scaffold\_7\_27607 | 4.1e+1 | 0.96 ± 0.01 |  | 80 | 52 | 0 | 28 | yes |  |  |  | blast | ucuuggacaacuuccagugugagg | ucacugagaguugugcaga | ucuuggacaacuuccagugugagguuguacauccucacugagaguugugcaga | manual\_scaffold\_7:1097242566..1097242619:- |
| manual\_scaffold\_1a\_248 | 4.1e+1 | 0.96 ± 0.01 |  | 80 | 75 | 0 | 5 | yes |  |  |  | blast | aucuagacacuguucucaugcu | caugaaaccauugucuggacu | aucuagacacuguucucaugcugcuuaacaucaucagcaugaaaccauugucuggacu | manual\_scaffold\_1a:307498367..307498425:+ |
| manual\_scaffold\_1b\_3523 | 4.1e+1 | 0.96 ± 0.01 |  | 89 | 88 | 0 | 1 | no |  |  |  | blast | uaccugguugauucugcc | guugauccugccaggagc | uaccugguugauucugccaauagggcuaguugguugauccugccaggagc | manual\_scaffold\_1b:845706325..845706375:- |
| manual\_scaffold\_4b\_15894 | 4.0e+1 | 0.96 ± 0.01 |  | 79 | 69 | 0 | 10 | yes |  |  |  | blast | uaucccguccaacucaauac | uaauaagguggacgggauagc | uaucccguccaacucaauacaagcacauuacaccuguaauaagguggacgggauagc | manual\_scaffold\_4b:1091934770..1091934827:+ |
| manual\_scaffold\_6\_22552 | 4.0e+1 | 0.96 ± 0.01 |  | 77 | 73 | 0 | 4 | yes |  |  |  | blast | acgggcugcacagcacaagugc | gcuugugcugugcaguccauaac | acgggcugcacagcacaagugcagucugcgcuugugcugugcaguccauaac | manual\_scaffold\_6:1706015718..1706015770:+ |
| manual\_scaffold\_11\_36087 | 4.0e+1 | 0.96 ± 0.01 |  | 77 | 50 | 0 | 27 | yes |  |  |  | blast | uugccguaccguuaguccucacu | ugaggacuugcgagagggcaaugu | ugaggacuugcgagagggcaauguacucuucauugccguaccguuaguccucacu | manual\_scaffold\_11:272701825..272701880:+ |
| manual\_scaffold\_1b\_3791 | 4.0e+1 | 0.96 ± 0.01 |  | 77 | 75 | 0 | 2 | yes |  |  |  | blast | ugugaaaucugggacuuguagu | uacaaaucccagaauucaaagg | ugugaaaucugggacuuguaguccuggugauggaaaaaaaaauacuacaaaucccagaauucaaagg | manual\_scaffold\_1b:1217741602..1217741669:- |
| manual\_scaffold\_3b\_12047 | 3.9e+1 | 0.96 ± 0.01 |  | 79 | 71 | 0 | 8 | yes |  |  |  | blast | uuccauaugcucacuguaacagu | uuaguggugcagcaucuggaa | uuccauaugcucacuguaacaguuuguuuauauaacuuaguggugcagcaucuggaa | manual\_scaffold\_3b:1010875615..1010875672:- |
| manual\_scaffold\_8\_29725 | 3.9e+1 | 0.96 ± 0.01 |  | 75 | 56 | 0 | 19 | yes |  |  |  | blast | ggggcuaccggaguuuuuguu | caaaaacucuguuagcucugcc | ggggcuaccggaguuuuuguucgaacucugcgcugagugacaaaaacucuguuagcucugcc | manual\_scaffold\_8:60936041..60936103:- |
| manual\_scaffold\_6\_22615 | 3.8e+1 | 0.96 ± 0.01 |  | 74 | 69 | 0 | 5 | yes |  |  |  | blast | uuuaugagcucuggggguacc | uacccccauuggcgcuuuaua | uacccccauuggcgcuuuauaaagggaugacuauuacuuuaugagcucuggggguacc | manual\_scaffold\_6:1748801949..1748802007:+ |
| manual\_scaffold\_6\_22325 | 3.8e+1 | 0.96 ± 0.01 |  | 74 | 41 | 0 | 33 | yes |  |  |  | blast | cacucugucgaauccucacgc | uggcgauccgucagaguuucugu | uggcgauccgucagaguuucugucacaucgcaggcacucugucgaauccucacgc | manual\_scaffold\_6:1519188385..1519188440:+ |
| manual\_scaffold\_12\_37811 | 3.8e+1 | 0.96 ± 0.01 |  | 75 | 51 | 0 | 24 | yes |  |  |  | blast | uacacauccuagaauucaaag | uuugaauucuaggacuuuuagu | uuugaauucuaggacuuuuagucuuauuuauuuauaaaagaauacuacacauccuagaauucaaag | manual\_scaffold\_12:11457607..11457673:+ |
| manual\_scaffold\_7\_26680 | 3.8e+1 | 0.96 ± 0.01 |  | 84 | 67 | 0 | 17 | no |  |  |  | blast | ucucauuucgggccugcacau | agugccucccugaucucagaga | agugccucccugaucucagagauuuguuuaccugaagagucucauuucgggccugcacau | manual\_scaffold\_7:138608158..138608218:- |
| manual\_scaffold\_7\_24983 | 3.7e+1 | 0.96 ± 0.01 |  | 73 | 63 | 0 | 10 | yes |  |  |  | blast | uugcaaucacguuaguccucccu | ugaggacuugugagagggcaaugu | ugaggacuugugagagggcaauguauucuucauugcaaucacguuaguccucccu | manual\_scaffold\_7:288919156..288919211:+ |
| manual\_scaffold\_3b\_10955 | 3.6e+1 | 0.96 ± 0.01 |  | 70 | 55 | 0 | 15 | yes |  |  |  | blast | ucaugagggggcuugcaaugc | uauugcaagcccccucaucgau | uauugcaagcccccucaucgauggcuuccucaacaaucaugagggggcuugcaaugc | manual\_scaffold\_3b:1062913590..1062913647:+ |
| manual\_scaffold\_3b\_10320 | 3.6e+1 | 0.96 ± 0.01 |  | 71 | 60 | 0 | 11 | yes |  |  |  | blast | auggaagcaugugucacguggucu | accaugucaccugcuucucaca | auggaagcaugugucacguggucuuuuguagacgcagaccaugucaccugcuucucaca | manual\_scaffold\_3b:377644377..377644436:+ |
| manual\_scaffold\_4b\_17256 | 3.6e+1 | 0.96 ± 0.01 |  | 72 | 50 | 0 | 22 | yes |  |  |  | blast | cuaacuucacauauauguaccu | uacauagaugucaaguuaguaa | cuaacuucacauauauguaccuacgcaguacguauaucuucaagguacauagaugucaaguuaguaa | manual\_scaffold\_4b:986919180..986919247:- |
| manual\_scaffold\_3a\_7648 | 3.6e+1 | 0.96 ± 0.01 |  | 71 | 57 | 0 | 14 | yes |  |  |  | blast | cgggaccuauugacuuugccaa | ugcaaugccagugggucuacgu | ugcaaugccagugggucuacguugucaacugaaaggcgggaccuauugacuuugccaa | manual\_scaffold\_3a:335872491..335872549:+ |
| manual\_scaffold\_3a\_9944 | 3.5e+1 | 0.96 ± 0.01 |  | 69 | 57 | 0 | 12 | yes |  |  |  | blast | guguggcagaaggaacagguac | uagcugaucaucugccacaaucc | guguggcagaaggaacagguacuugaguugacaggaccgaacaucaguagcugaucaucugccacaaucc | manual\_scaffold\_3a:930077313..930077383:- |
| manual\_scaffold\_6\_22290 | 3.5e+1 | 0.96 ± 0.01 |  | 69 | 66 | 0 | 3 | yes |  |  |  | blast | uuggaacccuaugauaagcagu | uugcuuaucauugguuuucauuc | uuggaacccuaugauaagcagugugucagcccauugcuuaucauugguuuucauuc | manual\_scaffold\_6:1475739008..1475739064:+ |
| manual\_scaffold\_3a\_7564 | 3.5e+1 | 0.96 ± 0.01 |  | 69 | 68 | 0 | 1 | yes |  |  |  | blast | ucguugauauuucgcuccgacu | ucgggauggauauuggugaag | ucguugauauuucgcuccgacuauaauacagggucgggauggauauuggugaag | manual\_scaffold\_3a:296824356..296824410:+ |
| manual\_scaffold\_3b\_11149 | 3.5e+1 | 0.96 ± 0.01 |  | 69 | 60 | 0 | 9 | yes |  |  |  | blast | auuugugacagauaagucuugcgc | ccaggacucaucuguugcuggga | auuugugacagauaagucuugcgcauccugcaugaaauugccaggacucaucuguugcuggga | manual\_scaffold\_3b:100842623..100842686:- |
| manual\_scaffold\_11\_37621 | 3.5e+1 | 0.96 ± 0.01 |  | 70 | 69 | 0 | 1 | yes |  |  |  | blast | uuuccaaaaugaguagaucagc | cugacuuacucuauuuuugaga | uuuccaaaaugaguagaucagcaccauuccuacuacgcaugcugacuuacucuauuuuugaga | manual\_scaffold\_11:933377660..933377723:- |
| manual\_scaffold\_3b\_11148 | 3.4e+1 | 0.96 ± 0.01 |  | 65 | 38 | 0 | 27 | yes |  |  |  | blast | cguaucauacaggauaacucu | gaguuauccuguaugauauggc | cguaucauacaggauaacucuguggugcuucggaguuauccuguaugauauggc | manual\_scaffold\_3b:98675691..98675745:- |
| manual\_scaffold\_3a\_9561 | 3.4e+1 | 0.96 ± 0.01 |  | 66 | 45 | 0 | 21 | yes |  |  |  | blast | cuggugcuuugcucaugcugcu | cagcaugagagcagcaccaaga | cuggugcuuugcucaugcugcuuaacauuaucagcagcaugagagcagcaccaaga | manual\_scaffold\_3a:536967559..536967615:- |
| manual\_scaffold\_1b\_1885 | 3.4e+1 | 0.96 ± 0.01 |  | 67 | 49 | 0 | 18 | yes |  |  |  | blast | ucagaacuacaaugggccuuau | uggcccauugucauucugucug | uggcccauugucauucugucugaagcuaauugucacauucagaacuacaaugggccuuau | manual\_scaffold\_1b:96386039..96386099:+ |
| manual\_scaffold\_2a\_4107 | 3.4e+1 | 0.96 ± 0.01 |  | 66 | 60 | 0 | 6 | yes |  |  |  | blast | cagggaauucugcaacccaagu | ugagguuacagaagguccuug | ugagguuacagaagguccuugugauagcguuguuuugccagggaauucugcaacccaagu | manual\_scaffold\_2a:359908659..359908719:+ |
| manual\_scaffold\_9\_31297 | 3.3e+1 | 0.96 ± 0.01 |  | 64 | 49 | 0 | 15 | yes |  |  |  | blast | caccagcugaaagauaaaauga | auuguaucuuucagcuccuggc | caccagcugaaagauaaaaugaauauuaaauaucauuguaucuuucagcuccuggc | manual\_scaffold\_9:437007591..437007647:+ |
| manual\_scaffold\_3a\_9676 | 3.3e+1 | 0.96 ± 0.01 |  | 64 | 51 | 0 | 13 | yes |  |  |  | blast | gaaguacuccuggcuuacuccu | aguacaccaggauuacucgau | gaaguacuccuggcuuacuccugaauucaggaguacaccaggauuacucgau | manual\_scaffold\_3a:604458975..604459027:- |
| manual\_scaffold\_1b\_2776 | 3.2e+1 | 0.96 ± 0.01 |  | 66 | 42 | 0 | 24 | yes |  |  |  | blast | agccccacuuagagcucuuugcu | caagagcucaaaauggugcaucu | agccccacuuagagcucuuugcugcaaaauuaauggcuucagcaagagcucaaaauggugcaucu | manual\_scaffold\_1b:1140990764..1140990829:+ |
| manual\_scaffold\_12\_39217 | 3.2e+1 | 0.96 ± 0.01 |  | 72 | 71 | 0 | 1 | no |  |  |  | blast | cacuuggccauucuguaacucu | aauuuggagcugccaagaggu | cacuuggccauucuguaacucugugcuaugauagcuaguagaauuuggagcugccaagaggu | manual\_scaffold\_12:252871196..252871258:- |
| manual\_scaffold\_6\_23465 | 3.2e+1 | 0.96 ± 0.01 |  | 70 | 68 | 0 | 2 | no |  |  |  | blast | ccucagacucuuuucggaggcu | ccucguucccggucugugcca | ccucagacucuuuucggaggcugucuuuccagccucguucccggucugugcca | manual\_scaffold\_6:467285252..467285305:- |
| manual\_scaffold\_5\_17879 | 3.2e+1 | 0.96 ± 0.01 |  | 69 | 58 | 0 | 11 | no |  |  |  | blast | gcugcauccucaauuugguug | caccaggguggggauugcauagccu | caccaggguggggauugcauagccugccauuuuauuucgaggcugcauccucaauuugguug | manual\_scaffold\_5:293639747..293639809:+ |
| manual\_scaffold\_3a\_9777 | 3.1e+1 | 0.96 ± 0.01 |  | 60 | 38 | 0 | 22 | yes |  |  |  | blast | uagcggaugcauaguucagaua | ucugagcacacauccauugga | uagcggaugcauaguucagauaaucaguuaaagucugagcacacauccauugga | manual\_scaffold\_3a:739038741..739038795:- |
| manual\_scaffold\_12\_39463 | 3.0e+1 | 0.96 ± 0.01 |  | 58 | 55 | 0 | 3 | yes |  |  |  | blast | caccagcugaaagauagaauga | uuuuaucuuucagcuccuggcu | caccagcugaaagauagaaugauaaagaaauaucguuuuaucuuucagcuccuggcu | manual\_scaffold\_12:552065438..552065495:- |
| manual\_scaffold\_9\_32946 | 3.0e+1 | 0.96 ± 0.01 |  | 57 | 41 | 0 | 16 | yes |  |  |  | blast | uuugaauuaugggauuuguagucu | cuaugucucagaauucaaaga | uuugaauuaugggauuuguagucuuguuuauuccacuauauaauacgacuaugucucagaauucaaaga | manual\_scaffold\_9:868409051..868409120:- |
| manual\_scaffold\_4b\_14822 | 2.9e+1 | 0.96 ± 0.01 |  | 57 | 56 | 0 | 1 | yes |  |  |  | blast | acugagcaugucagauugucu | ggccagccugacaugcacagu | acugagcaugucagauugucuggaauuuucaccguggccagccugacaugcacagu | manual\_scaffold\_4b:25489113..25489169:+ |
| manual\_scaffold\_5\_18965 | 2.9e+1 | 0.96 ± 0.01 |  | 58 | 57 | 0 | 1 | yes |  |  |  | blast | agcaagagcagugcuuggacu | auccuagcacuacuuuuauucugu | auccuagcacuacuuuuauucuguguagcacuguaagcagcaagagcagugcuuggacu | manual\_scaffold\_5:1777008525..1777008584:+ |
| manual\_scaffold\_5\_20189 | 2.9e+1 | 0.96 ± 0.01 |  | 58 | 51 | 0 | 7 | yes |  |  |  | blast | uacuuggcaaaaugaacaauau | uuguucauuuagccaacuacu | uacuuggcaaaaugaacaauauggguguaaaagucuauuguucauuuagccaacuacu | manual\_scaffold\_5:1311831163..1311831221:- |
| manual\_scaffold\_2b\_6787 | 2.9e+1 | 0.96 ± 0.01 |  | 56 | 55 | 0 | 1 | yes |  |  |  | blast | uuuccgagcugcgcugucugc | agauagaacugcuaggaaacag | agauagaacugcuaggaaacagagacaaucacuacuguuuccgagcugcgcugucugc | manual\_scaffold\_2b:328782287..328782345:- |
| manual\_scaffold\_4a\_13222 | 2.9e+1 | 0.96 ± 0.01 |  | 57 | 17 | 0 | 40 | yes |  |  |  | blast | ucccgagcucacucguucucaga | ugagaaccgaaggaacuuggaga | ugagaaccgaaggaacuuggagaguggccaauucucccgagcucacucguucucaga | manual\_scaffold\_4a:647158259..647158316:+ |
| manual\_scaffold\_12\_38066 | 2.9e+1 | 0.96 ± 0.01 |  | 57 | 17 | 0 | 40 | yes |  |  |  | blast | ucccgagcucacucguucucaga | ugagaaccgaaggaacuuggaga | ugagaaccgaaggaacuuggagaguggcuaauucucccgagcucacucguucucaga | manual\_scaffold\_12:122227001..122227058:+ |
| manual\_scaffold\_1a\_326 | 2.9e+1 | 0.96 ± 0.01 |  | 59 | 58 | 0 | 1 | yes |  |  |  | blast | uagcaagauugaucuaacaccu | gaauuagauccuucuucuaga | uagcaagauugaucuaacaccuguauaucaccaaauggaauuagauccuucuucuaga | manual\_scaffold\_1a:390899476..390899534:+ |
| manual\_scaffold\_11\_35901 | 2.8e+1 | 0.96 ± 0.01 |  | 53 | 38 | 0 | 15 | yes |  |  |  | blast | uuugaauugugggacuuguaguc | cacaaguccuagaauucaaaga | uuugaauugugggacuuguagucuucuuuuauaauguauuaagcaagaccacaaguccuagaauucaaaga | manual\_scaffold\_11:42435410..42435481:+ |
| manual\_scaffold\_9\_32500 | 2.7e+1 | 0.96 ± 0.01 |  | 53 | 52 | 0 | 1 | yes |  |  |  | blast | uguggaauuccagagucgaacu | cuccgccucugcugaauuccaca | cuccgccucugcugaauuccacauagguuuuuaucaacucuguggaauuccagagucgaacu | manual\_scaffold\_9:330347955..330348017:- |
| manual\_scaffold\_12\_37956 | 2.7e+1 | 0.96 ± 0.01 |  | 53 | 46 | 0 | 7 | yes |  |  |  | blast | caggagguauuaagaagucacga | cugcuuguucguaccuccuaua | caggagguauuaagaagucacgauuaucaguuauuucugcuuguucguaccuccuaua | manual\_scaffold\_12:76969383..76969441:+ |
| manual\_scaffold\_1b\_3435 | 2.7e+1 | 0.96 ± 0.01 |  | 52 | 48 | 0 | 4 | yes |  |  |  | blast | uucgguuaucuagcuuuaugac | auacagcuagauaaccaaagau | uucgguuaucuagcuuuaugacgaucaguacacucauacagcuagauaaccaaagau | manual\_scaffold\_1b:740579896..740579953:- |
| manual\_scaffold\_8\_30353 | 2.7e+1 | 0.96 ± 0.01 |  | 53 | 49 | 0 | 4 | yes |  |  |  | blast | ugaugggcggauaauaaaacaga | uguuuuuuguccacucuaaga | ugaugggcggauaauaaaacagaucuggguaccugauguguuuuuuguccacucuaaga | manual\_scaffold\_8:896985918..896985977:- |
| manual\_scaffold\_2b\_6146 | 2.7e+1 | 0.96 ± 0.01 |  | 53 | 52 | 0 | 1 | yes |  |  |  | blast | uuuguacauuugaagcgugugcc | cacaugauucauuuguacuuc | uuuguacauuugaagcgugugccuguuucccagggucagcacaugauucauuuguacuuc | manual\_scaffold\_2b:312529992..312530052:+ |
| manual\_scaffold\_8\_29840 | 2.7e+1 | 0.96 ± 0.01 |  | 53 | 51 | 0 | 2 | yes |  |  |  | blast | ugguacagcgagcauucugauu | ucagcaugcacucuguacaca | ucagcaugcacucuguacacaagacauccuuuuugguacagcgagcauucugauu | manual\_scaffold\_8:153762130..153762185:- |
| manual\_scaffold\_2a\_5738 | 2.7e+1 | 0.96 ± 0.01 |  | 51 | 48 | 0 | 3 | yes |  |  |  | blast | ggcaaaagugauaugugcacuu | ugugcaugucacuuuggcugga | ugugcaugucacuuuggcuggaugucuugcgcuggcaaaagugauaugugcacuu | manual\_scaffold\_2a:1213886670..1213886725:- |
| manual\_scaffold\_5\_18979 | 2.6e+1 | 0.96 ± 0.01 |  | 51 | 33 | 0 | 18 | yes |  |  |  | blast | acugagauucauggccauuaau | uuaaaggcccucuaccucauu | uuaaaggcccucuaccucauuauuauuauaggcuauaacugagauucauggccauuaau | manual\_scaffold\_5:1790866093..1790866152:+ |
| manual\_scaffold\_8\_29719 | 2.6e+1 | 0.96 ± 0.01 |  | 49 | 41 | 0 | 8 | yes |  |  |  | blast | cacccggcggacgaccucucgc | ugagaggucguccgccagguggg | cacccggcggacgaccucucgcuaauggugguaucagugagaggucguccgccagguggg | manual\_scaffold\_8:60696877..60696937:- |
| manual\_scaffold\_3b\_12098 | 2.6e+1 | 0.96 ± 0.01 |  | 51 | 50 | 0 | 1 | yes |  |  |  | blast | uuagaucugaguaaaggauagc | guauccuuacucagaaguaacu | guauccuuacucagaaguaacuuggguucucauaguuagaucugaguaaaggauagc | manual\_scaffold\_3b:1070270875..1070270932:- |
| manual\_scaffold\_10\_35582 | 2.6e+1 | 0.96 ± 0.01 |  | 50 | 25 | 0 | 25 | yes |  |  |  | blast | caccaaagcacugccugcacug | cgugcaguuagacuuuggugg | cgugcaguuagacuuugguggugauacauacagaaucccaccaaagcacugccugcacug | manual\_scaffold\_10:924430566..924430626:- |
| manual\_scaffold\_4b\_15271 | 2.6e+1 | 0.96 ± 0.01 |  | 50 | 23 | 0 | 27 | yes |  |  |  | blast | ucguggucacgcuaguccucacc | ugaggacuugcgagagggcaaugu | ugaggacuugcgagagggcaauguacucuucaucguggucacgcuaguccucacc | manual\_scaffold\_4b:422930261..422930316:+ |
| manual\_scaffold\_9\_32061 | 2.5e+1 | 0.96 ± 0.01 |  | 58 | 54 | 0 | 4 | no |  |  |  | blast | ugugagaucuauggaugccuggcc | acacgcauccagucuuaccuu | acacgcauccagucuuaccuugugaugaauaagaaaaugugagaucuauggaugccuggcc | manual\_scaffold\_9:1128589148..1128589209:+ |
| manual\_scaffold\_2b\_6481 | 2.5e+1 | 0.96 ± 0.01 |  | 51 | 31 | 0 | 20 | yes |  |  |  | blast | aacccagacugguuucugaagu | uccggagaucagccugagaagg | uccggagaucagccugagaaggauaauuguaaaauaaccaacccagacugguuucugaagu | manual\_scaffold\_2b:721641671..721641732:+ |
| manual\_scaffold\_9\_33204 | 2.5e+1 | 0.96 ± 0.01 |  | 49 | 43 | 0 | 6 | yes |  |  |  | blast | uggguuggugcugccuggaggu | agccaggccacuccaacuaacagu | agccaggccacuccaacuaacaguguuauuuuaaaaucacuggguuggugcugccuggaggu | manual\_scaffold\_9:1145279416..1145279478:- |
| manual\_scaffold\_5\_20341 | 2.5e+1 | 0.96 ± 0.01 |  | 47 | 34 | 0 | 13 | yes |  |  |  | blast | ucucugccucaaccacccaugu | augugugguuaaggcagagaa | ucucugccucaaccacccaugugaauaacaacgcacaugugugguuaaggcagagaa | manual\_scaffold\_5:1536540594..1536540651:- |
| manual\_scaffold\_10\_34386 | 2.5e+1 | 0.96 ± 0.01 |  | 47 | 44 | 0 | 3 | yes |  |  |  | blast | augauauugucaaugcuucagu | ugauaccguugacgauguuacc | augauauugucaaugcuucagucgacgaugacacuauugcugauaccguugacgauguuacc | manual\_scaffold\_10:1083885617..1083885679:+ |
| manual\_scaffold\_4a\_13181 | 2.4e+1 | 0.96 ± 0.01 |  | 53 | 44 | 0 | 9 | yes |  |  |  | blast | accacuacuuucuucggauagu | uauugccgaaaaaggauauguggu | uauugccgaaaaaggauauguggugaaaagcuaaacacaccacuacuuucuucggauagu | manual\_scaffold\_4a:606992794..606992854:+ |
| manual\_scaffold\_5\_18730 | 2.4e+1 | 0.96 ± 0.01 |  | 47 | 45 | 0 | 2 | yes |  |  |  | blast | cuggcugugcucuccugcugcu | cagcaugagagcaguguuagga | cuggcugugcucuccugcugcuuaacauuaucaacagcaugagagcaguguuagga | manual\_scaffold\_5:1468468462..1468468518:+ |
| manual\_scaffold\_7\_27784 | 2.4e+1 | 0.96 ± 0.01 |  | 49 | 48 | 0 | 1 | yes |  |  |  | blast | ucccuuccagguacuguacua | guucaguauuccgauggggu | guucaguauuccgaugggguguguuuaggguaucccuuccagguacuguacua | manual\_scaffold\_7:1224299176..1224299229:- |
| manual\_scaffold\_5\_18176 | 2.4e+1 | 0.96 ± 0.01 |  | 46 | 28 | 0 | 18 | yes |  |  |  | blast | cggccaaaccaacaugcucacu | ugagcaugccgguuuggcccgc | ugagcaugccgguuuggcccgcuguccuaggacggccaaaccaacaugcucacu | manual\_scaffold\_5:708215527..708215581:+ |
| manual\_scaffold\_3b\_10401 | 2.4e+1 | 0.96 ± 0.01 |  | 46 | 36 | 0 | 10 | yes |  |  |  | blast | caccaauccugaugcugcuuuc | aaagcagcgucagaauuuguu | caccaauccugaugcugcuuucaugcuuguuaccagcaugaaagcagcgucagaauuuguu | manual\_scaffold\_3b:459459021..459459082:+ |
| manual\_scaffold\_6\_23470 | 2.4e+1 | 0.96 ± 0.01 |  | 47 | 46 | 0 | 1 | yes |  |  |  | blast | acgagauacucggcuggcaaga | agccagucugguaggcucgac | agccagucugguaggcucgaccuaaacacacgaguggacgagauacucggcuggcaaga | manual\_scaffold\_6:467732536..467732595:- |
| manual\_scaffold\_10\_34258 | 2.4e+1 | 0.96 ± 0.01 |  | 46 | 34 | 0 | 12 | yes |  |  |  | blast | acacacauauguauuacacuuc | gugcuaauacauguguguguau | gugcuaauacauguguguguauguacacauagcaaaucugaaaauacacacauauguauuacacuuc | manual\_scaffold\_10:951463566..951463633:+ |
| manual\_scaffold\_11\_36444 | 2.4e+1 | 0.96 ± 0.01 |  | 45 | 15 | 0 | 30 | yes |  |  |  | blast | ucugcuaguguucugaauaacu | uuguucugagacacuggcagagc | ucugcuaguguucugaauaacuguuagagucaguuguucugagacacuggcagagc | manual\_scaffold\_11:739849134..739849190:+ |
| manual\_scaffold\_3a\_7403 | 2.3e+1 | 0.96 ± 0.01 |  | 45 | 41 | 0 | 4 | yes |  |  |  | blast | cuggcauccaggguugacucc | agucagcccuaggcacuugaaa | agucagcccuaggcacuugaaagacuuaaacuucuggcauccaggguugacucc | manual\_scaffold\_3a:183442394..183442448:+ |
| manual\_scaffold\_9\_32941 | 2.3e+1 | 0.96 ± 0.01 |  | 47 | 43 | 0 | 4 | yes |  |  |  | blast | cuguaguucauguaauuguuu | aacaauuacacaaacuacgcuu | cuguaguucauguaauuguuuaaagaauuuuucacaaacaauuacacaaacuacgcuu | manual\_scaffold\_9:856542210..856542268:- |
| manual\_scaffold\_1b\_3528 | 2.3e+1 | 0.96 ± 0.01 |  | 46 | 34 | 0 | 12 | yes |  |  |  | blast | auggcauaaguggaccaguauc | auuggcacacuugcaccaguaa | auuggcacacuugcaccaguaacuuguacuuggacauuaauggcauaaguggaccaguauc | manual\_scaffold\_1b:848945117..848945178:- |
| manual\_scaffold\_6\_22795 | 2.3e+1 | 0.96 ± 0.01 |  | 44 | 42 | 0 | 2 | yes |  |  |  | blast | uggcgcuguucucauuaugcu | cagcaugagagcagagucugga | uggcgcuguucucauuaugcuuaacauuaucagcagcaugagagcagagucugga | manual\_scaffold\_6:103997834..103997889:- |
| manual\_scaffold\_1b\_3106 | 2.3e+1 | 0.96 ± 0.01 |  | 44 | 38 | 0 | 6 | yes |  |  |  | blast | uaucuguaaaccacucgggucu | cccugaguggaacagaacuccu | cccugaguggaacagaacuccuugaaacacauuagagguaucuguaaaccacucgggucu | manual\_scaffold\_1b:280315699..280315759:- |
| manual\_scaffold\_4a\_12294 | 2.2e+1 | 0.96 ± 0.01 |  | 42 | 10 | 0 | 32 | yes |  |  |  | blast | uaugaauucuaggauuuguaguc | uacaagucccagaauucaaa | uaugaauucuaggauuuguagucuuguuuaucguguuauaaaacaggacuacaagucccagaauucaaa | manual\_scaffold\_4a:26890373..26890442:+ |
| manual\_scaffold\_1a\_34 | 2.2e+1 | 0.96 ± 0.01 |  | 41 | 19 | 0 | 22 | yes |  |  |  | blast | ucuacauccauccucccgggc | cccgggaggauggauguagacg | cccgggaggauggauguagacgcagggggcgccugcgucuacauccauccucccgggc | manual\_scaffold\_1a:53850491..53850549:+ |
| manual\_scaffold\_12\_37986 | 2.2e+1 | 0.96 ± 0.01 |  | 42 | 25 | 0 | 17 | yes |  |  |  | blast | uucgaguugcaauuugagagu | cucgcaaauugcaacucgcaau | uucgaguugcaauuugagaguccgagcgacucgcaaauugcaacucgcaau | manual\_scaffold\_12:85173462..85173513:+ |
| manual\_scaffold\_12\_38221 | 2.2e+1 | 0.96 ± 0.01 |  | 42 | 41 | 0 | 1 | yes |  |  |  | blast | uggccaaggugacaugcacacu | ugcacaugucacuuuggccg | ugcacaugucacuuuggccgacuguuuugcacuggccaaggugacaugcacacu | manual\_scaffold\_12:342940402..342940456:+ |
| manual\_scaffold\_9\_32476 | 2.2e+1 | 0.96 ± 0.01 |  | 43 | 39 | 0 | 4 | yes |  |  |  | blast | cugauguccagguucuugauc | ccauuaaucugggcaucaagga | ccauuaaucugggcaucaaggaaaguuuauggucaaaccugauguccagguucuugauc | manual\_scaffold\_9:300427544..300427603:- |
| manual\_scaffold\_6\_21975 | 2.2e+1 | 0.96 ± 0.01 |  | 45 | 37 | 0 | 8 | yes |  |  |  | blast | ugccuggccucuccaacuaucagu | uguguuggugcugccuggaggu | ugccuggccucuccaacuaucaguguuauuuaaaaccacuguguuggugcugccuggaggu | manual\_scaffold\_6:1190538241..1190538302:+ |
| manual\_scaffold\_6\_21589 | 2.2e+1 | 0.96 ± 0.01 |  | 44 | 43 | 0 | 1 | yes |  |  |  | blast | aaggccggcauugauuucuuaa | acagaaaucaaagccauggcu | acagaaaucaaagccauggcuauugacguugccaguucuaaaagugucaaggccggcauugauuucuuaa | manual\_scaffold\_6:784370160..784370230:+ |
| manual\_scaffold\_5\_17996 | 2.2e+1 | 0.96 ± 0.01 |  | 45 | 21 | 0 | 24 | yes |  |  |  | blast | uguucugcuuggaucuuuuucu | uaaacagguccuuaggcagaagu | uguucugcuuggaucuuuuucugugagaaaaaggagcauaaacagguccuuaggcagaagu | manual\_scaffold\_5:470064182..470064243:+ |
| manual\_scaffold\_4a\_13206 | 2.2e+1 | 0.96 ± 0.01 |  | 42 | 40 | 0 | 2 | yes |  |  |  | blast | uguccccauuccagaguuacu | uaacucuggaugugggggguauc | uguccccauuccagaguuacuagcacacccuguaucuguaacucuggaugugggggguauc | manual\_scaffold\_4a:632081534..632081595:+ |
| manual\_scaffold\_9\_33033 | 2.2e+1 | 0.96 ± 0.01 |  | 41 | 40 | 0 | 1 | yes |  |  |  | blast | aaauagaacuuuauugguauc | auaccaauaaagcucuauugu | aaauagaacuuuauugguauccuuuauccguuggcugggcuggauaccaauaaagcucuauugu | manual\_scaffold\_9:972569521..972569585:- |
| manual\_scaffold\_3a\_8312 | 2.1e+1 | 0.96 ± 0.01 |  | 42 | 13 | 0 | 29 | yes |  |  |  | blast | cacuuggcugcucuaaaacucugc | agaguuggagcugccaggaggu | cacuuggcugcucuaaaacucugcacuguuauagcugguagaguuggagcugccaggaggu | manual\_scaffold\_3a:571424306..571424367:+ |
| manual\_scaffold\_4b\_15763 | 2.1e+1 | 0.96 ± 0.01 |  | 48 | 45 | 0 | 3 | no |  |  |  | blast | ccuaggcuguugaugcccgccu | ugcgggcaguggcagccggucu | ugcgggcaguggcagccggucucggggagcccagccuaggcuguugaugcccgccu | manual\_scaffold\_4b:954793529..954793585:+ |
| manual\_scaffold\_5\_19729 | 2.1e+1 | 0.96 ± 0.01 |  | 42 | 41 | 0 | 1 | yes |  |  |  | blast | gaggugaauccuagauucugga | caguuuccagcggcuuaccuac | gaggugaauccuagauucuggauugaaugcauccugcagagcacuccaguuuccagcggcuuaccuac | manual\_scaffold\_5:744299830..744299898:- |
| manual\_scaffold\_6\_21569 | 2.1e+1 | 0.96 ± 0.01 |  | 42 | 33 | 0 | 9 | yes |  |  |  | blast | ccguagugcacugauuuccaccc | uggaugucucugccucugugcc | uggaugucucugccucugugcccaacugugguggccguagugcacugauuuccaccc | manual\_scaffold\_6:757629668..757629725:+ |
| manual\_scaffold\_6\_21565 | 2.1e+1 | 0.96 ± 0.01 |  | 42 | 33 | 0 | 9 | yes |  |  |  | blast | ccguagugcacugauuuccaccc | uggaugucucugccucugugcc | uggaugucucugccucugugcccaacugugguggccguagugcacugauuuccaccc | manual\_scaffold\_6:757374850..757374907:+ |
| manual\_scaffold\_8\_30790 | 2.1e+1 | 0.96 ± 0.01 |  | 42 | 40 | 0 | 2 | yes |  |  |  | blast | ucagacuacaacgcccagaau | uugggugucuuagucuaaauau | uugggugucuuagucuaaauauuucacagaaaguaaauucagacuacaacgcccagaau | manual\_scaffold\_8:1493739342..1493739401:- |
| manual\_scaffold\_4a\_14552 | 2.1e+1 | 0.96 ± 0.01 |  | 39 | 37 | 0 | 2 | yes |  |  |  | blast | auggcagcagcauucggauugga | aaucugaaugcugcugucaagc | aaucugaaugcugcugucaagcugcuggcagcauggcagcagcauucggauugga | manual\_scaffold\_4a:658126430..658126485:- |
| manual\_scaffold\_8\_28792 | 2.1e+1 | 0.96 ± 0.01 |  | 41 | 23 | 0 | 18 | yes |  |  |  | blast | cuggccuuuuggcuuugccaau | ugguaaaguugauaggucuugc | ugguaaaguugauaggucuugcuuuuaaaaagugaaugcuggccuuuuggcuuugccaau | manual\_scaffold\_8:290503887..290503947:+ |
| manual\_scaffold\_1b\_3440 | 2.1e+1 | 0.96 ± 0.01 |  | 41 | 34 | 0 | 7 | yes |  |  |  | blast | uucuccgaucgaacaccaagg | ugggugcucguuuuuggauaac | ugggugcucguuuuuggauaacaugacuacuuguucuccgaucgaacaccaagg | manual\_scaffold\_1b:741614573..741614627:- |
| manual\_scaffold\_9\_32541 | 2.1e+1 | 0.96 ± 0.01 |  | 42 | 30 | 0 | 12 | yes |  |  |  | blast | accaagcacugcuuuaaugcug | ccauaaaagcauugucaggacu | accaagcacugcuuuaaugcugcuuuuaauaguaaacaccauaaaagcauugucaggacu | manual\_scaffold\_9:389348491..389348551:- |
| manual\_scaffold\_12\_38235 | 2.1e+1 | 0.96 ± 0.01 |  | 41 | 31 | 0 | 10 | yes |  |  |  | blast | gaagaugcgcagauuguaauaug | cauuacaucauaugcaucugccu | cauuacaucauaugcaucugccugugugcucuguaaauaggaagaugcgcagauuguaauaug | manual\_scaffold\_12:362868635..362868698:+ |
| manual\_scaffold\_1a\_946 | 2.1e+1 | 0.96 ± 0.01 |  | 38 | 16 | 0 | 22 | yes |  |  |  | blast | ucuacauccauccucccgggc | cccgggaggauggauguagacg | cccgggaggauggauguagacgcaggcgcccccugcgucuacauccauccucccgggc | manual\_scaffold\_1a:53850490..53850548:- |
| manual\_scaffold\_10\_34306 | 2.1e+1 | 0.96 ± 0.01 |  | 39 | 12 | 0 | 27 | yes |  |  |  | blast | cuggcucuacucucaugcugcu | cagcaugagagcagugucugga | cuggcucuacucucaugcugcuuaacauuauuagcagcaugagagcagugucugga | manual\_scaffold\_10:1020059383..1020059439:+ |
| manual\_scaffold\_6\_21054 | 2.1e+1 | 0.96 ± 0.01 |  | 40 | 38 | 0 | 2 | yes |  |  |  | blast | uuugaauuguaugacuuguagu | uacaagucacagaauucaaauu | uuugaauuguaugacuuguagucguuuuuauuccauuaaaaccaucaggacuacaagucacagaauucaaauu | manual\_scaffold\_6:283393589..283393662:+ |
| manual\_scaffold\_4a\_14628 | 2.0e+1 | 0.96 ± 0.01 |  | 38 | 37 | 0 | 1 | yes |  |  |  | blast | cacagggacagaggaagag | ucuuccucugucccugagc | ucuuccucugucccugagcagcaagugcacagggacagaggaagag | manual\_scaffold\_4a:733426236..733426282:- |
| manual\_scaffold\_3b\_11359 | 2.0e+1 | 0.96 ± 0.01 |  | 38 | 35 | 0 | 3 | yes |  |  |  | blast | uagacgaggcucucaugcugcu | cagcaugagagcagcaucuaga | uagacgaggcucucaugcugcuuaauauuaucagcagcaugagagcagcaucuaga | manual\_scaffold\_3b:358229884..358229940:- |
| manual\_scaffold\_3a\_7456 | 2.0e+1 | 0.96 ± 0.01 |  | 39 | 24 | 0 | 15 | yes |  |  |  | blast | augugcucuuacauuccuuugu | aagggauuuuggaguacauaau | aagggauuuuggaguacauaauguaguauuguaguauucuuuaugugcucuuacauuccuuugu | manual\_scaffold\_3a:216647449..216647513:+ |
| manual\_scaffold\_3a\_7265 | 2.0e+1 | 0.96 ± 0.01 |  | 39 | 33 | 1 | 5 | yes |  |  |  | blast | ucuguucuccaacuccuuuauc | uuggaggaguguuggaggcag | uuggaggaguguuggaggcaggagaggacugagauauaucaccuucuguucuccaacuccuuuauc | manual\_scaffold\_3a:45780116..45780182:+ |
| manual\_scaffold\_4b\_16612 | 2.0e+1 | 0.96 ± 0.01 |  | 38 | 37 | 0 | 1 | yes |  |  |  | blast | cuaagguggacaggauaucugu | gauuuccuguccaccuuauu | gauuuccuguccaccuuauuacaaaugccacaggauuuacuaagguggacaggauaucugu | manual\_scaffold\_4b:158128266..158128327:- |
| manual\_scaffold\_12\_39049 | 2.0e+1 | 0.96 ± 0.01 |  | 38 | 37 | 0 | 1 | yes |  |  |  | blast | uuccgugacuauauagaggaa | uccuauauuaggucaugaagc | uuccgugacuauauagaggaacgaaacagaagguuguguuccuauauuaggucaugaagc | manual\_scaffold\_12:80363980..80364040:- |
| manual\_scaffold\_7\_24873 | 2.0e+1 | 0.96 ± 0.01 |  | 38 | 37 | 0 | 1 | yes |  |  |  | blast | cacaagagcgacauuaggauu | uccaaaugcugcucucaugc | uccaaaugcugcucucaugccauuuauuuacugaaauagcacaagagcgacauuaggauu | manual\_scaffold\_7:154286608..154286668:+ |
| manual\_scaffold\_4b\_15227 | 2.0e+1 | 0.96 ± 0.01 |  | 39 | 37 | 0 | 2 | yes |  |  |  | blast | cauuguaaacuaucuacccu | ugguagacaguuugcaaugc | ugguagacaguuugcaaugcuguauacauuucucaaugagcauuguaaacuaucuacccu | manual\_scaffold\_4b:384948210..384948270:+ |
| manual\_scaffold\_9\_32451 | 1.9e+1 | 0.96 ± 0.01 |  | 38 | 34 | 0 | 4 | yes |  |  |  | blast | aaaguauccuuaucuguaaucu | gaacacaggaaggauacuugc | aaaguauccuuaucuguaaucuguguuugucuccagaacacaggaaggauacuugc | manual\_scaffold\_9:261328795..261328851:- |
| manual\_scaffold\_5\_18964 | 1.9e+1 | 0.96 ± 0.01 |  | 37 | 35 | 0 | 2 | yes |  |  |  | blast | gcacgcaaaucgaccuaccuc | guagguugcaauuugugagc | gcacgcaaaucgaccuaccucauuaauauucauaagguagguugcaauuugugagc | manual\_scaffold\_5:1776368015..1776368071:+ |
| manual\_scaffold\_3b\_11965 | 1.9e+1 | 0.96 ± 0.01 |  | 35 | 34 | 0 | 1 | yes |  |  |  | blast | agccccguugcgcauucuacug | guggaaugcgcaacagggucugc | agccccguugcgcauucuacugcccgaauuacggacaguggaaugcgcaacagggucugc | manual\_scaffold\_3b:924808198..924808258:- |
| manual\_scaffold\_12\_39183 | 1.8e+1 | 0.96 ± 0.01 |  | 35 | 34 | 0 | 1 | yes |  |  |  | blast | auugugauguuauaguuaggcu | gccuaacuguaacaucacaau | auugugauguuauaguuaggcuuguguuucuccuggcagccuaacuguaacaucacaau | manual\_scaffold\_12:203233376..203233435:- |
| manual\_scaffold\_10\_34829 | 1.8e+1 | 0.96 ± 0.01 |  | 35 | 34 | 0 | 1 | yes |  |  |  | blast | ucuggauguuggcagaccgagg | uuggucuguaucuuccucaga | ucuggauguuggcagaccgaggugugccauucccuuggucuguaucuuccucaga | manual\_scaffold\_10:203074805..203074860:- |
| manual\_scaffold\_11\_36721 | 1.8e+1 | 0.96 ± 0.01 |  | 34 | 17 | 0 | 17 | yes |  |  |  | blast | aauucaagcacugcucucaugc | uaagagugguucuaggauuggc | aauucaagcacugcucucaugcgucuucuuaugauaaacagcauaagagugguucuaggauuggc | manual\_scaffold\_11:1041112005..1041112070:+ |
| manual\_scaffold\_3a\_8892 | 1.8e+1 | 0.96 ± 0.01 |  | 36 | 34 | 0 | 2 | yes |  |  |  | blast | gaacuguacccuguuggcugcc | uggcuaaaaggacacagcauu | uggcuaaaaggacacagcauuagcaaauggaccugaacuguacccuguuggcugcc | manual\_scaffold\_3a:185918925..185918981:- |
| manual\_scaffold\_10\_35264 | 1.8e+1 | 0.96 ± 0.01 |  | 44 | 37 | 0 | 7 | no |  |  |  | blast | ugccuggccucuccaacuaucagu | ugagucggugcugccuggaggu | ugccuggccucuccaacuaucaguguuauuuaaaccacugagucggugcugccuggaggu | manual\_scaffold\_10:592847444..592847504:- |
| manual\_scaffold\_3b\_12059 | 1.8e+1 | 0.96 ± 0.01 |  | 35 | 34 | 0 | 1 | yes |  |  |  | blast | ucgggcacuauuuguugaggga | uccacaaaaaugagugcccaug | ucgggcacuauuuguugagggaaaauaaaugucauuccacaaaaaugagugcccaug | manual\_scaffold\_3b:1034160088..1034160145:- |
| manual\_scaffold\_10\_35051 | 1.8e+1 | 0.96 ± 0.01 |  | 34 | 32 | 0 | 2 | yes |  |  |  | blast | aagagaucugauuggcggacugau | ggccgccauuacaggacucugga | aagagaucugauuggcggacugauugucaagacuauauugcggccgccauuacaggacucugga | manual\_scaffold\_10:352244557..352244621:- |
| manual\_scaffold\_4b\_14875 | 1.8e+1 | 0.96 ± 0.01 |  | 33 | 20 | 0 | 13 | yes |  |  |  | blast | ccugagagcaucaccuggauug | auccaggaagugcucucaugcu | auccaggaagugcucucaugcugauuaacagccugagagcaucaccuggauug | manual\_scaffold\_4b:78944347..78944400:+ |
| manual\_scaffold\_5\_18949 | 1.8e+1 | 0.96 ± 0.01 |  | 34 | 7 | 0 | 27 | yes |  |  |  | blast | ccuagcacuacuuuuaugcuguu | cagcaagagaucagugccugggu | ccuagcacuacuuuuaugcuguuuagcauuguaaacagcaagagaucagugccugggu | manual\_scaffold\_5:1754882130..1754882188:+ |
| manual\_scaffold\_6\_20672 | 1.7e+1 | 0.96 ± 0.01 |  | 33 | 31 | 0 | 2 | yes |  |  |  | blast | ucugaauucucggacuuguuuc | cuacaggucccagaauucaaauga | ucugaauucucggacuuguuucguuuauucuauuaaacuacaggucccagaauucaaauga | manual\_scaffold\_6:30240841..30240902:+ |
| manual\_scaffold\_9\_32540 | 1.7e+1 | 0.96 ± 0.01 |  | 41 | 24 | 0 | 17 | no |  |  |  | blast | uaaugaagagaagccccccac | agaggccuucucagcagauagc | agaggccuucucagcagauagcgcuaacucugaugcuaaugaagagaagccccccac | manual\_scaffold\_9:384309466..384309523:- |
| manual\_scaffold\_4b\_15591 | 1.7e+1 | 0.96 ± 0.01 |  | 33 | 18 | 0 | 15 | yes |  |  |  | blast | ugcaaggaagacugcucugcugc | ugcaagagagucugccuagcugc | ugcaagagagucugccuagcugcucuguugcucuagccugcaaggaagacugcucugcugc | manual\_scaffold\_4b:733649055..733649116:+ |
| manual\_scaffold\_3a\_8981 | 1.7e+1 | 0.96 ± 0.01 |  | 46 | 41 | 0 | 5 | no |  |  |  | blast | ugucagaucggccuauugggc | ucuauaggccaguccgacccu | ugucagaucggccuauugggcacugccugcuagcucuauaggccaguccgacccu | manual\_scaffold\_3a:273873025..273873080:- |
| manual\_scaffold\_3b\_11048 | 1.7e+1 | 0.96 ± 0.01 |  | 34 | 10 | 0 | 24 | yes |  |  |  | blast | uucagugagguucgaauaauuu | uuauuagaaccagacuguacgu | uucagugagguucgaauaauuuuuuuuuaauuugaauuauuagaaccagacuguacgu | manual\_scaffold\_3b:1192457344..1192457402:+ |
| manual\_scaffold\_8\_29479 | 1.7e+1 | 0.96 ± 0.01 |  | 33 | 30 | 0 | 3 | yes |  |  |  | blast | ugacgugugcuuugaauaaagg | uuuauucaaugcgcaggauggcu | uuuauucaaugcgcaggauggcuuuuuuucuuucagcugacgugugcuuugaauaaagg | manual\_scaffold\_8:1337330274..1337330333:+ |
| manual\_scaffold\_12\_39933 | 1.7e+1 | 0.96 ± 0.01 |  | 32 | 28 | 0 | 4 | yes |  |  |  | blast | aaugucuuccucgccacaguca | acuguggcgaggaaggcagagga | aaugucuuccucgccacagucaagguucaucaggugaugacuguggcgaggaaggcagagga | manual\_scaffold\_12:672920037..672920099:- |
| manual\_scaffold\_4b\_16755 | 1.7e+1 | 0.96 ± 0.01 |  | 36 | 34 | 0 | 2 | yes |  |  |  | blast | uaaggaauagcugaaguaauuu | auugccuuggcuauuccucaau | uaaggaauagcugaaguaauuugauuaaccacuugauugccuuggcuauuccucaau | manual\_scaffold\_4b:295321074..295321131:- |
| manual\_scaffold\_1a\_810 | 1.7e+1 | 0.96 ± 0.01 |  | 34 | 28 | 0 | 6 | yes |  |  |  | blast | acuggacagaggagguaguguu | ugcucccaccucuguccacagc | acuggacagaggagguaguguuucauuuccuacacacaugcucccaccucuguccacagc | manual\_scaffold\_1a:1054154752..1054154812:+ |
| manual\_scaffold\_11\_36708 | 1.7e+1 | 0.96 ± 0.01 |  | 31 | 29 | 0 | 2 | yes |  |  |  | blast | uaucccuccggcacugaugucu | caucagugccagagggaaag | uaucccuccggcacugaugucugugguuccugggacaucagugccagagggaaag | manual\_scaffold\_11:1013081684..1013081739:+ |
| manual\_scaffold\_9\_31405 | 1.6e+1 | 0.96 ± 0.01 |  | 31 | 30 | 0 | 1 | yes |  |  |  | blast | uugucuggccucauagauaccu | cauaucuaugaggccacacaaa | cauaucuaugaggccacacaaagccacuagcuuugucuggccucauagauaccu | manual\_scaffold\_9:544790111..544790165:+ |
| manual\_scaffold\_10\_35536 | 1.6e+1 | 0.96 ± 0.01 |  | 33 | 32 | 0 | 1 | yes |  |  |  | blast | uauuccgucggucuuccguuag | accggaagccggaggaauaua | accggaagccggaggaauauaaauguccaguguauauuccgucggucuuccguuag | manual\_scaffold\_10:879960864..879960920:- |
| manual\_scaffold\_6\_23919 | 1.6e+1 | 0.96 ± 0.01 |  | 32 | 29 | 0 | 3 | yes |  |  |  | blast | uugguaaagccaaaagcucu | gagcuacuggcuuugcuaagu | uugguaaagccaaaagcucuggccuuuaauauucguacacagagcuacuggcuuugcuaagu | manual\_scaffold\_6:1011719872..1011719934:- |
| manual\_scaffold\_4b\_15634 | 1.6e+1 | 0.96 ± 0.01 |  | 31 | 30 | 0 | 1 | yes |  |  |  | blast | accagaauccguggcugacacu | gugucagccauguauucuggua | accagaauccguggcugacacuguguucaagacagcguucucucagugucagccauguauucuggua | manual\_scaffold\_4b:775368014..775368081:+ |
| manual\_scaffold\_10\_33470 | 1.6e+1 | 0.96 ± 0.01 |  | 40 | 39 | 0 | 1 | no |  |  |  | blast | aaugaacuagggcucucuucu | aaagcgcuucuaguuugggua | aaugaacuagggcucucuucucguuuagaagcugagaaagcgcuucuaguuugggua | manual\_scaffold\_10:48218465..48218522:+ |
| manual\_scaffold\_3b\_11716 | 1.6e+1 | 0.96 ± 0.01 |  | 40 | 21 | 0 | 19 | no |  |  |  | blast | ucagcacuucuuaugacacccg | cuugucauaacaggugcugcu | cuugucauaacaggugcugcuggugagccugaugcaaucagcacuucuuaugacacccg | manual\_scaffold\_3b:622194446..622194505:- |
| manual\_scaffold\_3a\_7507 | 1.6e+1 | 0.96 ± 0.01 |  | 32 | 30 | 0 | 2 | yes |  |  |  | blast | ccgggauaaauucagcagaggc | gcgcugcuaaacuugucccugu | gcgcugcuaaacuugucccugugucugaaaauuacaccgggauaaauucagcagaggc | manual\_scaffold\_3a:255232403..255232461:+ |
| manual\_scaffold\_3a\_9809 | 1.6e+1 | 0.96 ± 0.01 |  | 32 | 20 | 0 | 12 | yes |  |  |  | blast | cuccucaucuguauaacuuga | caagguacauagauguggacuu | cuccucaucuguauaacuugauuuuauauauauugucaagguacauagauguggacuu | manual\_scaffold\_3a:787945347..787945405:- |
| manual\_scaffold\_11\_35999 | 1.5e+1 | 0.96 ± 0.01 |  | 29 | 19 | 0 | 10 | yes |  |  |  | blast | gccggcgugacggagcaggacu | uccugccccuccgcgcugucg | uccugccccuccgcgcugucggcgauguuaccgccaccgccggcgugacggagcaggacu | manual\_scaffold\_11:152800372..152800432:+ |
| manual\_scaffold\_1b\_1952 | 1.5e+1 | 0.96 ± 0.01 |  | 31 | 23 | 0 | 8 | yes |  |  |  | blast | uuggugucaucagggagguggu | acuccccuguauugcaucuugu | uuggugucaucagggaggugguguucuucaaaacugucacuccccuguauugcaucuugu | manual\_scaffold\_1b:192117430..192117490:+ |
| manual\_scaffold\_3b\_11361 | 1.5e+1 | 0.96 ± 0.01 |  | 29 | 27 | 0 | 2 | yes |  |  |  | blast | ggcgguacgucaugaccugga | cagguccguggcuugccgugg | ggcgguacgucaugaccuggaugcacguaugcguuuccagguccguggcuugccgugg | manual\_scaffold\_3b:358914136..358914194:- |
| manual\_scaffold\_7\_26092 | 1.5e+1 | 0.96 ± 0.01 |  | 39 | 30 | 0 | 9 | no |  |  |  | blast | uccuggacucucucggucucu | agucuuggagcugucuag | uccuggacucucucggucucugugauuuuaucuaucagagucuuggagcugucuag | manual\_scaffold\_7:1360902330..1360902386:+ |
| manual\_scaffold\_6\_23741 | 1.5e+1 | 0.96 ± 0.01 |  | 28 | 18 | 0 | 10 | yes |  |  |  | blast | caacaacuccaucccuucagcu | cugaagggauggaguugcu | cugaagggauggaguugcugaacggcagugcuuucaguucaacaacuccaucccuucagcu | manual\_scaffold\_6:792396262..792396323:- |
| manual\_scaffold\_11\_35859 | 1.5e+1 | 0.96 ± 0.01 |  | 29 | 24 | 0 | 5 | yes |  |  |  | blast | ucaggacaaaguucaugaauga | cauucuuguacuuuguccugu | cauucuuguacuuuguccuguguggcaugaucagaucaggacaaaguucaugaauga | manual\_scaffold\_11:16315440..16315497:+ |
| manual\_scaffold\_10\_35719 | 1.5e+1 | 0.96 ± 0.01 |  | 28 | 18 | 0 | 10 | yes |  |  |  | blast | accucucucggcuccuagagc | cauaggacccgagaggaguuga | accucucucggcuccuagagcaguuaauggaagaauagcauaggacccgagaggaguuga | manual\_scaffold\_10:978054669..978054729:- |
| manual\_scaffold\_8\_29331 | 1.5e+1 | 0.96 ± 0.01 |  | 31 | 30 | 0 | 1 | yes |  |  |  | blast | cuacuuuuaugccuggccuga | aggccagauacagaagucagacu | aggccagauacagaagucagacuaaaagcuuuaguccuacuuuuaugccuggccuga | manual\_scaffold\_8:1148528484..1148528541:+ |
| manual\_scaffold\_12\_38684 | 1.5e+1 | 0.96 ± 0.01 |  | 28 | 26 | 0 | 2 | yes |  |  |  | blast | cuggugcuacucucauguuguu | caacaugagagcagcaucuaga | cuggugcuacucucauguuguuaacauuaucagcaacaugagagcagcaucuaga | manual\_scaffold\_12:662319201..662319256:+ |
| manual\_scaffold\_1b\_2682 | 1.5e+1 | 0.96 ± 0.01 |  | 27 | 21 | 0 | 6 | yes |  |  |  | blast | aucuuggcacuacucuuaugcu | caugagagcagugcuuggau | aucuuggcacuacucuuaugcuguuuacagacagcaugagagcagugcuuggau | manual\_scaffold\_1b:1043920950..1043921004:+ |
| manual\_scaffold\_1b\_2159 | 1.5e+1 | 0.96 ± 0.01 |  | 28 | 26 | 0 | 2 | yes |  |  |  | blast | cagcugcugucaggaucugcu | caggaacugggagcaucuguga | cagcugcugucaggaucugcuuggcauuuguaaucuugagcaggaacugggagcaucuguga | manual\_scaffold\_1b:426081380..426081442:+ |
| manual\_scaffold\_12\_38018 | 1.5e+1 | 0.96 ± 0.01 |  | 27 | 16 | 0 | 11 | yes |  |  |  | blast | uuugaauucugugaccuguaguc | cacaagucccagaauucaaagu | uuugaauucugugaccuguagucuuguuuauuccauaauaaaacgggaccacaagucccagaauucaaagu | manual\_scaffold\_12:91591567..91591638:+ |
| manual\_scaffold\_2b\_6853 | 1.4e+1 | 0.96 ± 0.01 |  | 36 | 29 | 0 | 7 | no |  |  |  | blast | agaguuggagcugccaggaggu | cacuuggccacuguaagacucugc | cacuuggccacuguaagacucugcgcuguuaaggcuuguagaguuggagcugccaggaggu | manual\_scaffold\_2b:431585152..431585213:- |
| manual\_scaffold\_5\_20428 | 1.4e+1 | 0.96 ± 0.01 |  | 28 | 27 | 0 | 1 | yes |  |  |  | blast | uuacuuuuauaucugaccugccc | gacaggucagacauacaagua | gacaggucagacauacaaguagggcuaaauccuuuagucuuacuuuuauaucugaccugccc | manual\_scaffold\_5:1683218926..1683218988:- |
| manual\_scaffold\_2a\_4835 | 1.4e+1 | 0.96 ± 0.01 |  | 34 | 33 | 0 | 1 | yes |  |  |  | blast | uuucugaauauaacauaaaaugu | auuuuauguccauucacaaacu | auuuuauguccauucacaaacuguuuuaugcuugugguuucugaauauaacauaaaaugu | manual\_scaffold\_2a:1391912306..1391912366:+ |
| manual\_scaffold\_2b\_6083 | 1.4e+1 | 0.96 ± 0.01 |  | 30 | 11 | 0 | 19 | yes |  |  |  | blast | gagucuuagaguuguucagugau | cuugaacucucuuagccucug | cuugaacucucuuagccucugugauuuaucuaucagagucuuagaguuguucagugau | manual\_scaffold\_2b:218026110..218026168:+ |
| manual\_scaffold\_5\_20389 | 1.4e+1 | 0.96 ± 0.01 |  | 28 | 27 | 0 | 1 | yes |  |  |  | blast | aacuuggccagugauuguguag | cauagugauuggccaugugaua | aacuuggccagugauuguguaguuuccaaauaaagccacauagugauuggccaugugaua | manual\_scaffold\_5:1632609629..1632609689:- |
| manual\_scaffold\_8\_30160 | 1.4e+1 | 0.96 ± 0.01 |  | 28 | 18 | 0 | 10 | yes |  |  |  | blast | ucgccaucacauuaauccucacc | ugaggacuugugagagggcaaugu | ugaggacuugugagagggcaauguacucuucaucgccaucacauuaauccucacc | manual\_scaffold\_8:589014007..589014062:- |
| manual\_scaffold\_12\_39403 | 1.4e+1 | 0.96 ± 0.01 |  | 27 | 26 | 0 | 1 | yes |  |  |  | blast | aguugcaauguucguuuaaugu | guuaaaaggacauugcaaguua | aguugcaauguucguuuaauguucaugaguacguuaaaaggacauugcaaguua | manual\_scaffold\_12:501415475..501415529:- |
| manual\_scaffold\_8\_29467 | 1.4e+1 | 0.96 ± 0.01 |  | 27 | 22 | 0 | 5 | yes |  |  |  | blast | ugccaccagugaugccauuua | gauggcauccauguugucauug | gauggcauccauguugucauugguaauguccuauuugaugccaccagugaugccauuua | manual\_scaffold\_8:1322677863..1322677922:+ |
| manual\_scaffold\_9\_32813 | 1.4e+1 | 0.96 ± 0.01 |  | 25 | 20 | 0 | 5 | yes |  |  |  | blast | uucgaccaacacagugagugac | cacucacuguguuggucgaaug | uucgaccaacacagugagugaccuauucuauaggucacucacuguguuggucgaaug | manual\_scaffold\_9:766140149..766140206:- |
| manual\_scaffold\_1b\_2557 | 1.4e+1 | 0.96 ± 0.01 |  | 27 | 25 | 0 | 2 | yes |  |  |  | blast | ugcggucacguuaguucucacc | ugaggacuugugagagagc | ugaggacuugugagagagcaauguauucuucauugcggucacguuaguucucacc | manual\_scaffold\_1b:940704168..940704223:+ |
| manual\_scaffold\_1b\_2561 | 1.4e+1 | 0.96 ± 0.01 |  | 27 | 25 | 0 | 2 | yes |  |  |  | blast | ugcggucacguuaguucucacc | ugaggacuugugagagagc | ugaggacuugugagagagcaauguauucuucauugcggucacguuaguucucacc | manual\_scaffold\_1b:940731058..940731113:+ |
| manual\_scaffold\_7\_27647 | 1.4e+1 | 0.96 ± 0.01 |  | 28 | 19 | 0 | 9 | yes |  |  |  | blast | cuaaaaauagguaacucaucauu | ugaugugugaccuauuucaga | cuaaaaauagguaacucaucauugcaauuucuuugaugaugugugaccuauuucaga | manual\_scaffold\_7:1129230001..1129230058:- |
| manual\_scaffold\_6\_24302 | 1.3e+1 | 0.96 ± 0.01 |  | 25 | 14 | 0 | 11 | yes |  |  |  | blast | uacaaacugacaugcgcacuu | agugugcaugucaguuuggcc | agugugcaugucaguuuggccagccauccaaugauguacaaacugacaugcgcacuu | manual\_scaffold\_6:1495339576..1495339633:- |
| manual\_scaffold\_10\_34591 | 1.3e+1 | 0.96 ± 0.01 |  | 25 | 6 | 0 | 19 | yes |  |  |  | blast | cgcgcggacagagaacgggaagg | uuucucguucuccuggcgcccgu | uuucucguucuccuggcgcccguuucgaauuaaacgcgcggacagagaacgggaagg | manual\_scaffold\_10:39981407..39981464:- |
| manual\_scaffold\_6\_22618 | 1.3e+1 | 0.96 ± 0.01 |  | 25 | 24 | 0 | 1 | yes |  |  |  | blast | uuccggaauccuggacccuaccu | gaggguccagcauucgggaau | uuccggaauccuggacccuaccugugaacucaaggaggguccagcauucgggaau | manual\_scaffold\_6:1748901252..1748901307:+ |
| manual\_scaffold\_4a\_13406 | 1.3e+1 | 0.96 ± 0.01 |  | 25 | 6 | 0 | 19 | yes |  |  |  | blast | cgcgcggacagagaacgggaagg | uuucucguucuccuggcgcccgu | uuucucguucuccuggcgcccguuucgaauuaaacgcgcggacagagaacgggaagg | manual\_scaffold\_4a:729651697..729651754:+ |
| manual\_scaffold\_4a\_14566 | 1.3e+1 | 0.96 ± 0.01 |  | 23 | 11 | 0 | 12 | yes |  |  |  | blast | gcaugucggagcacagugcggcu | ccgcacugugcuccgacaug | gcaugucggagcacagugcggcugaaguacuuguguuuucagccgcacugugcuccgacaug | manual\_scaffold\_4a:680991821..680991883:- |
| manual\_scaffold\_8\_29897 | 1.3e+1 | 0.96 ± 0.01 |  | 35 | 22 | 0 | 13 | no |  |  |  | blast | ucuuggacucucucugucucu | gaguuuuggagcuguucaguggu | ucuuggacucucucugucucugugauuuaucuaucagaguuuuggagcuguucaguggu | manual\_scaffold\_8:243145768..243145827:- |
| manual\_scaffold\_4a\_13264 | 1.3e+1 | 0.96 ± 0.01 |  | 23 | 11 | 0 | 12 | yes |  |  |  | blast | gcaugucggagcacagugcggcu | ccgcacugugcuccgacaug | gcaugucggagcacagugcggcugaaaacacaaguacuucagccgcacugugcuccgacaug | manual\_scaffold\_4a:680991820..680991882:+ |
| manual\_scaffold\_6\_23691 | 1.3e+1 | 0.96 ± 0.01 |  | 26 | 15 | 0 | 11 | yes |  |  |  | blast | cuauaucuguguaccuugauga | ucaagguacguagaugugaagu | cuauaucuguguaccuugaugacuuauauaaauauuaucaagguacguagaugugaagu | manual\_scaffold\_6:713653650..713653709:- |
| manual\_scaffold\_10\_34036 | 1.3e+1 | 0.96 ± 0.01 |  | 23 | 21 | 0 | 2 | yes |  |  |  | blast | accucuugugcaccggucuguc | caggccggugcacaagagg | accucuugugcaccggucugucccauaggcacucuauaugggacaggccggugcacaagagg | manual\_scaffold\_10:624596395..624596457:+ |
| manual\_scaffold\_5\_18323 | 1.3e+1 | 0.96 ± 0.01 |  | 34 | 32 | 0 | 2 | no |  |  |  | blast | uugaguuccagaauucugcaau | uugcggaauuugguaacuuugu | uugcggaauuugguaacuuugugcuguacggaggaucauugaguuccagaauucugcaau | manual\_scaffold\_5:936050149..936050209:+ |
| manual\_scaffold\_1b\_2794 | 1.3e+1 | 0.96 ± 0.01 |  | 23 | 20 | 0 | 3 | yes |  |  |  | blast | aacugggcucugucacuuuaga | uaaagugacagagcccaguugu | aacugggcucugucacuuuagagguucucuaaagugacagagcccaguugu | manual\_scaffold\_1b:1170316185..1170316236:+ |
| manual\_scaffold\_3b\_10784 | 1.3e+1 | 0.96 ± 0.01 |  | 23 | 15 | 0 | 8 | yes |  |  |  | blast | cggccaaacacacaugcgcacu | ugcgcaaguguuuuuguccggc | ugcgcaaguguuuuuguccggccguuugaggccggccaaacacacaugcgcacu | manual\_scaffold\_3b:874896645..874896699:+ |
| manual\_scaffold\_7\_25651 | 1.3e+1 | 0.96 ± 0.01 |  | 24 | 22 | 0 | 2 | yes |  |  |  | blast | ucaguauuggaaacuuucauagc | uaugaaaguuccaauacuggag | uaugaaaguuccaauacuggaguaagaaauaaguucuucaguauuggaaacuuucauagc | manual\_scaffold\_7:999045679..999045739:+ |
| manual\_scaffold\_2b\_6915 | 1.3e+1 | 0.96 ± 0.01 |  | 33 | 22 | 0 | 11 | no |  |  |  | blast | ugaguuggugacaccgggaggu | cgccuggcuucaucauuacucugu | cgccuggcuucaucauuacucugugcuaaaauagcucucugaguuggugacaccgggaggu | manual\_scaffold\_2b:472890084..472890145:- |
| manual\_scaffold\_4a\_12755 | 1.3e+1 | 0.96 ± 0.01 |  | 25 | 24 | 0 | 1 | yes |  |  |  | blast | ugcuacugcagaaaagccgcac | ugcuuugcugcacuagcauc | ugcuuugcugcacuagcaucaauauuuuugaugcuacugcagaaaagccgcac | manual\_scaffold\_4a:94398428..94398481:+ |
| manual\_scaffold\_3a\_7239 | 1.3e+1 | 0.96 ± 0.01 |  | 23 | 18 | 0 | 5 | yes |  |  |  | blast | gaauuccgucgacugucaugg | augaccguagagggaauaccgcc | augaccguagagggaauaccgccagaaggcuggcggaauuccgucgacugucaugg | manual\_scaffold\_3a:29937129..29937185:+ |
| manual\_scaffold\_4b\_16754 | 1.2e+1 | 0.96 ± 0.01 |  | 23 | 22 | 0 | 1 | yes |  |  |  | blast | gaggagacaugaaccaguauu | auacugguucaugucuccuccu | auacugguucaugucuccuccuagaaugcgagguucaggaggagacaugaaccaguauu | manual\_scaffold\_4b:295093168..295093227:- |
| manual\_scaffold\_2b\_6081 | 1.2e+1 | 0.96 ± 0.01 |  | 25 | 6 | 0 | 19 | yes |  |  |  | blast | gagucuuagaguuguucaguggu | cuugaacucucuuagccucug | cuugaacucucuuagccucugugauuuaucuaucagagucuuagaguuguucaguggu | manual\_scaffold\_2b:217962687..217962745:+ |
| manual\_scaffold\_1a\_720 | 1.2e+1 | 0.96 ± 0.01 |  | 43 | 36 | 0 | 7 | no |  |  |  | blast | uuagaauuauuuucuuugccuacg | ugacaacaaaauaauucauacc | uuagaauuauuuucuuugccuacgucagcuauuucgugacaacaaaauaauucauacc | manual\_scaffold\_1a:908433745..908433803:+ |
| manual\_scaffold\_11\_36682 | 1.2e+1 | 0.96 ± 0.01 |  | 24 | 21 | 0 | 3 | yes |  |  |  | blast | auuggguuauagaguuaccuuggca | caggagauucagacccaaugg | auuggguuauagaguuaccuuggcauauuaauauugccaggagauucagacccaaugg | manual\_scaffold\_11:1004227639..1004227697:+ |
| manual\_scaffold\_3b\_12100 | 1.2e+1 | 0.96 ± 0.01 |  | 23 | 20 | 0 | 3 | yes |  |  |  | blast | uuaggugagcccagagaggacc | gucucucuggcuucgcugaau | gucucucuggcuucgcugaaugaugguaaagcagcuuaggugagcccagagaggacc | manual\_scaffold\_3b:1073970621..1073970678:- |
| manual\_scaffold\_7\_25703 | 1.2e+1 | 0.96 ± 0.01 |  | 32 | 23 | 0 | 9 | no |  |  |  | blast | cccggaccagaccuggagcuc | agcucccggugugcucugguaau | agcucccggugugcucugguaauaaacugcuugaucacggacccggaccagaccuggagcuc | manual\_scaffold\_7:1054826776..1054826838:+ |
| manual\_scaffold\_5\_20392 | 1.2e+1 | 0.96 ± 0.01 |  | 25 | 14 | 0 | 11 | yes |  |  |  | blast | ucacaaaaugacuccugccu | ugcuggaguucuuuuggauuc | ucacaaaaugacuccugccucaugcauauuaaagaugcuggaguucuuuuggauuc | manual\_scaffold\_5:1632690509..1632690565:- |
| manual\_scaffold\_5\_19390 | 1.2e+1 | 0.96 ± 0.01 |  | 23 | 14 | 0 | 9 | yes |  |  |  | blast | uguuucacugagcugccccaccu | uugggggcagcuuuggaaccaga | uugggggcagcuuuggaaccagacaguuguuacuuguuucacugagcugccccaccu | manual\_scaffold\_5:304494588..304494645:- |
| manual\_scaffold\_3a\_8766 | 1.2e+1 | 0.96 ± 0.01 |  | 25 | 22 | 0 | 3 | yes |  |  |  | blast | uugccaucacuuuaguccucauu | ugacgacuugugagagggcaaugu | ugacgacuugugagagggcaauguauucuuuauugccaucacuuuaguccucauu | manual\_scaffold\_3a:92011075..92011130:- |
| manual\_scaffold\_6\_21074 | 1.2e+1 | 0.96 ± 0.01 |  | 22 | 17 | 0 | 5 | yes |  |  |  | blast | accgccaccagucguaucaug | gauacggccuggugguguucu | accgccaccagucguaucaugacauauggacauaugauacggccuggugguguucu | manual\_scaffold\_6:309505552..309505608:+ |
| manual\_scaffold\_1b\_3231 | 1.2e+1 | 0.96 ± 0.01 |  | 24 | 18 | 0 | 6 | yes |  |  |  | blast | accguggccuuccuagcaaau | auauguauggcggccacg | accguggccuuccuagcaaauguuacaguaagcaacauauguauggcggccacg | manual\_scaffold\_1b:420078700..420078754:- |
| manual\_scaffold\_1a\_1252 | 1.2e+1 | 0.96 ± 0.01 |  | 22 | 15 | 0 | 7 | yes |  |  |  | blast | cagcagcguugggccugaggu | gcucaggacugaggcuguggca | gcucaggacugaggcuguggcaggacuuuggaggcgcugcagcagcguugggccugaggu | manual\_scaffold\_1a:478181025..478181085:- |
| manual\_scaffold\_7\_26648 | 1.2e+1 | 0.96 ± 0.01 |  | 23 | 18 | 0 | 5 | yes |  |  |  | blast | cccuguugaaagucuccuaugc | cucaggaggcuuuccauagcga | cucaggaggcuuuccauagcgaagcaacuuaagcuucccuguugaaagucuccuaugc | manual\_scaffold\_7:116314428..116314486:- |
| manual\_scaffold\_5\_18755 | 1.2e+1 | 0.96 ± 0.01 |  | 23 | 16 | 0 | 7 | yes |  |  |  | blast | cacggguagccaucuuagaaua | uucuuagaugcccacccuugcgu | uucuuagaugcccacccuugcguucuucauuguggaggcacggguagccaucuuagaaua | manual\_scaffold\_5:1482698695..1482698755:+ |
| manual\_scaffold\_7\_25866 | 1.1e+1 | 0.96 ± 0.01 |  | 21 | 19 | 0 | 2 | yes |  |  |  | blast | uuucucguucuccuggcgcccgu | cgcggacagagaacgggaaggu | uuucucguucuccuggcgcccguuucaaauuaaacacgcggacagagaacgggaaggu | manual\_scaffold\_7:1207738681..1207738739:+ |
| manual\_scaffold\_2a\_5246 | 1.1e+1 | 0.96 ± 0.01 |  | 35 | 34 | 0 | 1 | yes |  |  |  | blast | uuccuacguuaaucugugaacu | aacacagauuuuaugaggaau | aacacagauuuuaugaggaauaaaaaauuauggcacuuuuccuacguuaaucugugaacu | manual\_scaffold\_2a:481139151..481139211:- |
| manual\_scaffold\_6\_21209 | 1.1e+1 | 0.96 ± 0.01 |  | 23 | 21 | 0 | 2 | yes |  |  |  | blast | uggcggucacguuaguccucacu | ugaggacuugcaagagggcaaugu | ugaggacuugcaagagggcaauguacucuucauggcggucacguuaguccucacu | manual\_scaffold\_6:361835871..361835926:+ |
| manual\_scaffold\_6\_21072 | 1.1e+1 | 0.96 ± 0.01 |  | 22 | 8 | 0 | 14 | yes |  |  |  | blast | aguuuuggagcugucuugugg | accuggacucucuuaggcu | accuggacucucuuaggcucugugauauuucuuucagaguuuuggagcugucuugugg | manual\_scaffold\_6:307140185..307140243:+ |
| manual\_scaffold\_2b\_6020 | 1.1e+1 | 0.96 ± 0.01 |  | 23 | 22 | 0 | 1 | yes |  |  |  | blast | cacuuggccauucuuaacucu | aauuuggagcugccaagaggu | cacuuggccauucuuaacucugugcuauuagggcuaaaagaauuuggagcugccaagaggu | manual\_scaffold\_2b:139885699..139885760:+ |
| manual\_scaffold\_9\_30961 | 1.1e+1 | 0.96 ± 0.01 |  | 23 | 12 | 0 | 11 | yes |  |  |  | blast | ucugaaaauuccuuaccuuugc | gaagauaaugaauugucagccu | gaagauaaugaauugucagccuagaauccuauucugaaaauuccuuaccuuugc | manual\_scaffold\_9:120678902..120678956:+ |
| manual\_scaffold\_9\_30959 | 1.1e+1 | 0.96 ± 0.01 |  | 23 | 12 | 0 | 11 | yes |  |  |  | blast | ucugaaaauuccuuaccuuugc | gaagauaaugaauugucagccu | gaagauaaugaauugucagccuagaauccuauucugaaaauuccuuaccuuugc | manual\_scaffold\_9:120560647..120560701:+ |
| manual\_scaffold\_7\_26855 | 1.1e+1 | 0.96 ± 0.01 |  | 20 | 19 | 0 | 1 | yes |  |  |  | blast | uuugaauucuggggcuugucguc | cuacaagucccaggauucaaagg | uuugaauucuggggcuugucgucuuguucaugccauugugaaaaagacuacaagucccaggauucaaagg | manual\_scaffold\_7:285477410..285477480:- |
| manual\_scaffold\_2a\_4943 | 1.1e+1 | 0.96 ± 0.01 |  | 20 | 17 | 0 | 3 | yes |  |  |  | blast | ccggccacugauuuuaugcug | agcaugacagcaguguuuggau | ccggccacugauuuuaugcuguuugauauuagaagcagcaugacagcaguguuuggau | manual\_scaffold\_2a:51882011..51882069:- |
| manual\_scaffold\_7\_27870 | 1.0e+1 | 0.96 ± 0.01 |  | 19 | 18 | 0 | 1 | yes |  |  |  | blast | caaacugcgcagacuugagucu | ccucaagucugcgcagugugu | ccucaagucugcgcagugugucuuuaagaguccugaugacaaacugcgcagacuugagucu | manual\_scaffold\_7:1335828574..1335828635:- |
| manual\_scaffold\_4b\_15310 | 1.0e+1 | 0.96 ± 0.01 |  | 20 | 19 | 0 | 1 | yes |  |  |  | blast | cgaggacaccucucagcacugu | agucccggaaggugucccc | cgaggacaccucucagcacugucgaucuuaacaggggagucccggaaggugucccc | manual\_scaffold\_4b:448216573..448216629:+ |
| manual\_scaffold\_2a\_5856 | 1.0e+1 | 0.96 ± 0.01 |  | 19 | 18 | 0 | 1 | yes |  |  |  | blast | ugaacaaacugacauggcaaccu | agugcgcaugucgguuugg | agugcgcaugucgguuuggucagccucggacagcugaacaaacugacauggcaaccu | manual\_scaffold\_2a:1406528730..1406528787:- |
| manual\_scaffold\_4b\_16616 | 1.0e+1 | 0.96 ± 0.01 |  | 20 | 13 | 0 | 7 | yes |  |  |  | blast | aaagcaucagucuucagcccugg | guggugaagauugauguuuuuc | guggugaagauugauguuuuucuggccauagugaaaagcaucagucuucagcccugg | manual\_scaffold\_4b:161177180..161177237:- |
| manual\_scaffold\_4b\_14937 | 1.0e+1 | 0.96 ± 0.01 |  | 20 | 19 | 0 | 1 | yes |  |  |  | blast | caggaggaauacaauaguaagu | cuacuauuguaguacuccucgc | cuacuauuguaguacuccucgcauaaagcaguaaggcaggaggaauacaauaguaagu | manual\_scaffold\_4b:116042971..116043029:+ |
| manual\_scaffold\_5\_20388 | 1.0e+1 | 0.96 ± 0.01 |  | 21 | 16 | 0 | 5 | yes |  |  |  | blast | agaauauuccuagaugacugcu | auggccaucuuggaaugauuuucuu | auggccaucuuggaaugauuuucuuaaggcaaauuaaagaauauuccuagaugacugcu | manual\_scaffold\_5:1628894247..1628894306:- |
| manual\_scaffold\_10\_33558 | 1.0e+1 | 0.96 ± 0.01 |  | 19 | 9 | 0 | 10 | yes |  |  |  | blast | ugacagcuguccccgugccuugu | aggaguagggauugcuguuagc | ugacagcuguccccgugccuuguuggcuguuguaaacaaggaguagggauugcuguuagc | manual\_scaffold\_10:83676137..83676197:+ |
| manual\_scaffold\_10\_34675 | 1.0e+1 | 0.96 ± 0.01 |  | 18 | 6 | 0 | 12 | yes |  |  |  | blast | aaagauguggaggucacgcc | ucgugaccuccacauucauugggcu | ucgugaccuccacauucauugggcuccauacugggaacuaaagauguggaggucacgcc | manual\_scaffold\_10:119751822..119751881:- |
| manual\_scaffold\_9\_33090 | 1.0e+1 | 0.96 ± 0.01 |  | 19 | 17 | 0 | 2 | yes |  |  |  | blast | uuuacuuucucuagcuuccacu | cggcaggaagagaaaguaaaca | cggcaggaagagaaaguaaacaugagucuuagcaucuuguuuacuuucucuagcuuccacu | manual\_scaffold\_9:1037186217..1037186278:- |
| manual\_scaffold\_5\_20395 | 1.0e+1 | 0.96 ± 0.01 |  | 19 | 16 | 0 | 3 | yes |  |  |  | blast | aucaucauguagggccgaccaua | ugugcggaugcauggugacc | aucaucauguagggccgaccauaguguuauaaugcauaugugcggaugcauggugacc | manual\_scaffold\_5:1644593081..1644593139:- |
| manual\_scaffold\_8\_28844 | 1.0e+1 | 0.96 ± 0.01 |  | 19 | 16 | 0 | 3 | yes |  |  |  | blast | cgacccauugcuugcuuguagu | ugcaaacauuuagugggucacu | cgacccauugcuugcuuguaguguuuuucacauuugugauuagcccacugcacugcaaacauuuagugggucacu | manual\_scaffold\_8:373830323..373830398:+ |
| manual\_scaffold\_4b\_15742 | 1.0e+1 | 0.96 ± 0.01 |  | 22 | 21 | 0 | 1 | yes |  |  |  | blast | uagccaucucugaauguuuuu | aaugcauuccaagaugguu | uagccaucucugaauguuuuuuuuuauagagcuaggcaaaaugcauuccaagaugguu | manual\_scaffold\_4b:932538417..932538475:+ |
| manual\_scaffold\_4a\_14245 | 1.0e+1 | 0.96 ± 0.01 |  | 30 | 28 | 0 | 2 | no |  |  |  | blast | uucguucugaguagcucggguc | uucugaggaaaucgaauugaaguauccu | uucguucugaguagcucgggucggcacugaggaguuaucuucugcuucugaggaaaucgaauugaaguauccu | manual\_scaffold\_4a:260687246..260687319:- |
| manual\_scaffold\_11\_36102 | 1.0e+1 | 0.96 ± 0.01 |  | 19 | 17 | 0 | 2 | yes |  |  |  | blast | ucuguagucugucacagcuccu | cgggccuugacagacugccgga | ucuguagucugucacagcuccugucaaauguccacgggccuugacagacugccgga | manual\_scaffold\_11:280310538..280310594:+ |
| manual\_scaffold\_3a\_8621 | 1.0e+1 | 0.96 ± 0.01 |  | 17 | 15 | 0 | 2 | yes |  |  |  | blast | uuacuuccauggucaccucagu | caaggugaccauggagcu | caaggugaccauggagcuaaggcuucugggcugccuuacuuccauggucaccucagu | manual\_scaffold\_3a:902271978..902272035:+ |
| manual\_scaffold\_4b\_15169 | 1.0e+1 | 0.96 ± 0.01 |  | 17 | 16 | 0 | 1 | yes |  |  |  | blast | gaggagacaugaaccaguauu | auacugguucaugucuccuccu | auacugguucaugucuccuccugaaccucgcauucuaggaggagacaugaaccaguauu | manual\_scaffold\_4b:295093169..295093228:+ |
| manual\_scaffold\_6\_23267 | 1.0e+1 | 0.96 ± 0.01 |  | 17 | 16 | 0 | 1 | yes |  |  |  | blast | gcggcaccgggggucuugca | cgggacccccggucccgcuguuc | gcggcaccgggggucuugcaaacacucaccgcugcgggacccccggucccgcuguuc | manual\_scaffold\_6:243722546..243722603:- |
| manual\_scaffold\_1a\_188 | 9.9 | 0.96 ± 0.01 |  | 18 | 14 | 0 | 4 | yes |  |  |  | blast | ugaggacuugccugagggcaau | ugcggucucguuaguccucacc | ugaggacuugccugagggcaauguacucuucauugcggucucguuaguccucacc | manual\_scaffold\_1a:204311448..204311503:+ |
| manual\_scaffold\_7\_24978 | 9.7 | 0.96 ± 0.01 |  | 17 | 15 | 0 | 2 | yes |  |  |  | blast | ucgccuaucagaacauaaaucc | auuuagauuuugacaggcagga | ucgccuaucagaacauaaaucccguaagaaacacugggauuuagauuuugacaggcagga | manual\_scaffold\_7:276216198..276216258:+ |
| manual\_scaffold\_3b\_11093 | 9.5 | 0.96 ± 0.01 |  | 17 | 15 | 0 | 2 | yes |  |  |  | blast | cacaaguccuagaauucaaaga | uugaauccugggacuuguaguc | uugaauccugggacuuguaguccuguguuauaauucaguaaagaagaccacaaguccuagaauucaaaga | manual\_scaffold\_3b:1228869578..1228869648:+ |
| manual\_scaffold\_5\_19068 | 9.5 | 0.96 ± 0.01 |  | 18 | 17 | 0 | 1 | yes |  |  |  | blast | cuuugaacugaucggacccaggg | ugaguugggaaaguucgaagu | cuuugaacugaucggacccagggguauucauucaucucugaguugggaaaguucgaagu | manual\_scaffold\_5:1870908774..1870908833:+ |
| manual\_scaffold\_7\_24682 | 9.5 | 0.96 ± 0.01 |  | 16 | 12 | 0 | 4 | yes |  |  |  | blast | augucccuguggagcuaacggua | cuguugacucuacaggggcauc | augucccuguggagcuaacgguauugcaccuucugcuguugacucuacaggggcauc | manual\_scaffold\_7:58810030..58810087:+ |
| manual\_scaffold\_3b\_10632 | 9.5 | 0.96 ± 0.01 |  | 20 | 17 | 0 | 3 | yes |  |  |  | blast | uccgugcacugugcuguccgca | uggauuccauggagugcgguga | uccgugcacugugcuguccgcauacacguuaaauuuggauuccauggagugcgguga | manual\_scaffold\_3b:703197911..703197968:+ |
| manual\_scaffold\_3b\_11576 | 9.4 | 0.96 ± 0.01 |  | 18 | 4 | 0 | 14 | yes |  |  |  | blast | accgggaugaccuucauaaau | uuuaugaagaucaugucuguca | accgggaugaccuucauaaaugaacgaauccucaggauuuaugaagaucaugucuguca | manual\_scaffold\_3b:531835368..531835427:- |
| manual\_scaffold\_6\_20794 | 9.1 | 0.96 ± 0.01 |  | 17 | 8 | 0 | 9 | yes |  |  |  | blast | caucagaucucuccaaaaaucu | gauuuggagguaucuggaggu | caucagaucucuccaaaaaucugcgcuauaagagcauucagauuuggagguaucuggaggu | manual\_scaffold\_6:121245074..121245135:+ |
| manual\_scaffold\_8\_29785 | 9.1 | 0.96 ± 0.01 |  | 18 | 15 | 0 | 3 | yes |  |  |  | blast | cacuccagagcucuuaauugugu | augauuuagagcuuugggguc | cacuccagagcucuuaauuguguuuauaacggaccaugauuuagagcuuugggguc | manual\_scaffold\_8:106348373..106348429:- |
| manual\_scaffold\_1a\_1744 | 9.1 | 0.96 ± 0.01 |  | 16 | 14 | 0 | 2 | yes |  |  |  | blast | uugggcaccgaucuccacgaac | gguggagaucgaggucgcauc | gguggagaucgaggucgcaucugcugggagcgaauugggcaccgaucuccacgaac | manual\_scaffold\_1a:1098457089..1098457145:- |
| manual\_scaffold\_4a\_12959 | 9.1 | 0.96 ± 0.01 |  | 17 | 16 | 0 | 1 | yes |  |  |  | blast | caauagaauuuuauagacucucu | uagggcuauaaaauucuauu | uagggcuauaaaauucuauugauuagaaaucaauagaauuuuauagacucucu | manual\_scaffold\_4a:262748284..262748337:+ |
| manual\_scaffold\_6\_21331 | 9.1 | 0.96 ± 0.01 |  | 17 | 10 | 0 | 7 | yes |  |  |  | blast | gucugggcaugcaucuguugga | uaguggaugcaggguucagauaau | uaguggaugcaggguucagauaaucaguuaaggucugggcaugcaucuguugga | manual\_scaffold\_6:501243993..501244047:+ |
| manual\_scaffold\_11\_35983 | 8.9 | 0.96 ± 0.01 |  | 28 | 25 | 0 | 3 | no |  |  |  | blast | ccgagcuguugacuuugccugu | auuggcaaaggcaguagguugc | auuggcaaaggcaguagguugcaucuuuauaaugugaaugccgagcuguugacuuugccugu | manual\_scaffold\_11:130778518..130778580:+ |
| manual\_scaffold\_2b\_6997 | 8.8 | 0.96 ± 0.01 |  | 28 | 21 | 0 | 7 | no |  |  |  | blast | uagaaaucguaugaaaggcucacc | uggacuugcauacauuucaacu | uagaaaucguaugaaaggcucaccuuuugaugugguggacuugcauacauuucaacu | manual\_scaffold\_2b:584252943..584253000:- |
| manual\_scaffold\_12\_38934 | 8.8 | 0.96 ± 0.01 |  | 15 | 11 | 0 | 4 | yes |  |  |  | blast | cauggagugggagaaacucug | aguuuuucagaacuccaugcu | aguuuuucagaacuccaugcuguaagcggagcauggagugggagaaacucug | manual\_scaffold\_12:22256339..22256391:- |
| manual\_scaffold\_3a\_9922 | 8.8 | 0.96 ± 0.01 |  | 18 | 17 | 0 | 1 | yes |  |  |  | blast | uuggcuguuuuggaaugguaa | ucaaguuccaguauagccauca | ucaaguuccaguauagccaucauguuuagaaccaagguguuggcuguuuuggaaugguaa | manual\_scaffold\_3a:904579653..904579713:- |
| manual\_scaffold\_5\_19436 | 8.7 | 0.96 ± 0.01 |  | 15 | 14 | 0 | 1 | yes |  |  |  | blast | uucgugcucacuacgcugugc | gcagcguagugagcacgaac | gcagcguagugagcacgaacauuuuacauacauuuucgugcucacuacgcugugc | manual\_scaffold\_5:364164206..364164261:- |
| manual\_scaffold\_6\_24331 | 8.6 | 0.96 ± 0.01 |  | 15 | 14 | 0 | 1 | yes |  |  |  | blast | caacguuagcgccgccuuugagu | gcaaaggcggcgcuaacuu | caacguuagcgccgccuuugaguaaaaaaaugacgcaaaggcggcgcuaacuu | manual\_scaffold\_6:1561359512..1561359565:- |
| manual\_scaffold\_3b\_10428 | 8.6 | 0.96 ± 0.01 |  | 16 | 15 | 0 | 1 | yes |  |  |  | blast | uuugcagagcuuauggacuc | aaacuaugaggucugcaaugc | uuugcagagcuuauggacucaugccuuuaacaacaaagugaaacuaugaggucugcaaugc | manual\_scaffold\_3b:501697299..501697360:+ |
| manual\_scaffold\_3b\_10650 | 8.5 | 0.96 ± 0.01 |  | 15 | 14 | 0 | 1 | yes |  |  |  | blast | uagaguaaccacacaacggaug | guccgcugcgugguuacuccu | uagaguaaccacacaacggauggaacgcaguccgcugcgugguuacuccu | manual\_scaffold\_3b:716327127..716327177:+ |
| manual\_scaffold\_6\_21013 | 8.4 | 0.96 ± 0.01 |  | 25 | 20 | 0 | 5 | no |  |  |  | blast | ugaguuggugcugcccggaggu | ugccuggccucuccaaucgucagu | ugccuggccucuccaaucgucagugcuuuuacggcaacugaguuggugcugcccggaggu | manual\_scaffold\_6:248636525..248636585:+ |
| manual\_scaffold\_2a\_5378 | 8.3 | 0.96 ± 0.01 |  | 13 | 11 | 0 | 2 | yes |  |  |  | blast | cccaaagugacaugugcacu | ugugcaugucacuuuggcuggc | ugugcaugucacuuuggcuggcauaaaacagccgcccaaagugacaugugcacu | manual\_scaffold\_2a:603748414..603748468:- |
| manual\_scaffold\_1b\_2601 | 8.3 | 0.96 ± 0.01 |  | 15 | 12 | 0 | 3 | yes |  |  |  | blast | uagugcaucaacugacaacggc | aaguugucgggagaugagcu | aaguugucgggagaugagcugcgcgaacuggcguagugcaucaacugacaacggc | manual\_scaffold\_1b:984611624..984611679:+ |
| manual\_scaffold\_6\_21388 | 8.2 | 0.96 ± 0.01 |  | 15 | 12 | 0 | 3 | yes |  |  |  | blast | aaaagucuccagcagaagaac | uuuuuuugguggagacucuauc | aaaagucuccagcagaagaacagcuuaucuuuguugacgcuuuuuuugguggagacucuauc | manual\_scaffold\_6:572144508..572144570:+ |
| manual\_scaffold\_4a\_14439 | 8.2 | 0.96 ± 0.01 |  | 16 | 13 | 0 | 3 | yes |  |  |  | blast | ugcccgaaagacuggucugaca | ugagacauguuuuuucggcug | ugcccgaaagacuggucugacaagucauugugugagacauguuuuuucggcug | manual\_scaffold\_4a:486005176..486005229:- |
| manual\_scaffold\_6\_21936 | 8.0 | 0.96 ± 0.01 |  | 20 | 12 | 0 | 8 | yes |  |  |  | blast | aacacaauuuagauuacacagu | uugugugaacaugaauugguuaa | aacacaauuuagauuacacaguauguaaauauugugugaacaugaauugguuaa | manual\_scaffold\_6:1156769903..1156769957:+ |
| manual\_scaffold\_6\_21119 | 8.0 | 0.96 ± 0.01 |  | 15 | 7 | 0 | 8 | yes |  |  |  | blast | agcaggaaccaagcacugauu | ucagugacugucugcugcu | agcaggaaccaagcacugauugauucaacuuaaucagugacugucugcugcu | manual\_scaffold\_6:360255979..360256031:+ |
| manual\_scaffold\_6\_21938 | 8.0 | 0.96 ± 0.01 |  | 20 | 12 | 0 | 8 | yes |  |  |  | blast | aacacaauuuagauuacacagu | uugugugaacaugaauugguuaa | aacacaauuuagauuacacaguauguaaauauugugugaacaugaauugguuaa | manual\_scaffold\_6:1156789704..1156789758:+ |
| manual\_scaffold\_3a\_7386 | 8.0 | 0.96 ± 0.01 |  | 14 | 11 | 0 | 3 | yes |  |  |  | blast | uggggacacccgcugguacuc | agugccaagagguauccuggga | uggggacacccgcugguacuccuguguuaagaucgacagugccaagagguauccuggga | manual\_scaffold\_3a:177615786..177615845:+ |
| manual\_scaffold\_6\_21942 | 8.0 | 0.96 ± 0.01 |  | 20 | 12 | 0 | 8 | yes |  |  |  | blast | aacacaauuuagauuacacagu | uugugugaacaugaauugguuaa | aacacaauuuagauuacacaguauguaaauauugugugaacaugaauugguuaa | manual\_scaffold\_6:1156835370..1156835424:+ |
| manual\_scaffold\_12\_38354 | 7.9 | 0.96 ± 0.01 |  | 14 | 13 | 0 | 1 | yes |  |  |  | blast | agaggcacuuauguugacugc | agucaagguggugccucucugu | agaggcacuuauguugacugcggagcagcaaagaaagaggcagucaagguggugccucucugu | manual\_scaffold\_12:517485714..517485777:+ |
| manual\_scaffold\_3b\_10848 | 7.8 | 0.96 ± 0.01 |  | 14 | 7 | 0 | 7 | yes |  |  |  | blast | aaucuugaugcagcuuucaug | ugaaagcagugucaggauuggu | aaucuugaugcagcuuucaugcuguuuacccacaugaaagcagugucaggauuggu | manual\_scaffold\_3b:957964130..957964186:+ |
| manual\_scaffold\_6\_22227 | 7.7 | 0.96 ± 0.01 |  | 15 | 11 | 0 | 4 | yes |  |  |  | blast | uuggacagcgcuuuacuugagg | uugguaaaaguugugucggga | uuggacagcgcuuuacuugagguuaucauuccuugguaaaaguugugucggga | manual\_scaffold\_6:1418536879..1418536932:+ |
| manual\_scaffold\_3a\_9526 | 7.7 | 0.96 ± 0.01 |  | 13 | 8 | 0 | 5 | yes |  |  |  | blast | uugguugggcauugucuuggga | cuaggacagcgccuuauuugagg | cuaggacagcgccuuauuugagguuaucaauccuugguugggcauugucuuggga | manual\_scaffold\_3a:511356162..511356217:- |
| manual\_scaffold\_6\_22522 | 7.6 | 0.96 ± 0.01 |  | 15 | 12 | 0 | 3 | yes |  |  |  | blast | agaugcaaaacuuguggaauuu | auuccacuugucaugccuuauu | agaugcaaaacuuguggaauuugguacuuuaaacaccgauuccacuugucaugccuuauu | manual\_scaffold\_6:1673518910..1673518970:+ |
| manual\_scaffold\_7\_25604 | 7.6 | 0.96 ± 0.01 |  | 10 | 9 | 0 | 1 | yes |  |  |  | blast | uuggcggcccucugccccacu | uggggcagagggccgucaaucu | uuggcggcccucugccccacugagagcucaugagcucucaguggggcagagggccgucaaucu | manual\_scaffold\_7:967517629..967517692:+ |
| manual\_scaffold\_2a\_4645 | 7.6 | 0.96 ± 0.01 |  | 27 | 24 | 0 | 3 | no |  |  |  | blast | cacaguucggugcuguaaaguuc | uccuuugcaaaccucaaaauuug | uccuuugcaaaccucaaaauuugacuaaaaaacacuuuuuuucacaguucggugcuguaaaguuc | manual\_scaffold\_2a:1096472417..1096472482:+ |
| manual\_scaffold\_10\_34582 | 7.6 | 0.96 ± 0.01 |  | 13 | 9 | 0 | 4 | yes |  |  |  | blast | cguucuaucgccaccgcuguga | acaacagugacugauagcccgu | acaacagugacugauagcccgucugcugaaauauaagucccguucuaucgccaccgcuguga | manual\_scaffold\_10:36708050..36708112:- |
| manual\_scaffold\_2b\_6082 | 7.6 | 0.96 ± 0.01 |  | 14 | 6 | 3 | 5 | yes |  |  |  | blast | gagucuuagaguuguucaguggu | ccugaggaucuggugacucu | gagucuuagaguuguucagugguggacguguugggaacgugcacagacccugaggaucuggugacucu | manual\_scaffold\_2b:217962722..217962790:+ |
| manual\_scaffold\_2a\_4839 | 7.5 | 0.96 ± 0.01 |  | 14 | 12 | 0 | 2 | yes |  |  |  | blast | uuuucaugccuggacucugca | cccgaguuaaggcaggaaaacu | cccgaguuaaggcaggaaaacugcaagaguaaaguuuucaugccuggacucugca | manual\_scaffold\_2a:1394666501..1394666556:+ |
| manual\_scaffold\_6\_23283 | 7.5 | 0.96 ± 0.01 |  | 21 | 14 | 0 | 7 | no |  |  |  | blast | aagugccaagagguguuccagg | ugcgacaccugcugggguuucu | ugcgacaccugcugggguuucuguguccaccaagaagugccaagagguguuccagg | manual\_scaffold\_6:252314375..252314431:- |
| manual\_scaffold\_2a\_5729 | 7.4 | 0.96 ± 0.01 |  | 12 | 11 | 0 | 1 | yes |  |  |  | blast | agggagaagaacagaugucuau | agacaucuguucuccucccugu | agggagaagaacagaugucuaugggaacgucacagacaucuguucuccucccugu | manual\_scaffold\_2a:1205099206..1205099261:- |
| manual\_scaffold\_3b\_10945 | 7.4 | 0.96 ± 0.01 |  | 14 | 11 | 0 | 3 | yes |  |  |  | blast | agaggaugcacuuugcgcuagg | uaguucaaaauacauucucugg | uaguucaaaauacauucucugguacaaaauagaccagaggaugcacuuugcgcuagg | manual\_scaffold\_3b:1052949734..1052949791:+ |
| manual\_scaffold\_4b\_16650 | 7.4 | 0.96 ± 0.01 |  | 13 | 5 | 0 | 8 | yes |  |  |  | blast | auugaauucugggacguauaguc | uacaaaucucggaauucuaag | auugaauucugggacguauaguccguguauuccauucuaaaucaggauuacaaaucucggaauucuaag | manual\_scaffold\_4b:180001742..180001811:- |
| manual\_scaffold\_3a\_8640 | 7.3 | 0.96 ± 0.01 |  | 15 | 14 | 0 | 1 | yes |  |  |  | blast | ccaacagugguuaguaggcagu | cuguuacucaccacugguaccc | ccaacagugguuaguaggcaguguccucuuucuguacuguuacucaccacugguaccc | manual\_scaffold\_3a:917346351..917346409:+ |
| manual\_scaffold\_2a\_5093 | 7.3 | 0.96 ± 0.01 |  | 13 | 10 | 0 | 3 | yes |  |  |  | blast | aaaagcacugcucucaugcug | aacguaggagcagugcaagu | aaaagcacugcucucaugcuguauauaguaguaagcaacguaggagcagugcaagu | manual\_scaffold\_2a:225930698..225930754:- |
| manual\_scaffold\_3a\_9334 | 7.2 | 0.96 ± 0.01 |  | 12 | 11 | 0 | 1 | yes |  |  |  | blast | uucggccuuaauuccuccagcga | ucuggaggaggaggaggc | ucuggaggaggaggaggcugaagggccaucugauacuacuuucggccuuaauuccuccagcga | manual\_scaffold\_3a:380367688..380367751:- |
| manual\_scaffold\_6\_21725 | 7.2 | 0.96 ± 0.01 |  | 14 | 12 | 0 | 2 | yes |  |  |  | blast | ugcaucuaaagauggccacugu | uguggccauguuuuaaugauauu | uguggccauguuuuaaugauauuuguacaauaaaccaaaaugcaucuaaagauggccacugu | manual\_scaffold\_6:920972802..920972864:+ |
| manual\_scaffold\_5\_20419 | 7.2 | 0.96 ± 0.01 |  | 14 | 13 | 0 | 1 | yes |  |  |  | blast | uccggcacuucuaguacugauu | ccgguaguagaggucccagagu | uccggcacuucuaguacugauuuuauaauggcauaaccgguaguagaggucccagagu | manual\_scaffold\_5:1670740589..1670740647:- |
| manual\_scaffold\_1b\_3390 | 7.2 | 0.96 ± 0.01 |  | 13 | 12 | 0 | 1 | yes |  |  |  | blast | ugauagaauaugaauaggucu | gauccuauucaccuucuacau | gauccuauucaccuucuacaucacuguguuucucaagugauagaauaugaauaggucu | manual\_scaffold\_1b:670793049..670793107:- |
| manual\_scaffold\_3b\_12188 | 7.0 | 0.96 ± 0.01 |  | 13 | 9 | 0 | 4 | yes |  |  |  | blast | uugcugcccugugaguucucauu | uggaaacuccuaggguugu | uggaaacuccuaggguuguaguguguccuucauugcugcccugugaguucucauu | manual\_scaffold\_3b:1155387569..1155387624:- |
| manual\_scaffold\_8\_30298 | 7.0 | 0.96 ± 0.01 |  | 12 | 11 | 0 | 1 | yes |  |  |  | blast | cacaaacugacuguggugcuu | agcaccauagcauguuuggggc | agcaccauagcauguuuggggcagacacccuuacacuuugugggcacaaacugacuguggugcuu | manual\_scaffold\_8:810430828..810430893:- |
| manual\_scaffold\_10\_33872 | 6.9 | 0.96 ± 0.01 |  | 15 | 14 | 0 | 1 | yes |  |  |  | blast | aauuccggacuuugacacuacc | uaaugccaaggucuggaacu | aauuccggacuuugacacuaccaaggaaugguaaugccaaggucuggaacu | manual\_scaffold\_10:412726869..412726920:+ |
| manual\_scaffold\_5\_17764 | 6.8 | 0.96 ± 0.01 |  | 11 | 6 | 0 | 5 | yes |  |  |  | blast | augaguacugcagcauuucugc | gaaauccugcaguagucgugc | augaguacugcagcauuucugccacauuuuggguggaaauccugcaguagucgugc | manual\_scaffold\_5:248434044..248434100:+ |
| manual\_scaffold\_4a\_12854 | 6.8 | 0.96 ± 0.01 |  | 12 | 10 | 0 | 2 | yes |  |  |  | blast | uaugguccuaugccaggcugu | cggugcuggccuaggacccuggg | uaugguccuaugccaggcugugugaauuguaagcuacggugcuggccuaggacccuggg | manual\_scaffold\_4a:132467563..132467622:+ |
| manual\_scaffold\_5\_18020 | 6.8 | 0.96 ± 0.01 |  | 10 | 7 | 0 | 3 | yes |  |  |  | blast | agugcgaugcuauugugugccu | guacacgauagcaucgcacggc | agugcgaugcuauugugugccuguauagugaggaucagagguacacgauagcaucgcacggc | manual\_scaffold\_5:521743757..521743819:+ |
| manual\_scaffold\_3b\_10954 | 6.7 | 0.96 ± 0.01 |  | 11 | 10 | 0 | 1 | yes |  |  |  | blast | agcggccucuuuggaaugccu | ucauuccagaagugccgcugu | agcggccucuuuggaaugccuuauuguuuucuugaagaacagucauuccagaagugccgcugu | manual\_scaffold\_3b:1061440684..1061440747:+ |
| manual\_scaffold\_9\_31246 | 6.7 | 0.96 ± 0.01 |  | 11 | 9 | 0 | 2 | yes |  |  |  | blast | acggucauccuggaaugcuuuu | aaguauuccaggauggcugc | acggucauccuggaaugcuuuuugucauaaaguaaaaguauuccaggauggcugc | manual\_scaffold\_9:372976469..372976524:+ |
| manual\_scaffold\_1b\_2766 | 6.5 | 0.96 ± 0.01 |  | 11 | 10 | 0 | 1 | yes |  |  |  | blast | ugugccuuaacuacccaugugc | auaugcgugguuaaggcaga | ugugccuuaacuacccaugugccaaaccaugcauaugcgugguuaaggcaga | manual\_scaffold\_1b:1131212456..1131212508:+ |
| manual\_scaffold\_10\_35612 | 6.4 | 0.96 ± 0.01 |  | 11 | 3 | 0 | 8 | yes |  |  |  | blast | ugcuaccacggcacugugcaaacu | uucaagguacugugguagaa | ugcuaccacggcacugugcaaacuucaccaggcagcuuucaagguacugugguagaa | manual\_scaffold\_10:966235909..966235966:- |
| manual\_scaffold\_8\_28811 | 6.4 | 0.96 ± 0.01 |  | 12 | 10 | 0 | 2 | yes |  |  |  | blast | uaaacguacuacaaaugcagu | agcauuuuguaguacuuuuagu | agcauuuuguaguacuuuuaguagugcuaugauacuaaacguacuacaaaugcagu | manual\_scaffold\_8:310034869..310034925:+ |
| manual\_scaffold\_3b\_11829 | 6.4 | 0.96 ± 0.01 |  | 11 | 10 | 0 | 1 | yes |  |  |  | blast | uaaugggccacagaacgcaagu | uuguacuuuguagcccauaacu | uaaugggccacagaacgcaagugcaaacaaucuccgcuuguacuuuguagcccauaacu | manual\_scaffold\_3b:785069405..785069464:- |
| manual\_scaffold\_4b\_16782 | 6.3 | 0.96 ± 0.01 |  | 12 | 8 | 0 | 4 | yes |  |  |  | blast | ucugagcacuuaucuguugga | cagcggaugcaggguucaga | cagcggaugcaggguucagaaaaucaguuaaagucugagcacuuaucuguugga | manual\_scaffold\_4b:357971256..357971310:- |
| manual\_scaffold\_7\_25701 | 6.3 | 0.96 ± 0.01 |  | 19 | 10 | 0 | 9 | no |  |  |  | blast | accggaccagaccuggagcuc | agcucccggugugcucugguaau | agcucccggugugcucugguaauaaacugcuugaucacggaccggaccagaccuggagcuc | manual\_scaffold\_7:1054826617..1054826678:+ |
| manual\_scaffold\_12\_38287 | 6.3 | 0.96 ± 0.01 |  | 11 | 10 | 0 | 1 | yes |  |  |  | blast | aaaauucaaaccacgucugucu | ccagaugauguuuggauuuuu | ccagaugauguuuggauuuuuucauagcccaagaaaaaauucaaaccacgucugucu | manual\_scaffold\_12:453375242..453375299:+ |
| manual\_scaffold\_8\_30512 | 6.2 | 0.96 ± 0.01 |  | 13 | 12 | 0 | 1 | yes |  |  |  | blast | aaaaugcagccuuuuaccuugc | aaaguaaaaggaugcauuucuu | aaaguaaaaggaugcauuucuuuuacucucugccaucaagaaaaugcagccuuuuaccuugc | manual\_scaffold\_8:1131273822..1131273884:- |
| manual\_scaffold\_5\_20337 | 6.2 | 0.96 ± 0.01 |  | 10 | 8 | 0 | 2 | yes |  |  |  | blast | agcugcacaugucaauuugggc | ccaaccugacaugugcaguu | agcugcacaugucaauuugggcgguauuguuacuguggccaaccugacaugugcaguu | manual\_scaffold\_5:1525225322..1525225380:- |
| manual\_scaffold\_4a\_12890 | 6.2 | 0.96 ± 0.01 |  | 10 | 8 | 0 | 2 | yes |  |  |  | blast | caauccggaugcuacucucaug | ugagagcagcgucuggacuggc | caauccggaugcuacucucauggguauuaccugcaugagagcagcgucuggacuggc | manual\_scaffold\_4a:163329064..163329121:+ |
| manual\_scaffold\_9\_33146 | 6.1 | 0.96 ± 0.01 |  | 13 | 12 | 0 | 1 | yes |  |  |  | blast | uugggcggccgguauuaguaau | gauuaauaccugcaucucagagc | gauuaauaccugcaucucagagcauaaaagaauagcuugggcggccgguauuaguaau | manual\_scaffold\_9:1079383493..1079383551:- |
| manual\_scaffold\_6\_23666 | 6.0 | 0.96 ± 0.01 |  | 11 | 9 | 0 | 2 | yes |  |  |  | blast | ucuaagggcuguucuuccagug | caggaaguccacuccuuaguc | caggaaguccacuccuuaguccuuuuugaagcaagucuaagggcuguucuuccagug | manual\_scaffold\_6:694454158..694454215:- |
| manual\_scaffold\_4b\_17382 | 5.9 | 0.96 ± 0.01 |  | 11 | 3 | 0 | 8 | yes |  |  |  | blast | ugaaggcacuugccuaccacac | uguagugucauuguggcuuc | ugaaggcacuugccuaccacacauuucucuguguagugucauuguggcuuc | manual\_scaffold\_4b:1101345910..1101345961:- |
| manual\_scaffold\_6\_21536 | 5.9 | 0.96 ± 0.01 |  | 10 | 7 | 0 | 3 | yes |  |  |  | blast | caaguagucagcuggucuuagu | uugagaccaacaggcugcuggu | caaguagucagcuggucuuagugugugaucuucauugagaccaacaggcugcuggu | manual\_scaffold\_6:720474284..720474340:+ |
| manual\_scaffold\_3b\_11116 | 5.9 | 0.96 ± 0.01 |  | 11 | 8 | 0 | 3 | yes |  |  |  | blast | ugaguuggcguugccuggaagu | cgccuggcaccuccaaaacucagu | cgccuggcaccuccaaaacucagugccacaagggcauucugaguuggcguugccuggaagu | manual\_scaffold\_3b:32748315..32748376:- |
| manual\_scaffold\_3b\_10743 | 5.8 | 0.96 ± 0.01 |  | 9 | 8 | 0 | 1 | yes |  |  |  | blast | auuuagaauucgacguacggga | cccgucugccaaaaucuaaau | cccgucugccaaaaucuaaaucccauucuauccagugggauuuagaauucgacguacggga | manual\_scaffold\_3b:841918411..841918472:+ |
| manual\_scaffold\_9\_30912 | 5.8 | 0.96 ± 0.01 |  | 20 | 19 | 0 | 1 | no |  |  |  | blast | cacaccuacauuuccagacaccg | guuuugguuuugugcguguauc | cacaccuacauuuccagacaccguauuucugagcacgguuuugguuuugugcguguauc | manual\_scaffold\_9:53666835..53666894:+ |
| manual\_scaffold\_5\_19836 | 5.8 | 0.96 ± 0.01 |  | 18 | 17 | 0 | 1 | no |  |  |  | blast | gucugagcacgcgucuguugga | uagcgaaugcaggguucagaua | uagcgaaugcaggguucagauaaucaguaaagucugagcacgcgucuguugga | manual\_scaffold\_5:860753843..860753896:- |
| manual\_scaffold\_7\_27612 | 5.8 | 0.96 ± 0.01 |  | 10 | 8 | 0 | 2 | yes |  |  |  | blast | ugaggccagcaccuggauuagu | caguccaagugcuucucucaug | caguccaagugcuucucucauguagguuagcagcuugaggccagcaccuggauuagu | manual\_scaffold\_7:1108554689..1108554746:- |
| manual\_scaffold\_7\_26754 | 5.7 | 0.96 ± 0.01 |  | 10 | 9 | 0 | 1 | yes |  |  |  | blast | ccggucagcugguggaaauagu | uguuuccacuaggcugaccagc | uguuuccacuaggcugaccagcagaaacuuuguauuaugaugccggucagcugguggaaauagu | manual\_scaffold\_7:184487786..184487850:- |
| manual\_scaffold\_4b\_17214 | 5.7 | 0.96 ± 0.01 |  | 17 | 15 | 0 | 2 | no |  |  |  | blast | uagcaggcuccacgcacugagc | agaagugucuggagccgccaga | agaagugucuggagccgccagagcuagcaggcuccacgcacugagc | manual\_scaffold\_4b:936967468..936967514:- |
| manual\_scaffold\_2b\_7000 | 5.6 | 0.96 ± 0.01 |  | 10 | 9 | 0 | 1 | yes |  |  |  | blast | uaauuucauccggagcucuaga | uugggcacccgaugaauguuugga | uugggcacccgaugaauguuuggauuuuuuuuuucuaauuucauccggagcucuaga | manual\_scaffold\_2b:586464465..586464522:- |
| manual\_scaffold\_12\_38285 | 5.6 | 0.96 ± 0.01 |  | 8 | 6 | 0 | 2 | yes |  |  |  | blast | uacaguguuggcgucugggugu | uccuggacgccaacauuguaa | uccuggacgccaacauuguaaaguauucaaauucgacuuuacaguguuggcgucugggugu | manual\_scaffold\_12:453282478..453282539:+ |
| manual\_scaffold\_4a\_14712 | 5.6 | 0.96 ± 0.01 |  | 9 | 6 | 0 | 3 | yes |  |  |  | blast | cagccaaacacgcuugcgcacu | agugugcaugucuguuuggcc | agugugcaugucuguuuggccggccgaacacugccagccaaacacgcuugcgcacu | manual\_scaffold\_4a:794258127..794258183:- |
| manual\_scaffold\_10\_33431 | 5.5 | 0.96 ± 0.01 |  | 1752 | 1752 | 0 | 0 | yes |  | xtr-miR-428a |  | blast | aaagugcugucuaguuugggcu | cccaaaccggggcgcucucu | cccaaaccggggcgcucucuccucgcaugagaaagugcugucuaguuugggcu | manual\_scaffold\_10:48071808..48071861:+ |
| manual\_scaffold\_5\_20485 | 5.5 | 0.96 ± 0.01 |  | 8 | 6 | 0 | 2 | yes |  |  |  | blast | gagggcugcuugagcaugugcg | cacaugcuccaguagcccacu | gagggcugcuugagcaugugcgauagcaacuuuacugcacaugcuccaguagcccacu | manual\_scaffold\_5:1762955166..1762955224:- |
| manual\_scaffold\_10\_33479 | 5.5 | 0.96 ± 0.01 |  | 25 | 25 | 0 | 0 | yes |  | xtr-miR-428a |  | blast | uaagugcuuuguguuggaguga | gcuucaaggcacugcgcuugug | gcuucaaggcacugcgcuuguguagcacguugguaccccauaagugcuuuguguuggaguga | manual\_scaffold\_10:48286136..48286198:+ |
| manual\_scaffold\_7\_25560 | 5.5 | 0.96 ± 0.01 |  | 8 | 7 | 0 | 1 | yes |  |  |  | blast | agaagccagaggcuguuuggau | ccagacagcuucuggcuccu | agaagccagaggcuguuuggauguuagcuuaggcuaagccaaaauccagacagcuucuggcuccu | manual\_scaffold\_7:941935846..941935911:+ |
| manual\_scaffold\_10\_33467 | 5.5 | 0.96 ± 0.01 |  | 1754 | 1752 | 0 | 2 | yes |  | xtr-miR-428a |  | blast | aaagugcugucuaguuugggcu | cgcccacaccggggcgcucucu | cgcccacaccggggcgcucucuccucgcaugagaaagugcugucuaguuugggcu | manual\_scaffold\_10:48218312..48218367:+ |
| manual\_scaffold\_10\_33445 | 5.3 | 0.96 ± 0.01 |  | 1754 | 1752 | 0 | 2 | yes |  | xtr-miR-428a |  | blast | aaagugcugucuaguuugggcu | cgcccacaccggggcgcucucu | cgcccacaccggggcgcucucuccucgcaugagaaagugcugucuaguuugggcu | manual\_scaffold\_10:48079610..48079665:+ |
| manual\_scaffold\_4b\_16619 | 5.2 | 0.96 ± 0.01 |  | 10 | 9 | 0 | 1 | yes |  |  |  | blast | cagccuuuuccuugacucugcu | ggggagucagagaaucggcauu | cagccuuuuccuugacucugcuuuuucuaagaaguaaggggagucagagaaucggcauu | manual\_scaffold\_4b:162183388..162183447:- |
| manual\_scaffold\_5\_19164 | 5.2 | 0.96 ± 0.01 |  | 129 | 118 | 11 | 0 | yes |  | xtr-miR-129 |  | blast | uuuuuugcuggaacauuucugg | ggaaauguuuuagccaaaaagg | ggaaauguuuuagccaaaaagguucagccagacuuguaggguuuucuuuuuugcuggaacauuucugg | manual\_scaffold\_5:33306294..33306362:- |
| manual\_scaffold\_2a\_5867 | 5.1 | 0.96 ± 0.01 |  | 12 | 6 | 0 | 6 | yes |  |  |  | blast | ugugaccagaacagaaccaugau | ugugcuucuuuuccgguccauu | ugugcuucuuuuccgguccauuuauauaugcguagaaauuaaugugaccagaacagaaccaugau | manual\_scaffold\_2a:1420485779..1420485844:- |
| manual\_scaffold\_3a\_9098 | 5.1 | 0.96 ± 0.01 |  | 16 | 13 | 0 | 3 | no |  |  |  | blast | ugccagaggugauugucccgau | gcgggacaaauaccccugaccu | gcgggacaaauaccccugaccuugccugaucaggugccagaggugauugucccgau | manual\_scaffold\_3a:354226309..354226365:- |
| manual\_scaffold\_12\_38644 | 5.0 | 0.96 ± 0.01 |  | 8 | 7 | 0 | 1 | yes |  |  |  | blast | gcuuggacuggaucaaugcugu | agcauugguuuaggcaagc | gcuuggacuggaucaaugcuguaacaauacagcauugguuuaggcaagc | manual\_scaffold\_12:639744776..639744825:+ |
| manual\_scaffold\_2a\_5501 | 5.0 | 0.96 ± 0.01 |  | 7 | 6 | 0 | 1 | yes |  |  |  | blast | uccgacgcugcucucaugcug | agcaugaaagcagcgucuggau | uccgacgcugcucucaugcuggguaauaguauuaccagcaugaaagcagcgucuggau | manual\_scaffold\_2a:777765060..777765118:- |
| manual\_scaffold\_5\_18795 | 5.0 | 0.96 ± 0.01 |  | 13 | 10 | 0 | 3 | yes |  |  |  | blast | auguuuggauaugauuuucucu | uguuaaucauuuuccaucugugc | uguuaaucauuuuccaucugugcguuugcuagugcauguuuggauaugauuuucucu | manual\_scaffold\_5:1521349954..1521350011:+ |
| manual\_scaffold\_7\_25343 | 4.9 | 0.95 ± 0.01 |  | 18 | 17 | 0 | 1 | no |  |  |  | blast | uccccauguaugcaaagccucucu | gaugguuuguugcugggguga | gaugguuuguugcuggggugauguuucauagugcuuuccccauguaugcaaagccucucu | manual\_scaffold\_7:688643376..688643436:+ |
| manual\_scaffold\_8\_29806 | 4.9 | 0.95 ± 0.01 |  | 21245 | 21245 | 0 | 0 | yes |  | xtr-miR-223 |  | blast | ugucaguuugucaaauacc | cuuuugaugcauuuccagg | cuuuugaugcauuuccagguuuacaguauguuguaaaucugucaguuugucaaauacc | manual\_scaffold\_8:111334193..111334251:- |
| manual\_scaffold\_10\_33434 | 4.9 | 0.95 ± 0.01 |  | 1930 | 1853 | 0 | 77 | yes |  | xtr-miR-428a |  | blast | aaagugcuucuaguuugggc | acuugaacuagggcucucuucu | acuugaacuagggcucucuucucguuucgaaacugagaaagugcuucuaguuugggc | manual\_scaffold\_10:48071954..48072011:+ |
| manual\_scaffold\_4b\_16885 | 4.8 | 0.95 ± 0.01 |  | 8 | 6 | 0 | 2 | yes |  |  |  | blast | acccagaggauaguugugcuc | gcauacauuauucuuugggug | acccagaggauaguugugcucggugcuacaugaacagagcauacauuauucuuugggug | manual\_scaffold\_4b:496463735..496463794:- |
| manual\_scaffold\_10\_33435 | 4.8 | 0.95 ± 0.01 |  | 387 | 363 | 0 | 24 | yes |  | xtr-miR-428a |  | blast | aaagugcucuucuguuggggc | gcccaaacaccagcacuuccu | gcccaaacaccagcacuuccuuuguuuucaccaguaagaaagugcucuucuguuggggc | manual\_scaffold\_10:48074787..48074846:+ |
| manual\_scaffold\_7\_26832 | 4.8 | 0.95 ± 0.01 |  | 11 | 10 | 0 | 1 | yes |  |  |  | blast | acggauuggucauuuugcacucc | ugguggaaaauucaaugcguuu | ugguggaaaauucaaugcguuugcacuuaagggaaaacggauuggucauuuugcacucc | manual\_scaffold\_7:260610742..260610801:- |
| manual\_scaffold\_1b\_3745 | 4.7 | 0.95 ± 0.01 |  | 12 | 11 | 1 | 0 | yes |  | xtr-miR-17-5p |  | blast | caaagugcucauagugaaa | uuauauguaucacuugga | caaagugcucauagugaaaacccuauuugucaguguguuuuuauauguaucacuugga | manual\_scaffold\_1b:1155731014..1155731072:- |
| manual\_scaffold\_5\_19122 | 4.6 | 0.95 ± 0.01 |  | 16 | 12 | 0 | 4 | no |  |  |  | blast | cgccuggcuccaucaauacucu | aguuuggugacgccuggaggu | cgccuggcuccaucaauacucugcgcuguaauagcuuucggaguuuggugacgccuggaggu | manual\_scaffold\_5:1905862643..1905862705:+ |
| manual\_scaffold\_12\_38766 | 4.5 | 0.95 ± 0.01 |  | 17 | 11 | 0 | 6 | no |  |  |  | blast | ucaggcuugugucuuagugaau | uucacagaucucaggcuugugucu | ucaggcuugugucuuagugaauggagauauucuacaguguucacagaucucaggcuugugucu | manual\_scaffold\_12:700598622..700598685:+ |
| manual\_scaffold\_5\_19652 | 4.5 | 0.95 ± 0.01 |  | 7 | 5 | 0 | 2 | yes |  |  |  | blast | auuccaggugcuucucucaugc | uaugagagcaacaucuggauugg | auuccaggugcuucucucaugcugguuauuaguaugagagcaacaucuggauugg | manual\_scaffold\_5:666438913..666438968:- |
| manual\_scaffold\_9\_33138 | 4.4 | 0.95 ± 0.01 |  | 8517 | 8517 | 0 | 0 | yes |  | xtr-miR-142-5p |  | blast | cauaaaguagaaagcacu | uguuauguauuugaguggc | uguuauguauuugaguggcauaguguggagucacacugaguggcauacacuggagcgucauaaaguagaaagcacu | manual\_scaffold\_9:1076608997..1076609073:- |
| manual\_scaffold\_4b\_17500 | 4.4 | 0.95 ± 0.01 |  | 11199 | 11199 | 0 | 0 | yes |  | xtr-miR-122 |  | blast | uggagugugacaauggugu | auuacugacacccauu | auuacugacacccauucugcccugagcaauggguuaauggagugugacaauggugu | manual\_scaffold\_4b:1199724648..1199724704:- |
| manual\_scaffold\_9\_32474 | 4.4 | 0.95 ± 0.01 |  | 133 | 133 | 0 | 0 | yes |  | xtr-miR-155 |  | blast | uuaaugcuaaucgugauag | agcaugauuaguggcacgu | agcaugauuaguggcacguuuuuuuuaaugcuaaucgugauag | manual\_scaffold\_9:299486008..299486051:- |
| manual\_scaffold\_12\_37933 | 4.4 | 0.95 ± 0.01 |  | 6 | 5 | 0 | 1 | yes |  |  |  | blast | uguggcacuguuagcucucucc | agagagcugaagagugccacauc | agagagcugaagagugccacaucuuggaagauguggcacuguuagcucucucc | manual\_scaffold\_12:68836941..68836994:+ |
| manual\_scaffold\_4b\_16838 | 4.4 | 0.95 ± 0.01 |  | 17 | 15 | 0 | 2 | no |  |  |  | blast | uuguagagaucgugaccccca | ugugguccccugccucuacacu | ugugguccccugccucuacacugacccaccgcguccaacauuguagagaucgugaccccca | manual\_scaffold\_4b:437880577..437880638:- |
| manual\_scaffold\_9\_31798 | 4.4 | 0.95 ± 0.01 |  | 7 | 6 | 0 | 1 | yes |  |  |  | blast | acagggcccccaacuucacagu | cuugaguagggggcccugguu | acagggcccccaacuucacaguggaaaaauuagacauccuugaguagggggcccugguu | manual\_scaffold\_9:878175878..878175937:+ |
| manual\_scaffold\_5\_18991 | 4.4 | 0.95 ± 0.01 |  | 7 | 4 | 0 | 3 | yes |  |  |  | blast | auucagccuuugaggguagga | acugcccucacuggcugagucu | acugcccucacuggcugagucugacagguugucuuuuagauucagccuuugaggguagga | manual\_scaffold\_5:1810897460..1810897520:+ |
| manual\_scaffold\_5\_20083 | 4.2 | 0.95 ± 0.01 |  | 17 | 15 | 0 | 2 | no |  |  |  | blast | aagagcacuacagaacucagagc | caagguucuagagagcucuccc | aagagcacuacagaacucagagccacaguauaaccucgccaagguucuagagagcucuccc | manual\_scaffold\_5:1201956749..1201956810:- |
| manual\_scaffold\_1a\_1751 | 4.1 | 0.95 ± 0.01 |  | 7 | 6 | 0 | 1 | yes |  |  |  | blast | cagauuaacacauuuuagugu | cacuaaaauggauuaaucaca | cagauuaacacauuuuagugugguauccacaugauugcacuaaaauggauuaaucaca | manual\_scaffold\_1a:1102037676..1102037734:- |
| manual\_scaffold\_3a\_8671 | 4.1 | 0.95 ± 0.01 |  | 15 | 13 | 0 | 2 | no |  |  |  | blast | ucugagcacacauccguugga | uaccggaugcaggguucagau | uaccggaugcaggguucagauaaucaguuaaagucugagcacacauccguugga | manual\_scaffold\_3a:977936429..977936483:+ |
| manual\_scaffold\_6\_22142 | 4.1 | 0.95 ± 0.01 |  | 6 | 3 | 0 | 3 | yes |  |  |  | blast | accucuguguguaggucuccu | ggagacuuauacacagagggc | accucuguguguaggucuccuggggauagaacuaacaggagacuuauacacagagggc | manual\_scaffold\_6:1366335487..1366335545:+ |
| manual\_scaffold\_11\_37604 | 4.0 | 0.95 ± 0.01 |  | 11 | 10 | 0 | 1 | yes |  |  |  | blast | ugcgaccuucugacuuugccagu | uuggaaaagccaauagcucgac | uuggaaaagccaauagcucgacacucagauuuuaaaggugcgaccuucugacuuugccagu | manual\_scaffold\_11:904109756..904109817:- |
| manual\_scaffold\_2a\_5241 | 4.0 | 0.95 ± 0.01 |  | 6 | 2 | 0 | 4 | yes |  |  |  | blast | gagagcagugucaggauu | aucuuggcacuacucuuaugc | aucuuggcacuacucuuaugcaauguugguaaccuaacagcaagagagcagugucaggauu | manual\_scaffold\_2a:466548770..466548831:- |
| manual\_scaffold\_9\_32033 | 3.8 | 0.93 ± 0.01 |  | 7 | 6 | 0 | 1 | yes |  |  |  | blast | uuggaagccagcucuuggagua | cuauucaagaggggauuccaauu | cuauucaagaggggauuccaauuguaauguuguaaaguuggaagccagcucuuggagua | manual\_scaffold\_9:1091622379..1091622438:+ |
| manual\_scaffold\_2a\_3958 | 3.8 | 0.93 ± 0.01 |  | 37 | 37 | 0 | 0 | yes |  | xtr-miR-425-5p |  | blast | uaugacaccccacacaacug | guugugaugugauaau | uaugacaccccacacaacugaaaauacaccccugguuaguacaguuggguugugaugugauaau | manual\_scaffold\_2a:171547136..171547200:+ |
| manual\_scaffold\_4b\_15721 | 3.8 | 0.93 ± 0.01 |  | 7 | 1 | 0 | 6 | yes |  |  |  | blast | acaaugcccaaaggcaaagucu | gauuuuccuuuggacauugcag | acaaugcccaaaggcaaagucugcuuggagguagccaggauuuuccuuuggacauugcag | manual\_scaffold\_4b:905107341..905107401:+ |
| manual\_scaffold\_4b\_14951 | 3.8 | 0.93 ± 0.01 |  | 212 | 212 | 0 | 0 | yes |  | xtr-miR-454-3p |  | blast | cagugcaauaguauuguc | cauuuuuguugcacaaau | cauuuuuguugcacaaaucagcccuguccccagcagugcaauaguauuguc | manual\_scaffold\_4b:127670942..127670993:+ |
| manual\_scaffold\_4b\_16304 | 3.7 | 0.93 ± 0.01 |  | 6 | 5 | 0 | 1 | yes |  |  |  | blast | gcgagaccgacuggcauugc | aauggcaaccagucuuacuug | aauggcaaccagucuuacuuguauguaaagguaagcgagaccgacuggcauugc | manual\_scaffold\_4b:120882324..120882378:- |
| manual\_scaffold\_6\_21944 | 3.6 | 0.93 ± 0.01 |  | 14 | 12 | 0 | 2 | yes |  |  |  | blast | aacacaauuuagauuacacagu | uugugugaacaugaauuugu | aacacaauuuagauuacacaguauguaaauauugugugaacaugaauuugu | manual\_scaffold\_6:1156856229..1156856280:+ |
| manual\_scaffold\_6\_21940 | 3.4 | 0.93 ± 0.01 |  | 14 | 12 | 0 | 2 | yes |  |  |  | blast | aacacaauuuagauuacacagu | uugugugaacaugaauuugu | aacacaauuuagauuacacaguauguaaauauugugugaacaugaauuugu | manual\_scaffold\_6:1156810476..1156810527:+ |
| manual\_scaffold\_8\_30464 | 3.4 | 0.93 ± 0.01 |  | 21 | 21 | 0 | 0 | yes |  |  |  | blast | ugcccuggccuguacugacaca | ugucaguacaggccagggcaca | ugucaguacaggccagggcacaucggcuuggaugugcccuggccuguacugacaca | manual\_scaffold\_8:1058934790..1058934846:- |
| manual\_scaffold\_8\_29267 | 3.4 | 0.93 ± 0.01 |  | 21 | 21 | 0 | 0 | yes |  |  |  | blast | ugcccuggccuguacugacaca | ugucaguacaggccagggcaca | ugucaguacaggccagggcacauccaagccgaugugcccuggccuguacugacaca | manual\_scaffold\_8:1058934792..1058934848:+ |
| manual\_scaffold\_11\_36197 | 3.3 | 0.93 ± 0.01 |  | 16 | 15 | 0 | 1 | no |  |  |  | blast | acacuggcacucuggaccaggu | ugggaucaguugaccaguuaac | ugggaucaguugaccaguuaacuuguuuugaugaaaguacacuggcacucuggaccaggu | manual\_scaffold\_11:419222228..419222288:+ |
| manual\_scaffold\_1b\_3617 | 3.3 | 0.93 ± 0.01 |  | 17 | 16 | 0 | 1 | no |  |  |  | blast | acuugugucauggauaugugu | cacaccugcauagacacaugu | acuugugucauggauauguguaccuuuuggucacaauguacacaccugcauagacacaugu | manual\_scaffold\_1b:980373151..980373212:- |
| manual\_scaffold\_4b\_17376 | 3.3 | 0.93 ± 0.01 |  | 15 | 8 | 0 | 7 | no |  |  |  | blast | ucugggcacacaucuguugga | uaguggaugcaggguucagauaau | uaguggaugcaggguucagauaaucaguaaagucugggcacacaucuguugga | manual\_scaffold\_4b:1097907831..1097907884:- |
| manual\_scaffold\_2a\_4893 | 3.3 | 0.93 ± 0.01 |  | 19 | 19 | 0 | 0 | yes |  | xtr-miR-31a |  | blast | aggcaagauguuggcauu | ugcuaagucugacugg | ugcuaagucugacugguaacaucuccucuaggcacaaugugauuuagucaaggaaaguccaggcaagauguuggcauu | manual\_scaffold\_2a:1487385799..1487385877:+ |
| manual\_scaffold\_4b\_15800 | 3.2 | 0.93 ± 0.01 |  | 9 | 7 | 0 | 2 | yes |  |  |  | blast | uacgaugcauccagguuuaaug | uuagcccaggcugcgucauagc | uacgaugcauccagguuuaaugcacuucaaaaagaaaagagcauuagcccaggcugcgucauagc | manual\_scaffold\_4b:999103862..999103927:+ |
| manual\_scaffold\_5\_19758 | 2.9 | 0.92 ± 0.01 |  | 12 | 12 | 0 | 0 | yes |  |  |  | blast | acucgagcuguguggcccaua | ugggcuacacagcucaagugc | ugggcuacacagcucaagugcagacagucugcacucgagcuguguggcccaua | manual\_scaffold\_5:772578618..772578671:- |
| manual\_scaffold\_9\_31255 | 2.9 | 0.92 ± 0.01 |  | 302 | 302 | 0 | 0 | yes |  | xtr-miR-454-3p |  | blast | cagugcaauaaugaaagg | uaucguuauucugga | uaucguuauucuggagcacuccagugcaauaaugaaagg | manual\_scaffold\_9:381126006..381126045:+ |
| manual\_scaffold\_8\_29821 | 2.9 | 0.92 ± 0.01 |  | 16 | 15 | 0 | 1 | yes |  |  |  | blast | uaacccgaggcuaacgacacuc | gucguuagccucggguuau | uaacccgaggcuaacgacacuccgagcuggaaaguugagugucguuagccucggguuau | manual\_scaffold\_8:138446022..138446081:- |
| manual\_scaffold\_8\_28612 | 2.8 | 0.92 ± 0.01 |  | 18 | 17 | 0 | 1 | yes |  |  |  | blast | uaacccgaggcuaacgacacuc | gucguuagccucggguuau | uaacccgaggcuaacgacacucaacuuuccagcucggagugucguuagccucggguuau | manual\_scaffold\_8:138446023..138446082:+ |
| manual\_scaffold\_3a\_9528 | 2.8 | 0.92 ± 0.01 |  | 18 | 18 | 0 | 0 | yes |  |  |  | blast | cuugucuccugaucagaaaccu | guuucugaucaggagacaagcc | guuucugaucaggagacaagccaauuggcuugucuccugaucagaaaccu | manual\_scaffold\_3a:511923801..511923851:- |
| manual\_scaffold\_3b\_11836 | 2.8 | 0.92 ± 0.01 |  | 10 | 10 | 0 | 0 | yes |  | xtr-miR-449c-5p |  | blast | uggcagugacggaccugc | gcuuccguuccugugacu | gcuuccguuccugugacucguuacgguuuuggcagugacggaccugc | manual\_scaffold\_3b:789782288..789782335:- |
| manual\_scaffold\_9\_32862 | 2.7 | 0.92 ± 0.01 |  | 19 | 19 | 0 | 0 | yes |  |  |  | blast | auccguucggaugugugagggcu | cccucacacauccgaacagauga | cccucacacauccgaacagaugaagcaugacauccguucggaugugugagggcu | manual\_scaffold\_9:812560525..812560579:- |
| manual\_scaffold\_3a\_7939 | 2.7 | 0.92 ± 0.01 |  | 18 | 18 | 0 | 0 | yes |  |  |  | blast | cuugucuccugaucagaaaccu | guuucugaucaggagacaagcc | guuucugaucaggagacaagccaauuggcuugucuccugaucagaaaccu | manual\_scaffold\_3a:511923803..511923853:+ |
| manual\_scaffold\_3a\_8757 | 2.7 | 0.92 ± 0.01 |  | 19 | 17 | 0 | 2 | no |  |  |  | blast | ucacauuuauagccucuagacug | ucugagacuguaaauugauuu | ucacauuuauagccucuagacugugccuggaagaccgucugagacuguaaauugauuu | manual\_scaffold\_3a:75673205..75673263:- |
| manual\_scaffold\_8\_29081 | 2.7 | 0.92 ± 0.01 |  | 3 | 2 | 0 | 1 | yes |  |  |  | blast | ugaacuauagaaacagaauugg | aguuuuguuuccacaguucacc | aguuuuguuuccacaguucaccucuagguguuauguggaggugaacuauagaaacagaauugg | manual\_scaffold\_8:749914939..749915002:+ |
| manual\_scaffold\_7\_27425 | 2.6 | 0.92 ± 0.01 |  | 63 | 58 | 0 | 5 | yes |  |  |  | blast | acggaauaucauaagaagcuga | cuucuuaugauauuccgugauu | acggaauaucauaagaagcugacgucaugacgucagcuucuuaugauauuccgugauu | manual\_scaffold\_7:943971967..943972025:- |
| manual\_scaffold\_11\_37726 | 2.6 | 0.92 ± 0.01 |  | 35 | 23 | 0 | 12 | yes |  |  |  | blast | augggcugcacagcgcaagcgca | uugcccuguucagcccauaac | augggcugcacagcgcaagcgcagacucucugaacuugcccuguucagcccauaac | manual\_scaffold\_11:994073295..994073351:- |
| manual\_scaffold\_9\_32810 | 2.6 | 0.92 ± 0.01 |  | 3 | 2 | 0 | 1 | yes |  |  |  | blast | guagcucauucgguccaauuc | gacuggucuggaugggcuuag | guagcucauucgguccaauucuuuacuccacauucugagacuggucuggaugggcuuag | manual\_scaffold\_9:762944095..762944154:- |
| manual\_scaffold\_7\_25564 | 2.6 | 0.92 ± 0.01 |  | 63 | 58 | 0 | 5 | yes |  |  |  | blast | acggaauaucauaagaagcuga | cuucuuaugauauuccgugauu | acggaauaucauaagaagcugacgucaugacgucagcuucuuaugauauuccgugauu | manual\_scaffold\_7:943971971..943972029:+ |
| manual\_scaffold\_4a\_14485 | 2.6 | 0.92 ± 0.01 |  | 164 | 164 | 0 | 0 | yes |  |  |  | blast | cccgcccgucgaauuaugaguc | cucguaaugcggcgggcggauu | cccgcccgucgaauuaugaguccauuauuuccuauggaacucguaaugcggcgggcggauu | manual\_scaffold\_4a:565350621..565350682:- |
| manual\_scaffold\_9\_31214 | 2.5 | 0.92 ± 0.01 |  | 155 | 155 | 0 | 0 | yes |  |  |  | blast | auggcaaugccaaucggucuugc | gagaccuuuuggcauugccguuu | auggcaaugccaaucggucuugcuuguaugcaaagacaagugagaccuuuuggcauugccguuu | manual\_scaffold\_9:327155725..327155789:+ |
| manual\_scaffold\_6\_22726 | 2.5 | 0.92 ± 0.01 |  | 11 | 9 | 0 | 2 | no |  |  |  | blast | ucugaggggugucaguacugccu | ucaguacugccucugacagcagc | ucaguacugccucugacagcagcuagcccugcucugaggggugucaguacugccu | manual\_scaffold\_6:45746919..45746974:- |
| manual\_scaffold\_6\_22728 | 2.5 | 0.92 ± 0.01 |  | 11 | 9 | 0 | 2 | no |  |  |  | blast | ucugaggggugucaguacugccu | ucaguacugccucugacagcagc | ucaguacugccucugacagcagcuagcccugcucugaggggugucaguacugccu | manual\_scaffold\_6:45747060..45747115:- |
| manual\_scaffold\_5\_19455 | 2.5 | 0.92 ± 0.01 |  | 130 | 128 | 0 | 2 | yes |  |  |  | blast | auagaggacacagagcacacugu | gugugcucuguguccucugc | auagaggacacagagcacacuguguguguccuugugugcucuguguccucugc | manual\_scaffold\_5:406846727..406846780:- |
| manual\_scaffold\_3a\_7300 | 2.5 | 0.92 ± 0.01 |  | 29 | 29 | 0 | 0 | yes |  |  |  | blast | ucaccgggcugacaggcagaaac | uucugccugucagcccgguggaa | ucaccgggcugacaggcagaaacgucauaauacgacguuucugccugucagcccgguggaa | manual\_scaffold\_3a:85138730..85138791:+ |
| manual\_scaffold\_2a\_5036 | 2.4 | 0.92 ± 0.01 |  | 48 | 48 | 0 | 0 | yes |  | xtr-let-7a |  | blast | ugagguagaagauuguauagu | ugugaaagauagugcauaua | ugugaaagauagugcauauaacaugcuaauguuaugagguagaagauuguauagu | manual\_scaffold\_2a:165361943..165361998:- |
| manual\_scaffold\_9\_33226 | 2.4 | 0.92 ± 0.01 |  | 15 | 15 | 0 | 0 | yes |  |  |  | blast | cccagaagaacuuagaggcag | gccucuaaguucuucugggua | gccucuaaguucuucuggguaagucggcuacccagaagaacuuagaggcag | manual\_scaffold\_9:1167173081..1167173132:- |
| manual\_scaffold\_6\_20927 | 2.4 | 0.92 ± 0.01 |  | 14 | 14 | 0 | 0 | yes |  |  |  | blast | ccgaggucaauaacgcugcacu | ugcagcggcauuggcuucggcu | ugcagcggcauuggcuucggcucucaggaagagccgaggucaauaacgcugcacu | manual\_scaffold\_6:190089711..190089766:+ |
| manual\_scaffold\_4a\_13373 | 2.4 | 0.92 ± 0.01 |  | 29 | 27 | 0 | 2 | yes |  |  |  | blast | uauucugaagcggagcaaugcu | agcauugcuccgcuucagaau | agcauugcuccgcuucagaauagcuccaguagcuauucugaagcggagcaaugcu | manual\_scaffold\_4a:705871355..705871410:+ |
| manual\_scaffold\_12\_37882 | 2.4 | 0.92 ± 0.01 |  | 91 | 17 | 0 | 74 | yes |  |  |  | blast | uuaugaucacagucggauuuccgcc | cggaaauccggcuguggccaugc | uuaugaucacagucggauuuccgccacaagaagggcggaaauccggcuguggccaugc | manual\_scaffold\_12:48436439..48436497:+ |
| manual\_scaffold\_1b\_2955 | 2.4 | 0.92 ± 0.01 |  | 48 | 17 | 0 | 31 | yes |  |  |  | blast | accgccaccagucguaucaug | cggccuggcgguguucugcu | accgccaccagucguaucaugacacaugauacggccuggcgguguucugcu | manual\_scaffold\_1b:112788713..112788764:- |
| manual\_scaffold\_7\_27928 | 2.4 | 0.92 ± 0.01 |  | 9 | 8 | 0 | 1 | yes |  |  |  | blast | uaggacuacacaucccagaauu | ucugggaccugugaucuuguu | ucugggaccugugaucuuguuaguucaauuauaaaauaggacuacacaucccagaauu | manual\_scaffold\_7:1402100642..1402100700:- |
| manual\_scaffold\_1b\_3757 | 2.3 | 0.92 ± 0.01 |  | 47 | 47 | 0 | 0 | yes |  |  |  | blast | uacaacugggcucugucacuuu | agugacagagcccaguugucga | uacaacugggcucugucacuuuagagaaccucuaaagugacagagcccaguugucga | manual\_scaffold\_1b:1170316180..1170316237:- |
| manual\_scaffold\_6\_24032 | 2.3 | 0.92 ± 0.01 |  | 45 | 45 | 0 | 0 | yes |  |  |  | blast | uaguccaaaacucugcuagcc | cuaacagaguuuuuggaacucu | cuaacagaguuuuuggaacucugcccuccgcuuggugugcggaguuaguccaaaacucugcuagcc | manual\_scaffold\_6:1118743395..1118743461:- |
| manual\_scaffold\_7\_26801 | 2.3 | 0.92 ± 0.01 |  | 122 | 122 | 0 | 0 | yes |  |  |  | blast | aucgagagcuccgcacauaa | augugccgaguucuccaccc | aucgagagcuccgcacauaaaaaggcaggucuagccuaucuuuuuaugugccgaguucuccaccc | manual\_scaffold\_7:228419178..228419243:- |
| manual\_scaffold\_3a\_9685 | 2.3 | 0.92 ± 0.01 |  | 25 | 25 | 0 | 0 | yes |  |  |  | blast | ugguggucugggcugccaugcc | cauggcgcccagaccacaaca | ugguggucugggcugccaugccagcgguggcgguaaauaccgccauccagcauggcgcccagaccacaaca | manual\_scaffold\_3a:614412995..614413066:- |
| manual\_scaffold\_7\_24986 | 2.3 | 0.92 ± 0.01 |  | 22 | 22 | 0 | 0 | yes |  |  |  | blast | uaugagauuuguaaaugugacu | ucacauuuacaaaucucauauc | uaugagauuuguaaaugugacuuugcgccuuaaucgauucacauuuacaaaucucauauc | manual\_scaffold\_7:290065389..290065449:+ |
| manual\_scaffold\_6\_24340 | 2.3 | 0.92 ± 0.01 |  | 42 | 42 | 0 | 0 | yes |  |  |  | blast | aagggacauaccagcggcgc | acugcuggugugucacuuuu | acugcuggugugucacuuuuaauaugggaaaaagggacauaccagcggcgc | manual\_scaffold\_6:1567617008..1567617059:- |
| manual\_scaffold\_10\_34657 | 2.3 | 0.92 ± 0.01 |  | 29 | 28 | 0 | 1 | yes |  |  |  | blast | cacgcuaguagaucgcugccagc | guucugcuggcgggcgcu | cacgcuaguagaucgcugccagccguauuaugagaaauauuacagccuggagguguucugcuggcgggcgcu | manual\_scaffold\_10:106390172..106390244:- |
| manual\_scaffold\_1a\_930 | 2.3 | 0.92 ± 0.01 |  | 111 | 111 | 0 | 0 | yes |  |  |  | blast | agcggcagcagaauccucucga | gaguggauuugcugccgcuca | gaguggauuugcugccgcucaaguagauuuucuccuugagcggcagcagaauccucucga | manual\_scaffold\_1a:19068560..19068620:- |
| manual\_scaffold\_9\_31838 | 2.3 | 0.92 ± 0.01 |  | 97 | 52 | 0 | 45 | yes |  |  |  | blast | auucacacccagacugaacacc | uucagucugggugugaauauc | auucacacccagacugaacaccuccugguguucagucugggugugaauauc | manual\_scaffold\_9:903715024..903715075:+ |
| manual\_scaffold\_12\_38346 | 2.3 | 0.92 ± 0.01 |  | 73 | 73 | 0 | 0 | yes |  |  |  | blast | acgggcugcacagcacaagugc | gcuucugcuguaaagcccauac | acgggcugcacagcacaagugcagacugcgcuucugcuguaaagcccauac | manual\_scaffold\_12:513581116..513581167:+ |
| manual\_scaffold\_4a\_14590 | 2.3 | 0.92 ± 0.01 |  | 30 | 28 | 0 | 2 | yes |  |  |  | blast | uauucugaagcggagcaaugcu | agcauugcuccgcuucagaau | agcauugcuccgcuucagaauagcuacuggagcuauucugaagcggagcaaugcu | manual\_scaffold\_4a:705871355..705871410:- |
| manual\_scaffold\_9\_32131 | 2.3 | 0.92 ± 0.01 |  | 15 | 15 | 0 | 0 | yes |  |  |  | blast | cccagaagaacuuagaggcag | gccucuaaguucuucugggua | gccucuaaguucuucuggguagccgacuuacccagaagaacuuagaggcag | manual\_scaffold\_9:1167173083..1167173134:+ |
| manual\_scaffold\_4b\_17174 | 2.3 | 0.92 ± 0.01 |  | 45 | 45 | 0 | 0 | yes |  |  |  | blast | cuuggagacugggagccugugc | acaggcucccagccugcccugcg | acaggcucccagccugcccugcguuaguccugaugcugcuuggagacugggagccugugc | manual\_scaffold\_4b:904024238..904024298:- |
| manual\_scaffold\_6\_21459 | 2.3 | 0.92 ± 0.01 |  | 94 | 94 | 0 | 0 | yes |  |  |  | blast | ugggagaagcaccuggauugg | aaucugggugcuuuucucaug | aaucugggugcuuuucucaugcuggguaacagcaugggagaagcaccuggauugg | manual\_scaffold\_6:640615601..640615656:+ |
| manual\_scaffold\_9\_31150 | 2.2 | 0.92 ± 0.01 |  | 14 | 14 | 0 | 0 | yes |  |  |  | blast | aacucugugcguggcgccccu | gggugcugcggcccacaguuug | aacucugugcguggcgccccugucuuguuuuaauggcgcacaggggugcugcggcccacaguuug | manual\_scaffold\_9:287345250..287345315:+ |
| manual\_scaffold\_3b\_11681 | 2.2 | 0.92 ± 0.01 |  | 15 | 15 | 0 | 0 | yes |  |  |  | blast | uggcgggacuagcagaauuuuu | aaccuuuguuagcuccaccagc | uggcgggacuagcagaauuuuuggcaacucugcacucccaguggagcguggagugccaaaaccuuuguuagcuccaccagc | manual\_scaffold\_3b:598453724..598453805:- |
| manual\_scaffold\_8\_29518 | 2.2 | 0.92 ± 0.01 |  | 35 | 35 | 0 | 0 | yes |  |  |  | blast | agccccguugcgcauucuacug | guggaaugugcgaccugu | agccccguugcgcauucuacugucugaauuacaggcaguggaaugugcgaccugu | manual\_scaffold\_8:1402564217..1402564272:+ |
| manual\_scaffold\_9\_32989 | 2.2 | 0.92 ± 0.01 |  | 98 | 53 | 0 | 45 | yes |  |  |  | blast | auucacacccagacugaacacc | uucagucugggugugaauauc | auucacacccagacugaacaccaggagguguucagucugggugugaauauc | manual\_scaffold\_9:903715021..903715072:- |
| manual\_scaffold\_7\_25053 | 2.2 | 0.92 ± 0.01 |  | 41 | 41 | 0 | 0 | yes |  |  |  | blast | uggccaaggugacaugcacacu | ugugcaugucaggccggccauc | ugugcaugucaggccggccaucacgagacagauggccaaggugacaugcacacu | manual\_scaffold\_7:398369415..398369469:+ |
| manual\_scaffold\_4b\_17009 | 2.2 | 0.92 ± 0.01 |  | 107 | 107 | 0 | 0 | yes |  |  |  | blast | gucucgccgucggcacca | gugcagaaggcgagacca | gucucgccgucggcaccaguugcucagugcagaaggcgagacca | manual\_scaffold\_4b:671790286..671790330:- |
| manual\_scaffold\_7\_26147 | 2.2 | 0.92 ± 0.01 |  | 17 | 15 | 0 | 2 | yes |  |  |  | blast | ucucuaucaguugacuggcu | uuccagucagcugauagaca | uuccagucagcugauagacaaagaccauaaaacuaauccacuuucucuaucaguugacuggcu | manual\_scaffold\_7:1402053951..1402054014:+ |
| manual\_scaffold\_5\_19939 | 2.2 | 0.92 ± 0.01 |  | 13 | 13 | 0 | 0 | yes |  |  |  | blast | ccuagcacucuucuuaugcugc | agcauaaaagcagugcuuggau | ccuagcacucuucuuaugcugcuuaguauuauaugcagcauaaaagcagugcuuggau | manual\_scaffold\_5:1042376941..1042376999:- |
| manual\_scaffold\_7\_26655 | 2.2 | 0.92 ± 0.01 |  | 27 | 18 | 0 | 9 | yes |  |  |  | blast | aaacagacgucuagcacugagu | aguguuagaggucuguuucuauc | aaacagacgucuagcacugagucaccggggcacucaguguuagaggucuguuucuauc | manual\_scaffold\_7:123442903..123442961:- |
| manual\_scaffold\_9\_31990 | 2.2 | 0.92 ± 0.01 |  | 27 | 9 | 0 | 18 | yes |  |  |  | blast | aguguucacguaguuuuggccg | uggccaaacugacaugcgcacu | aguguucacguaguuuuggccggccgucuuaggguggccaaacugacaugcgcacu | manual\_scaffold\_9:1062194179..1062194235:+ |
| manual\_scaffold\_11\_35846 | 2.2 | 0.92 ± 0.01 |  | 18 | 16 | 0 | 2 | yes |  |  |  | blast | aggauaucccauucacuguauu | uacgguggacgggauaucc | aggauaucccauucacuguauuacaauuuccaucggcuagaauggcaucguaauacgguggacgggauaucc | manual\_scaffold\_11:3636373..3636445:+ |
| manual\_scaffold\_2a\_5151 | 2.2 | 0.92 ± 0.01 |  | 243 | 224 | 0 | 19 | yes |  |  |  | blast | acaugcgugguuaaggcacaga | ugccuuaaacacgcaugugcug | ugccuuaaacacgcaugugcugaacaacgcacaugcgugguuaaggcacaga | manual\_scaffold\_2a:320316546..320316598:- |
| manual\_scaffold\_4a\_13622 | 2.2 | 0.92 ± 0.01 |  | 2 | 1 | 0 | 1 | yes |  |  |  | blast | cgcauguguguuuggucagcc | ccggccaagcauaugugcuc | cgcauguguguuuggucagcccgagacggccggccaagcauaugugcuc | manual\_scaffold\_4a:37010226..37010275:- |
| manual\_scaffold\_7\_24681 | 2.2 | 0.92 ± 0.01 |  | 12 | 12 | 0 | 0 | yes |  |  |  | blast | augucccuguggagcuaacggua | ccguuagcuccacagggacaucc | ccguuagcuccacagggacauccuggugguggcuagaugucccuguggagcuaacggua | manual\_scaffold\_7:58809994..58810053:+ |
| manual\_scaffold\_9\_31556 | 2.2 | 0.92 ± 0.01 |  | 37 | 20 | 0 | 17 | yes |  |  |  | blast | uucgaccaacacagugagugac | cuguguuggucgaaugcgucu | uucgaccaacacagugagugaccuauagaauaggucacucacuguguuggucgaaugcgucu | manual\_scaffold\_9:766140151..766140213:+ |
| manual\_scaffold\_2a\_5269 | 2.2 | 0.92 ± 0.01 |  | 66 | 66 | 0 | 0 | yes |  |  |  | blast | uggaccgccauuccguuggug | ccgcuggcauggcggucauua | uggaccgccauuccguuggugggaaugaccgccaccgcuggcauggcggucauua | manual\_scaffold\_2a:510444284..510444339:- |
| manual\_scaffold\_7\_28008 | 2.2 | 0.92 ± 0.01 |  | 364 | 364 | 0 | 0 | yes |  |  |  | blast | auaaaucugccaucaaugccu | gcauugaugguagguauguau | gcauugaugguagguauguauuauuauuauauauauaaaucugccaucaaugccu | manual\_scaffold\_7:1438231999..1438232054:- |
| manual\_scaffold\_1b\_2382 | 2.2 | 0.92 ± 0.01 |  | 16 | 16 | 0 | 0 | yes |  |  |  | blast | accuccaugucagcaggacuu | guccugcuggcaugguggggc | accuccaugucagcaggacuuauuacaagccauaauacaccuuggcggaguccugcuggcaugguggggc | manual\_scaffold\_1b:710660510..710660580:+ |
| manual\_scaffold\_4b\_15980 | 2.1 | 0.92 ± 0.01 |  | 59 | 59 | 0 | 0 | yes |  |  |  | blast | gucuccuggcgguuugacugc | agucguaccaccaagacug | gucuccuggcgguuugacugcuaggaucauaaucuggcagucguaccaccaagacug | manual\_scaffold\_4b:1127964243..1127964300:+ |
| manual\_scaffold\_3a\_9468 | 2.1 | 0.92 ± 0.01 |  | 13 | 10 | 0 | 3 | yes |  |  |  | blast | agcauaaagcagugcuaggauu | caggcacugcucuuaugcugc | caggcacugcucuuaugcugcauauaauacuaagcagcauaaagcagugcuaggauu | manual\_scaffold\_3a:454356048..454356105:- |
| manual\_scaffold\_5\_20030 | 2.1 | 0.92 ± 0.01 |  | 19 | 19 | 0 | 0 | yes |  |  |  | blast | aacccagcggucgaaucuggcc | cuggucuccacugcugggucga | cuggucuccacugcugggucgaaagugccuaccgucaacccagcggucgaaucuggcc | manual\_scaffold\_5:1158241812..1158241870:- |
| manual\_scaffold\_11\_36465 | 2.1 | 0.92 ± 0.01 |  | 7 | 7 | 0 | 0 | yes |  | xtr-miR-199a-3p |  | blast | uacaguagagaugauguac | auauaaucuccuccguuua | auauaaucuccuccguuuagcuggccauaggcuauacaguagagaugauguac | manual\_scaffold\_11:772854520..772854573:+ |
| manual\_scaffold\_5\_20385 | 2.1 | 0.92 ± 0.01 |  | 66 | 66 | 0 | 0 | yes |  |  |  | blast | cggugccuuaaucacacaugug | caugugucauuaaggcacagaa | cggugccuuaaucacacaugugcugaaccacgcacaugugucauuaaggcacagaa | manual\_scaffold\_5:1625459600..1625459656:- |
| manual\_scaffold\_4a\_13129 | 2.1 | 0.92 ± 0.01 |  | 44 | 44 | 0 | 0 | yes |  |  |  | blast | agcgccggggacaugucugccu | gacagcagaugccgcggacaugu | gacagcagaugccgcggacauguucagaggcugagcgagcuggacagcgccggggacaugucugccu | manual\_scaffold\_4a:544594091..544594158:+ |
| manual\_scaffold\_4a\_13226 | 2.1 | 0.92 ± 0.01 |  | 35 | 35 | 0 | 0 | yes |  |  |  | blast | agccccguugcgcauucuacug | guggaaugcgcgacgggugcugc | agccccguugcgcauucuacuguccaaaugugcgaauuacgggccguggaaugcgcgacgggugcugc | manual\_scaffold\_4a:651584411..651584479:+ |
| manual\_scaffold\_1a\_1748 | 2.1 | 0.92 ± 0.01 |  | 18 | 18 | 0 | 0 | yes |  |  |  | blast | ucacccugacggaugguagucc | acuaccauccaucaggguggug | acuaccauccaucaggguggugcacuugaguccucaucacccugacggaugguagucc | manual\_scaffold\_1a:1100963500..1100963558:- |
| manual\_scaffold\_10\_33772 | 2.1 | 0.92 ± 0.01 |  | 51 | 51 | 0 | 0 | yes |  |  |  | blast | cagaggacagagcacacugugu | acaugugcucuguauccucugcc | cagaggacagagcacacuguguguauguauuuccacaugugcucuguauccucugcc | manual\_scaffold\_10:290676761..290676818:+ |
| manual\_scaffold\_1b\_3502 | 2.1 | 0.92 ± 0.01 |  | 65 | 65 | 0 | 0 | yes |  |  |  | blast | ugcggcaaacggggaacuuacu | gaaguucccugcuugccgcagc | gaaguucccugcuugccgcagccaggccgagcugcggcaaacggggaacuuacu | manual\_scaffold\_1b:826622463..826622517:- |
| manual\_scaffold\_11\_36518 | 2.1 | 0.92 ± 0.01 |  | 42 | 42 | 0 | 0 | yes |  |  |  | blast | cucagcacugcucucuugc | aaaagagcagugccaggau | cucagcacugcucucuugcugccuuuauuaguaaacagcaaaagagcagugccaggau | manual\_scaffold\_11:837979319..837979377:+ |
| manual\_scaffold\_8\_30471 | 2.1 | 0.92 ± 0.01 |  | 14 | 14 | 0 | 0 | yes |  |  |  | blast | uuucacaucucgucacagggug | cccugugacaaguuaugaaagg | uuucacaucucgucacagggugggguuuggucagugaagcuugguggccccgccucacccugugacaaguuaugaaagg | manual\_scaffold\_8:1068994843..1068994922:- |
| manual\_scaffold\_7\_27480 | 2.1 | 0.92 ± 0.01 |  | 79 | 77 | 2 | 0 | yes |  |  |  | blast | ccggggggacaaaaaaaa | auuucugccccccgggg | auuucugccccccgggggcagauuggcccaauauuauuaggccgaucugcccccggggggacaaaaaaaa | manual\_scaffold\_7:962488724..962488794:- |
| manual\_scaffold\_7\_25785 | 2.1 | 0.92 ± 0.01 |  | 12 | 12 | 0 | 0 | yes |  |  |  | blast | acacgaucugcucagaugcacu | cacgucugagcaggcugugucu | cacgucugagcaggcugugucuggucuucagacacgaucugcucagaugcacu | manual\_scaffold\_7:1119483961..1119484014:+ |
| manual\_scaffold\_12\_39437 | 2.1 | 0.92 ± 0.01 |  | 22 | 22 | 0 | 0 | yes |  |  |  | blast | cuucugggcauuucugcagccu | gcugcggaaaugcucagagagac | cuucugggcauuucugcagccugauugcuucaugaucgggcugcggaaaugcucagagagac | manual\_scaffold\_12:538286632..538286694:- |
| manual\_scaffold\_4a\_14151 | 2.1 | 0.92 ± 0.01 |  | 121 | 115 | 0 | 6 | yes |  |  |  | blast | cuggcacugcucuuaugcu | agcaugagaacagugccaggau | cuggcacugcucuuaugcuaaaccuagcaugagaacagugccaggau | manual\_scaffold\_4a:138353596..138353643:- |
| manual\_scaffold\_5\_18274 | 2.1 | 0.92 ± 0.01 |  | 34 | 34 | 0 | 0 | yes |  |  |  | blast | aggggguagagcacuguu | caguucugcccccuug | aggggguagagcacuguugggcaguucugcccccuug | manual\_scaffold\_5:857391733..857391770:+ |
| manual\_scaffold\_5\_18420 | 2.1 | 0.92 ± 0.01 |  | 103 | 103 | 0 | 0 | yes |  |  |  | blast | cuagcacugcucuuaugcuguu | cagcaagagagcagugcuuagau | cuagcacugcucuuaugcuguuuggugcuguaagcagcaagagagcagugcuuagau | manual\_scaffold\_5:1046308045..1046308102:+ |
| manual\_scaffold\_5\_20555 | 2.1 | 0.92 ± 0.01 |  | 23 | 23 | 0 | 0 | yes |  |  |  | blast | cucuguuugacagugggaccu | gucccacugucaaacaggcac | cucuguuugacagugggaccugcuuuaaaggucccacugucaaacaggcac | manual\_scaffold\_5:1885600460..1885600511:- |
| manual\_scaffold\_9\_32153 | 2.1 | 0.92 ± 0.01 |  | 12 | 9 | 0 | 3 | no |  |  |  | blast | uugucccggccugugguaagac | uuucccacagacugggccaauac | uuucccacagacugggccaauacuuauuuacaauacuguuugucccggccugugguaagac | manual\_scaffold\_9:1195081396..1195081457:+ |
| manual\_scaffold\_5\_18724 | 2.1 | 0.92 ± 0.01 |  | 67 | 67 | 0 | 0 | yes |  |  |  | blast | uggaccgccauuccguuggug | ccgccggcauggcaguccaga | uggaccgccauuccguuggugguaaugaccgccaccgccggcauggcaguccaga | manual\_scaffold\_5:1465970512..1465970567:+ |
| manual\_scaffold\_12\_38736 | 2.0 | 0.92 ± 0.01 |  | 414 | 412 | 2 | 0 | yes |  |  |  | blast | ucccgguggcucugcgcaugc | cugcgcaugcucccgguggc | cugcgcaugcucccgguggcucugcgcaugcucccgguggcucugcucugcgcaugcucccguuggcucugcgcaugcucccgguggcucugcgcaugc | manual\_scaffold\_12:683918106..683918205:+ |
| manual\_scaffold\_5\_19428 | 2.0 | 0.92 ± 0.01 |  | 87 | 78 | 0 | 9 | yes |  |  |  | blast | auacgaguacugaaggauuucu | ugaaaucuuccaguagucgugc | auacgaguacugaaggauuucugcugcaauuugagcugaaaucuuccaguagucgugc | manual\_scaffold\_5:362068963..362069021:- |
| manual\_scaffold\_3a\_9930 | 2.0 | 0.92 ± 0.01 |  | 12 | 11 | 0 | 1 | no |  |  |  | blast | uaccaucuugcccaaaggcugu | ucuccuuuggaguuagaugacu | ucuccuuuggaguuagaugacuagugaccaugcaauacuaccaucuugcccaaaggcugu | manual\_scaffold\_3a:910869823..910869883:- |
| manual\_scaffold\_1b\_3715 | 2.0 | 0.92 ± 0.01 |  | 103 | 103 | 0 | 0 | yes |  |  |  | blast | ccaccacguucccguggg | agcgggaaagugauaaug | ccaccacguucccguggggcugacggguggaaaccuuuguuuccaucgucagcccagcgggaaagugauaaug | manual\_scaffold\_1b:1115507461..1115507534:- |
| manual\_scaffold\_4b\_17082 | 2.0 | 0.92 ± 0.01 |  | 20 | 17 | 0 | 3 | yes |  |  |  | blast | uuucgugaugagaggaacagggaa | uuccccguuccucucauuac | uuccccguuccucucauuacgaaauuagguauucaagccuaauuucgugaugagaggaacagggaa | manual\_scaffold\_4b:748760733..748760799:- |
| manual\_scaffold\_9\_32936 | 2.0 | 0.92 ± 0.01 |  | 15 | 15 | 0 | 0 | yes |  |  |  | blast | auuguaucuuucagcuccuggc | caguagcugaaggauaaaauga | caguagcugaaggauaaaaugauaaugaaauauaauuguaucuuucagcuccuggc | manual\_scaffold\_9:839312282..839312338:- |
| manual\_scaffold\_1a\_532 | 2.0 | 0.92 ± 0.01 |  | 142 | 142 | 0 | 0 | yes |  |  |  | blast | uggaccucgagaagcgugaca | ucgcacuacugcgagguccaua | uggaccucgagaagcgugacagggguugucgcacuacugcgagguccaua | manual\_scaffold\_1a:659651025..659651075:+ |
| manual\_scaffold\_7\_27532 | 2.0 | 0.92 ± 0.01 |  | 21 | 21 | 0 | 0 | yes |  |  |  | blast | ugggacugagagcacugc | agcggcucagccacagc | agcggcucagccacagcuccugggacugagagcacugc | manual\_scaffold\_7:1031545923..1031545961:- |
| manual\_scaffold\_8\_30071 | 2.0 | 0.92 ± 0.01 |  | 34 | 17 | 0 | 17 | yes |  |  |  | blast | accgccaccagucguaucaug | auacggccuggcaguguucugc | accgccaccagucguaucaugacccaugauacggccuggcaguguucugc | manual\_scaffold\_8:455401032..455401082:- |
| manual\_scaffold\_7\_27178 | 2.0 | 0.92 ± 0.01 |  | 15 | 15 | 0 | 0 | yes |  |  |  | blast | auggacccagcaucaggauugu | aauccugacgcugguuucaugc | aauccugacgcugguuucaugcuguuaccagcauggacccagcaucaggauugu | manual\_scaffold\_7:644689460..644689514:- |
| manual\_scaffold\_10\_35794 | 2.0 | 0.92 ± 0.01 |  | 11 | 11 | 0 | 0 | yes |  |  |  | blast | uugcacuguguagccuauagccu | guuaugggccacacagugcaugc | guuaugggccacacagugcaugcgcagacagucugcuuuugcacuguguagccuauagccu | manual\_scaffold\_10:1051910387..1051910448:- |
| manual\_scaffold\_3a\_8423 | 2.0 | 0.92 ± 0.01 |  | 102 | 102 | 0 | 0 | yes |  |  |  | blast | uuuccgagcugugcugucuggc | ugacagcacaguucggaaaca | ugacagcacaguucggaaacaguaagagcgucucggucuguuuccgagcugugcugucuggc | manual\_scaffold\_3a:712106704..712106766:+ |
| manual\_scaffold\_9\_33131 | 2.0 | 0.92 ± 0.01 |  | 28 | 27 | 0 | 1 | yes |  |  |  | blast | uguuuuaaccaugcuugugcuu | augcgugguuaaggcacag | uguuuuaaccaugcuugugcuuuguaccuuaacuacgcauaugcgugguuaaggcacag | manual\_scaffold\_9:1075076424..1075076483:- |
| manual\_scaffold\_12\_38334 | 2.0 | 0.92 ± 0.01 |  | 20 | 20 | 0 | 0 | yes |  |  |  | blast | acgaaacugccgucgacggug | ccgucgacggggcugucgucg | acgaaacugccgucgacggugccaaugucgagaaaguuuccgucgacggggcugucgucg | manual\_scaffold\_12:501176156..501176216:+ |
| manual\_scaffold\_10\_35605 | 2.0 | 0.92 ± 0.01 |  | 12 | 12 | 0 | 0 | yes |  |  |  | blast | uggcaucuguagcgucaguguu | cacuggcgcuacagaugccaca | uggcaucuguagcgucaguguuuuggugacuuuucaccaaaaaaacacuggcgcuacagaugccaca | manual\_scaffold\_10:958380433..958380500:- |
| manual\_scaffold\_9\_31506 | 2.0 | 0.92 ± 0.01 |  | 31 | 31 | 0 | 0 | yes |  |  |  | blast | ugcucguugggaguagcagcc | cuguugcuccaaacgugcggg | ugcucguugggaguagcagccagauucuucagaccggcuguugcuccaaacgugcggg | manual\_scaffold\_9:710437526..710437584:+ |
| manual\_scaffold\_2b\_6648 | 2.0 | 0.92 ± 0.01 |  | 12 | 12 | 0 | 0 | yes |  |  |  | blast | ugccggaauugugagagccu | gcucucucaagccuggcaac | gcucucucaagccuggcaacuguguugccggaauugugagagccu | manual\_scaffold\_2b:162203328..162203373:- |
| manual\_scaffold\_2b\_6445 | 2.0 | 0.92 ± 0.01 |  | 19 | 19 | 0 | 0 | yes |  |  |  | blast | ugaagguaguguaaaccgacu | uugguuuacaccaccuuuauc | uugguuuacaccaccuuuaucaggacaugcagaugaagguaguguaaaccgacu | manual\_scaffold\_2b:696710357..696710411:+ |
| manual\_scaffold\_3a\_8407 | 2.0 | 0.92 ± 0.01 |  | 21 | 21 | 0 | 0 | yes |  |  |  | blast | ucgaauagguugauggugagg | ucuccaucaaccuauucguau | ucuccaucaaccuauucguauuugaaggagccaauucgaauagguugauggugagg | manual\_scaffold\_3a:685682056..685682112:+ |
| manual\_scaffold\_2a\_5508 | 2.0 | 0.92 ± 0.01 |  | 14 | 14 | 0 | 0 | yes |  |  |  | blast | cguugagggaacccggcgccu | ccgccggguuuccucaacguc | ccgccggguuuccucaacgucaguuauuuugacacguugagggaacccggcgccu | manual\_scaffold\_2a:796288816..796288871:- |
| manual\_scaffold\_4a\_14229 | 2.0 | 0.92 ± 0.01 |  | 140 | 140 | 0 | 0 | yes |  |  |  | blast | uacccauuugaacugcugcaa | gcagcaguucagcuggguaua | uacccauuugaacugcugcaaaacugugccuaaccaugcagcaguucagcuggguaua | manual\_scaffold\_4a:239992579..239992637:- |
| manual\_scaffold\_3b\_10767 | 2.0 | 0.92 ± 0.01 |  | 36 | 36 | 0 | 0 | yes |  |  |  | blast | agugccaacauguaguccuauu | uaggaguaaauguuggcacugg | uaggaguaaauguuggcacuggucagugcagugccaacauguaguccuauu | manual\_scaffold\_3b:861099160..861099211:+ |
| manual\_scaffold\_6\_24160 | 2.0 | 0.92 ± 0.01 |  | 17 | 17 | 0 | 0 | yes |  |  |  | blast | ucugagcuugcaucuguugga | caagcagaugcaggguucagaua | caagcagaugcaggguucagauaaucaguuaaagucugagcuugcaucuguugga | manual\_scaffold\_6:1312890457..1312890512:- |
| manual\_scaffold\_5\_18922 | 2.0 | 0.92 ± 0.01 |  | 65 | 65 | 0 | 0 | yes |  |  |  | blast | auccuggcacugcucauaugcu | caugaaaguauugccaggauug | auccuggcacugcucauaugcuaaaucuagcaugaaaguauugccaggauug | manual\_scaffold\_5:1718988267..1718988319:+ |
| manual\_scaffold\_1b\_3780 | 2.0 | 0.92 ± 0.01 |  | 30 | 30 | 0 | 0 | yes |  |  |  | blast | caugaaagcagcgacuggauu | uccaggcgcugcucucaugcu | uccaggcgcugcucucaugcugacuaacaacaugaaagcagcgacuggauu | manual\_scaffold\_1b:1197342063..1197342114:- |
| manual\_scaffold\_12\_38735 | 2.0 | 0.92 ± 0.01 |  | 425 | 425 | 0 | 0 | yes |  |  |  | blast | ucccgguggcucugcgcaugc | cugcgcaugcucccguuggc | cugcgcaugcucccguuggcucugcgcaugcucccguuggcucugcgcaugcucccgguggcucugcgcaugc | manual\_scaffold\_12:683918064..683918137:+ |
| manual\_scaffold\_1b\_3800 | 2.0 | 0.92 ± 0.01 |  | 14 | 14 | 0 | 0 | yes |  |  |  | blast | uugcaccacuguaccuuuagc | ucaaggugcaguggcccagug | ucaaggugcaguggcccaguguaaggauacuuucauugcaccacuguaccuuuagc | manual\_scaffold\_1b:1225734814..1225734870:- |
| manual\_scaffold\_4b\_17065 | 2.0 | 0.92 ± 0.01 |  | 17 | 17 | 0 | 0 | yes |  |  |  | blast | accgccaccagucguaucaug | ugauacgaccuggcaguguucu | accgccaccagucguaucaugacacaugauacgaccuggcaguguucu | manual\_scaffold\_4b:728283451..728283499:- |
| manual\_scaffold\_7\_26663 | 1.9 | 0.91 ± 0.01 |  | 32 | 31 | 0 | 1 | yes |  |  |  | blast | caugggcugacggaccugagau | uucucagguuggacaguccuu | caugggcugacggaccugagauugacagucacaucucaccauucucagguuggacaguccuu | manual\_scaffold\_7:127312967..127313029:- |
| manual\_scaffold\_12\_39062 | 1.9 | 0.91 ± 0.01 |  | 18 | 18 | 0 | 0 | yes |  |  |  | blast | aaaaucggacuccaguaacaaa | uguuacugggggccaacauugc | uguuacugggggccaacauugcagcacggcugcaaaaucggacuccaguaacaaa | manual\_scaffold\_12:85675893..85675948:- |
| manual\_scaffold\_12\_39151 | 1.9 | 0.91 ± 0.01 |  | 28 | 28 | 0 | 0 | yes |  |  |  | blast | uuuguuuugugacucgcgauu | uugcgaguugcaaaacaaaag | uuuguuuugugacucgcgauuugcgauucccaaauggauugcaaauugcgaguugcaaaacaaaag | manual\_scaffold\_12:188224104..188224170:- |
| manual\_scaffold\_1a\_1123 | 1.9 | 0.91 ± 0.01 |  | 394 | 393 | 0 | 1 | yes |  |  |  | blast | uugggacacuugccaggacu | agugcugagagguguccuuga | uugggacacuugccaggacuccuguauuaugucagcagugcugagagguguccuuga | manual\_scaffold\_1a:311206730..311206787:- |
| manual\_scaffold\_7\_24777 | 1.9 | 0.91 ± 0.01 |  | 265 | 265 | 0 | 0 | yes |  |  |  | blast | uuuugucuuucaucugcuggcu | ccaggaggugaaaaaugaaacg | ccaggaggugaaaaaugaaacgauauugaauuaucguuuugucuuucaucugcuggcu | manual\_scaffold\_7:96416463..96416521:+ |
| manual\_scaffold\_12\_38271 | 1.9 | 0.91 ± 0.01 |  | 51 | 51 | 0 | 0 | yes |  |  |  | blast | ggggauguaacucagugg | auaaagguccuccu | auaaagguccuccugcuggcaagugcagugagugacugcacauggccgcagggggauguaacucagugg | manual\_scaffold\_12:445469221..445469290:+ |
| manual\_scaffold\_7\_27494 | 1.9 | 0.91 ± 0.01 |  | 14 | 14 | 0 | 0 | yes |  |  |  | blast | ccgaaaccaucucugcuacuga | aguagcagagauaguuucagca | aguagcagagauaguuucagcaguauaauguacucugccgaaaccaucucugcuacuga | manual\_scaffold\_7:978580290..978580349:- |
| manual\_scaffold\_8\_28590 | 1.9 | 0.91 ± 0.01 |  | 18 | 18 | 0 | 0 | yes |  |  |  | blast | ugguccugcucucaugcugcu | cagcauaagugaagugucagg | ugguccugcucucaugcugcuaaacagcauaagugaagugucagg | manual\_scaffold\_8:96573507..96573552:+ |
| manual\_scaffold\_1a\_390 | 1.9 | 0.91 ± 0.01 |  | 42 | 38 | 0 | 4 | yes |  |  |  | blast | uuccccugaugcuguauugccu | uggaguauagugucaguggagc | uuccccugaugcuguauugccuauagguggauauauggaguauagugucaguggagc | manual\_scaffold\_1a:446126335..446126392:+ |
| manual\_scaffold\_4a\_14141 | 1.9 | 0.91 ± 0.01 |  | 1889 | 1889 | 0 | 0 | yes |  |  |  | blast | agcgucucggaccaaccgccu | gugguuggaaacgaagcgcgcg | agcgucucggaccaaccgccuugugaagaaaacuucaggugguuggaaacgaagcgcgcg | manual\_scaffold\_4a:131392923..131392983:- |
| manual\_scaffold\_6\_22235 | 1.9 | 0.91 ± 0.01 |  | 16 | 16 | 0 | 0 | yes |  |  |  | blast | cuugggaauggagagaccucu | gggucccuguauucccaagcu | gggucccuguauucccaagcuggagccuuagcuuuacagcuugggaauggagagaccucu | manual\_scaffold\_6:1428829828..1428829888:+ |
| manual\_scaffold\_2b\_7089 | 1.9 | 0.91 ± 0.01 |  | 129 | 127 | 0 | 2 | yes |  |  |  | blast | acagggccccacuggcacccu | agguguccaucaggcccugcgu | acagggccccacuggcacccucuaugugcauuggaccagagguguccaucaggcccugcgu | manual\_scaffold\_2b:704273666..704273727:- |
| manual\_scaffold\_1a\_811 | 1.9 | 0.91 ± 0.01 |  | 24 | 20 | 4 | 0 | yes |  |  |  | blast | acgaaacugccgucgacggug | ccgucaacggcacccccaucg | ccgucaacggcacccccaucgacgacagccccgucgacggaaacuuugucgacauugcgagcgucgacgaaacugccgucgacggug | manual\_scaffold\_1a:1054316768..1054316855:+ |
| manual\_scaffold\_2a\_5462 | 1.9 | 0.91 ± 0.01 |  | 66 | 64 | 0 | 2 | yes |  |  |  | blast | auguagcagcaucuggauuggu | auccaggcgcuucuuucaugc | auccaggcgcuucuuucaugcuaguuaacagcauguagcagcaucuggauuggu | manual\_scaffold\_2a:706703893..706703947:- |
| manual\_scaffold\_12\_38737 | 1.9 | 0.91 ± 0.01 |  | 427 | 425 | 2 | 0 | yes |  |  |  | blast | ucccgguggcucugcgcaugc | cugcgcaugcucccguuggc | ucccgguggcucugcgcaugcucccguuggcucugcgcaugcucccgguggcucugugcaugcucccgguggcucugcgcaugcucccguuggc | manual\_scaffold\_12:683918205..683918299:+ |
| manual\_scaffold\_3a\_7588 | 1.9 | 0.91 ± 0.01 |  | 131 | 131 | 0 | 0 | yes |  |  |  | blast | uaacaguaggagauaaugugcu | cacauuaauucuuacuguuccg | uaacaguaggagauaaugugcugauaauuuuuauuucacagcacauuaauucuuacuguuccg | manual\_scaffold\_3a:314918100..314918163:+ |
| manual\_scaffold\_4b\_17126 | 1.9 | 0.91 ± 0.01 |  | 21 | 21 | 0 | 0 | yes |  |  |  | blast | ugcccgggccacccucucuccu | gugagaggguggcuccaaggcugu | gugagaggguggcuccaaggcugucgauggugugcuuucugcccgggccacccucucuccu | manual\_scaffold\_4b:841825275..841825336:- |
| manual\_scaffold\_8\_29372 | 1.9 | 0.91 ± 0.01 |  | 16 | 16 | 0 | 0 | yes |  |  |  | blast | ucggggugucugcugccuggacu | uccaggugggucaccccuagu | ucggggugucugcugccuggacuaacaugaacuaucuuuguccaggugggucaccccuagu | manual\_scaffold\_8:1217843692..1217843753:+ |
| manual\_scaffold\_10\_34290 | 1.9 | 0.91 ± 0.01 |  | 17357 | 9574 | 0 | 7783 | yes |  |  |  | blast | cagagagcaggcucugugacc | ugucauggaguugcucucaaga | cagagagcaggcucugugaccagaauauucucaaaaccuugucauggaguugcucucaaga | manual\_scaffold\_10:998517453..998517514:+ |
| manual\_scaffold\_1b\_2036 | 1.9 | 0.91 ± 0.01 |  | 20 | 19 | 0 | 1 | yes |  |  |  | blast | ugguccugcucucaugcugcu | ugggagcagcaucuggauugg | ugguccugcucucaugcugcuuaacguuaucagcagcaugggagcagcaucuggauugg | manual\_scaffold\_1b:277611513..277611572:+ |
| manual\_scaffold\_1b\_2650 | 1.9 | 0.91 ± 0.01 |  | 75 | 75 | 0 | 0 | yes |  |  |  | blast | aucuagacacuguucucaugcu | caugggagaagugccuggguug | aucuagacacuguucucaugcuguucaccagcaugggagaagugccuggguug | manual\_scaffold\_1b:1020135601..1020135654:+ |
| manual\_scaffold\_11\_36590 | 1.9 | 0.91 ± 0.01 |  | 242 | 223 | 0 | 19 | yes |  |  |  | blast | acaugcgugguuaaggcacaga | ugccuuaaacacgcaugugcug | ugccuuaaacacgcaugugcugauccauguacaugcgugguuaaggcacaga | manual\_scaffold\_11:909540400..909540452:+ |
| manual\_scaffold\_9\_33171 | 1.9 | 0.91 ± 0.01 |  | 19 | 19 | 0 | 0 | yes |  |  |  | blast | uuucucguucuccuggcgcccgu | gcgcggacagagaacgggaaagu | uuucucguucuccuggcgcccguuucgaauuaaacgcgcggacagagaacgggaaagu | manual\_scaffold\_9:1114376837..1114376895:- |
| manual\_scaffold\_2a\_5692 | 1.9 | 0.91 ± 0.01 |  | 16 | 16 | 0 | 0 | yes |  |  |  | blast | uggcgucacuacaggagccu | gcucucaaugugaaggccaca | gcucucaaugugaaggccacacguauguuugaguguggcgucacuacaggagccu | manual\_scaffold\_2a:1150387018..1150387073:- |
| manual\_scaffold\_7\_25394 | 1.9 | 0.91 ± 0.01 |  | 20 | 18 | 0 | 2 | yes |  |  |  | blast | ucugugccuuaaucacccaugu | auaugugugguuaaggcacaag | ucugugccuuaaucacccauguacugaacuacgcauaugugugguuaaggcacaag | manual\_scaffold\_7:752030225..752030281:+ |
| manual\_scaffold\_5\_19430 | 1.9 | 0.91 ± 0.01 |  | 285 | 285 | 0 | 0 | yes |  |  |  | blast | ucugagcgcgacauagcuacu | uagcuauguagcucuuaua | uagcuauguagcucuuauauaauaaauaaaaaucugagcgcgacauagcuacu | manual\_scaffold\_5:363101366..363101419:- |
| manual\_scaffold\_5\_19414 | 1.9 | 0.91 ± 0.01 |  | 41 | 41 | 0 | 0 | yes |  |  |  | blast | uggccaaggugacaugcacacu | agugcauaugucccuuuggcuggc | agugcauaugucccuuuggcuggcuguuuugugcuggccaaggugacaugcacacu | manual\_scaffold\_5:338089182..338089238:- |
| manual\_scaffold\_2a\_5594 | 1.9 | 0.91 ± 0.01 |  | 13 | 13 | 0 | 0 | yes |  |  |  | blast | uggacuggccguguucugcuggu | caguugaacgccgccagcugua | caguugaacgccgccagcuguaucaugaccuguuauauggacuggccguguucugcuggu | manual\_scaffold\_2a:947002613..947002673:- |
| manual\_scaffold\_3b\_11732 | 1.9 | 0.91 ± 0.01 |  | 40 | 40 | 0 | 0 | yes |  |  |  | blast | cagcaugagagcaaugugagc | aagcauugcugucaugcuugu | aagcauugcugucaugcuuguuauacuagcaaacagcaugagagcaaugugagc | manual\_scaffold\_3b:660472255..660472309:- |
| manual\_scaffold\_10\_34446 | 1.9 | 0.91 ± 0.01 |  | 16 | 16 | 0 | 0 | yes |  |  |  | blast | acguggaaaccugcagaggu | cucugcauggcucuacaagu | cucugcauggcucuacaaguaaguguaacacacuuacacguggaaaccugcagaggu | manual\_scaffold\_10:15420077..15420134:- |
| manual\_scaffold\_3b\_10855 | 1.9 | 0.91 ± 0.01 |  | 17 | 16 | 0 | 1 | no |  |  |  | blast | ucagucuacauccgcuucuuacu | ugaguagcaguguaccugugg | ugaguagcaguguaccugugggcaugacaguaaaacaaccucagucuacauccgcuucuuacu | manual\_scaffold\_3b:978327935..978327998:+ |
| manual\_scaffold\_6\_22834 | 1.9 | 0.91 ± 0.01 |  | 145 | 145 | 0 | 0 | yes |  |  |  | blast | caggacugccuuucacuguucc | acagugagaggugguccagug | acagugagaggugguccaguggcuuuuaacaacaggacugccuuucacuguucc | manual\_scaffold\_6:118537371..118537425:- |
| manual\_scaffold\_3b\_11719 | 1.9 | 0.91 ± 0.01 |  | 85 | 85 | 0 | 0 | yes |  |  |  | blast | gagaggacacagaacacacugu | ggugugcucugugcccucagcc | gagaggacacagaacacacuguguguguguguuuccaggugugcucugugcccucagcc | manual\_scaffold\_3b:624474306..624474365:- |
| manual\_scaffold\_10\_33324 | 1.9 | 0.91 ± 0.01 |  | 25 | 25 | 0 | 0 | yes |  |  |  | blast | cugugaugaugacuugcu | uguucugugaugaugacacgc | cugugaugaugacuugcucccuucucuugagggacaggcaggucucagggagaggggaguguucugugaugaugacacgc | manual\_scaffold\_10:15784168..15784248:+ |
| manual\_scaffold\_5\_18955 | 1.9 | 0.91 ± 0.01 |  | 183 | 181 | 0 | 2 | yes |  |  |  | blast | cacggacauguacuggcagaca | ucccugccaguacacgucuguuu | ucccugccaguacacgucuguuuguguguuuguguauacacacacggacauguacuggcagaca | manual\_scaffold\_5:1766159010..1766159074:+ |
| manual\_scaffold\_4b\_16725 | 1.9 | 0.91 ± 0.01 |  | 2976 | 2976 | 0 | 0 | yes |  |  |  | blast | cuggcacugcucuuaugcuguu | cagcaugagagcaggguuuaaa | cuggcacugcucuuaugcuguuuaguauuuugggcagcaugagagcaggguuuaaa | manual\_scaffold\_4b:245168780..245168836:- |
| manual\_scaffold\_7\_24692 | 1.9 | 0.91 ± 0.01 |  | 16 | 16 | 0 | 0 | yes |  |  |  | blast | cuagcacugcucuuauaaugcu | cagcauaagagcagugcuuaga | cuagcacugcucuuauaaugcuuaguaauauaugcagcauaagagcagugcuuaga | manual\_scaffold\_7:63867592..63867648:+ |
| manual\_scaffold\_4a\_13091 | 1.9 | 0.91 ± 0.01 |  | 6148 | 6024 | 0 | 124 | yes |  |  |  | blast | uuauugcuuaagaauacgcgu | acggguauucuuggguggauaaua | acggguauucuuggguggauaauacggauuacguuguuauugcuuaagaauacgcgu | manual\_scaffold\_4a:497936440..497936497:+ |
| manual\_scaffold\_8\_30465 | 1.9 | 0.91 ± 0.01 |  | 32 | 32 | 0 | 0 | yes |  |  |  | blast | ugugacaccugucaagacugcu | cagcaccgagaggugucucagg | ugugacaccugucaagacugcugguacaaucagcagcaccgagaggugucucagg | manual\_scaffold\_8:1060789714..1060789769:- |
| manual\_scaffold\_8\_28763 | 1.9 | 0.91 ± 0.01 |  | 77 | 77 | 0 | 0 | yes |  |  |  | blast | gguaggucggaguuugugacccauu | uggguugcaaaucgaccuaacuc | uggguugcaaaucgaccuaacucuugaguauuaaugagguaggucggaguuugugacccauu | manual\_scaffold\_8:269864369..269864431:+ |
| manual\_scaffold\_2b\_6565 | 1.9 | 0.91 ± 0.01 |  | 222 | 222 | 0 | 0 | yes |  |  |  | blast | auugagacacaagacauucaggcu | ccugagucaugggucucaauga | auugagacacaagacauucaggcugugaaccugauauuucagccugagucaugggucucaauga | manual\_scaffold\_2b:63958435..63958499:- |
| manual\_scaffold\_10\_35379 | 1.9 | 0.91 ± 0.01 |  | 30 | 30 | 0 | 0 | yes |  |  |  | blast | uuuguuuugugacucgcgauu | uugcgagucacaaaacaaaag | uuuguuuugugacucgcgauuugcaaacggaucgcaaauugcgagucacaaaacaaaag | manual\_scaffold\_10:712718161..712718220:- |
| manual\_scaffold\_3b\_10291 | 1.9 | 0.91 ± 0.01 |  | 21 | 20 | 0 | 1 | yes |  |  |  | blast | auaugguucuuuccugcucucu | agagagcaggauagugccauau | agagagcaggauagugccauaugucuguagauaugguucuuuccugcucucu | manual\_scaffold\_3b:336602485..336602537:+ |
| manual\_scaffold\_9\_32472 | 1.9 | 0.91 ± 0.01 |  | 41 | 41 | 0 | 0 | yes |  |  |  | blast | uggccaaggugacaugcacacu | ugagcauguugcuuuggccagc | ugagcauguugcuuuggccagcugucuugcgauggccaaggugacaugcacacu | manual\_scaffold\_9:293763548..293763602:- |
| manual\_scaffold\_6\_23860 | 1.9 | 0.91 ± 0.01 |  | 12 | 12 | 0 | 0 | yes |  |  |  | blast | uagcacgguuccacaguauaau | gguacugugguacugugcaagc | gguacugugguacugugcaagcguagucaggaaagcuagcacgguuccacaguauaau | manual\_scaffold\_6:924917015..924917073:- |
| manual\_scaffold\_9\_32233 | 1.9 | 0.91 ± 0.01 |  | 35 | 35 | 0 | 0 | yes |  |  |  | blast | uggaacuuguaguuaauggcauc | ugcgggccuacaagucccagg | uggaacuuguaguuaauggcauccuguuaaagaugcgggccuacaagucccagg | manual\_scaffold\_9:46625610..46625664:- |
| manual\_scaffold\_5\_18672 | 1.9 | 0.91 ± 0.01 |  | 531 | 531 | 0 | 0 | yes |  |  |  | blast | cuggcccuacuccgaugcuguu | cagcaugagaguagugcuuaca | cuggcccuacuccgaugcuguuuaguauuauaggcagcaugagaguagugcuuaca | manual\_scaffold\_5:1386906495..1386906551:+ |
| manual\_scaffold\_6\_22790 | 1.9 | 0.91 ± 0.01 |  | 12 | 12 | 0 | 0 | yes |  |  |  | blast | acggagcagcgccuggauugga | caaucccggcguugcucucauguug | caaucccggcguugcucucauguuguuuaaacaacggagcagcgccuggauugga | manual\_scaffold\_6:102088552..102088607:- |
| manual\_scaffold\_4a\_14175 | 1.9 | 0.91 ± 0.01 |  | 23 | 16 | 0 | 7 | yes |  |  |  | blast | cggccaaacagacaugcgcacu | agugugcuugacuguuuggcc | agugugcuugacuguuuggccgacuguguuggaccggccaaacagacaugcgcacu | manual\_scaffold\_4a:159840915..159840971:- |
| manual\_scaffold\_5\_17566 | 1.9 | 0.91 ± 0.01 |  | 34 | 34 | 0 | 0 | yes |  |  |  | blast | uuugaauuguaugacuuguagu | uacaaguccaacaauuuaaagg | uuugaauuguaugacuuguaguuuuuauuuaguuccauuauaaaacagggcuacaaguccaacaauuuaaagg | manual\_scaffold\_5:29486653..29486726:+ |
| manual\_scaffold\_4b\_15622 | 1.9 | 0.91 ± 0.01 |  | 134 | 134 | 0 | 0 | yes |  |  |  | blast | uguucucuuacucugugcacu | cacacagaguaagaaaauuga | uguucucuuacucugugcacugagaagcuggcuagacacacagaguaagaaaauuga | manual\_scaffold\_4b:758040111..758040168:+ |
| manual\_scaffold\_10\_35735 | 1.8 | 0.91 ± 0.01 |  | 32 | 32 | 0 | 0 | yes |  |  |  | blast | uugagagcaacuccaugacaag | ggucacagagccugcucucuggg | uugagagcaacuccaugacaagguuuugagaauauucuggucacagagccugcucucuggg | manual\_scaffold\_10:998517451..998517512:- |
| manual\_scaffold\_1a\_1749 | 1.8 | 0.91 ± 0.01 |  | 77 | 77 | 0 | 0 | yes |  |  |  | blast | ucccgagcucuggaugacuccc | gaguucccagcgcuuggggga | ucccgagcucuggaugacucccuggagccucuggaguucccagcgcuuggggga | manual\_scaffold\_1a:1101014492..1101014546:- |
| manual\_scaffold\_8\_30741 | 1.8 | 0.91 ± 0.01 |  | 18 | 18 | 0 | 0 | yes |  |  |  | blast | ucuucguucugucuaaaugga | caguuagauagaaugaaaaaa | ucuucguucugucuaaauggauuacauuuuagaauccaguuagauagaaugaaaaaa | manual\_scaffold\_8:1459684426..1459684483:- |
| manual\_scaffold\_2a\_4436 | 1.8 | 0.91 ± 0.01 |  | 514 | 514 | 0 | 0 | yes |  |  |  | blast | uuugcucccucuaaugccu | guaugagagagagcaugcc | uuugcucccucuaaugccugccuauucauggguugguaugagagagagcaugcc | manual\_scaffold\_2a:772850893..772850947:+ |
| manual\_scaffold\_10\_34121 | 1.8 | 0.91 ± 0.01 |  | 13 | 13 | 0 | 0 | yes |  |  |  | blast | ugcugagucucugucugccg | gcagacagcugaaagcagcagg | gcagacagcugaaagcagcaggguuucuaguuucccugcugagucucugucugccg | manual\_scaffold\_10:748342283..748342339:+ |
| manual\_scaffold\_1b\_2035 | 1.8 | 0.91 ± 0.01 |  | 19 | 19 | 0 | 0 | yes |  |  |  | blast | ugguccugcucucaugcugcu | gagcauugaggcagccgauccagc | gagcauugaggcagccgauccagccaacccugguccugcucucaugcugcu | manual\_scaffold\_1b:277611483..277611534:+ |
| manual\_scaffold\_9\_32593 | 1.8 | 0.91 ± 0.01 |  | 345 | 345 | 0 | 0 | yes |  |  |  | blast | agcccgccgccgaccaugcuga | aguggcggcggcagacau | agcccgccgccgaccaugcugaugguugacagaagaccgccaguggcggcggcagacau | manual\_scaffold\_9:461359159..461359218:- |
| manual\_scaffold\_7\_26904 | 1.8 | 0.91 ± 0.01 |  | 19 | 19 | 0 | 0 | yes |  |  |  | blast | uuccuguuacggcuguccacc | cagacagccauaacagggauu | cagacagccauaacagggauuuaagauucucauacuuuuucuaggcauuuccuguuacggcuguccacc | manual\_scaffold\_7:349541948..349542017:- |
| manual\_scaffold\_7\_27641 | 1.8 | 0.91 ± 0.01 |  | 19 | 19 | 0 | 0 | yes |  |  |  | blast | uugaucgcagcgugauugc | aaucgcucugcgaacaaaa | uugaucgcagcgugauugcgcuauguaaaccccagcgcaaucgcucugcgaacaaaa | manual\_scaffold\_7:1129203263..1129203320:- |
| manual\_scaffold\_11\_36706 | 1.8 | 0.91 ± 0.01 |  | 45 | 45 | 0 | 0 | yes |  |  |  | blast | acggcuuuccagacgcgagacg | ccucggccaggaaggccuug | acggcuuuccagacgcgagacgaguaaagcguguuucucgccucggccaggaaggccuug | manual\_scaffold\_11:1012828743..1012828803:+ |
| manual\_scaffold\_11\_36149 | 1.8 | 0.91 ± 0.01 |  | 219 | 203 | 0 | 16 | yes |  |  |  | blast | uggcacugcucucaugcug | caccaugggaguagcgccuaga | uggcacugcucucaugcuguuuuuguaacauaucaccaugggaguagcgccuaga | manual\_scaffold\_11:344876626..344876681:+ |
| manual\_scaffold\_11\_35853 | 1.8 | 0.91 ± 0.01 |  | 704 | 699 | 0 | 5 | yes |  |  |  | blast | uuugaauucugggacuuguaguc | auucccagaauucaaagacg | uuugaauucugggacuuguaguccuguuuuaaaaugaaacaagacuauaauucccagaauucaaagacg | manual\_scaffold\_11:11951923..11951992:+ |
| manual\_scaffold\_9\_30903 | 1.8 | 0.91 ± 0.01 |  | 28 | 28 | 0 | 0 | yes |  |  |  | blast | uggcccccagaacuuuuucu | agaggagaucugggggccagu | agaggagaucugggggccaguggguauauuuggacaguuaccuggcccccagaacuuuuucu | manual\_scaffold\_9:50100725..50100787:+ |
| manual\_scaffold\_1a\_331 | 1.8 | 0.91 ± 0.01 |  | 33 | 33 | 0 | 0 | yes |  |  |  | blast | cagggguccgcagaccugggcu | ccugugguccguggacccccaggg | ccugugguccguggacccccagggguccgugacacauucccagggguccgcagaccugggcu | manual\_scaffold\_1a:394058125..394058187:+ |
| manual\_scaffold\_9\_32607 | 1.8 | 0.91 ± 0.01 |  | 146 | 125 | 0 | 21 | yes |  |  |  | blast | caugagagcagcaccuggau | auccaguucuugcucucuugc | auccaguucuugcucucuugcugcuaaguuaccaacacagcaugagagcagcaccuggau | manual\_scaffold\_9:480335482..480335542:- |
| manual\_scaffold\_8\_30046 | 1.8 | 0.91 ± 0.01 |  | 13 | 13 | 0 | 0 | yes |  |  |  | blast | cagucgagacacaacaaacagc | uguuugcuguggcuuggcagca | uguuugcuguggcuuggcagcagcugucaaagcugcagucgagacacaacaaacagc | manual\_scaffold\_8:407060630..407060687:- |
| manual\_scaffold\_4b\_15477 | 1.8 | 0.91 ± 0.01 |  | 23 | 23 | 0 | 0 | yes |  |  |  | blast | ugucgaagacgagaccggcuc | gcuggccucgcccucggccaca | gcuggccucgcccucggccacaguugccuugugucgaagacgagaccggcuc | manual\_scaffold\_4b:608774918..608774970:+ |
| manual\_scaffold\_6\_21358 | 1.8 | 0.91 ± 0.01 |  | 17 | 17 | 0 | 0 | yes |  |  |  | blast | cuagcacugcuuuuauguugc | agcauaagagcugugcuugaa | cuagcacugcuuuuauguugcuuauuauuauauucagcauaagagcugugcuugaa | manual\_scaffold\_6:525847441..525847497:+ |
| manual\_scaffold\_1a\_290 | 1.8 | 0.91 ± 0.01 |  | 41 | 15 | 0 | 26 | yes |  |  |  | blast | uccuucuuugggccgcuagc | cuugguggccuuaaagaaggagg | cuugguggccuuaaagaaggaggaagaggaauuggcaucuccuucuuugggccgcuagc | manual\_scaffold\_1a:353169794..353169853:+ |
| manual\_scaffold\_3b\_11452 | 1.8 | 0.91 ± 0.01 |  | 56 | 56 | 0 | 0 | yes |  |  |  | blast | cucccuguggaucccugugccu | gugcagggaaccaccagggugaa | gugcagggaaccaccagggugaaguguagucagcaccugcuucucccuguggaucccugugccu | manual\_scaffold\_3b:470959453..470959517:- |
| manual\_scaffold\_6\_22086 | 1.8 | 0.91 ± 0.01 |  | 902 | 902 | 0 | 0 | yes |  |  |  | blast | cuggcacugcucuuaugcug | gcaugagagcggcguucagau | cuggcacugcucuuaugcugcuaacauuaucagcagcaugagagcggcguucagau | manual\_scaffold\_6:1288375736..1288375792:+ |
| manual\_scaffold\_8\_29229 | 1.8 | 0.91 ± 0.01 |  | 12 | 12 | 0 | 0 | yes |  |  |  | blast | accggaaaaucagcacgaacag | gcuugugcugguuuuuagugc | gcuugugcugguuuuuagugccgggaucucguucggcaccggaaaaucagcacgaacag | manual\_scaffold\_8:1028162936..1028162995:+ |
| manual\_scaffold\_7\_28257 | 1.8 | 0.91 ± 0.01 |  | 17 | 12 | 0 | 5 | yes |  |  |  | blast | gcugcaauuugucguaccu | agguagggcgaauugcaaccc | gcugcaauuugucguaccucauuaauguuuuugagguagggcgaauugcaaccc | manual\_scaffold\_7:1523943450..1523943504:- |
| manual\_scaffold\_6\_24360 | 1.8 | 0.91 ± 0.01 |  | 12 | 12 | 0 | 0 | yes |  |  |  | blast | uacgagaccccugcacacuucu | auaggugugcaggaccagcgugug | auaggugugcaggaccagcguguggcauuuaaagguugcauacgagaccccugcacacuucu | manual\_scaffold\_6:1587586862..1587586924:- |
| manual\_scaffold\_3a\_9760 | 1.8 | 0.91 ± 0.01 |  | 24 | 22 | 2 | 0 | yes |  |  |  | blast | uuuccgaacugugcugucagca | ccagacagcacagcucggaaaca | ccagacagcacagcucggaaacagaccgagacgcucuuacuguuuccgaacugugcugucagca | manual\_scaffold\_3a:712106701..712106765:- |
| manual\_scaffold\_1b\_1880 | 1.8 | 0.91 ± 0.01 |  | 181 | 172 | 0 | 9 | yes |  |  |  | blast | caaaucagccuugcgucacuu | aagugacgcauggcugcuuugc | aagugacgcauggcugcuuugcagcacauaaagcaaggcaaaucagccuugcgucacuu | manual\_scaffold\_1b:90757006..90757065:+ |
| manual\_scaffold\_2b\_6650 | 1.8 | 0.91 ± 0.01 |  | 21 | 21 | 0 | 0 | yes |  |  |  | blast | cuaagguggacaggauaucugu | agauaucccaucugcuguauua | agauaucccaucugcuguauuaccauccccauaggauagaaugggauugugcuaagguggacaggauaucugu | manual\_scaffold\_2b:170959939..170960012:- |
| manual\_scaffold\_8\_28932 | 1.8 | 0.91 ± 0.01 |  | 14 | 14 | 0 | 0 | yes |  |  |  | blast | ucugacuuggcgagaacuguc | aagcgcccgccaaaucagagc | ucugacuuggcgagaacugucaaagcgcccgccaaaucagagc | manual\_scaffold\_8:485410867..485410910:+ |
| manual\_scaffold\_1a\_1349 | 1.8 | 0.91 ± 0.01 |  | 37 | 37 | 0 | 0 | yes |  |  |  | blast | ccuggcacuguucuuauccugu | agcaugacaucagugccuggau | ccuggcacuguucuuauccuguuugguauuaaaaacagcaugacaucagugccuggau | manual\_scaffold\_1a:605439539..605439597:- |
| manual\_scaffold\_9\_31780 | 1.8 | 0.91 ± 0.01 |  | 79 | 79 | 0 | 0 | yes |  |  |  | blast | uggagacacuugccggaacucc | agugccgggagguguccugga | uggagacacuugccggaacucccguauuacaucagcagugccgggagguguccugga | manual\_scaffold\_9:866363361..866363418:+ |
| manual\_scaffold\_4b\_16227 | 1.8 | 0.91 ± 0.01 |  | 13 | 13 | 0 | 0 | yes |  |  |  | blast | aacucugugcguggcgccccu | uggugccacagcgcacaguuug | aacucugugcguggcgccccuggcuuguuuuggcggcgcacaguggugccacagcgcacaguuug | manual\_scaffold\_4b:77997111..77997176:- |
| manual\_scaffold\_10\_35073 | 1.8 | 0.91 ± 0.01 |  | 79 | 79 | 0 | 0 | yes |  |  |  | blast | gcgaggauccuggaugcuguuu | acagcgccuggcucccgucug | gcgaggauccuggaugcuguuugcaagagaauauccaaacagcgccuggcucccgucug | manual\_scaffold\_10:404234699..404234758:- |
| manual\_scaffold\_8\_29950 | 1.8 | 0.91 ± 0.01 |  | 39 | 39 | 0 | 0 | yes |  |  |  | blast | ccggggggaccaaaaacc | auuuugccccccuggg | auuuugccccccugggccagaucggcugauuuuucggccgaucugcccccggggggaccaaaaacc | manual\_scaffold\_8:315695952..315696018:- |
| manual\_scaffold\_11\_37244 | 1.8 | 0.91 ± 0.01 |  | 19 | 19 | 0 | 0 | yes |  |  |  | blast | uuuuaucuuuccgcuccagacu | ccugcagcugaaagagaaaacg | ccugcagcugaaagagaaaacgauaaugaaauauuguuuuaucuuuccgcuccagacu | manual\_scaffold\_11:444559460..444559518:- |
| manual\_scaffold\_12\_38734 | 1.8 | 0.91 ± 0.01 |  | 425 | 425 | 0 | 0 | yes |  |  |  | blast | ucccgguggcucugcgcaugc | cugcgcaugcucccguuggc | ucccgguggcucugcgcaugcucccgguggcucuccucugcacaugcucccguuggcucugcgcaugcucccguuggc | manual\_scaffold\_12:683917985..683918063:+ |
| manual\_scaffold\_4b\_16617 | 1.8 | 0.91 ± 0.01 |  | 13 | 13 | 0 | 0 | yes |  |  |  | blast | uagggccgcuggagcaugcgc | ucgggcuccagcagcccucuu | uagggccgcuggagcaugcgcgauagccgcuuuacugcagaugcucgggcuccagcagcccucuu | manual\_scaffold\_4b:161348689..161348754:- |
| manual\_scaffold\_6\_23740 | 1.8 | 0.91 ± 0.01 |  | 56 | 56 | 0 | 0 | yes |  |  |  | blast | uuacacugcaguaaaggguacc | cgccacuuuacagcaguguaaca | cgccacuuuacagcaguguaacagugcucauugccauuguuacacugcaguaaaggguacc | manual\_scaffold\_6:789654562..789654623:- |
| manual\_scaffold\_10\_34163 | 1.8 | 0.91 ± 0.01 |  | 128 | 128 | 0 | 0 | yes |  |  |  | blast | acucugucgcaccaucugugga | cacagaggugugucaucgaugucc | cacagaggugugucaucgauguccgugacacccaacucggacucugucgcaccaucugugga | manual\_scaffold\_10:818151690..818151752:+ |
| manual\_scaffold\_1b\_2381 | 1.8 | 0.91 ± 0.01 |  | 16 | 16 | 0 | 0 | yes |  |  |  | blast | accuccaugucagcaggacuu | guccuggcgauaggcggugc | guccuggcgauaggcggugcagaagcgguuccaucgcuggcaccuccaugucagcaggacuu | manual\_scaffold\_1b:710660469..710660531:+ |
| manual\_scaffold\_11\_36069 | 1.8 | 0.91 ± 0.01 |  | 210 | 193 | 0 | 17 | yes |  |  |  | blast | uagcagaugcaggguucagau | gucugagcacgcgucuguugga | uagcagaugcaggguucagauaaucaguuaaagucugagcacgcgucuguugga | manual\_scaffold\_11:249944084..249944138:+ |
| manual\_scaffold\_1a\_1449 | 1.8 | 0.91 ± 0.01 |  | 204 | 204 | 0 | 0 | yes |  |  |  | blast | uucugggacauguaggcccgcuu | guggucuaccaguccuagaauu | uucugggacauguaggcccgcuugcccaauauaaaaguggucuaccaguccuagaauu | manual\_scaffold\_1a:748719571..748719629:- |
| manual\_scaffold\_2b\_6885 | 1.8 | 0.91 ± 0.01 |  | 33 | 33 | 0 | 0 | yes |  |  |  | blast | aauaccucgaugucuucaggcg | ccugagagcaccgagguugagc | aauaccucgaugucuucaggcgggaucuaaauucaccugagagcaccgagguugagc | manual\_scaffold\_2b:464612873..464612930:- |
| manual\_scaffold\_11\_36000 | 1.8 | 0.91 ± 0.01 |  | 19 | 19 | 0 | 0 | yes |  |  |  | blast | gccggcgugacggagcaggacu | ucuggucuguugcagucuggcac | gccggcgugacggagcaggacugccaaacacauaaugaccaccauuaugugcaagugcagucuggucuguugcagucuggcac | manual\_scaffold\_11:152800410..152800493:+ |
| manual\_scaffold\_1a\_660 | 1.8 | 0.91 ± 0.01 |  | 142 | 142 | 0 | 0 | yes |  |  |  | blast | uggaacaccuguugggguuuc | agcgccgagagguguuccagg | uggaacaccuguugggguuucuguguccaccaggaagcgccgagagguguuccagg | manual\_scaffold\_1a:829978474..829978530:+ |
| manual\_scaffold\_3b\_10217 | 1.7 | 0.91 ± 0.01 |  | 44 | 44 | 0 | 0 | yes |  |  |  | blast | ugggacacaagacuauacucu | aguauagucuugugucccacc | aguauagucuugugucccaccaacaugggacacaagacuauacucu | manual\_scaffold\_3b:249051213..249051259:+ |
| manual\_scaffold\_5\_19756 | 1.7 | 0.91 ± 0.01 |  | 12 | 12 | 0 | 0 | yes |  |  |  | blast | uaggguuauggaacuccgaggu | cgagcaguuccauaaacauaua | cgagcaguuccauaaacauauagaggaaagaggccgaaggcugaguucuuuuuuaggguuauggaacuccgaggu | manual\_scaffold\_5:771251881..771251956:- |
| manual\_scaffold\_4b\_15710 | 1.7 | 0.91 ± 0.01 |  | 68 | 68 | 0 | 0 | yes |  |  |  | blast | uuaccaauucgucacagggu | ccugugacgaguuggugaaag | uuaccaauucgucacagggugugguaccauucaauccugugacgaguuggugaaag | manual\_scaffold\_4b:897896838..897896894:+ |
| manual\_scaffold\_6\_21104 | 1.7 | 0.91 ± 0.01 |  | 36 | 36 | 0 | 0 | yes |  |  |  | blast | ucacagacaacauccugccaugu | auggcaggguggaguugugggc | ucacagacaacauccugccaugugguauugcaacaaacauggcaggguggaguugugggc | manual\_scaffold\_6:349949856..349949916:+ |
| manual\_scaffold\_10\_33427 | 1.7 | 0.91 ± 0.01 |  | 63 | 63 | 0 | 0 | yes |  |  |  | blast | uuaguacaucacgcuugcacgu | ggcaagaguggugugcuacgc | ggcaagaguggugugcuacgcugugcucuacaauuggugcuuaguacaucacgcuugcacgu | manual\_scaffold\_10:48071248..48071310:+ |
| manual\_scaffold\_5\_19833 | 1.7 | 0.91 ± 0.01 |  | 30 | 30 | 0 | 0 | yes |  |  |  | blast | acgcggauggcaguucagcugc | aguuggaauuccgucugcuua | acgcggauggcaguucagcugccauacaucucaauaaggcaguuggaauuccgucugcuua | manual\_scaffold\_5:858273807..858273868:- |
| manual\_scaffold\_4b\_15075 | 1.7 | 0.91 ± 0.01 |  | 29 | 29 | 0 | 0 | yes |  |  |  | blast | ucagugagcaucgaacaagga | cuuguuggucacucauuggcugagu | cuuguuggucacucauuggcugaguaguuaauucagugagcaucgaacaagga | manual\_scaffold\_4b:186045548..186045601:+ |
| manual\_scaffold\_3b\_11027 | 1.7 | 0.91 ± 0.01 |  | 381 | 381 | 0 | 0 | yes |  |  |  | blast | uuugaaacacucacgcauggacc | cacugcgugaguggcucacaca | cacugcgugaguggcucacacauggacacgguacuguuugaaacacucacgcauggacc | manual\_scaffold\_3b:1164692259..1164692318:+ |
| manual\_scaffold\_9\_31743 | 1.7 | 0.91 ± 0.01 |  | 79 | 79 | 0 | 0 | yes |  |  |  | blast | aagaggagugcuaggaag | ucugagacuuugcuugu | ucugagacuuugcuuguugauugcugugcccucaacaucaagaggagugcuaggaag | manual\_scaffold\_9:842607156..842607213:+ |
| manual\_scaffold\_1a\_375 | 1.7 | 0.91 ± 0.01 |  | 14 | 14 | 0 | 0 | yes |  |  |  | blast | uccaaccagacaugugugcuuu | ggugcacaucggguuggaca | ggugcacaucggguuggacaggucuguuauuguguccaaccagacaugugugcuuu | manual\_scaffold\_1a:428097874..428097930:+ |
| manual\_scaffold\_6\_24316 | 1.7 | 0.91 ± 0.01 |  | 1138 | 1124 | 0 | 14 | yes |  |  |  | blast | uagcggaugcaggguucagau | gucugaccacgcaucuguugga | uagcggaugcaggguucagauaaucaguuaaggucugaccacgcaucuguugga | manual\_scaffold\_6:1515354161..1515354215:- |
| manual\_scaffold\_1b\_1935 | 1.7 | 0.91 ± 0.01 |  | 43 | 38 | 0 | 5 | yes |  |  |  | blast | ucuaagugguguggaguugccu | aggcauuuccagcuaguuagga | aggcauuuccagcuaguuaggagucaugcugacagcuucuaagugguguggaguugccu | manual\_scaffold\_1b:171795298..171795357:+ |
| manual\_scaffold\_4b\_17407 | 1.7 | 0.91 ± 0.01 |  | 26 | 26 | 0 | 0 | yes |  |  |  | blast | uuugggacacuugucaggacu | cacugagaaguguccuggaag | uuugggacacuugucaggacucucauauuaugucaacagcacugagaaguguccuggaag | manual\_scaffold\_4b:1148948107..1148948167:- |
| manual\_scaffold\_12\_38945 | 1.7 | 0.91 ± 0.01 |  | 107 | 107 | 0 | 0 | yes |  |  |  | blast | uggauugcauggaaccugccu | gaaaguugcaauuugcgauccauu | uggauugcauggaaccugccucauuaaaauucacgaggaaaguugcaauuugcgauccauu | manual\_scaffold\_12:28339979..28340040:- |
| manual\_scaffold\_7\_27147 | 1.7 | 0.91 ± 0.01 |  | 729 | 729 | 0 | 0 | yes |  |  |  | blast | uuuaucagaccuagugaaaacu | aauucaccaggugugauaagua | uuuaucagaccuagugaaaacugauacacaaaagcuuggaauucaccaggugugauaagua | manual\_scaffold\_7:609297225..609297286:- |
| manual\_scaffold\_1a\_280 | 1.7 | 0.91 ± 0.01 |  | 133 | 127 | 0 | 6 | yes |  |  |  | blast | cuaacacuaccccuaugcugu | caugagagcagugcuuggau | cuaacacuaccccuaugcuguuuagcauuguaagcagcaugagagcagugcuuggau | manual\_scaffold\_1a:349828844..349828901:+ |
| manual\_scaffold\_9\_32772 | 1.7 | 0.91 ± 0.01 |  | 32 | 32 | 0 | 0 | yes |  |  |  | blast | agccaaagugacaugcgaacu | ugcgcaugucacuuuggaugg | ugcgcaugucacuuuggauggcuguuuugcaucagccaaagugacaugcgaacu | manual\_scaffold\_9:709750941..709750995:- |
| manual\_scaffold\_8\_29692 | 1.7 | 0.91 ± 0.01 |  | 19 | 16 | 0 | 3 | yes |  |  |  | blast | ucugggacuugaagucauguuu | ucaagucccagaauuccaa | ucugggacuugaagucauguuuuuauaacggaauaagcaagacuucaagucccagaauuccaa | manual\_scaffold\_8:32066566..32066629:- |
| manual\_scaffold\_5\_18973 | 1.7 | 0.91 ± 0.01 |  | 176471 | 174990 | 0 | 1481 | yes |  |  |  | blast | uaaggcacgcggugaaugcc | cguguucacagcggaccuugau | cguguucacagcggaccuugauuuaaauguccauacaauuaaggcacgcggugaaugcc | manual\_scaffold\_5:1790270357..1790270416:+ |
| manual\_scaffold\_4a\_14797 | 1.7 | 0.91 ± 0.01 |  | 90 | 90 | 0 | 0 | yes |  |  |  | blast | cuagcacugcucuuaugcuguu | cagcaagagagagaugcuuaga | cuagcacugcucuuaugcuguuuagcauuguaagcagcaagagagagaugcuuaga | manual\_scaffold\_4a:891919558..891919614:- |
| manual\_scaffold\_2a\_5285 | 1.7 | 0.91 ± 0.01 |  | 55 | 55 | 0 | 0 | yes |  |  |  | blast | ucgaggacuacaucugccaugu | agggcagauuguaguccugggu | ucgaggacuacaucugccauguggaauugcaacaagcagggcagauuguaguccugggu | manual\_scaffold\_2a:537683069..537683128:- |
| manual\_scaffold\_1b\_3756 | 1.7 | 0.91 ± 0.01 |  | 34 | 34 | 0 | 0 | yes |  |  |  | blast | aaugaugaggcuggugagucu | gcucaucugcucaccauccc | gcucaucugcucaccaucccuuaggaaauuacaacaggaaugaugaggcuggugagucu | manual\_scaffold\_1b:1169998773..1169998832:- |
| manual\_scaffold\_9\_30870 | 1.7 | 0.91 ± 0.01 |  | 26 | 26 | 0 | 0 | yes |  |  |  | blast | ucaccgggcugacaggcagaaac | uucagccggucagcccagugaaa | ucaccgggcugacaggcagaaacaucuuaauaugaugauucagccggucagcccagugaaa | manual\_scaffold\_9:27187193..27187254:+ |
| manual\_scaffold\_4a\_12956 | 1.7 | 0.91 ± 0.01 |  | 26 | 26 | 0 | 0 | yes |  |  |  | blast | guuuccuaugucaagcugaau | ucagcuugacuuagguaauug | guuuccuaugucaagcugaauuguuaauagucaguucagcuugacuuagguaauug | manual\_scaffold\_4a:253403139..253403195:+ |
| manual\_scaffold\_1a\_1591 | 1.7 | 0.91 ± 0.01 |  | 43 | 43 | 0 | 0 | yes |  |  |  | blast | auccuggcgcuucucugaugcu | caugagaacagcacuaggauug | auccuggcgcuucucugaugcugguuaauagcaugagaacagcacuaggauug | manual\_scaffold\_1a:964687876..964687929:- |
| manual\_scaffold\_9\_31833 | 1.7 | 0.91 ± 0.01 |  | 21 | 21 | 0 | 0 | yes |  |  |  | blast | ccugcggauuauagugaugccu | gugucacuguaauccuagcccggcc | gugucacuguaauccuagcccggccuugcacacuggccugcggauuauagugaugccu | manual\_scaffold\_9:900852590..900852648:+ |
| manual\_scaffold\_7\_25759 | 1.7 | 0.91 ± 0.01 |  | 246 | 246 | 0 | 0 | yes |  |  |  | blast | uucggaauuuuuggaaaugc | augucccaaauugcgaauu | augucccaaauugcgaauuccuaacuggaauucggaauuuuuggaaaugc | manual\_scaffold\_7:1098441320..1098441370:+ |
| scaffold\_366\_40022 | 1.7 | 0.91 ± 0.01 |  | 32 | 32 | 0 | 0 | yes |  |  |  | blast | uuaccuucugauugaauacacu | ugucuuugaucauaaggugugg | uuaccuucugauugaauacacugugaauauucuggugucuuugaucauaaggugugg | scaffold\_366:99123..99180:+ |
| manual\_scaffold\_6\_23501 | 1.7 | 0.91 ± 0.01 |  | 369 | 369 | 0 | 0 | yes |  |  |  | blast | cucgggcuagaauuuccaguc | cugacuugaaauuuuagccccagug | cucgggcuagaauuuccagucaccuauccugugacugacuugaaauuuuagccccagug | manual\_scaffold\_6:503221128..503221187:- |
| manual\_scaffold\_6\_21890 | 1.7 | 0.91 ± 0.01 |  | 20 | 20 | 0 | 0 | yes |  |  |  | blast | ucugcaacguguaguccugauu | acaggacuacaagugccagaau | ucugcaacguguaguccugauuuaucccauuaaaaacaggacuacaagugccagaau | manual\_scaffold\_6:1116887421..1116887478:+ |
| manual\_scaffold\_7\_27575 | 1.7 | 0.91 ± 0.01 |  | 15 | 15 | 0 | 0 | yes |  |  |  | blast | caggaggggccaugacucacc | uggguuaugaugcccuucccauu | caggaggggccaugacucaccugugauaaaacuuaguggguuaugaugcccuucccauu | manual\_scaffold\_7:1056712846..1056712905:- |
| manual\_scaffold\_5\_19987 | 1.7 | 0.91 ± 0.01 |  | 15 | 15 | 0 | 0 | yes |  |  |  | blast | ugccgagaugguacucugugccu | ggagagaguccaucugggucua | ugccgagaugguacucugugccugcuccagggcgaaaaagggagagaguccaucugggucua | manual\_scaffold\_5:1108328680..1108328742:- |
| manual\_scaffold\_7\_26055 | 1.7 | 0.91 ± 0.01 |  | 13 | 13 | 0 | 0 | yes |  |  |  | blast | cagcacucuggacucuga | ugagccgugcugcc | ugagccgugcugcccuaguaccaguuagccuggaggcagcacucuggacucuga | manual\_scaffold\_7:1317849485..1317849539:+ |
| manual\_scaffold\_1a\_1613 | 1.7 | 0.91 ± 0.01 |  | 121 | 121 | 0 | 0 | yes |  |  |  | blast | aacggccagucucugcauggcu | ccaugcagugauuggcuaugcu | aacggccagucucugcauggcuucaaaauagagccaugcagugauuggcuaugcu | manual\_scaffold\_1a:982807461..982807516:- |
| manual\_scaffold\_5\_20143 | 1.7 | 0.91 ± 0.01 |  | 14 | 14 | 0 | 0 | yes |  |  |  | blast | ucugacuuggcgagaacuguc | aagcuuccaccaaguuagagc | ucugacuuggcgagaacugucaaagcuuccaccaaguuagagc | manual\_scaffold\_5:1267809596..1267809639:- |
| manual\_scaffold\_3a\_7924 | 1.7 | 0.91 ± 0.01 |  | 12 | 12 | 0 | 0 | yes |  |  |  | blast | gaugcuaggcuguuaacacc | cguuaaccagccuagcauaau | gaugcuaggcuguuaacaccaauauggcguuaaccagccuagcauaau | manual\_scaffold\_3a:493405563..493405611:+ |
| manual\_scaffold\_7\_26294 | 1.7 | 0.91 ± 0.01 |  | 32 | 32 | 0 | 0 | yes |  |  |  | blast | uuaccuucugauugaauacacu | ugucuuugaucauaaggugugg | uuaccuucugauugaauacacugugaauauucuggugucuuugaucauaaggugugg | manual\_scaffold\_7:1467763746..1467763803:+ |
| manual\_scaffold\_10\_34651 | 1.7 | 0.91 ± 0.01 |  | 6056 | 6056 | 0 | 0 | yes |  |  |  | blast | uucuagaaguuguuggauacu | gaccugacuucuucuagauua | uucuagaaguuguuggauacuaggguacuaucacuggaccugacuucuucuagauua | manual\_scaffold\_10:103735590..103735647:- |
| manual\_scaffold\_9\_31814 | 1.7 | 0.91 ± 0.01 |  | 65 | 65 | 0 | 0 | yes |  |  |  | blast | auccuggcacugcucauaugcu | cauuuaagcagcgucaggauug | auccuggcacugcucauaugcuaagcacagcauuuaagcagcgucaggauug | manual\_scaffold\_9:889051977..889052029:+ |
| manual\_scaffold\_2b\_6846 | 1.7 | 0.91 ± 0.01 |  | 12 | 12 | 0 | 0 | yes |  |  |  | blast | cccagagugacaugugcacu | uguguaugucacuuuggcca | uguguaugucacuuuggccagugcaaaacagccucccagagugacaugugcacu | manual\_scaffold\_2b:418099677..418099731:- |
| manual\_scaffold\_12\_39432 | 1.7 | 0.91 ± 0.01 |  | 14 | 14 | 0 | 0 | yes |  |  |  | blast | ucggggaggacagaaacc | uuucggguccccuugggg | uuucggguccccuugggggcagaacggccuauaucaaauuaggccgaacugcucucggggaggacagaaacc | manual\_scaffold\_12:527475858..527475930:- |
| manual\_scaffold\_12\_37897 | 1.7 | 0.91 ± 0.01 |  | 842 | 815 | 0 | 27 | yes |  |  |  | blast | uuugaauucugggacuuguaguc | acaagccguagaauucaauga | uuugaauucugggacuuguagucuuguuucacaguggaauaaagccgacuacaagccguagaauucaauga | manual\_scaffold\_12:55687535..55687606:+ |
| manual\_scaffold\_5\_20125 | 1.7 | 0.91 ± 0.01 |  | 31 | 31 | 0 | 0 | yes |  |  |  | blast | uuugaugcauugcugacguugc | aacuuuagcaaugcaucagcuu | uuugaugcauugcugacguugcgucaguucugacaccaacuuuagcaaugcaucagcuu | manual\_scaffold\_5:1243496692..1243496751:- |
| manual\_scaffold\_2b\_6809 | 1.7 | 0.91 ± 0.01 |  | 21 | 21 | 0 | 0 | yes |  |  |  | blast | aguacccuaccuuucguccuga | aggauguaggguaaggcucugg | aguacccuaccuuucguccugauuuuuagagaugcacucaggauguaggguaaggcucugg | manual\_scaffold\_2b:356077291..356077352:- |
| manual\_scaffold\_9\_32919 | 1.7 | 0.91 ± 0.01 |  | 17 | 11 | 0 | 6 | yes |  |  |  | blast | cuugaauucuaggacuaguagu | cacaagucccugaauucaaaga | cuugaauucuaggacuaguaguccugguaaugggauaaauaagaccacaagucccugaauucaaaga | manual\_scaffold\_9:822488679..822488746:- |
| manual\_scaffold\_7\_26296 | 1.7 | 0.91 ± 0.01 |  | 32 | 32 | 0 | 0 | yes |  |  |  | blast | uuaccuucugauugaauacacu | ugucuuugaucauaaggugugg | uuaccuucugauugaauacacugugaauauucuggugucuuugaucauaaggugugg | manual\_scaffold\_7:1467874216..1467874273:+ |
| manual\_scaffold\_5\_20543 | 1.7 | 0.91 ± 0.01 |  | 35 | 26 | 0 | 9 | yes |  |  |  | blast | ucaaggcagccccuguccagccc | uggcuaugggugcugccuggc | ucaaggcagccccuguccagcccgaguaugugcuauccuggcuaugggugcugccuggc | manual\_scaffold\_5:1855960969..1855961028:- |
| manual\_scaffold\_6\_23628 | 1.7 | 0.91 ± 0.01 |  | 165 | 165 | 0 | 0 | yes |  |  |  | blast | uccauugugccagcucugcaga | ugcagagcaggcuuccgggauc | ugcagagcaggcuuccgggauccuaaggcgauccauugugccagcucugcaga | manual\_scaffold\_6:648736676..648736729:- |
| manual\_scaffold\_8\_30794 | 1.7 | 0.91 ± 0.01 |  | 48564 | 25282 | 0 | 23282 | yes |  |  |  | blast | uggagauacagcccuguugga | ucuacagugcaugugucuccagu | ucuacagugcaugugucuccagugauacugaagcaacuggagauacagcccuguugga | manual\_scaffold\_8:1495326145..1495326203:- |
| manual\_scaffold\_7\_26452 | 1.7 | 0.91 ± 0.01 |  | 132 | 132 | 0 | 0 | yes |  |  |  | blast | ucucucugauccuauaguaugu | auccuauaguaugugagagcag | auccuauaguaugugagagcagauuuccagccuucucucugauccuauaguaugu | manual\_scaffold\_7:1588543460..1588543515:+ |
| manual\_scaffold\_7\_25220 | 1.7 | 0.91 ± 0.01 |  | 93 | 92 | 1 | 0 | yes |  |  |  | blast | gaagcgcuuucaggacugcugu | aguggagcugggaagcgcuuuca | aguggagcugggaagcgcuuucaggacugcugucugcuguugaugugcaguggagcugggaagcgcuuucaggacugcugu | manual\_scaffold\_7:607634369..607634450:+ |
| manual\_scaffold\_1b\_1937 | 1.7 | 0.91 ± 0.01 |  | 43 | 38 | 0 | 5 | yes |  |  |  | blast | ucuaagugguguggaguugccu | aggcauuuccagcuaguuagga | aggcauuuccagcuaguuaggagucaugcugacagcuucuaagugguguggaguugccu | manual\_scaffold\_1b:171798178..171798237:+ |
| manual\_scaffold\_3a\_8357 | 1.7 | 0.91 ± 0.01 |  | 484 | 484 | 0 | 0 | yes |  |  |  | blast | ugcggaccuuaaagauucuucc | aagaaucuuuauuaaggucugcagc | aagaaucuuuauuaaggucugcagcugcaaaaacauaggugcggaccuuaaagauucuucc | manual\_scaffold\_3a:623537546..623537607:+ |
| manual\_scaffold\_10\_34349 | 1.7 | 0.91 ± 0.01 |  | 114 | 114 | 0 | 0 | yes |  |  |  | blast | augcacugagacuccacacuugu | gagcguggagucucugcacauaa | gagcguggagucucugcacauaaaaagaucuauuguuuuaugcacugagacuccacacuugu | manual\_scaffold\_10:1049588291..1049588353:+ |
| manual\_scaffold\_5\_17777 | 1.7 | 0.91 ± 0.01 |  | 110 | 110 | 0 | 0 | yes |  |  |  | blast | aauuguuaggucgccucugcac | gcagaggcguccugacaaguuug | gcagaggcguccugacaaguuugcagaccuccaaauuguuaggucgccucugcac | manual\_scaffold\_5:263863631..263863686:+ |
| manual\_scaffold\_9\_31340 | 1.7 | 0.91 ± 0.01 |  | 87 | 87 | 0 | 0 | yes |  |  |  | blast | cuugcagugaucuugugcuguu | cagcauaagagcacugcuuucg | cuugcagugaucuugugcuguuuacuauuauaaacagcauaagagcacugcuuucg | manual\_scaffold\_9:482256324..482256380:+ |
| manual\_scaffold\_1b\_2314 | 1.7 | 0.91 ± 0.01 |  | 40 | 40 | 0 | 0 | yes |  |  |  | blast | uccgggacuuguaguauaguuu | auuaggacuacaggucccauaau | uccgggacuuguaguauaguuuauuucauuauaaauuaggacuacaggucccauaau | manual\_scaffold\_1b:605463383..605463440:+ |
| manual\_scaffold\_6\_21859 | 1.7 | 0.91 ± 0.01 |  | 35 | 35 | 0 | 0 | yes |  |  |  | blast | uuauuuuuuugcuguuagcucu | agccagcagcagaaaaauaaaa | agccagcagcagaaaaauaaaauauauucaaauacuguuuuauuuuuuugcuguuagcucu | manual\_scaffold\_6:1077795134..1077795195:+ |
| manual\_scaffold\_5\_18273 | 1.7 | 0.91 ± 0.01 |  | 34 | 34 | 0 | 0 | yes |  |  |  | blast | aggggguagagcacuguu | caguucugcccccuug | aggggguagagcacuguugggcaguucugcccccuug | manual\_scaffold\_5:857391733..857391770:+ |
| manual\_scaffold\_1b\_3478 | 1.7 | 0.91 ± 0.01 |  | 16 | 16 | 0 | 0 | yes |  |  |  | blast | cugaucagacuccacccucgga | ugggguggcaguggauucacggg | ugggguggcaguggauucacggguugcuauucccugaucagacuccacccucgga | manual\_scaffold\_1b:792833029..792833084:- |
| manual\_scaffold\_10\_35340 | 1.7 | 0.91 ± 0.01 |  | 306 | 306 | 0 | 0 | yes |  |  |  | blast | uugaauucugggacuuguaguc | cuacaagucacacaauucaaagg | uugaauucugggacuuguagucuuguuuaugccauuguauaacugggcuacaagucacacaauucaaagg | manual\_scaffold\_10:666872518..666872588:- |
| manual\_scaffold\_7\_27642 | 1.7 | 0.91 ± 0.01 |  | 19 | 19 | 0 | 0 | yes |  |  |  | blast | uugaucgcagcgugauugc | gaucgcgcugcgaacaaaa | gaucgcgcugcgaacaaaaguuaaguaggacggugauccguacaauuaucuuuugaucgcagcgugauugc | manual\_scaffold\_7:1129203301..1129203372:- |
| manual\_scaffold\_6\_24143 | 1.7 | 0.91 ± 0.01 |  | 87 | 87 | 0 | 0 | yes |  |  |  | blast | uucuagaagucagcgaguacu | ggcucggcuucuucuagauuu | uucuagaagucagcgaguacuaggaguuuugucccugggcucggcuucuucuagauuu | manual\_scaffold\_6:1276803975..1276804033:- |
| manual\_scaffold\_1a\_895 | 1.6 | 0.91 ± 0.01 |  | 16 | 15 | 0 | 1 | yes |  |  |  | blast | uuuagaauucgacggacuggagu | cguccgccgaaaucuaaauccu | cguccgccgaaaucuaaauccuauuuuauucuaagggauuuagaauucgacggacuggagu | manual\_scaffold\_1a:1191563129..1191563190:+ |
| manual\_scaffold\_2a\_5175 | 1.6 | 0.91 ± 0.01 |  | 214 | 155 | 0 | 59 | yes |  |  |  | blast | aacccuaccccagcuaucuug | caggcagugggguagggaac | aacccuaccccagcuaucuuguggaagucuuuugaaaauacaggcagugggguagggaac | manual\_scaffold\_2a:365872410..365872470:- |
| manual\_scaffold\_9\_32994 | 1.6 | 0.91 ± 0.01 |  | 36 | 28 | 0 | 8 | yes |  |  |  | blast | cauggggcaaaucauugaguuu | ucuugaugauuuuccccacuugcu | ucuugaugauuuuccccacuugcugugcuuuccugucuaguaaugcauggggcaaaucauugaguuu | manual\_scaffold\_9:908516697..908516764:- |
| manual\_scaffold\_11\_37746 | 1.6 | 0.91 ± 0.01 |  | 300 | 287 | 0 | 13 | yes |  |  |  | blast | uagggcacuggggcaucugccu | ugaaagaugccauggugcc | ugaaagaugccauggugccuguuagugauugugaauagggcacuggggcaucugccu | manual\_scaffold\_11:1013878547..1013878604:- |
| manual\_scaffold\_2a\_5052 | 1.6 | 0.91 ± 0.01 |  | 238 | 238 | 0 | 0 | yes |  |  |  | blast | uauaguauuccaccuacccuga | aagguggguggaauaauauaac | aagguggguggaauaauauaacaauaacccccguuguuauaguauuccaccuacccuga | manual\_scaffold\_2a:181285432..181285491:- |
| manual\_scaffold\_3a\_9941 | 1.6 | 0.91 ± 0.01 |  | 78 | 78 | 0 | 0 | yes |  |  |  | blast | uguaguacuccugcauuguuccu | gaauaggcaguaguacaucgac | uguaguacuccugcauuguuccuguuauccagguaaugaccugcaccuaaaacucaggaauaggcaguaguacaucgac | manual\_scaffold\_3a:930058075..930058154:- |
| manual\_scaffold\_6\_24326 | 1.6 | 0.91 ± 0.01 |  | 224 | 224 | 0 | 0 | yes |  |  |  | blast | auuugcgugcauacgucccgc | ggggggcaugcccucaaaucc | ggggggcaugcccucaaauccuguuaaaaaauaaugggauuugcgugcauacgucccgc | manual\_scaffold\_6:1554149798..1554149857:- |
| manual\_scaffold\_6\_23736 | 1.6 | 0.91 ± 0.01 |  | 60 | 60 | 0 | 0 | yes |  |  |  | blast | uagcagaacagacgcauugccu | ccaaugcaucuacucuccugcg | ccaaugcaucuacucuccugcgcggugacauccguagcagaacagacgcauugccu | manual\_scaffold\_6:783400256..783400312:- |
| manual\_scaffold\_10\_34347 | 1.6 | 0.91 ± 0.01 |  | 38 | 38 | 0 | 0 | yes |  |  |  | blast | cugacugagccagcgagu | ccgcugacuaugagccagca | ccgcugacuaugagccagcagcagaaaaauaaaacaauauuucaaauaguuuauuuuucugcugcugacugagccagcgagu | manual\_scaffold\_10:1048924596..1048924678:+ |
| manual\_scaffold\_9\_30851 | 1.6 | 0.91 ± 0.01 |  | 64 | 57 | 0 | 7 | yes |  |  |  | blast | agugaugaaucugcgcugagcu | agcgcagcauugauuuuucagc | agugaugaaucugcgcugagcuggcgaugcaucaauuucaccagcgcagcauugauuuuucagc | manual\_scaffold\_9:18836457..18836521:+ |
| manual\_scaffold\_4b\_16100 | 1.6 | 0.91 ± 0.01 |  | 11 | 4 | 0 | 7 | yes |  |  |  | blast | aagacaggagucaugcccacc | caugggcaugaguccugucu | caugggcaugaguccugucuaaauaaagacaggagucaugcccacc | manual\_scaffold\_4b:1191913767..1191913813:+ |
| manual\_scaffold\_9\_33008 | 1.6 | 0.91 ± 0.01 |  | 638 | 638 | 0 | 0 | yes |  |  |  | blast | uuccugagcuucugcaacaaga | uugccucaggagcccaggaagu | uugccucaggagcccaggaaguuucuauggcuugcugcuuccugagcuucugcaacaaga | manual\_scaffold\_9:939655446..939655506:- |
| manual\_scaffold\_3b\_11963 | 1.6 | 0.91 ± 0.01 |  | 14 | 14 | 0 | 0 | yes |  |  |  | blast | uacuuguaauccuuaugggccc | gcccacugguuauuaugaguaac | uacuuguaauccuuaugggcccagugacucacuuucugggcccacugguuauuaugaguaac | manual\_scaffold\_3b:923991015..923991077:- |
| manual\_scaffold\_6\_22699 | 1.6 | 0.91 ± 0.01 |  | 23 | 23 | 0 | 0 | yes |  |  |  | blast | accccgguggcugcggcugu | ggggugcagcuuagggugucu | accccgguggcugcggcuguggggugucugauucaugcucuucucugagaccccguggggugcagcuuagggugucu | manual\_scaffold\_6:7395558..7395635:- |
| manual\_scaffold\_1a\_1559 | 1.6 | 0.91 ± 0.01 |  | 11 | 11 | 0 | 0 | yes |  |  |  | blast | ggugaugagcccuaggauuggcu | ccaauccuagguaaccuuacaug | ggugaugagcccuaggauuggcuggggacacacgagccaauccuagguaaccuuacaug | manual\_scaffold\_1a:892363190..892363249:- |
| manual\_scaffold\_6\_21928 | 1.6 | 0.91 ± 0.01 |  | 112 | 112 | 0 | 0 | yes |  |  |  | blast | uuguggcuguugggcacagacu | ucugugugcgcagauacagaagg | uuguggcuguugggcacagacuguuuucuagucugugugcgcagauacagaagg | manual\_scaffold\_6:1155143780..1155143834:+ |
| manual\_scaffold\_3a\_9106 | 1.6 | 0.91 ± 0.01 |  | 58 | 38 | 0 | 20 | yes |  |  |  | blast | uaagauugaauggcacugcca | ugguaaugccaaucggucuugc | ugguaaugccaaucggucuugcuuacauuuacauacaaguaagauugaauggcacugcca | manual\_scaffold\_3a:362748289..362748349:- |
| manual\_scaffold\_9\_32159 | 1.6 | 0.91 ± 0.01 |  | 199 | 199 | 0 | 0 | yes |  |  |  | blast | ugggagaagcaccuggauugg | auuccuggagcugcucucuug | auuccuggagcugcucucuugcuguuugcagcaugggagaagcaccuggauugg | manual\_scaffold\_9:1198773719..1198773773:+ |
| manual\_scaffold\_8\_28980 | 1.6 | 0.91 ± 0.01 |  | 53 | 53 | 0 | 0 | yes |  |  |  | blast | aaucguaaucccaaggacagu | uguccugucggauuaggaccac | aaucguaaucccaaggacaguguuuuuugcagugcuguccugucggauuaggaccac | manual\_scaffold\_8:564507849..564507906:+ |
| manual\_scaffold\_1b\_2246 | 1.6 | 0.91 ± 0.01 |  | 31 | 31 | 0 | 0 | yes |  |  |  | blast | ugugguaccugcuggaacucuc | gagggccaagaggugcuuuugg | ugugguaccugcuggaacucucuaacugugugggagggccaagaggugcuuuugg | manual\_scaffold\_1b:512137762..512137817:+ |
| manual\_scaffold\_6\_24226 | 1.6 | 0.91 ± 0.01 |  | 1134 | 1124 | 0 | 10 | yes |  |  |  | blast | uagcggaugcaggguucagau | gucugggcaugcaucuguugga | uagcggaugcaggguucagauaaucaguuaaagucugggcaugcaucuguugga | manual\_scaffold\_6:1399316550..1399316604:- |
| manual\_scaffold\_6\_22256 | 1.6 | 0.91 ± 0.01 |  | 14 | 14 | 0 | 0 | yes |  |  |  | blast | uugaggucucccugugacucccu | ugggucacuagugggggauccaaca | uugaggucucccugugacucccugucccugcuaccagugggucacuagugggggauccaaca | manual\_scaffold\_6:1452955958..1452956020:+ |
| manual\_scaffold\_3b\_10235 | 1.6 | 0.91 ± 0.01 |  | 22 | 22 | 0 | 0 | yes |  |  |  | blast | uguaacgaucgcuuccggaga | uccgguagcgggucgacgcggu | uccgguagcgggucgacgcgguggaaggacuguaacgaucgcuuccggaga | manual\_scaffold\_3b:265817214..265817265:+ |
| manual\_scaffold\_7\_25219 | 1.6 | 0.91 ± 0.01 |  | 93 | 92 | 1 | 0 | yes |  |  |  | blast | gaagcgcuuucaggacugcugu | agcaucagugggagcgcuuuca | agcaucagugggagcgcuuucaggacugcugucugcuguugaugugcaguggagcugggaagcgcuuucaggacugcugu | manual\_scaffold\_7:607634322..607634402:+ |
| manual\_scaffold\_6\_22629 | 1.6 | 0.91 ± 0.01 |  | 105 | 105 | 0 | 0 | yes |  |  |  | blast | cacugugaucacucugaugccu | gucgucagaggcacacugugcc | gucgucagaggcacacugugccuguuauuccaaggcacugugaucacucugaugccu | manual\_scaffold\_6:1759685057..1759685114:+ |
| manual\_scaffold\_1a\_1196 | 1.6 | 0.91 ± 0.01 |  | 21 | 21 | 0 | 0 | yes |  |  |  | blast | gcgguaguugauauucugcacu | ugcagaauauuaacuaaaacug | ugcagaauauuaacuaaaacuguaucuugaaaguauagcgguaguugauauucugcacu | manual\_scaffold\_1a:396075615..396075674:- |
| manual\_scaffold\_5\_18144 | 1.6 | 0.91 ± 0.01 |  | 14 | 14 | 0 | 0 | yes |  |  |  | blast | aggauaucccauucacuguauu | uaugguggaggggauauccauc | aggauaucccauucacuguauuacaagugccauucauauaaugcacuuguuauaugguggaggggauauccauc | manual\_scaffold\_5:673564478..673564552:+ |
| manual\_scaffold\_5\_19696 | 1.6 | 0.91 ± 0.01 |  | 10 | 8 | 0 | 2 | no |  |  |  | blast | ugaguugguguugccggaaggu | uucgggcaccuccaaaucucagc | uucgggcaccuccaaaucucagcgcuauaaaggcauucugaguugguguugccggaaggu | manual\_scaffold\_5:700121571..700121631:- |
| manual\_scaffold\_6\_24511 | 1.6 | 0.91 ± 0.01 |  | 12 | 12 | 0 | 0 | yes |  |  |  | blast | ccuuggcugugucugagc | ucagaccaaucccggag | ccuuggcugugucugagcaccaguccgagcugcucagaccaaucccggag | manual\_scaffold\_6:1713079957..1713080007:- |
| manual\_scaffold\_9\_32821 | 1.6 | 0.91 ± 0.01 |  | 332 | 332 | 0 | 0 | yes |  |  |  | blast | acguguaucaaaggauuuuacc | gaaaaugcuuuaguacauuugg | acguguaucaaaggauuuuacccauucugugucuaugggaaaaugcuuuaguacauuugg | manual\_scaffold\_9:772342641..772342701:- |
| manual\_scaffold\_6\_21891 | 1.6 | 0.91 ± 0.01 |  | 113 | 113 | 0 | 0 | yes |  |  |  | blast | ucggcagggggacuggcucugc | agggcucagcucccuuucggag | agggcucagcucccuuucggaguucuccagaugacuugaucggcagggggacuggcucugc | manual\_scaffold\_6:1120248861..1120248922:+ |
| manual\_scaffold\_9\_31991 | 1.6 | 0.91 ± 0.01 |  | 142 | 117 | 0 | 25 | yes |  |  |  | blast | agcgcagcaucugaauuggc | cccaauccugaugcugcucuca | cccaauccugaugcugcucucauggguauuaccagcaugagcgcagcaucugaauuggc | manual\_scaffold\_9:1062194325..1062194384:+ |
| manual\_scaffold\_9\_32693 | 1.6 | 0.91 ± 0.01 |  | 29 | 27 | 0 | 2 | yes |  |  |  | blast | augauuguaaccagccagggc | cuccuggcugauuacaaccc | augauuguaaccagccagggcagcgcugaacucaguaccuccuggcugauuacaaccc | manual\_scaffold\_9:599718074..599718132:- |
| manual\_scaffold\_8\_28638 | 1.6 | 0.91 ± 0.01 |  | 133 | 133 | 0 | 0 | yes |  |  |  | blast | cuuuucuugcacucaucccuuu | aggggugguggcaaggauagag | cuuuucuugcacucaucccuuuugagucuuauaaaaggggugguggcaaggauagag | manual\_scaffold\_8:162142416..162142473:+ |
| manual\_scaffold\_8\_29250 | 1.6 | 0.91 ± 0.01 |  | 31 | 31 | 0 | 0 | yes |  |  |  | blast | cuagcugugcucuuaugcuguc | cagcaagggagcagugcuugua | cuagcugugcucuuaugcugucuagcacuguaagcagcaagggagcagugcuugua | manual\_scaffold\_8:1044523005..1044523061:+ |
| manual\_scaffold\_4b\_15063 | 1.6 | 0.91 ± 0.01 |  | 20 | 18 | 0 | 2 | yes |  |  |  | blast | uucaagauggccaguugccu | ugguggcucaccauuuugaaag | ugguggcucaccauuuugaaaguuuaugaaaugcccuuucaagauggccaguugccu | manual\_scaffold\_4b:181048088..181048145:+ |
| manual\_scaffold\_4b\_14980 | 1.6 | 0.91 ± 0.01 |  | 16 | 16 | 0 | 0 | yes |  |  |  | blast | ccuggacuucaacgaauguaauguu | cacuguguguuggagcagggu | ccuggacuucaacgaauguaauguucuugagccagccauuccugguuauggugcacuguguguuggagcagggu | manual\_scaffold\_4b:144979053..144979127:+ |
| manual\_scaffold\_4b\_16102 | 1.6 | 0.91 ± 0.01 |  | 11 | 4 | 0 | 7 | yes |  |  |  | blast | aagacaggagucaugcccacc | caugggcaugaguccugucu | caugggcaugaguccugucuaaauaaagacaggagucaugcccacc | manual\_scaffold\_4b:1192106805..1192106851:+ |
| manual\_scaffold\_6\_23435 | 1.6 | 0.91 ± 0.01 |  | 101 | 70 | 0 | 31 | yes |  |  |  | blast | gaaaugaugcacggcuguuuagc | accagccuaucgucauuuuua | gaaaugaugcacggcuguuuagcugcauuuuuuugucucuaaccagccuaucgucauuuuua | manual\_scaffold\_6:440066282..440066344:- |
| manual\_scaffold\_3a\_7488 | 1.6 | 0.91 ± 0.01 |  | 15 | 15 | 0 | 0 | yes |  |  |  | blast | uacccugguugcuguauauaua | uauauauaucaaccagggcaaa | uacccugguugcuguauauauauauauauauauauauauauauaucaaccagggcaaa | manual\_scaffold\_3a:231604108..231604166:+ |
| manual\_scaffold\_2b\_6725 | 1.6 | 0.91 ± 0.01 |  | 19 | 19 | 0 | 0 | yes |  |  |  | blast | cggugccuuaaacaugcaug | uguuuaguuaaggcacagaa | cggugccuuaaacaugcaugugcugaaccacgcacauguuuaguuaaggcacagaa | manual\_scaffold\_2b:275880722..275880778:- |
| manual\_scaffold\_11\_37246 | 1.6 | 0.91 ± 0.01 |  | 356 | 350 | 6 | 0 | yes |  |  |  | blast | ugggacaccugccggucuuucu | caagacguggcaguguuuugucucuuu | caagacguggcaguguuuugucucuuucucuuccacucccccuuuccuuuugggacaccugccggucuuucu | manual\_scaffold\_11:444943313..444943385:- |
| manual\_scaffold\_7\_24809 | 1.6 | 0.91 ± 0.01 |  | 176471 | 174990 | 0 | 1481 | yes |  |  |  | blast | uaaggcacgcggugaaugcc | cguguucacagcggaccuugau | cguguucacagcggaccuugauuuaauauccauacaauuaaggcacgcggugaaugcc | manual\_scaffold\_7:111850323..111850381:+ |
| manual\_scaffold\_11\_35995 | 1.6 | 0.91 ± 0.01 |  | 224 | 223 | 0 | 1 | yes |  |  |  | blast | uacagcggugggauaucugucc | ugacggauaucccaaccgccgu | ugacggauaucccaaccgccguauccuauggagauaauaauacagcggugggauaucugucc | manual\_scaffold\_11:146763038..146763100:+ |
| manual\_scaffold\_3a\_7365 | 1.6 | 0.91 ± 0.01 |  | 23 | 23 | 0 | 0 | yes |  |  |  | blast | uugcuuuuacuuuguagguguccu | gagccuacguuguagaagaaaau | gagccuacguuguagaagaaaauguuaccuuuaaucuugcuuuuacuuuguagguguccu | manual\_scaffold\_3a:137577132..137577192:+ |
| manual\_scaffold\_6\_21718 | 1.6 | 0.91 ± 0.01 |  | 75 | 38 | 0 | 37 | yes |  |  |  | blast | aaaacagcgucuugauuggaug | aucaagacuguuuuuucaugcu | aucaagacuguuuuuucaugcugcuuaaaaucaugaaaacagcgucuugauuggaug | manual\_scaffold\_6:914345363..914345420:+ |
| manual\_scaffold\_4a\_13272 | 1.6 | 0.91 ± 0.01 |  | 123 | 123 | 0 | 0 | yes |  |  |  | blast | uucgaauucugugacuuguagu | uacaagucccagaauucacaga | uucgaauucugugacuuguaguccuguuuuuaucaugacaaagcaagacuacaagucccagaauucacaga | manual\_scaffold\_4a:682389802..682389873:+ |
| manual\_scaffold\_3b\_9989 | 1.6 | 0.91 ± 0.01 |  | 23 | 23 | 0 | 0 | yes |  |  |  | blast | cagaguagaauguuugcccuugc | aggugcaagcauuuuauuuucuuugcu | aggugcaagcauuuuauuuucuuugcuguauccaagugcgagcagaguagaauguuugcccuugc | manual\_scaffold\_3b:4245738..4245803:+ |
| manual\_scaffold\_5\_19184 | 1.6 | 0.91 ± 0.01 |  | 54 | 54 | 0 | 0 | yes |  |  |  | blast | gcaugaaagcagcgacuggau | cuuguucuucuuucauucug | cuuguucuucuuucauucugggcaauauuaucagcagcaugaaagcagcgacuggau | manual\_scaffold\_5:64137149..64137206:- |
| manual\_scaffold\_10\_35783 | 1.6 | 0.91 ± 0.01 |  | 36 | 36 | 0 | 0 | yes |  |  |  | blast | auacuaacccuucuuucaugcu | caugaaagcagaguuaggauua | auacuaacccuucuuucaugcugcaagcagcaugaaagcagaguuaggauua | manual\_scaffold\_10:1042405684..1042405736:- |
| manual\_scaffold\_3b\_11733 | 1.6 | 0.91 ± 0.01 |  | 68 | 68 | 0 | 0 | yes |  |  |  | blast | ugcaggaucacauagauugug | caccaugugacgccugucgc | ugcaggaucacauagauugugggguacuaaguacaauuccacaccaugugacgccugucgc | manual\_scaffold\_3b:661560625..661560686:- |
| manual\_scaffold\_5\_19531 | 1.6 | 0.91 ± 0.01 |  | 22 | 22 | 0 | 0 | yes |  |  |  | blast | ugcuguaaucaucuguaacccu | ggguucaggugagguacagugag | ugcuguaaucaucuguaacccugggaagcuuggggguucaggugagguacagugag | manual\_scaffold\_5:501020804..501020860:- |
| manual\_scaffold\_9\_31861 | 1.6 | 0.91 ± 0.01 |  | 14 | 14 | 0 | 0 | yes |  |  |  | blast | cgaacccgacaugcacacuga | agugcgcaugucgggcuggcu | agugcgcaugucgggcuggcuauaacaagacaaccggcgaacccgacaugcacacuga | manual\_scaffold\_9:916082599..916082657:+ |
| manual\_scaffold\_3a\_8696 | 1.6 | 0.91 ± 0.01 |  | 12 | 12 | 0 | 0 | yes |  |  |  | blast | uauaaauccuagaauucaaaga | uuugcauucugggaguuguagc | uuugcauucugggaguuguagcccuguuuuauacuggagacaagauuauaaauccuagaauucaaaga | manual\_scaffold\_3a:11009649..11009717:- |
| manual\_scaffold\_9\_32833 | 1.6 | 0.91 ± 0.01 |  | 22 | 22 | 0 | 0 | yes |  |  |  | blast | ucuguccuggguguucugugga | cucagaacgccggggauggggg | ucuguccuggguguucuguggaacaacaugucauuuccucagaacgccggggauggggg | manual\_scaffold\_9:797451397..797451456:- |
| manual\_scaffold\_4b\_17215 | 1.6 | 0.91 ± 0.01 |  | 11 | 11 | 0 | 0 | yes |  |  |  | blast | cuugcagugcucuuauggugu | agcaugagagcacugcuucca | cuugcagugcucuuaugguguuagcuaauauaaacagcaugagagcacugcuucca | manual\_scaffold\_4b:938733229..938733285:- |
| manual\_scaffold\_3a\_8381 | 1.6 | 0.91 ± 0.01 |  | 21 | 21 | 0 | 0 | yes |  |  |  | blast | uguagguccaggaaaguauguuu | acaugcuuuccuuugggacugcucu | acaugcuuuccuuugggacugcucugaauuaugggugcaaguguagguccaggaaaguauguuu | manual\_scaffold\_3a:654070061..654070125:+ |
| manual\_scaffold\_6\_24104 | 1.6 | 0.91 ± 0.01 |  | 11 | 11 | 0 | 0 | yes |  |  |  | blast | cccaaagugacaugugcacu | ugcucauguuacuuuggcca | ugcucauguuacuuuggccagugcaaaacagccgcccaaagugacaugugcacu | manual\_scaffold\_6:1187883644..1187883698:- |
| manual\_scaffold\_4b\_15399 | 1.6 | 0.91 ± 0.01 |  | 23 | 23 | 0 | 0 | yes |  |  |  | blast | caugugacaggaaaaguguacc | uauacuuuuccuuuugcaccacagug | uauacuuuuccuuuugcaccacagugcaucuuuaagcacaugugacaggaaaaguguacc | manual\_scaffold\_4b:544055451..544055511:+ |
| manual\_scaffold\_4b\_16834 | 1.6 | 0.91 ± 0.01 |  | 22 | 22 | 0 | 0 | yes |  |  |  | blast | accuguagccaaacaugauugu | agucauguucuucuccgggugc | agucauguucuucuccgggugcacgaaacgagagugcaccuguagccaaacaugauugu | manual\_scaffold\_4b:436531250..436531309:- |
| manual\_scaffold\_5\_17642 | 1.6 | 0.91 ± 0.01 |  | 29 | 26 | 0 | 3 | yes |  |  |  | blast | ucacuaaauuugucaggaagcu | ugcuuuuugugauuuggug | ucacuaaauuugucaggaagcuagaucuuaguccacagaucaugcuuuuugugauuuggug | manual\_scaffold\_5:123010412..123010473:+ |
| manual\_scaffold\_4b\_14827 | 1.6 | 0.91 ± 0.01 |  | 14 | 14 | 0 | 0 | yes |  |  |  | blast | gucugaccacgcaucuguugga | uggcagaugcaggguucagauaa | uggcagaugcaggguucagauaaucaguaaagucugaccacgcaucuguugga | manual\_scaffold\_4b:39583091..39583144:+ |
| manual\_scaffold\_7\_26703 | 1.6 | 0.91 ± 0.01 |  | 10 | 10 | 0 | 0 | yes |  |  |  | blast | uggcaacaaccuccaggucugu | aaaucuggagguuguugcuaga | uggcaacaaccuccaggucugugauguuaauacccaacauaaaucuggagguuguugcuaga | manual\_scaffold\_7:157902034..157902096:- |
| manual\_scaffold\_5\_18314 | 1.6 | 0.91 ± 0.01 |  | 30 | 30 | 0 | 0 | yes |  |  |  | blast | accgcggucggcggacaugccu | gcugucuggaagacuguuggagu | accgcggucggcggacaugccuguaugacuuguaauagggcugucuggaagacuguuggagu | manual\_scaffold\_5:903727710..903727772:+ |
| manual\_scaffold\_2b\_6324 | 1.5 | 0.91 ± 0.01 |  | 13 | 13 | 0 | 0 | yes |  |  |  | blast | ucugaauauuuuuuguaauaagcu | uuuuacaaaaaacauucaaaga | ucugaauauuuuuuguaauaagcuuuucguacuaaguuuuacaaaaaacauucaaaga | manual\_scaffold\_2b:587083831..587083889:+ |
| manual\_scaffold\_4b\_15450 | 1.5 | 0.91 ± 0.01 |  | 19 | 19 | 0 | 0 | yes |  |  |  | blast | uacuauuccaaaaugucagcuu | gccaucuuggaaugguagu | uacuauuccaaaaugucagcuucuuaaauauugcacugggcggccaucuuggaaugguagu | manual\_scaffold\_4b:582142123..582142184:+ |
| manual\_scaffold\_1a\_1093 | 1.5 | 0.91 ± 0.01 |  | 19 | 19 | 0 | 0 | yes |  |  |  | blast | aagggcuguguugcuuuggcac | gcaaaagcaacacaaccgucaa | aagggcuguguugcuuuggcaccaugcaacagggugcaaaagcaacacaaccgucaa | manual\_scaffold\_1a:261076038..261076095:- |
| manual\_scaffold\_4a\_12779 | 1.5 | 0.91 ± 0.01 |  | 17 | 17 | 0 | 0 | yes |  |  |  | blast | uggauaauuucugcucuucccu | gagagggcaggaaugauccaua | gagagggcaggaaugauccauauuuacaaggguauggauaauuucugcucuucccu | manual\_scaffold\_4a:96273512..96273568:+ |
| manual\_scaffold\_9\_31401 | 1.5 | 0.91 ± 0.01 |  | 25 | 25 | 0 | 0 | yes |  |  |  | blast | cugcccagugcucugaaa | ccagugcagggaacaggg | ccagugcagggaacaggggugagugauaaucaaauaucccugcccagugcucugaaa | manual\_scaffold\_9:541161818..541161875:+ |
| manual\_scaffold\_6\_22140 | 1.5 | 0.91 ± 0.01 |  | 11 | 11 | 0 | 0 | yes |  |  |  | blast | ugucacggcccuuggcucgccc | ggagucacugggcagugacuug | ugucacggcccuuggcucgcccagaacacagggggccuguggggagucacugggcagugacuug | manual\_scaffold\_6:1366265970..1366266034:+ |
| manual\_scaffold\_2b\_6102 | 1.5 | 0.91 ± 0.01 |  | 37 | 37 | 0 | 0 | yes |  |  |  | blast | uuccgugacuauauagaggaa | uacaaggauuauaagguuacggaauu | uuccgugacuauauagaggaacaaagguuuacaaggauuauaagguuacggaauu | manual\_scaffold\_2b:247208572..247208627:+ |
| manual\_scaffold\_8\_29772 | 1.5 | 0.91 ± 0.01 |  | 59 | 59 | 0 | 0 | yes |  |  |  | blast | uucgcaauuuuuggaaauuc | auuuccaaaauugcgaauu | auuuccaaaauugcgaauuccuaucugcaauucgcaauuuuuggaaauuc | manual\_scaffold\_8:81013317..81013367:- |
| manual\_scaffold\_6\_21424 | 1.5 | 0.91 ± 0.01 |  | 20 | 20 | 0 | 0 | yes |  |  |  | blast | aacaagaagucauaaauugc | aauuugugauuucuuauuag | aacaagaagucauaaauugcaucaaauaaggauuugcaauuugugauuucuuauuag | manual\_scaffold\_6:619307106..619307163:+ |
| manual\_scaffold\_10\_35286 | 1.5 | 0.91 ± 0.01 |  | 25 | 23 | 0 | 2 | yes |  |  |  | blast | uuucucaggaauacucauggaacu | ugccagaguacuugugggaagcu | ugccagaguacuugugggaagcuguauccauuuggugguuucucaggaauacucauggaacu | manual\_scaffold\_10:614683579..614683641:- |
| manual\_scaffold\_7\_25211 | 1.5 | 0.91 ± 0.01 |  | 40 | 39 | 0 | 1 | yes |  |  |  | blast | caaagaaguuuggugagcgu | cugcucaccaaacuucuugguu | cugcucaccaaacuucuugguuucuuugcaaaaacaaacucccaaacaaagaaguuuggugagcgu | manual\_scaffold\_7:606082951..606083017:+ |
| manual\_scaffold\_5\_20425 | 1.5 | 0.91 ± 0.01 |  | 45 | 45 | 0 | 0 | yes |  |  |  | blast | ccgggccaagcugagcgugguu | ccacauucuuccagaccccuga | ccgggccaagcugagcgugguuauggaagacaagaccacauucuuccagaccccuga | manual\_scaffold\_5:1679146136..1679146193:- |
| manual\_scaffold\_10\_34445 | 1.5 | 0.91 ± 0.01 |  | 16 | 16 | 0 | 0 | yes |  |  |  | blast | acguggaaaccugcagaggu | cucugcauggcucuacaagu | acguggaaaccugcagagguguaacacacccaccucugcauggcucuacaagu | manual\_scaffold\_10:15420044..15420097:- |
| manual\_scaffold\_6\_21894 | 1.5 | 0.91 ± 0.01 |  | 73 | 73 | 0 | 0 | yes |  |  |  | blast | uugaauucugggacuagaaguc | cuacuaguccuagaauucaaag | uugaauucugggacuagaaguccuauuuuauaaagcaauagacaaaacuacuaguccuagaauucaaag | manual\_scaffold\_6:1123718495..1123718564:+ |
| manual\_scaffold\_12\_38693 | 1.5 | 0.91 ± 0.01 |  | 17 | 17 | 0 | 0 | yes |  |  |  | blast | uaccucacauuucauggacu | uccaugaaacaaggggugca | uccaugaaacaaggggugcagugaccuaaacccuuguaccucacauuucauggacu | manual\_scaffold\_12:665665838..665665894:+ |
| manual\_scaffold\_5\_20130 | 1.5 | 0.91 ± 0.01 |  | 24 | 24 | 0 | 0 | yes |  |  |  | blast | auaugguucuuuccugcucucu | agagaaggagagugccguuucu | agagaaggagagugccguuucuauguguauaugguucuuuccugcucucu | manual\_scaffold\_5:1249048809..1249048859:- |
| manual\_scaffold\_3b\_10317 | 1.5 | 0.91 ± 0.01 |  | 41 | 37 | 0 | 4 | yes |  |  |  | blast | uugugauauggagaacgggaa | auccugucuaccguauuacaagu | auccugucuaccguauuacaagucccacggcuauaaugcauuugugauauggagaacgggaa | manual\_scaffold\_3b:377179552..377179614:+ |
| manual\_scaffold\_10\_33437 | 1.5 | 0.91 ± 0.01 |  | 1754 | 1752 | 0 | 2 | no |  | xtr-miR-428a |  | blast | aaagugcugucuaguuugggcu | cgcccacaccggggcgcucucu | cgcccacaccggggcgcucucuccucguaugagaaagugcugucuaguuugggcu | manual\_scaffold\_10:48075595..48075650:+ |
| manual\_scaffold\_2a\_4745 | 1.5 | 0.91 ± 0.01 |  | 12 | 12 | 0 | 0 | yes |  |  |  | blast | cuugaucguggagugccugcua | gagggcaguuuaugaucaaaagga | gagggcaguuuaugaucaaaaggauuaggguuccuugaucguggagugccugcua | manual\_scaffold\_2a:1244128020..1244128075:+ |
| manual\_scaffold\_7\_25820 | 1.5 | 0.91 ± 0.01 |  | 26 | 26 | 0 | 0 | yes |  |  |  | blast | ucaccgggcugacaggcagaaac | uuccgcuggucagcacggcagaa | ucaccgggcugacaggcagaaacaucaaauuaugacacuuccgcuggucagcacggcagaa | manual\_scaffold\_7:1161231433..1161231494:+ |
| manual\_scaffold\_12\_38755 | 1.5 | 0.91 ± 0.01 |  | 25 | 25 | 0 | 0 | yes |  |  |  | blast | uccggauuccuugucugaggau | ccucagaugagggucugggaua | ccucagaugagggucugggauauucugcuuuuuauccggauuccuugucugaggau | manual\_scaffold\_12:692600663..692600719:+ |
| manual\_scaffold\_6\_23595 | 1.5 | 0.91 ± 0.01 |  | 21 | 18 | 0 | 3 | yes |  |  |  | blast | augggacuguuucgagucc | aggcucgcuuuuggucccauu | augggacuguuucgaguccugcugucuguacaaggcucgcuuuuggucccauu | manual\_scaffold\_6:621240683..621240736:- |
| manual\_scaffold\_8\_28711 | 1.5 | 0.91 ± 0.01 |  | 25 | 25 | 0 | 0 | yes |  |  |  | blast | gaaugcaacgaaaaaccugggu | gcagguuauuuauuugcauuuug | gcagguuauuuauuugcauuuuggugguuacaguuagcagaaugcaacgaaaaaccugggu | manual\_scaffold\_8:232476557..232476618:+ |
| manual\_scaffold\_1b\_2680 | 1.5 | 0.91 ± 0.01 |  | 153 | 153 | 0 | 0 | yes |  |  |  | blast | auggcaaugccaaucggucuugc | gggacugauaggcauuaccaaug | auggcaaugccaaucggucuugcuuaccguuacauacaagggggacugauaggcauuaccaaug | manual\_scaffold\_1b:1043745971..1043746035:+ |
| manual\_scaffold\_4a\_12777 | 1.5 | 0.91 ± 0.01 |  | 17 | 17 | 0 | 0 | yes |  |  |  | blast | uggauaauuucugcucuucccu | gagagggcaggaaugauccaua | gagagggcaggaaugauccauauuuacaaggguauggauaauuucugcucuucccu | manual\_scaffold\_4a:96252927..96252983:+ |
| manual\_scaffold\_6\_21676 | 1.5 | 0.91 ± 0.01 |  | 42 | 42 | 0 | 0 | yes |  |  |  | blast | uaacucuacacuuuuguaccu | guacaaagaugugggguuaag | uaacucuacacuuuuguaccuggacaaugugcagauauuucagguacaaagaugugggguuaag | manual\_scaffold\_6:866104281..866104345:+ |
| manual\_scaffold\_2b\_7066 | 1.5 | 0.91 ± 0.01 |  | 241 | 241 | 0 | 0 | yes |  |  |  | blast | ugagagcuguuggccuuggccaau | uggcaaagccaacagcuugacacu | uggcaaagccaacagcuugacacuuucaucauauaagugagagcuguuggccuuggccaau | manual\_scaffold\_2b:662759712..662759773:- |
| manual\_scaffold\_2a\_5164 | 1.5 | 0.91 ± 0.01 |  | 1188 | 963 | 0 | 225 | yes |  |  |  | blast | ugucaccuguugccuuuggu | accaaaacugcaaguggcgcuu | accaaaacugcaaguggcgcuuuguugccauaucgagugucaccuguugccuuuggu | manual\_scaffold\_2a:343331262..343331319:- |
| manual\_scaffold\_2b\_5998 | 1.5 | 0.91 ± 0.01 |  | 17 | 17 | 0 | 0 | yes |  |  |  | blast | uaucccacugggagauacuggcc | cccugguaucucucugcagggauaau | uaucccacugggagauacuggccaguaucgucaaauccggcccugguaucucucugcagggauaau | manual\_scaffold\_2b:100896209..100896275:+ |
| manual\_scaffold\_11\_36435 | 1.5 | 0.91 ± 0.01 |  | 59 | 59 | 0 | 0 | yes |  |  |  | blast | cgccgguggugcaaugcuuugu | aaggccuugcgccacuagagca | aaggccuugcgccacuagagcaucauuuuuagcaacgcgccgguggugcaaugcuuugu | manual\_scaffold\_11:731334906..731334965:+ |
| manual\_scaffold\_8\_30055 | 1.5 | 0.91 ± 0.01 |  | 35 | 35 | 0 | 0 | yes |  |  |  | blast | agguuugugaacgggcugucu | ccggcccgucacagacauau | agguuugugaacgggcugucuccgaccggcccgucacagacauau | manual\_scaffold\_8:420725683..420725728:- |
| manual\_scaffold\_12\_39991 | 1.5 | 0.91 ± 0.01 |  | 38 | 38 | 0 | 0 | yes |  |  |  | blast | cugaccgaggagaagcuucugcu | gagacgcuucugcugcuuucagug | cugaccgaggagaagcuucugcugcuuucagugcugugacaccacugacagaggagacgcuucugcugcuuucagug | manual\_scaffold\_12:714023956..714024033:- |
| manual\_scaffold\_9\_30845 | 1.5 | 0.91 ± 0.01 |  | 17 | 17 | 0 | 0 | yes |  |  |  | blast | uuugaaacguauuuggaggucu | auauccaaauauguuuugggaa | auauccaaauauguuuugggaagauuggcgagaagcuuuugaaacguauuuggaggucu | manual\_scaffold\_9:11507056..11507115:+ |
| manual\_scaffold\_7\_27960 | 1.5 | 0.91 ± 0.01 |  | 19 | 19 | 0 | 0 | yes |  |  |  | blast | augugcuguccucgcucuccu | uagaaagaggacagugcguca | uagaaagaggacagugcgucaagcaagaugugcuguccucgcucuccu | manual\_scaffold\_7:1427088993..1427089041:- |
| manual\_scaffold\_11\_36625 | 1.5 | 0.91 ± 0.01 |  | 12 | 12 | 0 | 0 | yes |  |  |  | blast | cagaggaccauuaauuuaacuc | ggcaaauuaaugguccaucagugcu | ggcaaauuaaugguccaucagugcugauagagaugcagaggaccauuaauuuaacuc | manual\_scaffold\_11:948823362..948823419:+ |
| manual\_scaffold\_4a\_14167 | 1.5 | 0.91 ± 0.01 |  | 40 | 37 | 0 | 3 | yes |  |  |  | blast | ugaggacuugcaugacggca | ugcggucacauaaguccucacc | ugaggacuugcaugacggcaauguacucuucauugcggucacauaaguccucacc | manual\_scaffold\_4a:155792569..155792624:- |
| manual\_scaffold\_2b\_6685 | 1.5 | 0.91 ± 0.01 |  | 30 | 30 | 0 | 0 | yes |  |  |  | blast | uuuguuuugugacucgcgauu | uugcgaguugcaaaacaaaag | uuuguuuugugacucgcgauuugcaauucacaaaggaucgcaaauugcgaguugcaaaacaaaag | manual\_scaffold\_2b:223129570..223129635:- |
| manual\_scaffold\_9\_32081 | 1.5 | 0.91 ± 0.01 |  | 19 | 19 | 0 | 0 | yes |  |  |  | blast | ucuaugaacccugccugagg | caaggcaagguucacagaca | caaggcaagguucacagacauuugauucacacugaggucuaugaacccugccugagg | manual\_scaffold\_9:1145865431..1145865488:+ |
| manual\_scaffold\_9\_31429 | 1.5 | 0.91 ± 0.01 |  | 207 | 193 | 0 | 14 | yes |  |  |  | blast | uagcagaugcaggguucagau | gucugaccacgcaucuguugga | uagcagaugcaggguucagauaaucaguuaaagucugaccacgcaucuguugga | manual\_scaffold\_9:575581756..575581810:+ |
| manual\_scaffold\_3a\_7372 | 1.5 | 0.91 ± 0.01 |  | 174 | 173 | 0 | 1 | yes |  |  |  | blast | caccaggccacuccaagaaucu | agauuuggaggugccuggaggg | caccaggccacuccaagaaucugugcuguuauagcauacagauuuggaggugccuggaggg | manual\_scaffold\_3a:153969482..153969543:+ |
| manual\_scaffold\_6\_23506 | 1.5 | 0.91 ± 0.01 |  | 19 | 19 | 0 | 0 | yes |  |  |  | blast | uuuauaaaugucacuggagccc | guucuguugacauuugcaaacc | guucuguugacauuugcaaaccgaaaggacgagugguuuauaaaugucacuggagccc | manual\_scaffold\_6:503311334..503311392:- |
| manual\_scaffold\_3a\_9611 | 1.5 | 0.91 ± 0.01 |  | 30 | 30 | 0 | 0 | yes |  |  |  | blast | ccuugcagugcucuugugcu | caugagagcagugcuuucau | ccuugcagugcucuugugcuguuaacuuuuauaaacaucaugagagcagugcuuucau | manual\_scaffold\_3a:558144531..558144589:- |
| manual\_scaffold\_9\_30919 | 1.5 | 0.91 ± 0.01 |  | 9 | 8 | 0 | 1 | no |  |  |  | blast | gugcgcaccgccucgggc | ccggcggucgcgguacgc | ccggcggucgcgguacgcaccgccgcggugcgcaccgccucgggc | manual\_scaffold\_9:65571378..65571423:+ |
| manual\_scaffold\_10\_33355 | 1.5 | 0.91 ± 0.01 |  | 28 | 28 | 0 | 0 | yes |  |  |  | blast | auuuuccaucacucugacaccu | cggucggaaugaugaagaacgg | cggucggaaugaugaagaacggcuauagggucauuuuccaucacucugacaccu | manual\_scaffold\_10:21092274..21092328:+ |
| manual\_scaffold\_10\_33943 | 1.5 | 0.91 ± 0.01 |  | 40 | 40 | 0 | 0 | yes |  |  |  | blast | cagcaugagagcaaugugagc | uagaagcuggucccuugcugga | uagaagcuggucccuugcuggaaacagcaugagagcaaugugagc | manual\_scaffold\_10:519053263..519053308:+ |
| manual\_scaffold\_7\_25695 | 1.5 | 0.91 ± 0.01 |  | 72 | 72 | 0 | 0 | yes |  |  |  | blast | agggaaaguugaaaagaa | cuuuucaguuucuuuuu | agggaaaguugaaaagaaaacuuuucaguuucuuuuu | manual\_scaffold\_7:1052661106..1052661143:+ |
| manual\_scaffold\_5\_19965 | 1.5 | 0.91 ± 0.01 |  | 24 | 24 | 0 | 0 | yes |  |  |  | blast | aggucuaaacaaaucucccugu | agggagauuuacuuaagccuuu | aggucuaaacaaaucucccuguaaauaaaaugcucagggagauuuacuuaagccuuu | manual\_scaffold\_5:1074408531..1074408588:- |
| manual\_scaffold\_7\_25653 | 1.5 | 0.91 ± 0.01 |  | 17 | 13 | 0 | 4 | yes |  |  |  | blast | uuccaagcugucugccaggugu | acauugcagccauuuuggaaug | acauugcagccauuuuggaaugguaagauaaaacauuccaagcugucugccaggugu | manual\_scaffold\_7:1020205546..1020205603:+ |
| manual\_scaffold\_6\_24430 | 1.5 | 0.91 ± 0.01 |  | 54 | 54 | 0 | 0 | yes |  |  |  | blast | uuauguagccuccauuuuucaga | ugagaaauggauacuacagaaaca | ugagaaauggauacuacagaaacagcguguuacuuuguuauguagccuccauuuuucaga | manual\_scaffold\_6:1625544960..1625545020:- |
| manual\_scaffold\_4b\_15759 | 1.5 | 0.91 ± 0.01 |  | 21 | 21 | 0 | 0 | yes |  |  |  | blast | uuaacacguuuuuggagccu | guucagauucguggaaaag | guucagauucguggaaaagguuggcuucaaucuuuaacacguuuuuggagccu | manual\_scaffold\_4b:951914151..951914204:+ |
| manual\_scaffold\_3a\_9808 | 1.5 | 0.91 ± 0.01 |  | 30 | 30 | 0 | 0 | yes |  |  |  | blast | uaguacuauuccaagauggccu | gccauauuggcaugguaaaacu | gccauauuggcaugguaaaacugugaaacacaauggguaguacuauuccaagauggccu | manual\_scaffold\_3a:786438739..786438798:- |
| manual\_scaffold\_3b\_11152 | 1.5 | 0.91 ± 0.01 |  | 9020 | 9017 | 0 | 3 | yes |  |  |  | blast | ucugagcaugcaucuguugga | ccuagcagaugcaggguucaggu | ccuagcagaugcaggguucagguaaucaguaaauucugagcaugcaucuguugga | manual\_scaffold\_3b:102527632..102527687:- |
| manual\_scaffold\_5\_19492 | 1.5 | 0.91 ± 0.01 |  | 41 | 41 | 0 | 0 | yes |  |  |  | blast | ucaagucccagcucuggcucu | agccagagcugccgacuuuagaac | agccagagcugccgacuuuagaacuggagaaccagguucaagucccagcucuggcucu | manual\_scaffold\_5:472345771..472345829:- |
| manual\_scaffold\_7\_25752 | 1.5 | 0.91 ± 0.01 |  | 574 | 447 | 0 | 127 | yes |  |  |  | blast | gaaaaagcuuucguacauauggc | gauguacgcaaggauuuuaccc | gauguacgcaaggauuuuacccauuuuguguccaugggaaaaagcuuucguacauauggc | manual\_scaffold\_7:1093511627..1093511687:+ |
| manual\_scaffold\_4b\_16177 | 1.5 | 0.91 ± 0.01 |  | 132 | 132 | 0 | 0 | yes |  |  |  | blast | auuaaauugacagcuggacagu | ugugcaguugccgauuuuauca | auuaaauugacagcuggacagugagcacggugugcacugugcaguugccgauuuuauca | manual\_scaffold\_4b:29506973..29507032:- |
| manual\_scaffold\_1a\_98 | 1.5 | 0.91 ± 0.01 |  | 33 | 33 | 0 | 0 | yes |  |  |  | blast | gaggaguagaaucgauuguaga | uguaaugauucuccuccuguc | gaggaguagaaucgauuguagauguuagauuccauacgucuguaaugauucuccuccuguc | manual\_scaffold\_1a:86723371..86723432:+ |
| manual\_scaffold\_5\_18731 | 1.5 | 0.91 ± 0.01 |  | 12 | 12 | 0 | 0 | yes |  |  |  | blast | uugagaggggcugcuccu | gagaggccuccuuugau | gagaggccuccuuugaugugcaaauggcaagcacacuugagaggggcugcuccu | manual\_scaffold\_5:1469094499..1469094553:+ |
| manual\_scaffold\_1b\_2948 | 1.4 | 0.91 ± 0.01 |  | 31 | 31 | 0 | 0 | yes |  |  |  | blast | ccacugagaacgagagagcgc | gcucauccguucucagugcca | gcucauccguucucagugccagcccauggaaucucugccacugagaacgagagagcgc | manual\_scaffold\_1b:104439312..104439370:- |
| manual\_scaffold\_5\_19533 | 1.4 | 0.91 ± 0.01 |  | 22 | 22 | 0 | 0 | yes |  |  |  | blast | ugcuguaaucaucuguaacccu | gguucaggugagguacagugag | ugcuguaaucaucuguaacccugggaagcuugaggguucaggugagguacagugag | manual\_scaffold\_5:501021294..501021350:- |
| manual\_scaffold\_5\_20545 | 1.4 | 0.91 ± 0.01 |  | 78 | 78 | 0 | 0 | yes |  |  |  | blast | aaagcucugccaccgacuuccu | gcucagugccagagcucaga | aaagcucugccaccgacuuccugugaacauuguucagggcucagugccagagcucaga | manual\_scaffold\_5:1856006165..1856006223:- |
| manual\_scaffold\_7\_27782 | 1.4 | 0.91 ± 0.01 |  | 17 | 17 | 0 | 0 | yes |  |  |  | blast | uggauaauuucugcucuucccu | gagagagcaaaaauuuuccaua | gagagagcaaaaauuuuccauauuuacaaagauguggauaauuucugcucuucccu | manual\_scaffold\_7:1223396178..1223396234:- |
| manual\_scaffold\_1b\_2634 | 1.4 | 0.91 ± 0.01 |  | 2889 | 2886 | 0 | 3 | yes |  |  |  | blast | uucuagaagucauuggauacu | uggauccaacaucuucuaga | uucuagaagucauuggauacuaggaauacuaucaauggauccaacaucuucuaga | manual\_scaffold\_1b:1008540928..1008540983:+ |
| manual\_scaffold\_5\_18582 | 1.4 | 0.91 ± 0.01 |  | 90 | 90 | 0 | 0 | yes |  |  |  | blast | cuagcacugcucuuaugcuguu | cagcaagacagcaguacguggac | cuagcacugcucuuaugcuguuuagcauuguaagcagcaagacagcaguacguggac | manual\_scaffold\_5:1292766893..1292766950:+ |
| manual\_scaffold\_3a\_9346 | 1.4 | 0.91 ± 0.01 |  | 55 | 55 | 0 | 0 | yes |  |  |  | blast | aaugcacuggaaacugcaccu | gugccgauuccagcacaucucu | gugccgauuccagcacaucucuuugaacaaagaaugcacuggaaacugcaccu | manual\_scaffold\_3a:393329709..393329762:- |
| manual\_scaffold\_6\_21681 | 1.4 | 0.91 ± 0.01 |  | 14 | 14 | 0 | 0 | yes |  |  |  | blast | ugcggcacgaggacuccuagugg | acuuggagucucgugcagccgu | acuuggagucucgugcagccgugagguuagcugcggcacgaggacuccuagugg | manual\_scaffold\_6:872418068..872418122:+ |
| manual\_scaffold\_11\_37292 | 1.4 | 0.91 ± 0.01 |  | 18 | 18 | 0 | 0 | yes |  |  |  | blast | aaaaucggacuccaguaacaaa | cguuacuguaggcugauugugc | cguuacuguaggcugauugugcagcaguguugaaaaaucggacuccaguaacaaa | manual\_scaffold\_11:541015192..541015247:- |
| manual\_scaffold\_6\_21854 | 1.4 | 0.91 ± 0.01 |  | 11 | 8 | 3 | 0 | yes |  |  |  | blast | cucgggagggugaccuugcucugcc | cagugcagugccccccguugcccccagca | cucgggagggugaccuugcucugcccacgagaauguugugggcagugcagugccccccguugcccccagca | manual\_scaffold\_6:1064341205..1064341276:+ |
| manual\_scaffold\_11\_35965 | 1.4 | 0.91 ± 0.01 |  | 14 | 14 | 0 | 0 | yes |  |  |  | blast | uaccaggugcuguaagcuu | gcauuauagugccugguuagu | gcauuauagugccugguuaguaacuuggugggcgaccauaugggaguaccaggugcuguaagcuu | manual\_scaffold\_11:114892228..114892293:+ |
| manual\_scaffold\_8\_28795 | 1.4 | 0.91 ± 0.01 |  | 997 | 736 | 0 | 261 | yes |  |  |  | blast | uagcggaugcaggguucagau | gucuaggcaugcaucuguugga | uagcggaugcaggguucagaucaucauuaaagucuaggcaugcaucuguugga | manual\_scaffold\_8:304152835..304152888:+ |
| manual\_scaffold\_7\_27500 | 1.4 | 0.91 ± 0.01 |  | 20 | 20 | 0 | 0 | yes |  |  |  | blast | ugauaaccuuuagcgagcugu | gggucacuaagggugcucaau | gggucacuaagggugcucaauguuacagaugggaauugauaaccuuuagcgagcugu | manual\_scaffold\_7:983511144..983511201:- |
| manual\_scaffold\_9\_33268 | 1.4 | 0.91 ± 0.01 |  | 15 | 15 | 0 | 0 | yes |  |  |  | blast | cuuccgagguagauaaaaug | uuuggcgcaacuuuagauugu | uuuggcgcaacuuuagauuguggaagauccacucagaccuuccuccuuccgagguagauaaaaug | manual\_scaffold\_9:1193028237..1193028302:- |
| manual\_scaffold\_1a\_1592 | 1.4 | 0.91 ± 0.01 |  | 43 | 43 | 0 | 0 | yes |  |  |  | blast | auccuggcgcuucucugaugcu | uguccccgggagcgcugagagag | uguccccgggagcgcugagagagaaugcuucuuccaauccuggcgcuucucugaugcu | manual\_scaffold\_1a:964687907..964687965:- |
| manual\_scaffold\_10\_35453 | 1.4 | 0.91 ± 0.01 |  | 525 | 525 | 0 | 0 | yes |  |  |  | blast | ccaggacuccucgcucacugcu | cagugaucguuuaguccagag | ccaggacuccucgcucacugcuuguaaucuaagcagugaucguuuaguccagag | manual\_scaffold\_10:819042413..819042467:- |
| manual\_scaffold\_6\_22870 | 1.4 | 0.91 ± 0.01 |  | 36 | 34 | 0 | 2 | yes |  |  |  | blast | cacuuugugaaucuguaacaccu | aagcuguuacucgcacaguggaa | aagcuguuacucgcacaguggaagauguccaucauggcuuccacuuugugaaucuguaacaccu | manual\_scaffold\_6:155821557..155821621:- |
| manual\_scaffold\_12\_38142 | 1.4 | 0.91 ± 0.01 |  | 39 | 39 | 0 | 0 | yes |  |  |  | blast | ugugcccagaacaucaguugga | cagcugguggaacuagguacaug | ugugcccagaacaucaguuggagcuaaccaucggguaauucagcugguggaacuagguacaug | manual\_scaffold\_12:225273836..225273899:+ |
| manual\_scaffold\_12\_39367 | 1.4 | 0.91 ± 0.01 |  | 21 | 21 | 0 | 0 | yes |  |  |  | blast | acgcaccuguggacauuugaa | cauggacucaggugcaugc | acgcaccuguggacauuugaaguuuuauaugaaagaaaaaucauuaacucauggacucaggugcaugc | manual\_scaffold\_12:477957403..477957471:- |
| manual\_scaffold\_1a\_993 | 1.4 | 0.91 ± 0.01 |  | 10 | 10 | 0 | 0 | yes |  |  |  | blast | uugcuugcugcacuguacgacu | ucgaaguacagcgcagcaagcucca | uugcuugcugcacuguacgacuuuuucaaaaagucgaaguacagcgcagcaagcucca | manual\_scaffold\_1a:140322286..140322344:- |
| manual\_scaffold\_4b\_16999 | 1.4 | 0.91 ± 0.01 |  | 50 | 45 | 0 | 5 | yes |  |  |  | blast | uccagacucuuuacucaggcu | agcaugagaacagugucaggauu | uccagacucuuuacucaggcuacugauaaugcuagcagcaugagaacagugucaggauu | manual\_scaffold\_4b:655640768..655640827:- |
| manual\_scaffold\_7\_27135 | 1.4 | 0.91 ± 0.01 |  | 36 | 36 | 0 | 0 | yes |  |  |  | blast | cacuaggacucucucaggcucu | aguuuuggagcggucuaguggu | cacuaggacucucucaggcucugugguauaucuauuggaguuuuggagcggucuaguggu | manual\_scaffold\_7:595969207..595969267:- |
| manual\_scaffold\_7\_24712 | 1.4 | 0.91 ± 0.01 |  | 33 | 33 | 0 | 0 | yes |  |  |  | blast | ugugacgucucugcaggagagu | ugaaucugcaugugacgucucugc | ugugacgucucugcaggagagugcuccauucacugguacugaaucugcaugugacgucucugc | manual\_scaffold\_7:80531232..80531295:+ |
| manual\_scaffold\_4b\_16567 | 1.4 | 0.91 ± 0.01 |  | 574 | 447 | 0 | 127 | yes |  |  |  | blast | gaaaaagcuuucguacauauggc | gauguacgcaaggauuuuaccc | gauguacgcaaggauuuuacccauucugugucuaugggaaaaagcuuucguacauauggc | manual\_scaffold\_4b:141514281..141514341:- |
| manual\_scaffold\_1b\_2606 | 1.4 | 0.91 ± 0.01 |  | 800 | 763 | 0 | 37 | yes |  |  |  | blast | ugucaguguaauguagcugagu | aacuacaucacaccgacgagcc | ugucaguguaauguagcugaguucaucucaaugcaugucaacuacaucacaccgacgagcc | manual\_scaffold\_1b:988077865..988077926:+ |
| manual\_scaffold\_11\_36320 | 1.4 | 0.91 ± 0.01 |  | 1445 | 1401 | 3 | 41 | yes |  |  |  | blast | aguaccacauuucugaugcug | cagcaucugaguaggugguacuuc | aguaccacauuucugaugcugaaaugccaucuacagaauugcagcaucugaguaggugguacuuc | manual\_scaffold\_11:623491349..623491414:+ |
| manual\_scaffold\_6\_20847 | 1.4 | 0.91 ± 0.01 |  | 22 | 22 | 0 | 0 | yes |  |  |  | blast | ucaggacuacuguucccagagccu | auucugggaucaguaguccuguuu | auucugggaucaguaguccuguuuauucauauuaagcaaucaggacuacuguucccagagccu | manual\_scaffold\_6:152271197..152271260:+ |
| manual\_scaffold\_2a\_4682 | 1.4 | 0.91 ± 0.01 |  | 15 | 15 | 0 | 0 | yes |  |  |  | blast | cuagggggcacaaaaaaa | uuaauaaaugugcccuuuggua | cuagggggcacaaaaaaaguggcucugcaauaggugcagcaccacauuuaauaaaugugcccuuuggua | manual\_scaffold\_2a:1155934681..1155934750:+ |
| manual\_scaffold\_7\_28318 | 1.4 | 0.91 ± 0.01 |  | 17 | 17 | 0 | 0 | yes |  |  |  | blast | ucugagcuugcaucuguugga | cuuguggaugcaggguucagaua | cuuguggaugcaggguucagauaaucaguugaagucugagcuugcaucuguugga | manual\_scaffold\_7:1578135641..1578135696:- |
| manual\_scaffold\_4b\_17083 | 1.4 | 0.91 ± 0.01 |  | 1278 | 1278 | 0 | 0 | yes |  |  |  | blast | cccaucaacuguauuacaagu | cuguauuacgaaggaugagag | cccaucaacuguauuacaagugccauagccuauauugcacuguauuacgaaggaugagag | manual\_scaffold\_4b:751817039..751817099:- |
| manual\_scaffold\_4b\_15175 | 1.4 | 0.91 ± 0.01 |  | 11 | 11 | 0 | 0 | yes |  |  |  | blast | ucgggcccagagacacuuguau | ucugguaucucccggcccagaau | ucugguaucucccggcccagaauguuuagaccucguaaauuucgggcccagagacacuuguau | manual\_scaffold\_4b:309465554..309465617:+ |
| manual\_scaffold\_1b\_2962 | 1.4 | 0.91 ± 0.01 |  | 771 | 771 | 0 | 0 | yes |  |  |  | blast | ccucugggacuccuucug | gggaguuccuguuggagguu | gggaguuccuguuggagguuuuuuccccuuugggguuuuuccucugggacuccuucug | manual\_scaffold\_1b:116077511..116077569:- |
| manual\_scaffold\_6\_20951 | 1.4 | 0.91 ± 0.01 |  | 17 | 17 | 0 | 0 | yes |  |  |  | blast | uuggaugcagggucuagucu | auguucuuacguccagcc | auguucuuacguccagccccuacaugucagaggcuguggccuuaugcagucgguguuggaugcagggucuagucu | manual\_scaffold\_6:202959952..202960027:+ |
| manual\_scaffold\_9\_32675 | 1.4 | 0.91 ± 0.01 |  | 18 | 18 | 0 | 0 | yes |  |  |  | blast | cacuggaaacucuacauagugu | acuauauagagagccagcuuc | cacuggaaacucuacauaguguauuucauuuuagugcacuauauagagagccagcuuc | manual\_scaffold\_9:570500829..570500887:- |
| manual\_scaffold\_12\_37886 | 1.4 | 0.91 ± 0.01 |  | 33 | 33 | 0 | 0 | yes |  |  |  | blast | auagccaagacacgcugaauuucu | agauucagcgaguuuggguacca | auagccaagacacgcugaauuucuucugcaagacugaacaagauucagcgaguuuggguacca | manual\_scaffold\_12:50271951..50272014:+ |
| manual\_scaffold\_4b\_15879 | 1.4 | 0.91 ± 0.01 |  | 188 | 188 | 0 | 0 | yes |  |  |  | blast | ugcauuuguaguaagguggaca | ucagccuuauuacaagcacc | ucagccuuauuacaagcaccauaggauauaaugcauuuguaguaagguggaca | manual\_scaffold\_4b:1079524512..1079524565:+ |
| manual\_scaffold\_2a\_5539 | 1.4 | 0.91 ± 0.01 |  | 46 | 38 | 0 | 8 | yes |  |  |  | blast | acaauucuggaacuuguagucc | aaggcccagaauuuuaagaaaa | acaauucuggaacuuguaguccagcauuauuacuguguuaaaaacgacuacaaggcccagaauuuuaagaaaa | manual\_scaffold\_2a:832423440..832423513:- |
| manual\_scaffold\_5\_19820 | 1.4 | 0.91 ± 0.01 |  | 14 | 14 | 0 | 0 | yes |  |  |  | blast | uguuucacugagcugccccaccu | cuggaggcaguugauggaacuag | cuggaggcaguugauggaacuagauuguuguuacuuguuucacugagcugccccaccu | manual\_scaffold\_5:835156659..835156717:- |
| manual\_scaffold\_1a\_79 | 1.4 | 0.91 ± 0.01 |  | 13 | 13 | 0 | 0 | yes |  |  |  | blast | uucacauaugcauaaacggcu | gcauuuaugauuaugugaagu | gcauuuaugauuaugugaaguuuugcugcaggaauauacuucacauaugcauaaacggcu | manual\_scaffold\_1a:84963295..84963355:+ |
| manual\_scaffold\_5\_18145 | 1.4 | 0.91 ± 0.01 |  | 27 | 27 | 0 | 0 | yes |  |  |  | blast | uaccucaggcuaaggacucuugu | gagcuuccuuagaaugagggaua | gagcuuccuuagaaugagggauaucgagcaaucauggauaccucaggcuaaggacucuugu | manual\_scaffold\_5:678199502..678199563:+ |
| manual\_scaffold\_10\_33475 | 1.4 | 0.91 ± 0.01 |  | 1754 | 1752 | 0 | 2 | no |  | xtr-miR-428a |  | blast | aaagugcugucuaguuugggcu | cgcccacaccggggcgcucucu | cgcccacaccggggcgcucucuccuugcaauggaaagugcugucuaguuugggcu | manual\_scaffold\_10:48275295..48275350:+ |
| manual\_scaffold\_4a\_13113 | 1.4 | 0.91 ± 0.01 |  | 12 | 12 | 0 | 0 | yes |  |  |  | blast | uuucauuuuccauuuuccug | ggaaaauggaaaauuaaagu | ggaaaauggaaaauuaaaguaaaauuuacuuucauuuuccauuuuccug | manual\_scaffold\_4a:519043477..519043526:+ |
| manual\_scaffold\_9\_32685 | 1.4 | 0.91 ± 0.01 |  | 89 | 89 | 0 | 0 | yes |  |  |  | blast | ucaggcauaauccacagacacc | uguuggggauuguuccuuguag | ucaggcauaauccacagacaccuauuacuucuuuuggguguuggggauuguuccuuguag | manual\_scaffold\_9:584378655..584378715:- |
| manual\_scaffold\_5\_19741 | 1.4 | 0.91 ± 0.01 |  | 43 | 42 | 0 | 1 | yes |  |  |  | blast | cagcagacacauccauuggacu | uagccuauggcugugucu | cagcagacacauccauuggacugugaccagcacuggcaacaacauuagccuauggcugugucu | manual\_scaffold\_5:757724214..757724277:- |
| manual\_scaffold\_2b\_6094 | 1.4 | 0.91 ± 0.01 |  | 33 | 33 | 0 | 0 | yes |  |  |  | blast | uacacgucaucauugucgaug | ucgucauuguugacgguaga | uacacgucaucauugucgauggcaguaacaaugguguccgcgucgucauuguugacgguaga | manual\_scaffold\_2b:237332359..237332421:+ |
| manual\_scaffold\_1b\_3388 | 1.4 | 0.91 ± 0.01 |  | 29 | 29 | 0 | 0 | yes |  |  |  | blast | aaagcccggaugaagauaaga | uuaccuucaccuuuggguuucag | uuaccuucaccuuuggguuucaguguuuuugauuauuuuugaaagcccggaugaagauaaga | manual\_scaffold\_1b:664691353..664691415:- |
| manual\_scaffold\_1a\_1182 | 1.4 | 0.91 ± 0.01 |  | 26 | 26 | 0 | 0 | yes |  |  |  | blast | cuugcgugaucaaggcacag | guggguuaaccacgcaugug | guggguuaaccacgcaugugcugaacaacucacuugcgugaucaaggcacag | manual\_scaffold\_1a:386843127..386843179:- |
| manual\_scaffold\_8\_30044 | 1.4 | 0.91 ± 0.01 |  | 10 | 10 | 0 | 0 | yes |  |  |  | blast | uagggccuagcgguguucugcu | uagacugccgccagcuguauu | uagacugccgccagcuguauuaugagaaauaauagggccuagcgguguucugcu | manual\_scaffold\_8:402485719..402485773:- |
| manual\_scaffold\_10\_34005 | 1.4 | 0.91 ± 0.01 |  | 10 | 10 | 0 | 0 | yes |  |  |  | blast | uggccagagugacaugcacacu | ugugaaugucacauuucucggcgcaa | ugugaaugucacauuucucggcgcaaagcugcuggccagagugacaugcacacu | manual\_scaffold\_10:594621439..594621493:+ |
| manual\_scaffold\_2a\_4497 | 1.4 | 0.91 ± 0.01 |  | 11 | 11 | 0 | 0 | yes |  |  |  | blast | caauucuguaugguggucuucu | aaaaagaccgcccacagacuuaca | aaaaagaccgcccacagacuuacaaccacaauucuguaugguggucuucu | manual\_scaffold\_2a:863360689..863360739:+ |
| manual\_scaffold\_10\_35588 | 1.4 | 0.91 ± 0.01 |  | 91 | 83 | 0 | 8 | yes |  |  |  | blast | caccuggccauucuuuaacucu | caguuuagagcugccaagaggu | caccuggccauucuuuaacucugugcuauaacggcuaguacaguuuagagcugccaagaggu | manual\_scaffold\_10:943428660..943428722:- |
| manual\_scaffold\_6\_24066 | 1.4 | 0.91 ± 0.01 |  | 29 | 29 | 0 | 0 | yes |  |  |  | blast | uuggaacaccaucgacaugac | uguggcgguugguggcucagcc | uguggcgguugguggcucagccucaaagcauacaaugguuggaacaccaucgacaugac | manual\_scaffold\_6:1150879559..1150879618:- |
| manual\_scaffold\_9\_32818 | 1.4 | 0.91 ± 0.01 |  | 20 | 19 | 0 | 1 | yes |  |  |  | blast | acacaaacaacuaugaacggu | accguucguagucguuuauguu | accguucguagucguuuauguuguuuauuaauccaacauacaucacaacacaaacaacuaugaacggu | manual\_scaffold\_9:770105378..770105446:- |
| manual\_scaffold\_6\_23872 | 1.4 | 0.91 ± 0.01 |  | 18 | 18 | 0 | 0 | yes |  |  |  | blast | auucucuggccugugauugacu | uaagucgcaugccaaggaacaa | uaagucgcaugccaaggaacaauaaaauaaaguauucucuggccugugauugacu | manual\_scaffold\_6:930241901..930241956:- |
| manual\_scaffold\_9\_31825 | 1.4 | 0.91 ± 0.01 |  | 53 | 53 | 0 | 0 | yes |  |  |  | blast | gacuuugguguggccuggaagu | ggcugggccacucgaaaguucag | ggcugggccacucgaaaguucagcgcuauuacagcaucugacuuugguguggccuggaagu | manual\_scaffold\_9:893984604..893984665:+ |
| manual\_scaffold\_12\_39451 | 1.4 | 0.91 ± 0.01 |  | 56 | 56 | 0 | 0 | yes |  |  |  | blast | uuuguagagccuuggauaacu | uuauucaaagcucugcaugca | uuuguagagccuuggauaacugcacgcaagcuaguuauucaaagcucugcaugca | manual\_scaffold\_12:539199705..539199760:- |
| manual\_scaffold\_1b\_1934 | 1.4 | 0.91 ± 0.01 |  | 20 | 20 | 0 | 0 | yes |  |  |  | blast | aaauugugauuacuugauugca | caauuagguaauugcuaauugg | aaauugugauuacuugauugcaguguuuugcaaaaugcaauuagguaauugcuaauugg | manual\_scaffold\_1b:170617506..170617565:+ |
| manual\_scaffold\_4a\_12775 | 1.4 | 0.91 ± 0.01 |  | 17 | 17 | 0 | 0 | yes |  |  |  | blast | uggauaauuucugcucuucccu | gagagggcaggaaugauccaua | gagagggcaggaaugauccauauuuacaaggguauggauaauuucugcucuucccu | manual\_scaffold\_4a:96064062..96064118:+ |
| manual\_scaffold\_8\_29759 | 1.4 | 0.91 ± 0.01 |  | 36 | 36 | 0 | 0 | yes |  |  |  | blast | cuugaaccugagccuaugcugg | ggcauagccagguucauuuu | cuugaaccugagccuaugcuggagcccacacagcaagccuaggcauagccagguucauuuu | manual\_scaffold\_8:67812829..67812890:- |
| manual\_scaffold\_7\_25907 | 1.4 | 0.91 ± 0.01 |  | 13 | 12 | 0 | 1 | yes |  |  |  | blast | aaagcagcgucagaauuuguua | acuaauccugacguugcuuuc | acuaauccugacguugcuuucaugaugguuaccagcacgaaagcagcgucagaauuuguua | manual\_scaffold\_7:1253484220..1253484281:+ |
| manual\_scaffold\_6\_22047 | 1.3 | 0.91 ± 0.01 |  | 335 | 335 | 0 | 0 | yes |  |  |  | blast | ugcgugaggacaagaccaacuagu | gagguggucucauccucagcaac | gagguggucucauccucagcaacauugaggcuggcugcgugaggacaagaccaacuagu | manual\_scaffold\_6:1242624019..1242624078:+ |
| manual\_scaffold\_1a\_1643 | 1.3 | 0.91 ± 0.01 |  | 14 | 14 | 0 | 0 | yes |  |  |  | blast | auccugggacugcucuuaugcu | caugauagcagagcuuggaucu | auccugggacugcucuuaugcuuuuuaguauuauaagcaacaugauagcagagcuuggaucu | manual\_scaffold\_1a:1049475534..1049475596:- |
| manual\_scaffold\_10\_33889 | 1.3 | 0.91 ± 0.01 |  | 51 | 51 | 0 | 0 | yes |  |  |  | blast | uacuaggugcuguaggcac | auguacagcacccuguaaa | auguacagcacccuguaaauacagauuaccuuaggugagguauuuacuaggugcuguaggcac | manual\_scaffold\_10:429723361..429723424:+ |
| manual\_scaffold\_3a\_7570 | 1.3 | 0.91 ± 0.01 |  | 213209 | 165550 | 13 | 47646 | yes |  |  |  | blast | ccggauaucaucauauacugua | uacaguauagaugauguacu | ccggauaucaucauauacuguaaguuuguaauaaggcacuacaguauagaugauguacu | manual\_scaffold\_3a:298508040..298508099:+ |
| manual\_scaffold\_3b\_11328 | 1.3 | 0.91 ± 0.01 |  | 15 | 15 | 0 | 0 | yes |  |  |  | blast | ucauauacucuuaaaugccu | gcauguaugaguauuugaaa | gcauguaugaguauuugaaaagguacuacugcagucuauuucauauacucuuaaaugccu | manual\_scaffold\_3b:294307267..294307327:- |
| manual\_scaffold\_7\_25758 | 1.3 | 0.91 ± 0.01 |  | 92 | 92 | 0 | 0 | yes |  |  |  | blast | uucuagaaguuguuggauac | auccaaauucuccuaaauuu | uucuagaaguuguuggauaccaggaguucuaucacuggauccaaauucuccuaaauuu | manual\_scaffold\_7:1097527378..1097527436:+ |
| manual\_scaffold\_8\_30528 | 1.3 | 0.91 ± 0.01 |  | 3483 | 2584 | 1 | 898 | yes |  |  |  | blast | uguauuggaacacuacagcu | aguuguagucuuucaaacaga | aguuguagucuuucaaacagagagcuguaaguguauaccuguauuggaacacuacagcu | manual\_scaffold\_8:1156361820..1156361879:- |
| manual\_scaffold\_2b\_6735 | 1.3 | 0.91 ± 0.01 |  | 11 | 11 | 0 | 0 | yes |  |  |  | blast | aagaagcaucuggucuuggcuc | gccaagaccagaugcuucuuuugu | aagaagcaucuggucuuggcucuaugcauuugcauuucuuacagccaagaccagaugcuucuuuugu | manual\_scaffold\_2b:285811829..285811896:- |
| manual\_scaffold\_6\_22371 | 1.3 | 0.91 ± 0.01 |  | 31 | 26 | 0 | 5 | yes |  |  |  | blast | cagcacugucuaguaagaug | caucguuaccagacaguguuaga | caucguuaccagacaguguuagagaaguguuauaauauccagcacugucuaguaagaug | manual\_scaffold\_6:1580596796..1580596855:+ |
| manual\_scaffold\_9\_31422 | 1.3 | 0.91 ± 0.01 |  | 108 | 91 | 17 | 0 | yes |  |  |  | blast | ccucuggcacuacuucug | gaaagagucgccccuacca | ccucuggcacuacuucugacggacacggccugcucuuggcguacccauugccugcagcaccguggcuaccagaaagagucgccccuacca | manual\_scaffold\_9:564441733..564441823:+ |
| manual\_scaffold\_3b\_10702 | 1.3 | 0.91 ± 0.01 |  | 23 | 23 | 0 | 0 | yes |  |  |  | blast | ccccagcggaugcagaguuc | gcacacauuugugggaggg | ccccagcggaugcagaguucagauaaucaguuaaagucugagcacacauuugugggaggg | manual\_scaffold\_3b:804089982..804090042:+ |
| manual\_scaffold\_2a\_3978 | 1.3 | 0.91 ± 0.01 |  | 22 | 17 | 2 | 3 | yes |  |  |  | blast | cgaggcugcguguucauaucaug | uuguguggagacgcccgucaggga | cgaggcugcguguucauaucauggagauuuguguugaaaauaauacuguucgcuccauuguguggagacgcccgucaggga | manual\_scaffold\_2a:198225031..198225112:+ |
| manual\_scaffold\_1b\_3681 | 1.3 | 0.91 ± 0.01 |  | 612 | 457 | 155 | 0 | yes |  |  |  | blast | ccuaauccccuuuucccc | ggacauguugggaacguaggca | ccuaauccccuuuuccccugcuuggacucucucagacucugugauauuucuaucagaguuuuggagcugucuagugguggacauguugggaacguaggca | manual\_scaffold\_1b:1064445940..1064446040:- |
| manual\_scaffold\_3b\_11301 | 1.3 | 0.91 ± 0.01 |  | 14 | 14 | 0 | 0 | yes |  |  |  | blast | uacacuagggcccagaagaac | ucuucugggcccaagugcaca | uacacuagggcccagaagaacacaucuguagcugagauuuuuggaucuucugggcccaagugcaca | manual\_scaffold\_3b:260395140..260395206:- |
| manual\_scaffold\_10\_34433 | 1.3 | 0.91 ± 0.01 |  | 13 | 13 | 0 | 0 | yes |  |  |  | blast | ucauccaugacaguuaaggccu | gucuuacuggcauguggaugugc | ucauccaugacaguuaaggccucuggauuggaagaguggggucuuacuggcauguggaugugc | manual\_scaffold\_10:1155646..1155709:- |
| manual\_scaffold\_7\_24715 | 1.3 | 0.91 ± 0.01 |  | 83 | 83 | 0 | 0 | yes |  |  |  | blast | cagcugggauaucucugagaga | ucucagugaugucccacuuag | ucucagugaugucccacuuaggauauccugccagcugggauaucucugagaga | manual\_scaffold\_7:80551236..80551289:+ |
| manual\_scaffold\_4b\_16652 | 1.3 | 0.91 ± 0.01 |  | 55 | 55 | 0 | 0 | yes |  |  |  | blast | acccagcaccccguucuacccc | aguggaauugggaguggugugg | aguggaauugggagugguguggcccugcaaaccccccacccagcaccccguucuacccc | manual\_scaffold\_4b:180469507..180469566:- |
| manual\_scaffold\_3a\_9598 | 1.3 | 0.91 ± 0.01 |  | 11 | 10 | 0 | 1 | yes |  |  |  | blast | uuggcugaaguauaaugccacu | aguggcaugucucuuucagc | aguggcaugucucuuucagccagugugcugugcucauaaauucguuggcugaaguauaaugccacu | manual\_scaffold\_3a:548548578..548548644:- |
| manual\_scaffold\_9\_33223 | 1.3 | 0.91 ± 0.01 |  | 13 | 13 | 0 | 0 | yes |  |  |  | blast | ucuggccaagcuuccaccauu | uggggaggagacuggccaagcu | ucuggccaagcuuccaccauucuuagugacaucauaaauggggaggagacuggccaagcu | manual\_scaffold\_9:1164190439..1164190499:- |
| manual\_scaffold\_12\_39342 | 1.3 | 0.91 ± 0.01 |  | 25 | 25 | 0 | 0 | yes |  |  |  | blast | cacaauuuggucaucgcuauug | auagcgauuacuuaauugcgag | auagcgauuacuuaauugcgagucacaaaaaaucacaauuuggucaucgcuauug | manual\_scaffold\_12:436688038..436688093:- |
| manual\_scaffold\_2a\_4138 | 1.3 | 0.91 ± 0.01 |  | 17 | 17 | 0 | 0 | yes |  |  |  | blast | uaccaggugcuguagucc | acugaggaagcaguggc | uaccaggugcuguaguccuugcaagugaagacaaaggacugaggaagcaguggc | manual\_scaffold\_2a:429665008..429665062:+ |
| manual\_scaffold\_8\_30253 | 1.3 | 0.91 ± 0.01 |  | 67 | 67 | 0 | 0 | yes |  |  |  | blast | ccgagcaaugcugucaugcug | gcauaaaagcagugccaugau | ccgagcaaugcugucaugcugcuuuuaauaccaaacagcauaaaagcagugccaugau | manual\_scaffold\_8:742268958..742269016:- |
| manual\_scaffold\_3a\_7634 | 1.3 | 0.91 ± 0.01 |  | 126 | 126 | 0 | 0 | yes |  |  |  | blast | accugggcacucuacuacucugu | agaguuggaggugccuggagga | accugggcacucuacuacucugugcuucaaagguuuccagaguuggaggugccuggagga | manual\_scaffold\_3a:333868081..333868141:+ |
| manual\_scaffold\_3b\_10296 | 1.3 | 0.91 ± 0.01 |  | 965 | 638 | 327 | 0 | yes |  |  |  | blast | ccuaauccccuuuucccc | ggaaguguugggaa | ccuaauccccuuuuccccacuuagacucuccuagacucugugauaaaucuaaucagaguauuggagcugucuugugguggaaguguugggaa | manual\_scaffold\_3b:346936242..346936334:+ |
| manual\_scaffold\_7\_24714 | 1.3 | 0.91 ± 0.01 |  | 33 | 33 | 0 | 0 | yes |  |  |  | blast | ugugacgucucugcaggagagu | ugaaucugcaugagaccucucugc | ugugacgucucugcaggagagugcuccauucacugguacugaaucugcaugagaccucucugc | manual\_scaffold\_7:80531379..80531442:+ |
| manual\_scaffold\_3a\_8564 | 1.3 | 0.91 ± 0.01 |  | 245 | 245 | 0 | 0 | yes |  |  |  | blast | ccguaggacuuguagucuugc | aagacuauaagucccauaauu | ccguaggacuuguagucuugcuguauaauggaauaaacaagacuauaagucccauaauu | manual\_scaffold\_3a:848397668..848397727:+ |
| manual\_scaffold\_5\_18357 | 1.3 | 0.91 ± 0.01 |  | 19 | 19 | 0 | 0 | yes |  |  |  | blast | ucggagcacgcaucuguugga | uagugggugcaggguucagaca | uagugggugcaggguucagacaaucaguaaagucggagcacgcaucuguugga | manual\_scaffold\_5:982123899..982123952:+ |
| manual\_scaffold\_5\_18569 | 1.3 | 0.91 ± 0.01 |  | 14 | 14 | 0 | 0 | yes |  |  |  | blast | ugcauuuguaguaagguagaca | cccaccuuaagugcaagugccaua | cccaccuuaagugcaagugccauaggauaaaaugcauuuguaguaagguagaca | manual\_scaffold\_5:1277653429..1277653483:+ |
| manual\_scaffold\_8\_29120 | 1.3 | 0.91 ± 0.01 |  | 166 | 163 | 0 | 3 | yes |  |  |  | blast | acaaaguaucaacaucgggucu | aagauacuugugaggccu | acaaaguaucaacaucgggucuguaaucuguguuugucaccagaccaccgagaagauacuugugaggccu | manual\_scaffold\_8:842645844..842645914:+ |
| manual\_scaffold\_5\_20051 | 1.3 | 0.91 ± 0.01 |  | 55 | 55 | 0 | 0 | yes |  |  |  | blast | ucugggacaaguagucuuguuu | acaggccugcaggucuuagaau | ucugggacaaguagucuuguuuauuccauaguaaaacaggccugcaggucuuagaau | manual\_scaffold\_5:1170025659..1170025716:- |
| manual\_scaffold\_3a\_9855 | 1.3 | 0.91 ± 0.01 |  | 45 | 45 | 0 | 0 | yes |  |  |  | blast | uagaucucacuuugaaaaggg | ccaaacugagauccagu | uagaucucacuuugaaaagggggaaaucauuguugguacccccaaacugagauccagu | manual\_scaffold\_3a:845229877..845229935:- |
| manual\_scaffold\_5\_17986 | 1.3 | 0.91 ± 0.01 |  | 71 | 71 | 0 | 0 | yes |  |  |  | blast | uccgucauacugcaagugcaau | ugcacuuguaauauggcagaca | uccgucauacugcaagugcaauaggauauaaugcacuuguaauauggcagaca | manual\_scaffold\_5:454631503..454631556:+ |
| manual\_scaffold\_3a\_8897 | 1.3 | 0.91 ± 0.01 |  | 11 | 11 | 0 | 0 | yes |  |  |  | blast | ggugaugagcccuaggauuggcu | ccaauccuagguaacauuacac | ggugaugagcccuaggauuggcuggggauauacaagccaauccuagguaacauuacac | manual\_scaffold\_3a:199151350..199151408:- |
| manual\_scaffold\_8\_29328 | 1.3 | 0.91 ± 0.01 |  | 25 | 25 | 0 | 0 | yes |  |  |  | blast | ugcaggacugaucggcagugc | acugccuaucacuccucacgccagu | ugcaggacugaucggcagugcagugcaacaucaugcacugccuaucacuccucacgccagu | manual\_scaffold\_8:1146275949..1146276010:+ |
| manual\_scaffold\_7\_25924 | 1.3 | 0.91 ± 0.01 |  | 25 | 25 | 0 | 0 | yes |  |  |  | blast | acugugcauguccgaucggcca | gccagccugacaugcucaguuu | acugugcauguccgaucggccaguguuuuugcuguggccagccugacaugcucaguuu | manual\_scaffold\_7:1274135384..1274135442:+ |
| manual\_scaffold\_1b\_2030 | 1.3 | 0.91 ± 0.01 |  | 128 | 128 | 0 | 0 | yes |  |  |  | blast | ggguugcauucagaagaug | ccuccugauggcguccuu | ggguugcauucagaagaugcacugccugugaaccaccuuuuuguggcacaaagucaguguaccuccugauggcguccuu | manual\_scaffold\_1b:263370745..263370824:+ |
| manual\_scaffold\_8\_30499 | 1.3 | 0.91 ± 0.01 |  | 28 | 28 | 0 | 0 | yes |  |  |  | blast | aaguuuuggguuggggcg | ccccuuuuuauuucuuuu | aaguuuuggguuggggcgcggggugcagcaggguugcacgcuagccccuuuuuauuucuuuu | manual\_scaffold\_8:1109844760..1109844822:- |
| manual\_scaffold\_10\_34950 | 1.3 | 0.91 ± 0.01 |  | 111 | 111 | 0 | 0 | yes |  |  |  | blast | gucccuucguggucgcca | gccgaccacgagcuuacug | gccgaccacgagcuuacugucguuggacaucccucgaagaggucccuucguggucgcca | manual\_scaffold\_10:296100284..296100343:- |
| manual\_scaffold\_7\_25173 | 1.3 | 0.91 ± 0.01 |  | 100 | 77 | 0 | 23 | yes |  |  |  | blast | augaaugcucacaugaugacu | ugucaucaagugagcaugcacu | ugucaucaagugagcaugcacugcaguguagaaaugugaugaaugcucacaugaugacu | manual\_scaffold\_7:561811649..561811708:+ |
| manual\_scaffold\_3a\_8844 | 1.3 | 0.91 ± 0.01 |  | 44 | 44 | 0 | 0 | yes |  |  |  | blast | auccugagagacuccauacu | gauggagaccugacaggaccu | gauggagaccugacaggaccuaccugcuguugcuguaauccugagagacuccauacu | manual\_scaffold\_3a:129988310..129988367:- |
| manual\_scaffold\_9\_33062 | 1.3 | 0.91 ± 0.01 |  | 10 | 10 | 0 | 0 | yes |  |  |  | blast | agcauaaagcagugcuaggauu | uccaagcauugcucuuaugcugc | uccaagcauugcucuuaugcugcaaguaauacuaagcagcauaaagcagugcuaggauu | manual\_scaffold\_9:1003560662..1003560721:- |
| manual\_scaffold\_3a\_9901 | 1.2 | 0.91 ± 0.01 |  | 78 | 74 | 0 | 4 | yes |  |  |  | blast | ccccagcggaugcagaguucagu | ucugagcacgcauccauuggag | ccccagcggaugcagaguucaguuaaucaguaaagucugagcacgcauccauuggag | manual\_scaffold\_3a:892789809..892789866:- |
| manual\_scaffold\_6\_23710 | 1.2 | 0.91 ± 0.01 |  | 771 | 771 | 0 | 0 | yes |  |  |  | blast | ccucugggacuccuucug | gggaguuccuguuggagguu | gggaguuccuguuggagguuuuuucccccuuugggguuuuuccucugggacuccuucug | manual\_scaffold\_6:746821750..746821809:- |
| manual\_scaffold\_7\_27194 | 1.2 | 0.91 ± 0.01 |  | 19517 | 19502 | 0 | 15 | yes |  |  |  | blast | auagcucuuuaaaugguacu | agcgccauuuucacagcuaua | agcgccauuuucacagcuauaaacaguaugagugucauagcucuuuaaaugguacu | manual\_scaffold\_7:668335944..668336000:- |
| manual\_scaffold\_12\_38223 | 1.2 | 0.91 ± 0.01 |  | 771 | 771 | 0 | 0 | yes |  |  |  | blast | ccucugggacuccuucug | gggaguuccuguuggag | gggaguuccuguuggaguuuuuuuuacccuuuugggguuuuuccucugggacuccuucug | manual\_scaffold\_12:345136078..345136138:+ |
| manual\_scaffold\_9\_31748 | 1.2 | 0.91 ± 0.01 |  | 10 | 10 | 0 | 0 | yes |  |  |  | blast | agguucaagucagguuagagu | ucuaaccugacuagagccaau | agguucaagucagguuagagucaaaaauaguggcucuaaccugacuagagccaau | manual\_scaffold\_9:844605812..844605867:+ |
| manual\_scaffold\_2a\_5188 | 1.2 | 0.91 ± 0.01 |  | 18 | 18 | 0 | 0 | yes |  |  |  | blast | cgcagucgcguuaguccucacu | ugaggacuaucgagagggcaau | ugaggacuaucgagagggcaauguacucuucaucgcagucgcguuaguccucacu | manual\_scaffold\_2a:387391204..387391259:- |
| manual\_scaffold\_11\_36704 | 1.2 | 0.91 ± 0.01 |  | 10 | 10 | 0 | 0 | yes |  |  |  | blast | ccuggcacuguucuugcuaccu | gucgcaaauacagugccagaggu | ccuggcacuguucuugcuaccuccgagggcagaggucgcaaauacagugccagaggu | manual\_scaffold\_11:1012192059..1012192116:+ |
| manual\_scaffold\_9\_32574 | 1.2 | 0.91 ± 0.01 |  | 25 | 25 | 0 | 0 | yes |  |  |  | blast | ucacacaucauucuucaaagga | cuuagaagaaagguggccaugaau | cuuagaagaaagguggccaugaaugcauugucaaaauuucacacaucauucuucaaagga | manual\_scaffold\_9:441341412..441341472:- |
| manual\_scaffold\_5\_19557 | 1.2 | 0.91 ± 0.01 |  | 15 | 15 | 0 | 0 | yes |  |  |  | blast | ugcacccucggacuuguaguuuu | agcuguaagacccaggcugcaga | ugcacccucggacuuguaguuuuacuuuuaaaaugauaaagcuguaagacccaggcugcaga | manual\_scaffold\_5:541800167..541800229:- |
| manual\_scaffold\_2b\_7058 | 1.2 | 0.91 ± 0.01 |  | 125 | 125 | 0 | 0 | yes |  |  |  | blast | cuccuggaccacaaggcc | uuuugugaauuucuaggggga | uuuugugaauuucuagggggaccaggguccuccuggaccacaaggcc | manual\_scaffold\_2b:652844965..652845012:- |
| manual\_scaffold\_10\_35191 | 1.2 | 0.91 ± 0.01 |  | 12 | 12 | 0 | 0 | yes |  |  |  | blast | ucuguggugcaugaaccccgacu | uggggaucaacaucaguagc | ucuguggugcaugaaccccgacuugucaugguguaccaaaguggggaucaacaucaguagc | manual\_scaffold\_10:528830624..528830685:- |
| manual\_scaffold\_3a\_9751 | 1.2 | 0.91 ± 0.01 |  | 36 | 16 | 6 | 14 | yes |  |  |  | blast | agucuuggaguuguucauuaga | ucuugaauucucacagacucug | ucuugaauucucacagacucugugaauugucuaucagagucuuggaguuguucauuaga | manual\_scaffold\_3a:703909123..703909182:- |
| manual\_scaffold\_10\_33516 | 1.2 | 0.91 ± 0.01 |  | 15 | 15 | 0 | 0 | yes |  |  |  | blast | ucaacacuguauuaugggacc | gccuaucuuagacaguguuguga | ucaacacuguauuaugggaccccuuuuccugaaguggccuaucuuagacaguguuguga | manual\_scaffold\_10:73540928..73540987:+ |
| manual\_scaffold\_7\_27372 | 1.2 | 0.91 ± 0.01 |  | 36 | 36 | 0 | 0 | yes |  |  |  | blast | accagcacagaacucugc | uuggucuucaaugcgcugguug | uuggucuucaaugcgcugguugucuuuuuuagaccaccagcacagaacucugc | manual\_scaffold\_7:884802766..884802819:- |
| manual\_scaffold\_7\_25618 | 1.2 | 0.91 ± 0.01 |  | 34 | 34 | 0 | 0 | yes |  |  |  | blast | cauccggccacucugauucucu | agagucagugcugccagaaggu | cauccggccacucugauucucuggugauagaauuacagagucagugcugccagaaggu | manual\_scaffold\_7:980558351..980558409:+ |
| manual\_scaffold\_4a\_14440 | 1.2 | 0.91 ± 0.01 |  | 13 | 13 | 0 | 0 | yes |  |  |  | blast | ugcccgaaagacuggucugaca | acagauuuuucagaggcgcc | acagauuuuucagaggcgccagggucggacuggacaauagggcaacccugcauugcccgaaagacuggucugaca | manual\_scaffold\_4a:486005207..486005282:- |
| manual\_scaffold\_3b\_10496 | 1.2 | 0.91 ± 0.01 |  | 71 | 69 | 0 | 2 | yes |  |  |  | blast | ucuagacacugaucuugcacu | caugagagaagugccuggauu | ucuagacacugaucuugcacuguuaauguuaaccagcaugagagaagugccuggauu | manual\_scaffold\_3b:561274090..561274147:+ |
| manual\_scaffold\_6\_21662 | 1.2 | 0.91 ± 0.01 |  | 25 | 25 | 0 | 0 | yes |  |  |  | blast | acaccccuaaagcuguagucgcuc | gaccuacagcucugggguccag | acaccccuaaagcuguagucgcucuuagauacccccagagaccuacagcucugggguccag | manual\_scaffold\_6:863192932..863192993:+ |
| manual\_scaffold\_5\_19220 | 1.2 | 0.91 ± 0.01 |  | 19 | 19 | 0 | 0 | yes |  |  |  | blast | caauggaguaacaaugaacau | gcucauuuuugcuccauggug | gcucauuuuugcuccauggugauaaaguuagcacaauggaguaacaaugaacau | manual\_scaffold\_5:113729932..113729986:- |
| manual\_scaffold\_12\_39963 | 1.2 | 0.91 ± 0.01 |  | 23 | 23 | 0 | 0 | yes |  |  |  | blast | uuaggcugccccucugugagg | uuucaacggggagccuuagg | uuaggcugccccucugugaggaagguaggauuuaacaaauguccuuucaacggggagccuuagg | manual\_scaffold\_12:695235221..695235285:- |
| manual\_scaffold\_1b\_3441 | 1.2 | 0.91 ± 0.01 |  | 47 | 47 | 0 | 0 | yes |  |  |  | blast | cuaacacuguucuuaugcugu | agaaugauagcagugcuuaca | cuaacacuguucuuaugcuguuuggcauuguaagcagaaugauagcagugcuuaca | manual\_scaffold\_1b:747292582..747292638:- |
| manual\_scaffold\_3a\_9745 | 1.2 | 0.91 ± 0.01 |  | 11 | 11 | 0 | 0 | yes |  |  |  | blast | cgccuggcuucaucauuacucugu | ugaguuggugaagccgggaggu | cgccuggcuucaucauuacucugugcucugauagcuuucugaguuggugaagccgggaggu | manual\_scaffold\_3a:684349544..684349605:- |
| manual\_scaffold\_1a\_336 | 1.2 | 0.91 ± 0.01 |  | 33 | 33 | 0 | 0 | yes |  |  |  | blast | cuagcacugccuuugcgaacccu | guggcaaauucagugccagaa | cuagcacugccuuugcgaacccucgcuuuagagguggcaaauucagugccagaa | manual\_scaffold\_1a:394935001..394935055:+ |
| manual\_scaffold\_6\_23896 | 1.2 | 0.91 ± 0.01 |  | 22 | 22 | 0 | 0 | yes |  |  |  | blast | uccguggucugaggagca | cuccuuuaacagccauaagu | cuccuuuaacagccauaaguuaucccccacuccguggucugaggagca | manual\_scaffold\_6:970550118..970550166:- |
| manual\_scaffold\_2a\_5417 | 1.2 | 0.91 ± 0.01 |  | 18 | 18 | 0 | 0 | yes |  |  |  | blast | uaugucuacucucuguaccccu | gugguacagggcuuggacaaguc | uaugucuacucucuguaccccuguagaguggacaagugguacagggcuuggacaaguc | manual\_scaffold\_2a:663736958..663737016:- |
| manual\_scaffold\_10\_34202 | 1.2 | 0.91 ± 0.01 |  | 203 | 177 | 0 | 26 | yes |  |  |  | blast | cacuuggccacuccaauacucu | agaguuggagcugccaggaggu | cacuuggccacuccaauacucugcgcuauuaaggcuuguagaguuggagcugccaggaggu | manual\_scaffold\_10:870364431..870364492:+ |
| manual\_scaffold\_5\_17948 | 1.2 | 0.91 ± 0.01 |  | 11 | 11 | 0 | 0 | yes |  |  |  | blast | cagaacacaucucaacacuacc | gaguguugggagauguucccaa | cagaacacaucucaacacuaccgcacuauaacaggaguguugggagauguucccaa | manual\_scaffold\_5:387229816..387229872:+ |
| manual\_scaffold\_4a\_14278 | 1.2 | 0.91 ± 0.01 |  | 41 | 41 | 0 | 0 | yes |  |  |  | blast | uaggccggugggaaaagcaagu | augcuucacucccagcuaugca | augcuucacucccagcuaugcaaaucuuuuuguaggccggugggaaaagcaagu | manual\_scaffold\_4a:306320854..306320908:- |
| manual\_scaffold\_3b\_12257 | 1.2 | 0.91 ± 0.01 |  | 59 | 59 | 0 | 0 | yes |  |  |  | blast | cugugggcaguuuuuggaac | uucauuaagcugccccagcu | cugugggcaguuuuuggaacuagacaauuucauuaagcugccccagcu | manual\_scaffold\_3b:1236743096..1236743144:- |
| manual\_scaffold\_2a\_4845 | 1.2 | 0.91 ± 0.01 |  | 13 | 13 | 0 | 0 | yes |  |  |  | blast | ucugagcacacauccguugga | uagcggaagcaggguucagaua | uagcggaagcaggguucagauaaucaguuaaagucugagcacacauccguugga | manual\_scaffold\_2a:1402517356..1402517410:+ |
| manual\_scaffold\_11\_37028 | 1.2 | 0.91 ± 0.01 |  | 13 | 13 | 0 | 0 | yes |  |  |  | blast | aaaacggcucuguauagcuugu | aaguuuuacagagcugaugacc | aaguuuuacagagcugaugaccucaaaacaaaggggaaaacggcucuguauagcuugu | manual\_scaffold\_11:171010644..171010702:- |
| manual\_scaffold\_4b\_16967 | 1.2 | 0.91 ± 0.01 |  | 484 | 331 | 0 | 153 | yes |  |  |  | blast | aguuuggagcugccaagaggu | cacuuggccacucuucaacucu | cacuuggccacucuucaacucugugcuauaauggcuaguagaguuuggagcugccaagaggu | manual\_scaffold\_4b:620353779..620353841:- |
| manual\_scaffold\_10\_35125 | 1.2 | 0.91 ± 0.01 |  | 1016 | 1016 | 0 | 0 | yes |  |  |  | blast | gucccuguucgggcgcca | aagccucagcaaggccua | gucccuguucgggcgccacuuugucaaaguaagccucagcaaggccua | manual\_scaffold\_10:483598231..483598279:- |
| manual\_scaffold\_4a\_14519 | 1.2 | 0.91 ± 0.01 |  | 112 | 112 | 0 | 0 | yes |  |  |  | blast | ugcuaagcagacucugaggcugc | agccugugcuucgcuuggcaau | ugcuaagcagacucugaggcugcugcuaauuuggaaagcuugggcagccugugcuucgcuuggcaau | manual\_scaffold\_4a:608570758..608570825:- |
| manual\_scaffold\_3b\_11393 | 1.2 | 0.91 ± 0.01 |  | 14 | 14 | 0 | 0 | yes |  |  |  | blast | accacuggcagauucugc | aguguccgccacuguag | accacuggcagauucugccauuggaccaagccugcuacagacaguaauggucuccuauggcaguguccgccacuguag | manual\_scaffold\_3b:422413586..422413664:- |
| manual\_scaffold\_1a\_1004 | 1.2 | 0.91 ± 0.01 |  | 10 | 10 | 0 | 0 | yes |  |  |  | blast | uagggccuagcgguguucugcu | uagaccccugucagccauauu | uagaccccugucagccauauuaugaaaaauaauagggccuagcgguguucugcu | manual\_scaffold\_1a:149302026..149302080:- |
| manual\_scaffold\_8\_30154 | 1.2 | 0.91 ± 0.01 |  | 24 | 24 | 0 | 0 | yes |  |  |  | blast | aaguuucauuucugugaaggacu | uccugacacagaaugaaacauuu | uccugacacagaaugaaacauuucauagauuuuguaacggaaaguuucauuucugugaaggacu | manual\_scaffold\_8:572095650..572095714:- |
| manual\_scaffold\_6\_23788 | 1.2 | 0.91 ± 0.01 |  | 62 | 62 | 0 | 0 | yes |  |  |  | blast | caccucugagaaaauggcac | cucaugcugcagagggga | cucaugcugcagaggggagguugcacugagcauaaaauacuaaugcugccaccgccaccucugagaaaauggcac | manual\_scaffold\_6:846372702..846372777:- |
| manual\_scaffold\_4b\_17237 | 1.2 | 0.91 ± 0.01 |  | 27 | 27 | 0 | 0 | yes |  |  |  | blast | ugaaagaucuaauccuggacu | uccaguauugguaucuuucaua | ugaaagaucuaauccuggacuaagaaacugaguuacuccaguauugguaucuuucaua | manual\_scaffold\_4b:960777355..960777413:- |
| manual\_scaffold\_5\_20270 | 1.2 | 0.91 ± 0.01 |  | 20 | 20 | 0 | 0 | yes |  |  |  | blast | uugggaggcugaggcagu | uaccugcagaaacaggg | uaccugcagaaacaggggacagcaauggggacaucuuuugccccaacucuugcugggauuuugggaggcugaggcagu | manual\_scaffold\_5:1439248947..1439249025:- |
| manual\_scaffold\_2a\_3890 | 1.2 | 0.91 ± 0.01 |  | 1382 | 1335 | 0 | 47 | yes |  |  |  | blast | ccggaucccuuaccugcccu | agagcagguaugaggucugag | ccggaucccuuaccugcccuccgugauugucaaaagagagcagguaugaggucugag | manual\_scaffold\_2a:78499090..78499147:+ |
| manual\_scaffold\_2a\_3848 | 1.2 | 0.91 ± 0.01 |  | 11 | 11 | 0 | 0 | yes |  |  |  | blast | caagcuaacgcucucaggccu | accugagggcgcaagcuggu | caagcuaacgcucucaggccugcacugcuuaucuaaaccugagggcgcaagcuggu | manual\_scaffold\_2a:53382312..53382368:+ |
| manual\_scaffold\_4b\_16170 | 1.2 | 0.91 ± 0.01 |  | 18450 | 18450 | 0 | 0 | yes |  |  |  | blast | ucugagcacgcaucuguugga | ugguggaugcaagguucagaua | ugguggaugcaagguucagauaaucaguaaacucugagcacgcaucuguugga | manual\_scaffold\_4b:11925672..11925725:- |
| manual\_scaffold\_10\_35344 | 1.1 | 0.91 ± 0.01 |  | 19 | 19 | 0 | 0 | yes |  |  |  | blast | cuccgggugcccccucca | caggggacccaccuggccac | caggggacccaccuggccaccaggauacuuacuccgggugcccccucca | manual\_scaffold\_10:671254895..671254944:- |
| manual\_scaffold\_5\_18556 | 1.1 | 0.91 ± 0.01 |  | 17 | 17 | 0 | 0 | yes |  |  |  | blast | gugacgcuuggcugcuuugc | aaagcaaggcaaaucaugc | gugacgcuuggcugcuuugcaacauguaaagcaaggcaaaucaugc | manual\_scaffold\_5:1256135086..1256135132:+ |
| manual\_scaffold\_3b\_10986 | 1.1 | 0.91 ± 0.01 |  | 45 | 45 | 0 | 0 | yes |  |  |  | blast | augaggacuagcgugacggcc | cccucucccaaguccucaagg | augaggacuagcgugacggccauaaagaauacauugcccucucccaaguccucaagg | manual\_scaffold\_3b:1118968435..1118968492:+ |
| manual\_scaffold\_3a\_9732 | 1.1 | 0.91 ± 0.01 |  | 18450 | 18450 | 0 | 0 | yes |  |  |  | blast | ucugagcacgcaucuguugga | cauuagaugcaggguucagaua | cauuagaugcaggguucagauaaucaguaaacucugagcacgcaucuguugga | manual\_scaffold\_3a:668215492..668215545:- |
| manual\_scaffold\_11\_37060 | 1.1 | 0.91 ± 0.01 |  | 11 | 11 | 0 | 0 | yes |  |  |  | blast | ccauggagcuuuacugua | ggguguugccauggcu | ggguguugccauggcuuaaggguggguccacugaguaagccauggagcuuuacugua | manual\_scaffold\_11:233413501..233413558:- |
| manual\_scaffold\_5\_18839 | 1.1 | 0.91 ± 0.01 |  | 37 | 37 | 0 | 0 | yes |  |  |  | blast | uugcggucacauuaguucucacu | ugaggacuugugaaaggacaaug | ugaggacuugugaaaggacaauguacucuucauugcggucacauuaguucucacu | manual\_scaffold\_5:1608528339..1608528394:+ |
| manual\_scaffold\_5\_20162 | 1.1 | 0.91 ± 0.01 |  | 11 | 11 | 0 | 0 | yes |  |  |  | blast | cuuuccauuagggcuguguacu | uauacugcacuaauggaaauau | uauacugcacuaauggaaauauuguucaugaaaagucacuuuccauuagggcuguguacu | manual\_scaffold\_5:1288747576..1288747636:- |
| manual\_scaffold\_2a\_4725 | 1.1 | 0.91 ± 0.01 |  | 6690 | 6690 | 0 | 0 | yes |  |  |  | blast | uccgggguacucagcacgaagg | uucgugaguagcucggguc | uccgggguacucagcacgaaggauuaauauucgugaguagcucggguc | manual\_scaffold\_2a:1226347722..1226347770:+ |
| manual\_scaffold\_12\_39074 | 1.1 | 0.91 ± 0.01 |  | 11 | 11 | 0 | 0 | yes |  |  |  | blast | agauuuccagcuaauugucggu | ucacaauuagcugagagucccugc | ucacaauuagcugagagucccugcagagagcagauuuccagcuaauugucggu | manual\_scaffold\_12:92884061..92884114:- |
| manual\_scaffold\_10\_33819 | 1.1 | 0.91 ± 0.01 |  | 53 | 53 | 0 | 0 | yes |  |  |  | blast | aaucaacagaucucucacuuggu | uagguugagugaucaguugaguuuugu | uagguugagugaucaguugaguuuuguguuaaucuuuacaaucaacagaucucucacuuggu | manual\_scaffold\_10:327942420..327942482:+ |
| manual\_scaffold\_5\_19846 | 1.1 | 0.91 ± 0.01 |  | 9 | 9 | 0 | 0 | yes |  |  |  | blast | uaaaugcagcugugaaucuuua | aagauucacagcugcauuuacg | aagauucacagcugcauuuacggaaccccguaaaugcagcugugaaucuuua | manual\_scaffold\_5:867595329..867595381:- |
| manual\_scaffold\_4b\_14830 | 1.1 | 0.91 ± 0.01 |  | 71 | 71 | 0 | 0 | yes |  |  |  | blast | aaaguucugugaugcacugacu | ucuaaguguauaacagaacuucac | aaaguucugugaugcacugacuucaagcaaucacagucuaaguguauaacagaacuucac | manual\_scaffold\_4b:40732872..40732932:+ |
| manual\_scaffold\_5\_18615 | 1.1 | 0.91 ± 0.01 |  | 11 | 11 | 0 | 0 | yes |  |  |  | blast | uccagacgagcuauguuauau | guaacaaagcuggucugaaga | guaacaaagcuggucugaagauuugaaauuugaaccguuuccagacgagcuauguuauau | manual\_scaffold\_5:1331560981..1331561041:+ |
| manual\_scaffold\_9\_31525 | 1.1 | 0.91 ± 0.01 |  | 162 | 162 | 0 | 0 | yes |  |  |  | blast | uuccgagguagauaaaaugagu | ucaucuuuuuaucuacuaguuaaagg | ucaucuuuuuaucuacuaguuaaaggauuuaggcuuugcagccaugagcuccacucagccuaucauccuuccgagguagauaaaaugagu | manual\_scaffold\_9:737299273..737299363:+ |
| manual\_scaffold\_10\_33503 | 1.1 | 0.91 ± 0.01 |  | 10 | 10 | 0 | 0 | yes |  |  |  | blast | cccggccugaucaguaacuga | aguuacugaucaggccgggca | aguuacugaucaggccgggcaagcauacccggccugaucaguaacuga | manual\_scaffold\_10:68050956..68051004:+ |
| manual\_scaffold\_12\_39636 | 1.1 | 0.91 ± 0.01 |  | 18 | 18 | 0 | 0 | yes |  |  |  | blast | uuucaagucguagugaugcu | cgcaucacgacuugaaagc | cgcaucacgacuugaaagccgagacaagcuuucaagucguagugaugcu | manual\_scaffold\_12:597958757..597958806:- |
| manual\_scaffold\_10\_35074 | 1.1 | 0.91 ± 0.01 |  | 79 | 79 | 0 | 0 | yes |  |  |  | blast | gcgaggauccuggaugcuguuu | acagcgucuggcucuugcug | acagcgucuggcucuugcuguuaugcagacacacacacaugucuucacaccagcgaggauccuggaugcuguuu | manual\_scaffold\_10:404234736..404234810:- |
| manual\_scaffold\_1a\_1805 | 1.1 | 0.91 ± 0.01 |  | 11 | 11 | 0 | 0 | yes |  |  |  | blast | aauuuggcccuuuagauuuacu | uaacucuggaaggccagaguua | aauuuggcccuuuagauuuacugaugaagugaguaaugguaacucuggaaggccagaguua | manual\_scaffold\_1a:1152357210..1152357271:- |
| manual\_scaffold\_3b\_11139 | 1.1 | 0.91 ± 0.01 |  | 20 | 19 | 1 | 0 | yes |  |  |  | blast | uaguaccagugguuguagacac | gucuggaucaacugaugcuaca | uaguaccagugguuguagacacagaaagcacuguucaggacaaggcucuggagugucuggaucaacugaugcuaca | manual\_scaffold\_3b:78247263..78247339:- |
| manual\_scaffold\_7\_27214 | 1.1 | 0.91 ± 0.01 |  | 124 | 115 | 0 | 9 | yes |  |  |  | blast | ugaaccagucuacgucagguauc | gauacccgcaaacuguucaca | gauacccgcaaacuguucacaagacauuuucugugugaaccagucuacgucagguauc | manual\_scaffold\_7:675127611..675127669:- |
| manual\_scaffold\_1b\_2113 | 1.1 | 0.91 ± 0.01 |  | 15 | 15 | 0 | 0 | yes |  |  |  | blast | aaccuguggaaugcaucaaaau | uuuggugcauccccagacuua | uuuggugcauccccagacuuaccaagauugauaaaccuguggaaugcaucaaaau | manual\_scaffold\_1b:362833084..362833139:+ |
| manual\_scaffold\_3b\_10707 | 1.1 | 0.91 ± 0.01 |  | 135 | 134 | 0 | 1 | yes |  |  |  | blast | guuccccugguagaacaccaa | cuggugcacuccuuggugggua | cuggugcacuccuugguggguaguauaaucuguuccccugguagaacaccaa | manual\_scaffold\_3b:810002521..810002573:+ |
| manual\_scaffold\_12\_38933 | 1.1 | 0.91 ± 0.01 |  | 11 | 11 | 0 | 0 | yes |  |  |  | blast | cauggagugggagaaacucug | gaguucacugcccuccuaauugcc | cauggagugggagaaacucugugaacagaguucacugcccuccuaauugcc | manual\_scaffold\_12:22256309..22256360:- |
| manual\_scaffold\_5\_18283 | 1.1 | 0.91 ± 0.01 |  | 9 | 9 | 0 | 0 | yes |  |  |  | blast | uaaaugcagcugugaaucuuua | aagauucacagcugcauuuacg | aagauucacagcugcauuuacgggguuccguaaaugcagcugugaaucuuua | manual\_scaffold\_5:867595331..867595383:+ |
| manual\_scaffold\_4b\_16894 | 1.1 | 0.91 ± 0.01 |  | 15 | 15 | 0 | 0 | yes |  |  |  | blast | uuggaucugucuacacuuuuga | aggaguuuagauaagaucuagag | aggaguuuagauaagaucuagagguaaaaaagaucacuuuucuuggaucugucuacacuuuuga | manual\_scaffold\_4b:507580360..507580424:- |
| manual\_scaffold\_1a\_1114 | 1.1 | 0.91 ± 0.01 |  | 25 | 25 | 0 | 0 | yes |  |  |  | blast | cacaauuuggucaucgcuauug | guagcgauuacuuaauugcaau | guagcgauuacuuaauugcaauucacaaaaauucacaauuuggucaucgcuauug | manual\_scaffold\_1a:297095353..297095408:- |
| manual\_scaffold\_6\_21031 | 1.1 | 0.91 ± 0.01 |  | 771 | 771 | 0 | 0 | yes |  |  |  | blast | ccucugggacuccuucug | gggaguuccuguuggagguu | gggaguuccuguuggagguuuuuuacccuauugggguuuuuccucugggacuccuucug | manual\_scaffold\_6:266539861..266539920:+ |
| manual\_scaffold\_7\_24672 | 1.1 | 0.91 ± 0.01 |  | 53 | 53 | 0 | 0 | yes |  |  |  | blast | uguggucugccauugaugauc | acaucagugauagagcagagu | uguggucugccauugaugaucagugcuucccaucugacaucagugauagagcagagu | manual\_scaffold\_7:48677255..48677312:+ |
| manual\_scaffold\_1a\_1054 | 1.1 | 0.91 ± 0.01 |  | 46 | 45 | 0 | 1 | yes |  |  |  | blast | aggccuguuuuccugaccucu | aagggccagguucaacagu | aagggccagguucaacagucccuuguacaauauacaaaaggccuguuuuccugaccucu | manual\_scaffold\_1a:199511099..199511158:- |
| manual\_scaffold\_6\_21994 | 1.1 | 0.91 ± 0.01 |  | 105 | 105 | 0 | 0 | yes |  |  |  | blast | ugugggcaguuuuuggaacc | uuucacuaacugcccuagc | ugugggcaguuuuuggaaccagacauuuguuaauuguuucacuaacugcccuagc | manual\_scaffold\_6:1210805600..1210805655:+ |
| manual\_scaffold\_7\_27338 | 1.1 | 0.91 ± 0.01 |  | 191 | 191 | 0 | 0 | yes |  |  |  | blast | gauagaucgcgaauugugacu | ucacaauccgaccuaccuc | ucacaauccgaccuaccucaugaauauugaagagauagaucgcgaauugugacu | manual\_scaffold\_7:828340581..828340635:- |
| manual\_scaffold\_6\_23524 | 1.1 | 0.91 ± 0.01 |  | 10 | 8 | 0 | 2 | no |  |  |  | blast | uuggucgggcguugugcuggga | cuaggacagcaccuuauuugagg | cuaggacagcaccuuauuugagguuaucauugcuuggucgggcguugugcuggga | manual\_scaffold\_6:516310656..516310711:- |
| manual\_scaffold\_10\_35798 | 1.1 | 0.91 ± 0.01 |  | 33 | 33 | 0 | 0 | yes |  |  |  | blast | ugaugaaaucagacagacaccuc | uguguguguuuuuuuuuaucugg | uguguguguuuuuuuuuaucugguuuuaaguaaaauauccugaugaaaucagacagacaccuc | manual\_scaffold\_10:1053313876..1053313939:- |
| manual\_scaffold\_10\_33838 | 1.1 | 0.91 ± 0.01 |  | 11 | 11 | 0 | 0 | yes |  |  |  | blast | cuugcagugcucuuauggugu | aucaugagagcaaugcuucca | cuugcagugcucuuaugguguuaauugauacuaacaucaugagagcaaugcuucca | manual\_scaffold\_10:349096466..349096522:+ |
| manual\_scaffold\_3a\_9661 | 1.1 | 0.91 ± 0.01 |  | 36 | 36 | 0 | 0 | yes |  |  |  | blast | uacuggaaaaugugugcacuca | agugcagcacaucagcugguacu | uacuggaaaaugugugcacucaaguuucuauauuuagugcagcacaucagcugguacu | manual\_scaffold\_3a:594707192..594707250:- |
| manual\_scaffold\_6\_24532 | 1.1 | 0.91 ± 0.01 |  | 48 | 48 | 0 | 0 | yes |  |  |  | blast | cuugucuguuggaguaaaucu | auuuucuccuacggacaaucc | auuuucuccuacggacaauccuucaauuuuguggcuugucuguuggaguaaaucu | manual\_scaffold\_6:1721699828..1721699883:- |
| manual\_scaffold\_3a\_7983 | 1.1 | 0.91 ± 0.01 |  | 11 | 11 | 0 | 0 | yes |  |  |  | blast | uuggcaccggaaccccguaau | cucgggguucgggugucauga | cucgggguucgggugucaugauagcaacagacuuggcaccggaaccccguaau | manual\_scaffold\_3a:529044310..529044363:+ |
| manual\_scaffold\_10\_33666 | 1.1 | 0.91 ± 0.01 |  | 15 | 15 | 0 | 0 | yes |  |  |  | blast | cuagggggcacaaaaaaa | uuuuucaaauaugccccauaggg | cuagggggcacaaaaaaaguggcacagaccugugugcagcgccacauuuuuucaaauaugccccauaggg | manual\_scaffold\_10:173321790..173321860:+ |
| manual\_scaffold\_9\_32274 | 1.1 | 0.91 ± 0.01 |  | 142 | 142 | 0 | 0 | yes |  |  |  | blast | aauaucacaccugguaauuacug | gugucuauggugacaugguaugcu | gugucuauggugacaugguaugcugggcaccuuagugaacagaauaucacaccugguaauuacug | manual\_scaffold\_9:73399400..73399465:- |
| manual\_scaffold\_1a\_443 | 1.1 | 0.91 ± 0.01 |  | 771 | 771 | 0 | 0 | yes |  |  |  | blast | ccucugggacuccuucug | gggaguuccuguggagguu | gggaguuccuguggagguuuuuuuacccuacugggguuuuuccucugggacuccuucug | manual\_scaffold\_1a:528810959..528811018:+ |
| manual\_scaffold\_6\_22193 | 1.1 | 0.91 ± 0.01 |  | 32 | 32 | 0 | 0 | yes |  |  |  | blast | uuuaguucucuaguguugguauc | uccaauacuggagaacaagu | uccaauacuggagaacaagugagguaccuguaacuuuaguucucuaguguugguauc | manual\_scaffold\_6:1376646454..1376646511:+ |
| manual\_scaffold\_6\_20861 | 1.1 | 0.91 ± 0.01 |  | 13 | 13 | 0 | 0 | yes |  |  |  | blast | ugagaccgucacagcugacagc | cgccagcugucacggucuucga | cgccagcugucacggucuucgaucgaggacugauaaucgugaaacucugagaccgucacagcugacagc | manual\_scaffold\_6:161564143..161564212:+ |
| manual\_scaffold\_8\_30446 | 1.1 | 0.91 ± 0.01 |  | 47 | 47 | 0 | 0 | yes |  |  |  | blast | ucggggacugagaacuuuaga | uaaaaguuuuuuagucuccggau | uaaaaguuuuuuagucuccggauaagaauauagucggggacugagaacuuuaga | manual\_scaffold\_8:1031054372..1031054426:- |
| manual\_scaffold\_3b\_12186 | 1.1 | 0.91 ± 0.01 |  | 10 | 10 | 0 | 0 | yes |  |  |  | blast | uacauuaacuguggcuagcauu | agcuagccacaguuaauguaga | agcuagccacaguuaauguagagaguugggcucucuacauuaacuguggcuagcauu | manual\_scaffold\_3b:1153716354..1153716411:- |
| manual\_scaffold\_9\_31281 | 1.1 | 0.91 ± 0.01 |  | 18 | 18 | 0 | 0 | yes |  |  |  | blast | uuuccuguuggaugaauuguggcu | ucccuuucagccaacaaggaggu | uuuccuguuggaugaauuguggcuuugaacuuguuggucccuuucagccaacaaggaggu | manual\_scaffold\_9:414483892..414483952:+ |
| manual\_scaffold\_1a\_1388 | 1.1 | 0.91 ± 0.01 |  | 47 | 47 | 0 | 0 | yes |  |  |  | blast | uucugggaggcguagucucacu | ggcagacugccucuuucaguugc | ggcagacugccucuuucaguugcuauaggucagcuucugggaggcguagucucacu | manual\_scaffold\_1a:670033544..670033600:- |
| manual\_scaffold\_11\_37109 | 1.1 | 0.91 ± 0.01 |  | 64 | 64 | 0 | 0 | yes |  |  |  | blast | ucuggaaguugucuggaaucu | guucagcuccuucuugaaa | ucuggaaguugucuggaaucuaggaguacugucccuggguucagcuccuucuugaaa | manual\_scaffold\_11:286861436..286861493:- |
| manual\_scaffold\_7\_24710 | 1.1 | 0.91 ± 0.01 |  | 65 | 65 | 0 | 0 | yes |  |  |  | blast | cuaaaaaggaacucggca | cuuuguuccucuguauggu | cuaaaaaggaacucggcauucagcuuuguuccucuguauggu | manual\_scaffold\_7:80494764..80494806:+ |
| manual\_scaffold\_8\_30276 | 1.1 | 0.91 ± 0.01 |  | 24 | 18 | 0 | 6 | yes |  |  |  | blast | ugccugagucagaccagaacag | uuuggucugugccagacacacc | uuuggucugugccagacacaccuuucagaaugugugccugagucagaccagaacag | manual\_scaffold\_8:783480782..783480838:- |
| manual\_scaffold\_8\_29021 | 1.1 | 0.91 ± 0.01 |  | 19 | 19 | 0 | 0 | yes |  |  |  | blast | uugcuauccugucaguucucacu | uaggaacuuccaggaugcagug | uaggaacuuccaggaugcaguguguccuucauugcuauccugucaguucucacu | manual\_scaffold\_8:641240896..641240950:+ |
| manual\_scaffold\_3a\_7400 | 1.0 | 0.91 ± 0.01 |  | 690 | 454 | 236 | 0 | yes |  |  |  | blast | ccuaauccccucuucccc | ggacguguugggaa | ccuaauccccucuuccccucucgaacacucuuagccucugugauuuaucuaucagagucuuggagcuguucggugguggacguguugggaa | manual\_scaffold\_3a:180851358..180851449:+ |
| manual\_scaffold\_3b\_10229 | 1.0 | 0.91 ± 0.01 |  | 675 | 675 | 0 | 0 | yes |  |  |  | blast | aacgugacugcaagcccagaa | cugggccuuguggucuuguuuu | cugggccuuguggucuuguuuuauaauggaaaaaacgugacugcaagcccagaa | manual\_scaffold\_3b:263137781..263137835:+ |
| manual\_scaffold\_10\_35403 | 1.0 | 0.91 ± 0.01 |  | 11 | 11 | 0 | 0 | yes |  |  |  | blast | acuuggcucugugcacacuaucu | auaguauauacaaagccagcuuc | acuuggcucugugcacacuaucucucaguuugagauaguauauacaaagccagcuuc | manual\_scaffold\_10:749391946..749392003:- |
| manual\_scaffold\_9\_31431 | 1.0 | 0.91 ± 0.01 |  | 26 | 26 | 0 | 0 | yes |  |  |  | blast | uucucucugaucauuuuccaca | uggagaaguucagagaaaguaaggaaa | uggagaaguucagagaaaguaaggaaaguuggaaaacuauucucucugaucauuuuccaca | manual\_scaffold\_9:586752885..586752946:+ |
| manual\_scaffold\_12\_39351 | 1.0 | 0.91 ± 0.01 |  | 18 | 18 | 0 | 0 | yes |  |  |  | blast | guucuucugaaaaucuuggcauu | ugccaaguauuuugaagaauca | guucuucugaaaaucuuggcauuguauguguuaaugccaaguauuuugaagaauca | manual\_scaffold\_12:449196397..449196453:- |
| manual\_scaffold\_2b\_6611 | 1.0 | 0.91 ± 0.01 |  | 532 | 532 | 0 | 0 | yes |  |  |  | blast | uguagcaauucuguuuggauu | uucaacaguauuguuaucgc | uguagcaauucuguuuggauuuuagauuacauuauucaaaauucaacaguauuguuaucgc | manual\_scaffold\_2b:98736342..98736403:- |
| manual\_scaffold\_6\_22409 | 1.0 | 0.91 ± 0.01 |  | 23 | 23 | 0 | 0 | yes |  |  |  | blast | uuugaacagggcuccauuguga | acugugggacuuuugugcaauug | acugugggacuuuugugcaauugugucaguguucuauacauuugaacagggcuccauuguga | manual\_scaffold\_6:1609888692..1609888754:+ |
| manual\_scaffold\_1a\_1170 | 1.0 | 0.91 ± 0.01 |  | 19 | 19 | 0 | 0 | yes |  |  |  | blast | aacuaggaaaaaaauacucucc | agguauugguuuuccucguuug | agguauugguuuuccucguuugccaacuuagagcacaaacuaggaaaaaaauacucucc | manual\_scaffold\_1a:354395390..354395449:- |
| manual\_scaffold\_2a\_4082 | 1.0 | 0.91 ± 0.01 |  | 34 | 34 | 0 | 0 | yes |  |  |  | blast | cauccggccacuccaaacuucu | aguuggugcugccagaaggu | cauccggccacuccaaacuucugacaacuaauauagucacagaguuggugcugccagaaggu | manual\_scaffold\_2a:322996397..322996459:+ |
| manual\_scaffold\_2b\_6127 | 1.0 | 0.91 ± 0.01 |  | 176471 | 174990 | 0 | 1481 | yes |  |  |  | blast | uaaggcacgcggugaaugcc | cguguucacagcggaccuugau | cguguucacagcggaccuugauuuaaugucauacaauuaaggcacgcggugaaugcc | manual\_scaffold\_2b:287000379..287000436:+ |
| manual\_scaffold\_4b\_17134 | 1.0 | 0.91 ± 0.01 |  | 17 | 17 | 0 | 0 | yes |  |  |  | blast | aaacaggacuucaguuccc | ggcuuguaguuguguuugu | ggcuuguaguuguguuuguucuaauauaaaacaggacuucaguuccc | manual\_scaffold\_4b:851098426..851098473:- |
| manual\_scaffold\_4b\_15164 | 1.0 | 0.91 ± 0.01 |  | 11 | 11 | 0 | 0 | yes |  |  |  | blast | gaauuuuuggaugaucuc | gugcccaaaacucca | gaauuuuuggaugaucuccgagcccggagugcccaaaacucca | manual\_scaffold\_4b:282920391..282920434:+ |
| manual\_scaffold\_9\_31178 | 1.0 | 0.91 ± 0.01 |  | 17 | 17 | 0 | 0 | yes |  |  |  | blast | aucuuguagucuuggggc | gucaggugcugaggaugg | aucuuguagucuuggggcauugcgguugcaaugucaggugcugaggaugg | manual\_scaffold\_9:310649095..310649145:+ |
| manual\_scaffold\_4b\_15554 | 1.0 | 0.91 ± 0.01 |  | 21 | 21 | 0 | 0 | yes |  |  |  | blast | ugugugucuuucucugugagccu | gccguagcgcuugacacauauu | ugugugucuuucucugugagccuucccacacagggccguagcgcuugacacauauu | manual\_scaffold\_4b:685431528..685431584:+ |
| manual\_scaffold\_11\_36964 | 1.0 | 0.91 ± 0.01 |  | 14 | 14 | 0 | 0 | yes |  |  |  | blast | accgguauccuugcacuuaaau | uuaggugcccggauaauauguu | uuaggugcccggauaauauguugcuagaaauaacaauucugaccgguauccuugcacuuaaau | manual\_scaffold\_11:101301284..101301347:- |
| manual\_scaffold\_6\_22040 | 1.0 | 0.91 ± 0.01 |  | 30 | 30 | 0 | 0 | yes |  |  |  | blast | aauggaugccucuguaggauaca | uauccuccacuggcggccaguuu | aauggaugccucuguaggauacacacaacauuauuugacuguauccuccacuggcggccaguuu | manual\_scaffold\_6:1239629726..1239629790:+ |
| manual\_scaffold\_9\_33088 | 1.0 | 0.91 ± 0.01 |  | 156 | 156 | 0 | 0 | yes |  |  |  | blast | uuggcacuguguucucuuccc | gaugagagcauugcagagccauau | gaugagagcauugcagagccauaucuacuaacauuuggcacuguguucucuuccc | manual\_scaffold\_9:1036358808..1036358863:- |
| manual\_scaffold\_3b\_12046 | 1.0 | 0.91 ± 0.01 |  | 75 | 75 | 0 | 0 | yes |  |  |  | blast | ugacagggcauuuaaugaugagc | uaguuauuaaaugcccucuucuu | uaguuauuaaaugcccucuucuuuguuauuggagccacagaugacagggcauuuaaugaugagc | manual\_scaffold\_3b:1008801614..1008801678:- |
| manual\_scaffold\_7\_26073 | 1.0 | 0.91 ± 0.01 |  | 37 | 33 | 0 | 4 | yes |  |  |  | blast | uauggcgauauuguggaguau | auucuccacaacgucguaau | auucuccacaacgucguaaucauacgcaacaccuccucguauggcgauauuguggaguau | manual\_scaffold\_7:1343907634..1343907694:+ |
| manual\_scaffold\_1a\_296 | 1.0 | 0.91 ± 0.01 |  | 30 | 30 | 0 | 0 | yes |  |  |  | blast | aaagaccucucucggugggcu | ucuaccauagugaggccucaua | aaagaccucucucggugggcuguguucucucucacuucuaccauagugaggccucaua | manual\_scaffold\_1a:358139213..358139271:+ |
| manual\_scaffold\_9\_33006 | 1.0 | 0.91 ± 0.01 |  | 19 | 19 | 0 | 0 | yes |  |  |  | blast | cacaucuguuuuguuuagggu | ucuaaauagggcagauggagua | ucuaaauagggcagauggaguaauguauuuccgucacacaucuguuuuguuuagggu | manual\_scaffold\_9:938765543..938765600:- |
| manual\_scaffold\_9\_32660 | 1.0 | 0.91 ± 0.01 |  | 31 | 31 | 0 | 0 | yes |  |  |  | blast | agccaaagugacaugcgaacu | ugcacaucacuuuggcuug | ugcacaucacuuuggcuugcacaaaauacccagccaaagugacaugcgaacu | manual\_scaffold\_9:549078290..549078342:- |
| manual\_scaffold\_7\_25728 | 1.0 | 0.91 ± 0.01 |  | 11 | 10 | 0 | 1 | no |  |  |  | blast | ucugggucuuguaguugcugucu | gaggaacaaaagucccagucu | ucugggucuuguaguugcugucuuuccauagugcaagaggaacaaaagucccagucu | manual\_scaffold\_7:1085252059..1085252116:+ |
| manual\_scaffold\_11\_36380 | 1.0 | 0.91 ± 0.01 |  | 13 | 13 | 0 | 0 | yes |  |  |  | blast | caccuggcaacuccaaaacuca | aguugguggugccuggag | caccuggcaacuccaaaacucagugcuauaauagccuucugaguugguggugccuggag | manual\_scaffold\_11:698308022..698308081:+ |
| manual\_scaffold\_1a\_1812 | 1.0 | 0.91 ± 0.01 |  | 56 | 55 | 0 | 1 | yes |  |  |  | blast | uuaaggaugguagauaaggugc | gagccuuauccuacaucccaagu | gagccuuauccuacaucccaaguuaaucuacacuuaaggaugguagauaaggugc | manual\_scaffold\_1a:1159354233..1159354288:- |
| manual\_scaffold\_12\_39136 | 1.0 | 0.91 ± 0.01 |  | 39 | 39 | 0 | 0 | yes |  |  |  | blast | auggcacucguaaaaugguuga | ugccauguuacaagugacauau | ugccauguuacaagugacauaucaagugacauaucuuauggcacucguaaaaugguuga | manual\_scaffold\_12:158167938..158167997:- |
| manual\_scaffold\_7\_25069 | 1.0 | 0.91 ± 0.01 |  | 62 | 62 | 0 | 0 | yes |  |  |  | blast | uaccauuucucauuggacu | uccauugauaucugguggu | uccauugauaucuggugguguuaucucuaguguguuaccauuucucauuggacu | manual\_scaffold\_7:407821006..407821060:+ |
| manual\_scaffold\_3b\_10222 | 1.0 | 0.91 ± 0.01 |  | 22 | 22 | 0 | 0 | yes |  |  |  | blast | cacguggccauucuuuaacucu | aguuuggagcugccacgaggu | cacguggccauucuuuaacucuaugcuauaacggcuaguagaguuuggagcugccacgaggu | manual\_scaffold\_3b:251814765..251814827:+ |
| manual\_scaffold\_1b\_2139 | 1.0 | 0.91 ± 0.01 |  | 9 | 9 | 0 | 0 | yes |  |  |  | blast | ugccuuggacuuguaaccugca | cagguuacaaguucaaugugcc | cagguuacaaguucaaugugcccagacgguuugaagugccuuggacuuguaaccugca | manual\_scaffold\_1b:394072358..394072416:+ |
| manual\_scaffold\_9\_30937 | 1.0 | 0.91 ± 0.01 |  | 29 | 29 | 0 | 0 | yes |  |  |  | blast | acgagaguaccgucgacugcac | gcaacgucaacaccaucgucg | gcaacgucaacaccaucgucgaugagaacaucguuguugaugacaucgucgacgagaguaccgucgacugcac | manual\_scaffold\_9:96471378..96471451:+ |
| manual\_scaffold\_4a\_13479 | 1.0 | 0.91 ± 0.01 |  | 14 | 14 | 0 | 0 | yes |  |  |  | blast | uccaaccagacaugugugcuuu | ugcccauaucuggugggaca | ugcccauaucuggugggacaggucuguuauuauguccaaccagacaugugugcuuu | manual\_scaffold\_4a:794833015..794833071:+ |
| manual\_scaffold\_2b\_6043 | 1.0 | 0.91 ± 0.01 |  | 96473 | 96473 | 0 | 0 | yes |  |  |  | blast | agaagacgaucaaacuugac | uuaguaagauccuuggau | uuaguaagauccuuggaucgccccugccagggucagcaauagcccugguggagcacauagaagacgaucaaacuugac | manual\_scaffold\_2b:167602182..167602260:+ |
| manual\_scaffold\_5\_19019 | 1.0 | 0.91 ± 0.01 |  | 100 | 100 | 0 | 0 | yes |  |  |  | blast | acauuggcuuauggauucucu | ggaaucuucuggcaacugcuc | ggaaucuucuggcaacugcucuaucccuguuacagaagaacauuggcuuauggauucucu | manual\_scaffold\_5:1822203004..1822203064:+ |
| manual\_scaffold\_3b\_12082 | 1.0 | 0.91 ± 0.01 |  | 13 | 13 | 0 | 0 | yes |  |  |  | blast | cgguagagaaaaaggcauc | ugccucuucucauuuuu | ugccucuucucauuuuucccagcuccucgaguuuaacugcagcgggcacugggaggacgguagagaaaaaggcauc | manual\_scaffold\_3b:1052424652..1052424728:- |
| manual\_scaffold\_7\_27262 | 1.0 | 0.91 ± 0.01 |  | 17 | 17 | 0 | 0 | yes |  |  |  | blast | gucugagcacgcgucuguugga | ccuggcggauacaguguucagauaa | ccuggcggauacaguguucagauaaucaguaaagucugagcacgcgucuguugga | manual\_scaffold\_7:741888534..741888589:- |
| manual\_scaffold\_7\_26075 | 1.0 | 0.91 ± 0.01 |  | 37 | 33 | 0 | 4 | yes |  |  |  | blast | uauggcgauauuguggaguau | auucuccacaacgucguaau | auucuccacaacgucguaaucauacgcaacaccuccucguauggcgauauuguggaguau | manual\_scaffold\_7:1344943781..1344943841:+ |
| manual\_scaffold\_10\_33643 | 1.0 | 0.91 ± 0.01 |  | 12 | 12 | 0 | 0 | yes |  |  |  | blast | cuugccaaucgacucgcacgcu | cgcgcgagccgaccggcauuuc | cgcgcgagccgaccggcauuucuccuucuauuucaaggacuugccaaucgacucgcacgcu | manual\_scaffold\_10:149148334..149148395:+ |
| manual\_scaffold\_8\_30839 | 1.0 | 0.91 ± 0.01 |  | 6531 | 6531 | 0 | 0 | yes |  |  |  | blast | uccgucuucucugaguugaguu | cucauuucgguguggacauuuc | uccgucuucucugaguugaguuugaggcaacugguuuccauccaagcaacgacugcgcucauuucgguguggacauuuc | manual\_scaffold\_8:1577929392..1577929471:- |
| manual\_scaffold\_12\_39126 | 1.0 | 0.91 ± 0.01 |  | 17 | 17 | 0 | 0 | yes |  |  |  | blast | uccuauuuuggcaaagugcuu | gcgcuuuccuagaauagcugg | gcgcuuuccuagaauagcugguguauuucagaccuccuauuuuggcaaagugcuu | manual\_scaffold\_12:132906846..132906901:- |
| manual\_scaffold\_9\_32029 | 1.0 | 0.91 ± 0.01 |  | 11328 | 11328 | 0 | 0 | yes |  |  |  | blast | ucugagcacacaucuguugga | caagcagaugcaggguucagaua | caagcagaugcaggguucagauaaucaguuaaauucugagcacacaucuguugga | manual\_scaffold\_9:1088155133..1088155188:+ |
| manual\_scaffold\_2b\_6084 | 0.9 | 0.75 ± 0.01 |  | 19 | 11 | 3 | 5 | yes |  |  |  | blast | gagucuuagaguuguucagugau | ccugaggaucuggugacucu | gagucuuagaguuguucagugauggacguguugggaacgugcacagacccugaggaucuggugacucu | manual\_scaffold\_2b:218026145..218026213:+ |
| manual\_scaffold\_12\_38907 | 0.9 | 0.75 ± 0.01 |  | 21 | 17 | 0 | 4 | yes |  |  |  | blast | aagauuuaugacgcuaagcaag | cguggcuucauaaaucucacu | aagauuuaugacgcuaagcaaggugggcuugcguggcuucauaaaucucacu | manual\_scaffold\_12:18582550..18582602:- |
| manual\_scaffold\_10\_34596 | 0.9 | 0.75 ± 0.01 |  | 12 | 12 | 0 | 0 | yes |  |  |  | blast | augguucuuuccugcucucu | agagcagaaauacaccauag | agagcagaaauacaccauaggauaguaacuaugguucuuuccugcucucu | manual\_scaffold\_10:46327959..46328009:- |
| manual\_scaffold\_2a\_5330 | 0.9 | 0.75 ± 0.01 |  | 11 | 11 | 0 | 0 | yes |  |  |  | blast | aaaccuggacuggggcauc | ugcuccaguccggguuuuu | ugcuccaguccggguuuuuauuuauuuaaguacgaagcaugucuacaugucccagaaaucaaaguggaaaaccuggacuggggcauc | manual\_scaffold\_2a:557023209..557023296:- |
| manual\_scaffold\_9\_30900 | 0.9 | 0.75 ± 0.01 |  | 76 | 76 | 0 | 0 | yes |  |  |  | blast | auuuggaaaaacgcuguaacaag | uguuggugugcuuuucaaaaga | auuuggaaaaacgcuguaacaagggaauaaauauacuuguuggugugcuuuucaaaaga | manual\_scaffold\_9:47982247..47982306:+ |
| manual\_scaffold\_4a\_13234 | 0.9 | 0.75 ± 0.01 |  | 45 | 45 | 0 | 0 | yes |  |  |  | blast | ucuggaacuuguaaucuuguuu | aaacggacuacaaguuccacaau | ucuggaacuuguaaucuuguuuauugaauuauaaaaacggacuacaaguuccacaau | manual\_scaffold\_4a:659128037..659128094:+ |
| manual\_scaffold\_5\_18905 | 0.9 | 0.75 ± 0.01 |  | 63223 | 63223 | 0 | 0 | yes |  |  |  | blast | auccucugagccaagcuuu | uucuggucagaguuuca | uucuggucagaguuucauuaguaguggaguagcuauguugcugugaccuacuaaaaaucauccucugagccaagcuuu | manual\_scaffold\_5:1683572098..1683572176:+ |
| manual\_scaffold\_3a\_8656 | 0.9 | 0.75 ± 0.01 |  | 94 | 94 | 0 | 0 | yes |  |  |  | blast | uuucauaccuauauaugagacu | ucucauaguauaugugugccuuu | uuucauaccuauauaugagacuguuuguugguauaaaaaccacagucucauaguauaugugugccuuu | manual\_scaffold\_3a:936516187..936516255:+ |
| manual\_scaffold\_9\_31591 | 0.9 | 0.75 ± 0.01 |  | 24 | 24 | 0 | 0 | yes |  |  |  | blast | uugaacuguuaagaaccacu | ugguccuaaacauuucacaa | ugguccuaaacauuucacaauuucgauagagaacuguugaacuguuaagaaccacu | manual\_scaffold\_9:801739558..801739614:+ |
| manual\_scaffold\_4a\_14430 | 0.9 | 0.75 ± 0.01 |  | 13 | 13 | 0 | 0 | yes |  |  |  | blast | ucagcucugaucaugcaacu | cugaaugaucagagccauug | cugaaugaucagagccauugucgugauuccauuuuguucccccaaguggucccaggcaucagcucugaucaugcaacu | manual\_scaffold\_4a:483444213..483444291:- |
| manual\_scaffold\_10\_34817 | 0.9 | 0.75 ± 0.01 |  | 20 | 20 | 0 | 0 | yes |  |  |  | blast | uuucagcuguaaaaacuacuaagg | uuaguuuuuuuacaguuaaacu | uuucagcuguaaaaacuacuaagggagcagcaaaauggccauuuaguuuuuuuacaguuaaacu | manual\_scaffold\_10:180602549..180602613:- |
| manual\_scaffold\_10\_33555 | 0.9 | 0.75 ± 0.01 |  | 27 | 27 | 0 | 0 | yes |  |  |  | blast | cugcccagugcucugaaa | caagacaacugugguuagc | caagacaacugugguuagcaucuuaaaugcugcaugccugcuuggggugcagcacuucagacaccugcccagugcucugaaa | manual\_scaffold\_10:82685988..82686070:+ |
| manual\_scaffold\_4b\_16173 | 0.9 | 0.75 ± 0.01 |  | 10 | 10 | 0 | 0 | yes |  |  |  | blast | agcggcacucauguuauuaga | uaauaacaugggugcccgcaca | agcggcacucauguuauuagaagcauaguggcuucuaauaacaugggugcccgcaca | manual\_scaffold\_4b:12731487..12731544:- |
| manual\_scaffold\_9\_30906 | 0.9 | 0.75 ± 0.01 |  | 11 | 11 | 0 | 0 | yes |  |  |  | blast | agucacaauuuugcaaccuaccu | guagguuggauugagacuaa | agucacaauuuugcaaccuaccuauugaauaauaauuagguagguuggauugagacuaa | manual\_scaffold\_9:51168220..51168279:+ |
| manual\_scaffold\_3b\_10028 | 0.9 | 0.75 ± 0.01 |  | 13 | 13 | 0 | 0 | yes |  |  |  | blast | cccuuccuguggaucgca | cgagccaagagaaggaagggau | cccuuccuguggaucgcagacacaccccgagccaagagaaggaagggau | manual\_scaffold\_3b:73842964..73843013:+ |
| manual\_scaffold\_6\_23613 | 0.9 | 0.75 ± 0.01 |  | 12 | 12 | 0 | 0 | yes |  |  |  | blast | ccugaaugauguaucugauc | ucuggucucugauaggga | ccugaaugauguaucugaucucuuacagggaagcuugcacugcugcucccugaaugguguaucuggucucugauaggga | manual\_scaffold\_6:641469294..641469373:- |
| manual\_scaffold\_3b\_11378 | 0.9 | 0.75 ± 0.01 |  | 17 | 17 | 0 | 0 | yes |  |  |  | blast | gaugagaacucaggagauuuuu | auuuccucugaacucucaacua | auuuccucugaacucucaacuauuaucucacuagggauugaugagaacucaggagauuuuu | manual\_scaffold\_3b:380295997..380296058:- |
| manual\_scaffold\_11\_37548 | 0.9 | 0.75 ± 0.01 |  | 691 | 607 | 0 | 84 | yes |  |  |  | blast | ccagacauauuggcucugcca | aaaagcaauaggucuagcaugc | aaaagcaauaggucuagcaugcauuuugaguuuugugccagacauauuggcucugcca | manual\_scaffold\_11:882799508..882799566:- |
| manual\_scaffold\_6\_21064 | 0.9 | 0.75 ± 0.01 |  | 16 | 16 | 0 | 0 | yes |  |  |  | blast | aagacaaaggugagucugaggcu | cccagccccgccuuucuccacu | aagacaaaggugagucugaggcugcuuucagacaagcccagccccgccuuucuccacu | manual\_scaffold\_6:297602371..297602429:+ |
| manual\_scaffold\_12\_39107 | 0.9 | 0.75 ± 0.01 |  | 77 | 31 | 0 | 46 | yes |  |  |  | blast | caccuggcuucauuauaacucu | ugaguuggugacgccaggaggu | caccuggcuucauuauaacucugugcucugauagcuuucugaguuggugacgccaggaggu | manual\_scaffold\_12:115509275..115509336:- |
| manual\_scaffold\_11\_36273 | 0.9 | 0.75 ± 0.01 |  | 30 | 30 | 0 | 0 | yes |  |  |  | blast | uccaagggcucuguucuccagu | gggugaacaaagccguugaggu | gggugaacaaagccguugaggucaaaguuacuacuccaagggcucuguucuccagu | manual\_scaffold\_11:542257629..542257685:+ |
| manual\_scaffold\_7\_25736 | 0.9 | 0.75 ± 0.01 |  | 9 | 9 | 0 | 0 | yes |  |  |  | blast | ccggccaaacauacaugcgcacu | ugcgcauguauguuuugcuguuc | ugcgcauguauguuuugcuguuccgagacggccggccaaacauacaugcgcacu | manual\_scaffold\_7:1090853482..1090853536:+ |
| manual\_scaffold\_4a\_14210 | 0.9 | 0.75 ± 0.01 |  | 19 | 19 | 0 | 0 | yes |  |  |  | blast | ucugagcaugcaucugcugga | uagcaaaugcaugauuccgaua | uagcaaaugcaugauuccgauaaucauuuaaagucugagcaugcaucugcugga | manual\_scaffold\_4a:209023850..209023904:- |
| manual\_scaffold\_1b\_3497 | 0.9 | 0.75 ± 0.01 |  | 10 | 10 | 0 | 0 | yes |  |  |  | blast | uuuuuaguacuucuggcuuacu | uaagcaaggagaacuagaagag | uuuuuaguacuucuggcuuacugugacguacuccuggaauucagaaguaagcaaggagaacuagaagag | manual\_scaffold\_1b:814615798..814615867:- |
| manual\_scaffold\_5\_18480 | 0.9 | 0.75 ± 0.01 |  | 51 | 51 | 0 | 0 | yes |  |  |  | blast | uuugaugaaucuguugacauag | augucuauuguuacaucaagag | uuugaugaaucuguugacauagcuaaggcuguaaauaugucuauuguuacaucaagag | manual\_scaffold\_5:1146288117..1146288175:+ |
| manual\_scaffold\_3a\_8925 | 0.9 | 0.75 ± 0.01 |  | 63 | 63 | 0 | 0 | yes |  |  |  | blast | ucacacugacuagucucaaac | uugucacuggucuugguguaaag | ucacacugacuagucucaaacauuuucugguaugcuugucacuggucuugguguaaag | manual\_scaffold\_3a:222982642..222982700:- |
| manual\_scaffold\_9\_31051 | 0.9 | 0.75 ± 0.01 |  | 17 | 17 | 0 | 0 | yes |  |  |  | blast | ucugguugauuuucuaacucu | gguuagaaguauccaucaaaa | gguuagaaguauccaucaaaagaacaaguuacucuuucugguugauuuucuaacucu | manual\_scaffold\_9:185419213..185419270:+ |
| manual\_scaffold\_1a\_955 | 0.9 | 0.75 ± 0.01 |  | 1736 | 1721 | 0 | 15 | yes |  |  |  | blast | uaaggucugacagagacuucu | cggggucuguggcagagcugguga | uaaggucugacagagacuucugaaaauuuuccauucuccggggucuguggcagagcugguga | manual\_scaffold\_1a:73862585..73862647:- |
| manual\_scaffold\_1b\_3409 | 0.9 | 0.75 ± 0.01 |  | 18 | 18 | 0 | 0 | yes |  |  |  | blast | agucacaaauucugagcuaccu | guaggccgaauugcgacccu | agucacaaauucugagcuaccucauuaauauuuaugagguaggccgaauugcgacccu | manual\_scaffold\_1b:706297927..706297985:- |
| manual\_scaffold\_1a\_1286 | 0.9 | 0.75 ± 0.01 |  | 45 | 45 | 0 | 0 | no |  | xtr-miR-30a-3p |  | blast | cuuucagucggauguuug | gagauccccuugccggug | gagauccccuugccggugggugcaggagugacuacaucugcuuucagucggauguuug | manual\_scaffold\_1a:501519897..501519955:- |
| manual\_scaffold\_5\_18604 | 0.9 | 0.75 ± 0.01 |  | 11 | 11 | 0 | 0 | yes |  |  |  | blast | uaguuggcuaaaugaacaaua | uuguucauuuugccaaguaca | uaguuggcuaaaugaacaauagacuuuuacacccauauuguucauuuugccaaguaca | manual\_scaffold\_5:1311831165..1311831223:+ |
| manual\_scaffold\_3a\_9017 | 0.9 | 0.75 ± 0.01 |  | 14 | 11 | 0 | 3 | yes |  |  |  | blast | auggcaaagucaauggaucuu | ccuauuggcuuugccaaugcuu | auggcaaagucaauggaucuugcauacccgagaccuauuggcuuugccaaugcuu | manual\_scaffold\_3a:293419825..293419880:- |
| manual\_scaffold\_11\_36877 | 0.9 | 0.75 ± 0.01 |  | 55 | 55 | 0 | 0 | yes |  |  |  | blast | ggaacaagauccuguuuggauu | uccaaacagcgccuuggcucuuu | ggaacaagauccuguuuggauucucucauaggcuaagaauccaaacagcgccuuggcucuuu | manual\_scaffold\_11:57507794..57507856:- |
| manual\_scaffold\_3a\_9457 | 0.9 | 0.75 ± 0.01 |  | 32 | 32 | 0 | 0 | yes |  |  |  | blast | uaagaaauuauaacuaguagccu | gcacaaguuauaguuucuucag | uaagaaauuauaacuaguagccuaaaguuacuuuccggcacaaguuauaguuucuucag | manual\_scaffold\_3a:446661410..446661469:- |
| manual\_scaffold\_1a\_769 | 0.9 | 0.75 ± 0.01 |  | 20 | 20 | 0 | 0 | yes |  |  |  | blast | cgaaauuaugacaugaggccu | gucucauaucauaguugug | gucucauaucauaguugugcaaagucugaccuuuugugcgaaauuaugacaugaggccu | manual\_scaffold\_1a:988636693..988636752:+ |
| manual\_scaffold\_12\_37831 | 0.8 | 0.75 ± 0.01 |  | 20 | 20 | 0 | 0 | yes |  |  |  | blast | aguuuaccuagaacuccuguuu | acagcaguuauagggaaauggu | acagcaguuauagggaaaugguacucgcugauccuucuuggugcaguuuaccuagaacuccuguuu | manual\_scaffold\_12:20500296..20500362:+ |
| manual\_scaffold\_6\_22365 | 0.8 | 0.75 ± 0.01 |  | 81 | 44 | 0 | 37 | yes |  |  |  | blast | aguucugggacuuguagucuu | uacacauccuagaauucaaag | aguucugggacuuguagucuugucuauuccauaguaaaacagaacuacacauccuagaauucaaag | manual\_scaffold\_6:1574968720..1574968786:+ |
| manual\_scaffold\_6\_20848 | 0.8 | 0.75 ± 0.01 |  | 22 | 22 | 0 | 0 | yes |  |  |  | blast | ucaggacuacuguucccagagccu | gccugggaagcuuuagaguuguguau | ucaggacuacuguucccagagccuaaaaaagcaugcacuggaugcgcuccucaugcaugggccugggaagcuuuagaguuguguau | manual\_scaffold\_6:152271236..152271322:+ |
| manual\_scaffold\_4b\_15546 | 0.8 | 0.75 ± 0.01 |  | 17 | 17 | 0 | 0 | yes |  |  |  | blast | cgcuuggccauucuuaaacucu | aguuuggagcugccaaaaggu | cgcuuggccauucuuaaacucugugcuauaauagcuaguagaguuuggagcugccaaaaggu | manual\_scaffold\_4b:675283550..675283612:+ |
| manual\_scaffold\_6\_22070 | 0.8 | 0.75 ± 0.01 |  | 1247 | 1226 | 0 | 21 | yes |  |  |  | blast | ucgaaguauccuugucuguaaucu | acagggaggauacuugugaggccu | ucgaaguauccuugucuguaaucuguguuuaucaccagaacacagggaggauacuugugaggccu | manual\_scaffold\_6:1269505004..1269505069:+ |
| manual\_scaffold\_4a\_12977 | 0.8 | 0.75 ± 0.01 |  | 11 | 11 | 0 | 0 | yes |  |  |  | blast | uuugaagugcuucuuuguuuggu | ugaacaaguguacuuucucuuaauu | ugaacaaguguacuuucucuuaauuauauguaguuugaagugcuucuuuguuuggu | manual\_scaffold\_4a:295550255..295550311:+ |
| manual\_scaffold\_9\_33018 | 0.8 | 0.75 ± 0.01 |  | 15 | 13 | 0 | 2 | yes |  |  |  | blast | uaccagugugauccucugccu | ugacagaggaaacacuuguugc | ugacagaggaaacacuuguugcugaagcaaacguaccagugugauccucugccu | manual\_scaffold\_9:947575360..947575414:- |
| manual\_scaffold\_7\_26139 | 0.8 | 0.75 ± 0.01 |  | 19 | 19 | 0 | 0 | yes |  |  |  | blast | gugaaccaucuggauuga | aaucaagacucuguucucau | aaucaagacucuguucucaugcugcuaagcagcaugugugaaccaucuggauuga | manual\_scaffold\_7:1394361925..1394361980:+ |
| manual\_scaffold\_10\_34432 | 0.8 | 0.75 ± 0.01 |  | 11 | 11 | 0 | 0 | yes |  |  |  | blast | ugaauuaccaacuaacauacu | ucugguauuuuguaauucaca | ugaauuaccaacuaacauacucuucaauaguagagucugguauuuuguaauucaca | manual\_scaffold\_10:1101655822..1101655878:+ |
| manual\_scaffold\_7\_25603 | 0.8 | 0.75 ± 0.01 |  | 9 | 9 | 0 | 0 | yes |  |  |  | blast | uuggcggcccucugccccacu | ugucucagggggcugucaauu | ugucucagggggcugucaauugguagcgcuuucgcgcgcuacuagauuggcggcccucugccccacu | manual\_scaffold\_7:967517583..967517650:+ |
| manual\_scaffold\_2a\_3837 | 0.8 | 0.75 ± 0.01 |  | 21 | 21 | 0 | 0 | yes |  |  |  | blast | aacucugacacaggugcu | cagucuucaggguugg | cagucuucaggguugggugacaguuuucggacuguauuauauccaacucugacacaggugcu | manual\_scaffold\_2a:30285999..30286061:+ |
| manual\_scaffold\_7\_27343 | 0.8 | 0.75 ± 0.01 |  | 29 | 29 | 0 | 0 | yes |  |  |  | blast | aaucggaauugcugaaacagc | ugauucaucauuucugacugu | aaucggaauugcugaaacagccugaaacugaaggcugauucaucauuucugacugu | manual\_scaffold\_7:829897096..829897152:- |
| manual\_scaffold\_3a\_8682 | 0.8 | 0.75 ± 0.01 |  | 10 | 10 | 0 | 0 | yes |  |  |  | blast | gugcuggggcaggaggggc | gucucugagccuuucugcagccc | gugcuggggcaggaggggcaucaaaggaaaggccuuugaugugucucugagccuuucugcagccc | manual\_scaffold\_3a:1013830593..1013830658:+ |
| manual\_scaffold\_10\_33973 | 0.8 | 0.75 ± 0.01 |  | 182 | 182 | 0 | 0 | yes |  |  |  | blast | uagaccuguuggcauuguaaauu | uuugcaauguaaugggucucgc | uuugcaauguaaugggucucgcauuuccuccaguuagaccuguuggcauuguaaauu | manual\_scaffold\_10:570899615..570899672:+ |
| manual\_scaffold\_4b\_16696 | 0.8 | 0.75 ± 0.01 |  | 22 | 22 | 0 | 0 | yes |  |  |  | blast | uuaaagcugcaaaagacauguu | cauguuuuuucauggagcuaaaagu | cauguuuuuucauggagcuaaaaguguguaauauaaacuuaaagcugcaaaagacauguu | manual\_scaffold\_4b:209748394..209748454:- |
| manual\_scaffold\_3a\_7655 | 0.8 | 0.75 ± 0.01 |  | 310 | 310 | 0 | 0 | yes |  |  |  | blast | augagguggaguuugaguucu | gacacaagcugccacagccauug | gacacaagcugccacagccauuguguagaccuuuucaaugagguggaguuugaguucu | manual\_scaffold\_3a:340176509..340176567:+ |
| manual\_scaffold\_10\_33654 | 0.8 | 0.75 ± 0.01 |  | 5 | 5 | 0 | 0 | yes |  | xtr-miR-146b |  | blast | ugagaacuucacacauaaaag | uuuagguguguagaucucuau | ugagaacuucacacauaaaagaacacaccuguguacacuuuuagguguguagaucucuau | manual\_scaffold\_10:158814009..158814069:+ |
| manual\_scaffold\_10\_35538 | 0.8 | 0.75 ± 0.01 |  | 15 | 15 | 0 | 0 | yes |  |  |  | blast | aaugauugagacuuguucuug | ggaacaacugccagucauggu | ggaacaacugccagucaugguucauaaaacaaugauugagacuuguucuug | manual\_scaffold\_10:880894750..880894801:- |
| manual\_scaffold\_4a\_13858 | 0.8 | 0.75 ± 0.01 |  | 5093 | 5093 | 0 | 0 | yes |  |  |  | blast | ggggauguagcucagugg | acugaauauaucuacuc | acugaauauaucuacucacucauauaugacacaagagugaguacuucacauaccaggggauguagcucagugg | manual\_scaffold\_4a:75647970..75648043:- |
| manual\_scaffold\_2b\_6992 | 0.8 | 0.75 ± 0.01 |  | 9 | 7 | 0 | 2 | no |  |  |  | blast | ucucaacaagacuccagugcgg | uaacuguggcuuuguugcggugu | uaacuguggcuuuguugcggugugccguucaucucaacaagacuccagugcgg | manual\_scaffold\_2b:579723885..579723938:- |
| manual\_scaffold\_3a\_7678 | 0.8 | 0.75 ± 0.01 |  | 2227 | 2227 | 0 | 0 | yes |  | xtr-miR-138 |  | blast | agcugguguugugaaucag | gguuaaacuuuccagcugg | agcugguguugugaaucaggucaaguuuccucucaaaauugguuaaacuuuccagcugg | manual\_scaffold\_3a:350601293..350601352:+ |
| manual\_scaffold\_6\_21607 | 0.8 | 0.75 ± 0.01 |  | 17 | 17 | 0 | 0 | yes |  |  |  | blast | ugacgcacaauggaucuaagga | cuuagcaccaucuuugcaucaua | cuuagcaccaucuuugcaucauauuuaaaaaaugacgcacaauggaucuaagga | manual\_scaffold\_6:793433803..793433857:+ |
| manual\_scaffold\_9\_31735 | 0.8 | 0.75 ± 0.01 |  | 15 | 15 | 0 | 0 | yes |  |  |  | blast | auaguuccauuugcugcccacu | ugggucgcaaauugcgaccuaccu | ugggucgcaaauugcgaccuaccuugugaauauugaugagauaguuccauuugcugcccacu | manual\_scaffold\_9:834323391..834323453:+ |
| manual\_scaffold\_3a\_9805 | 0.8 | 0.75 ± 0.01 |  | 47 | 47 | 0 | 0 | yes |  |  |  | blast | cacuugggcauucuuuaacucu | aguuuggagcuuccaagaggu | cacuugggcauucuuuaacucugugcuauaauagcuaguagaguuuggagcuuccaagaggu | manual\_scaffold\_3a:785262405..785262467:- |
| manual\_scaffold\_11\_37006 | 0.8 | 0.75 ± 0.01 |  | 18 | 18 | 0 | 0 | yes |  |  |  | blast | uuuugauucugagcaacu | auguuagggcaagaga | auguuagggcaagagaugccuuccuuggaacugguugagcaucuuuugauucugagcaacu | manual\_scaffold\_11:146579496..146579557:- |
| manual\_scaffold\_8\_29926 | 0.8 | 0.75 ± 0.01 |  | 33 | 33 | 0 | 0 | yes |  |  |  | blast | ucaaaugcggacugaaagauu | ucucucagcacugcguuuguca | ucucucagcacugcguuugucauaaguauuguaccugucaaaugcggacugaaagauu | manual\_scaffold\_8:276432633..276432691:- |
| manual\_scaffold\_5\_19275 | 0.8 | 0.75 ± 0.01 |  | 11 | 11 | 0 | 0 | yes |  |  |  | blast | uuuccgagcugugcugucu | auguguaucaguucaauu | uuuccgagcugugcugucuggaaacgauguguaucaguucaauu | manual\_scaffold\_5:164937008..164937052:- |
| manual\_scaffold\_3b\_11830 | 0.8 | 0.75 ± 0.01 |  | 10 | 10 | 0 | 0 | yes |  |  |  | blast | uaaugggccacagaacgcaagu | uugcgcuguguggcuccuaauauuucu | uugcgcuguguggcuccuaauauuucugcagcacaguaaugggccacagaacgcaagu | manual\_scaffold\_3b:785069442..785069500:- |
| manual\_scaffold\_12\_39835 | 0.7 | 0.75 ± 0.01 |  | 16 | 16 | 0 | 0 | yes |  |  |  | blast | uuugaauucugugaccuguaguc | cuacaagucccauaauucaaagu | uuugaauucugugaccuguagucuuguuuauuccauuagaaaacaagacuacaagucccauaauucaaagu | manual\_scaffold\_12:635200360..635200431:- |
| manual\_scaffold\_5\_19778 | 0.7 | 0.75 ± 0.01 |  | 61 | 61 | 0 | 0 | no |  | xtr-miR-428b |  | blast | aagaaagugguagauugguu | ccgauguuuaugggacugcuguug | ccgauguuuaugggacugcuguugaugagccaaagaaagugguagauugguu | manual\_scaffold\_5:789966488..789966540:- |
| manual\_scaffold\_2a\_4136 | 0.7 | 0.75 ± 0.01 |  | 32 | 21 | 11 | 0 | yes |  |  |  | blast | agccacuacuguaaagaacaag | uguuucagagaagggcacu | agccacuacuguaaagaacaaggauaucugaaaguucuuggaugguaucuauguuucagagaagggcacu | manual\_scaffold\_2a:428372856..428372926:+ |
| manual\_scaffold\_12\_38941 | 0.7 | 0.75 ± 0.01 |  | 23 | 23 | 0 | 0 | yes |  |  |  | blast | ucuauuuccauugcauugugacu | uccuauccgauggaaauucugc | ucuauuuccauugcauugugacuuauaucccagaaguccuauccgauggaaauucugc | manual\_scaffold\_12:28320353..28320411:- |
| manual\_scaffold\_7\_27811 | 0.7 | 0.75 ± 0.01 |  | 26 | 26 | 0 | 0 | yes |  |  |  | blast | ucagggucugacacugacagcc | cuucagggcgugacccucacc | ucagggucugacacugacagccaguaaguaagacugggcuucagggcgugacccucacc | manual\_scaffold\_7:1264471084..1264471143:- |
| manual\_scaffold\_7\_25478 | 0.7 | 0.75 ± 0.01 |  | 65 | 65 | 0 | 0 | yes |  |  |  | blast | ugcggaauuuggcuagaacucugc | ugagucaccaaauuccaucaa | ugcggaauuuggcuagaacucugcgugguaagaauaaugcugagucaccaaauuccaucaa | manual\_scaffold\_7:845979510..845979571:+ |
| manual\_scaffold\_2a\_4304 | 0.7 | 0.75 ± 0.01 |  | 30 | 30 | 0 | 0 | yes |  |  |  | blast | uguuaaaaaaauaggacu | gacugugugagugacaag | uguuaaaaaaauaggacuugaaguagcaggacugugugagugacaag | manual\_scaffold\_2a:673650277..673650324:+ |
| manual\_scaffold\_4b\_17121 | 0.7 | 0.75 ± 0.01 |  | 15 | 15 | 0 | 0 | yes |  |  |  | blast | aauucugcgaugacugugacu | ccacagguaacgccgaaaaaa | aauucugcgaugacugugacugcuuaagaaagaugaggccacagguaacgccgaaaaaa | manual\_scaffold\_4b:835201624..835201683:- |
| manual\_scaffold\_3a\_9471 | 0.7 | 0.75 ± 0.01 |  | 16 | 16 | 0 | 0 | yes |  |  |  | blast | ugaauugcaauuagaaagggau | ccccuccuaacugcuauucucu | ugaauugcaauuagaaagggaugugaucaggccauccccuccuaacugcuauucucu | manual\_scaffold\_3a:459556312..459556369:- |
| manual\_scaffold\_5\_19029 | 0.7 | 0.75 ± 0.01 |  | 10 | 10 | 0 | 0 | yes |  |  |  | blast | caugccuguggugcucugauu | acagggaccccacagguaacaugug | acagggaccccacagguaacauguguuguuuuuguuaaacacaugccuguggugcucugauu | manual\_scaffold\_5:1827206585..1827206647:+ |
| manual\_scaffold\_2a\_4270 | 0.7 | 0.75 ± 0.01 |  | 11 | 11 | 0 | 0 | yes |  |  |  | blast | cgccuggcuucaucauuacucugu | agaguuggugacgcccugagguggg | cgccuggcuucaucauuacucugugcuauaauagcugucagaguuggugacgcccugagguggg | manual\_scaffold\_2a:635860363..635860427:+ |
| manual\_scaffold\_4b\_17269 | 0.7 | 0.75 ± 0.01 |  | 434 | 434 | 0 | 0 | yes |  |  |  | blast | uuuucauacaccgcucugaagg | uucagaugagugucugagaaua | uuuucauacaccgcucugaagggacacugcaaggcuuucagaugagugucugagaaua | manual\_scaffold\_4b:994230931..994230989:- |
| manual\_scaffold\_5\_20286 | 0.7 | 0.75 ± 0.01 |  | 9 | 9 | 0 | 0 | yes |  |  |  | blast | acuauguggauuuggccgucu | acggccaaauccaaguagucc | acggccaaauccaaguaguccaaaaacggacuauguggauuuggccgucu | manual\_scaffold\_5:1463794871..1463794921:- |
| manual\_scaffold\_4a\_14791 | 0.7 | 0.75 ± 0.01 |  | 34 | 34 | 0 | 0 | yes |  |  |  | blast | cacuuggcccuucuuuaacucu | aguuuggagaugccaagaggu | cacuuggcccuucuuuaacucugugcuauaauagcuaguggaguuuggagaugccaagaggu | manual\_scaffold\_4a:884244179..884244241:- |
| manual\_scaffold\_1a\_1285 | 0.7 | 0.75 ± 0.01 |  | 45 | 45 | 0 | 0 | no |  | xtr-miR-30a-3p |  | blast | cuuucagucggauguuug | uccauccagaggaccgcu | cuuucagucggauguuuggagggggccacgggaccccauuuuauccauccagaggaccgcu | manual\_scaffold\_1a:501519854..501519915:- |
| manual\_scaffold\_7\_25568 | 0.7 | 0.75 ± 0.01 |  | 127 | 127 | 0 | 0 | yes |  |  |  | blast | gauguacgcaaggauuuuaccc | gaaaaagcuuucguacccaug | gauguacgcaaggauuuuacccauucugugucuaugggaaaaagcuuucguacccaug | manual\_scaffold\_7:944472207..944472265:+ |
| manual\_scaffold\_6\_21371 | 0.7 | 0.75 ± 0.01 |  | 24 | 24 | 0 | 0 | yes |  |  |  | blast | uucuguauccugggcaua | uguaaucaggaucagaggg | uguaaucaggaucagagggaggagguuuguacuuuuucuguauccugggcaua | manual\_scaffold\_6:549550628..549550681:+ |
| manual\_scaffold\_11\_37557 | 0.7 | 0.75 ± 0.01 |  | 74 | 73 | 0 | 1 | yes |  |  |  | blast | uggcugaaaccucguccugggu | ucccaagaugagauuuccagc | uggcugaaaccucguccugggugcugccaucuucccaagaugagauuuccagc | manual\_scaffold\_11:888520327..888520380:- |
| manual\_scaffold\_6\_20990 | 0.7 | 0.75 ± 0.01 |  | 7794 | 7794 | 0 | 0 | no |  | xtr-miR-203 |  | blast | gugaaauguuuaggacca | gggaggggggucaccu | gugaaauguuuaggaccacauccacugaggugucagcuggcuucauguggggaggggggucaccu | manual\_scaffold\_6:234616897..234616962:+ |
| manual\_scaffold\_3b\_10563 | 0.7 | 0.75 ± 0.01 |  | 13 | 13 | 0 | 0 | yes |  |  |  | blast | aaaguguuccuuuguuuggug | ccaaucaaggauucuuccu | ccaaucaaggauucuuccuuuuaauuauauguagaaaguguuccuuuguuuggug | manual\_scaffold\_3b:663747367..663747422:+ |
| manual\_scaffold\_3b\_11096 | 0.7 | 0.75 ± 0.01 |  | 83 | 83 | 0 | 0 | yes |  |  |  | blast | uacacugcauugguauggaugag | caucuuaccagugaaggugauc | uacacugcauugguauggaugagauacguuauacuucaucuuaccagugaaggugauc | manual\_scaffold\_3b:1230348000..1230348058:+ |
| manual\_scaffold\_12\_39929 | 0.7 | 0.75 ± 0.01 |  | 14 | 14 | 0 | 0 | yes |  |  |  | blast | accuccacacauacguaccuug | agguauguaaauguggcguuaa | accuccacacauacguaccuuggugauauauuuucgagguauguaaauguggcguuaa | manual\_scaffold\_12:671305961..671306019:- |
| manual\_scaffold\_7\_25264 | 0.7 | 0.75 ± 0.01 |  | 18 | 18 | 0 | 0 | yes |  |  |  | blast | ugucaaugaugcugcuuuu | guucggucucgucaacauu | ugucaaugaugcugcuuuuggcgucagacucuccugccugcgccaguucggucucgucaacauu | manual\_scaffold\_7:638136663..638136727:+ |
| manual\_scaffold\_8\_30451 | 0.7 | 0.75 ± 0.01 |  | 18 | 15 | 0 | 3 | yes |  |  |  | blast | uagaaacuagccuuggucacu | ugugacaaguccuagguucugu | uagaaacuagccuuggucacuaacaauaaaugugugacaaguccuagguucugu | manual\_scaffold\_8:1039967525..1039967579:- |
| manual\_scaffold\_7\_26066 | 0.7 | 0.75 ± 0.01 |  | 22 | 22 | 0 | 0 | no |  | xtr-miR-451 |  | blast | uaaccguuaagcuguggagcuuuu | cagcccaaguuggaggagggugca | uaaccguuaagcuguggagcuuuuguucuuucccgccaucccccagacagcgugaaggccagcccaaguuggaggagggugca | manual\_scaffold\_7:1341610851..1341610934:+ |
| manual\_scaffold\_3a\_9844 | 0.7 | 0.75 ± 0.01 |  | 310 | 310 | 0 | 0 | yes |  |  |  | blast | aggcuaauuuuuggaaagu | uuuccaggaaguagcuga | uuuccaggaaguagcugaggucgaucaggcuaauuuuuggaaagu | manual\_scaffold\_3a:818973117..818973162:- |
| manual\_scaffold\_4b\_16774 | 0.7 | 0.75 ± 0.01 |  | 13 | 13 | 0 | 0 | yes |  |  |  | blast | cccucugguagcuuggcaca | agucaagcugagcuuagaggca | cccucugguagcuuggcacaaagcagucaagcugagcuuagaggca | manual\_scaffold\_4b:329969647..329969693:- |
| manual\_scaffold\_2a\_4799 | 0.6 | 0.75 ± 0.01 |  | 13 | 13 | 0 | 0 | yes |  |  |  | blast | auaucauucugaggugcc | cuccuggcccagauaaag | cuccuggcccagauaaaguuccccggggagcuauaucauucugaggugcc | manual\_scaffold\_2a:1338173984..1338174034:+ |
| manual\_scaffold\_5\_17999 | 0.6 | 0.75 ± 0.01 |  | 10 | 10 | 0 | 0 | yes |  |  |  | blast | ugacuggccucugagcgcauc | ugcguucagaggucacgcc | ugcguucagaggucacgcccuggauaauugcucacaguagugacuggccucugagcgcauc | manual\_scaffold\_5:478949229..478949290:+ |
| manual\_scaffold\_9\_32123 | 0.6 | 0.75 ± 0.01 |  | 10 | 10 | 0 | 0 | yes |  |  |  | blast | aguuauuacacuguccagccuc | gucuggauaauguaauuaccuua | gucuggauaauguaauuaccuuauugguuauuuuauuaaguuauuacacuguccagccuc | manual\_scaffold\_9:1153079866..1153079926:+ |
| manual\_scaffold\_10\_34917 | 0.6 | 0.75 ± 0.01 |  | 3 | 3 | 0 | 0 | yes |  | xtr-miR-30b |  | blast | uguaaacauccugacugg | aucuuggaaaaucuacaga | uguaaacauccugacuggcuuccaacacuucaagaaggaaguguuggcagccaucuuggaaaaucuacaga | manual\_scaffold\_10:268432196..268432267:- |
| manual\_scaffold\_11\_37552 | 0.6 | 0.75 ± 0.01 |  | 19 | 19 | 0 | 0 | yes |  |  |  | blast | cuguguggcuacuguagucagcu | cuguuacagaaguucuucagug | cuguuacagaaguucuucagugcaauacgugccacuguguggcuacuguagucagcu | manual\_scaffold\_11:885802217..885802274:- |
| manual\_scaffold\_8\_30792 | 0.6 | 0.75 ± 0.01 |  | 25 | 25 | 0 | 0 | yes |  |  |  | blast | ucaugacuauaaaugccgaacu | uuauggcauuuuuaauuauguuu | uuauggcauuuuuaauuauguuuucaucuuugagcaucaugacuauaaaugccgaacu | manual\_scaffold\_8:1494678595..1494678653:- |
| manual\_scaffold\_3b\_10147 | 0.6 | 0.75 ± 0.01 |  | 10 | 10 | 0 | 0 | yes |  |  |  | blast | ucugacaugaaaccgggccu | ggucggaugcaugucaaaau | ggucggaugcaugucaaaauggauauccuguaacucugacaugaaaccgggccu | manual\_scaffold\_3b:184911214..184911268:+ |
| manual\_scaffold\_9\_33186 | 0.6 | 0.75 ± 0.01 |  | 9 | 9 | 0 | 0 | yes |  |  |  | blast | uggcuccuucuguauuacc | uauuacagaaggggccgca | uauuacagaaggggccgcacaagcaucuguggcuccuucuguauuacc | manual\_scaffold\_9:1120177550..1120177598:- |
| manual\_scaffold\_6\_24074 | 0.6 | 0.75 ± 0.01 |  | 10 | 10 | 0 | 0 | yes |  |  |  | blast | ugauuucaguuccugccucgggu | uggaggcaguggguggauuagg | uggaggcaguggguggauuaggguugauuucaccugauuucaguuccugccucgggu | manual\_scaffold\_6:1162289507..1162289564:- |
| manual\_scaffold\_11\_37650 | 0.6 | 0.75 ± 0.01 |  | 11 | 7 | 0 | 4 | yes |  |  |  | blast | ucccgagccucacguucucaga | ugugagaacagaagaaacuuggaga | ugugagaacagaagaaacuuggagaguggccaauucucccgagccucacguucucaga | manual\_scaffold\_11:954256948..954257006:- |
| manual\_scaffold\_10\_33401 | 0.6 | 0.75 ± 0.01 |  | 11 | 11 | 0 | 0 | yes |  |  |  | blast | aauuucucuaaaccuugguguu | ugccaagguuaagggaaaauga | ugccaagguuaagggaaaaugaaagacuggaaaauaauuucucuaaaccuugguguu | manual\_scaffold\_10:32942068..32942125:+ |
| manual\_scaffold\_3b\_10334 | 0.6 | 0.75 ± 0.01 |  | 11 | 11 | 0 | 0 | yes |  |  |  | blast | agguucagacauuugguuu | gccacaaugcugagagaga | agguucagacauuugguuucugggugccugauuuggccacaaugcugagagaga | manual\_scaffold\_3b:387264006..387264060:+ |
| manual\_scaffold\_2b\_6785 | 0.6 | 0.75 ± 0.01 |  | 28 | 28 | 0 | 0 | yes |  |  |  | blast | ugcugcacuugacuagucu | aaaugucaaugaaguugu | ugcugcacuugacuagucugcugagagagcauaccuuggcugugccauuaugcauuccagaaaugucaaugaaguugu | manual\_scaffold\_2b:328177370..328177448:- |
| manual\_scaffold\_4b\_16208 | 0.6 | 0.75 ± 0.01 |  | 17 | 15 | 0 | 2 | yes |  |  |  | blast | uaaggaccacuaacgauuacu | uauuuuuguuagucggacuugu | uauuuuuguuagucggacuugugaauuacauuauauuacugcauaaggaccacuaacgauuacu | manual\_scaffold\_4b:51873762..51873826:- |
| manual\_scaffold\_6\_24484 | 0.6 | 0.75 ± 0.01 |  | 14 | 14 | 0 | 0 | yes |  |  |  | blast | uacugacaacuaaucaugaugu | auuaugacuaguugucauuaug | auuaugacuaguugucauuaugccccauuaucacaucccauccaacuauacauacugacaacuaaucaugaugu | manual\_scaffold\_6:1673927134..1673927208:- |
| manual\_scaffold\_8\_29338 | 0.5 | 0.75 ± 0.01 |  | 87 | 87 | 0 | 0 | yes |  |  |  | blast | ugcugcaucgaaaaauccggc | cauuuuuuuaugcauccca | ugcugcaucgaaaaauccggcgcauaaccuccucugcgucauuuuuuuaugcauccca | manual\_scaffold\_8:1151222145..1151222203:+ |
| manual\_scaffold\_7\_25514 | 0.5 | 0.75 ± 0.01 |  | 23 | 23 | 0 | 0 | yes |  |  |  | blast | uucugagacuugugguguucu | aaggcuacaagacccagcauu | uucugagacuugugguguucuuuuauauuggaauaaagaaggcuacaagacccagcauu | manual\_scaffold\_7:907067702..907067761:+ |
| manual\_scaffold\_10\_34998 | 0.5 | 0.75 ± 0.01 |  | 4467 | 4467 | 0 | 0 | no |  | xtr-miR-142-3p |  | blast | cguaguguagugguuauc | uuccaguacaaugca | uuccaguacaaugcauagccacaugaagcaaaccagcaagugugucuguuugcguaguguagugguuauc | manual\_scaffold\_10:330538738..330538808:- |
| manual\_scaffold\_7\_25864 | 0.5 | 0.75 ± 0.01 |  | 10 | 10 | 0 | 0 | yes |  |  |  | blast | cgaggacaaauaaaaugauaau | uaucauuuuauuuuuccuuuuu | cgaggacaaauaaaaugauaauaacauaauguuauuaucauuuuauuuuuccuuuuu | manual\_scaffold\_7:1207067556..1207067613:+ |
| manual\_scaffold\_6\_21401 | 0.5 | 0.75 ± 0.01 |  | 9 | 9 | 0 | 0 | yes |  |  |  | blast | ucacaaucccggauccagaaa | ucuggauuugggauuaccagg | ucuggauuugggauuaccaggccuacacaccaggggucucacaaucccggauccagaaa | manual\_scaffold\_6:581348601..581348660:+ |
| manual\_scaffold\_1a\_940 | 0.5 | 0.75 ± 0.01 |  | 10 | 10 | 0 | 0 | yes |  |  |  | blast | caauucagggaucggucuu | gaagauccucuacacauggc | gaagauccucuacacauggcucugaggaacaaguaccuuaccaauucagggaucggucuu | manual\_scaffold\_1a:29235015..29235075:- |
| manual\_scaffold\_3a\_9766 | 0.5 | 0.75 ± 0.01 |  | 157 | 157 | 0 | 0 | yes |  |  |  | blast | gucugagcaaacaucuguugga | uaguggaugcaggguugagauaa | uaguggaugcaggguugagauaaucaguuaaagucugagcaaacaucuguugga | manual\_scaffold\_3a:717306582..717306636:- |
| manual\_scaffold\_2b\_7174 | 0.5 | 0.75 ± 0.01 |  | 11 | 11 | 0 | 0 | yes |  |  |  | blast | uguagguuggacugugacc | uugcaauuugugaccuaccuc | uugcaauuugugaccuaccucuuacauauuaaugauguagguuggacugugacc | manual\_scaffold\_2b:807542525..807542579:- |
| manual\_scaffold\_9\_32988 | 0.5 | 0.75 ± 0.01 |  | 11 | 10 | 0 | 1 | no |  |  |  | blast | uugguuuaacacugcuuucaca | ugaacacagugcuuggccuagu | ugaacacagugcuuggccuagugguuagcugcuugguuuaacacugcuuucaca | manual\_scaffold\_9:900924522..900924576:- |
| manual\_scaffold\_9\_32484 | 0.5 | 0.75 ± 0.01 |  | 9 | 9 | 0 | 0 | yes |  |  |  | blast | gggaucggcuggaagacagcuguc | ccuagccguccucagucggcgggugccgc | ccuagccguccucagucggcgggugccgccuaguuuagagcugggugggaucggcuggaagacagcuguc | manual\_scaffold\_9:307898166..307898236:- |
| manual\_scaffold\_10\_35112 | 0.5 | 0.75 ± 0.01 |  | 13 | 13 | 0 | 0 | yes |  |  |  | blast | cacaguucgggucuguaaaguuc | auucuuugcaaccucaauguugg | auucuuugcaaccucaauguugggcuaaaaaacacuuuuuccucacaguucgggucuguaaaguuc | manual\_scaffold\_10:469808865..469808931:- |
| manual\_scaffold\_1a\_392 | 0.5 | 0.75 ± 0.01 |  | 14 | 14 | 0 | 0 | yes |  |  |  | blast | ucuaagaagcccugacuugacu | uccagacagaugcuucugaggua | ucuaagaagcccugacuugacuauucaaaacuauuuauuguccagacagaugcuucugaggua | manual\_scaffold\_1a:446280911..446280974:+ |
| manual\_scaffold\_12\_39229 | 0.5 | 0.75 ± 0.01 |  | 344 | 344 | 0 | 0 | yes |  |  |  | blast | ugaggacuugcgagacggca | cgguugccuucauccuuacu | ugaggacuugcgagacggcaauguacuccucauugcgguugccuucauccuuacu | manual\_scaffold\_12:281677502..281677557:- |
| manual\_scaffold\_4b\_16232 | 0.5 | 0.75 ± 0.01 |  | 10 | 10 | 0 | 0 | yes |  |  |  | blast | uuuuuggaacuuguuggucaguu | uugaucaacaaguuccacaaaca | uugaucaacaaguuccacaaacauaagguccuaguauuuuuggaacuuguuggucaguu | manual\_scaffold\_4b:83017555..83017614:- |
| manual\_scaffold\_3a\_8505 | 0.5 | 0.75 ± 0.01 |  | 5974 | 5974 | 0 | 0 | no |  | xtr-miR-449c-5p |  | blast | aggcaguguaguuagcugauugu | ucucagcugugaggcauucacuuc | ucucagcugugaggcauucacuucucgaagaacuugugcuugguuuguaggcaguguaguuagcugauugu | manual\_scaffold\_3a:794602788..794602859:+ |
| manual\_scaffold\_10\_33505 | 0.5 | 0.75 ± 0.01 |  | 8 | 7 | 0 | 1 | no |  |  |  | blast | ugucaaccacccgcgcaggag | uccugugauggucgaugacaug | uccugugauggucgaugacaugccguccuuaccugucaaccacccgcgcaggag | manual\_scaffold\_10:70208456..70208510:+ |
| manual\_scaffold\_7\_25521 | 0.5 | 0.75 ± 0.01 |  | 12 | 12 | 0 | 0 | yes |  |  |  | blast | ugcaucuaaagauggccacugu | aguggguaucuuugaaucggauauauu | aguggguaucuuugaaucggauauauuaaaauagacucaaaugcaucuaaagauggccacugu | manual\_scaffold\_7:909990815..909990878:+ |
| manual\_scaffold\_3b\_11980 | 0.5 | 0.75 ± 0.01 |  | 108 | 108 | 0 | 0 | yes |  |  |  | blast | uuagagagacugaacaaagcu | cuuuacucagugccucuacuu | cuuuacucagugccucuacuuuccuuaaucuaaucaauuagagagacugaacaaagcu | manual\_scaffold\_3b:951422500..951422558:- |
| manual\_scaffold\_6\_24168 | 0.4 | 0.75 ± 0.01 |  | 801 | 760 | 0 | 41 | yes |  |  |  | blast | ucgcaaucauguuaguccuca | gaggacuugcgagagagca | gaggacuugcgagagagcaauguauucuucaucgcaaucauguuaguccuca | manual\_scaffold\_6:1319380548..1319380600:- |
| manual\_scaffold\_4b\_16945 | 0.4 | 0.75 ± 0.01 |  | 77 | 77 | 0 | 0 | yes |  |  |  | blast | uccgggauuuguaguuuuuauu | uaaaugggacuauaaaucccagaau | uccgggauuuguaguuuuuauuuucuaaauaaauaaaugggacuauaaaucccagaau | manual\_scaffold\_4b:578223024..578223082:- |
| manual\_scaffold\_1b\_2086 | 0.4 | 0.75 ± 0.01 |  | 9 | 9 | 0 | 0 | yes |  |  |  | blast | accuugugagcagaucggcuua | agcugaucugcccccgggggg | accuugugagcagaucggcuuauuucuauuaagcugaucugcccccgggggg | manual\_scaffold\_1b:339481379..339481431:+ |
| manual\_scaffold\_5\_20477 | 0.4 | 0.75 ± 0.01 |  | 38 | 16 | 6 | 16 | yes |  |  |  | blast | agucuuggaguuguucauuaga | ucuugaacucuuacagucucugu | ucuugaacucuuacagucucugugaauugucuaucagagucuuggaguuguucauuaga | manual\_scaffold\_5:1750770736..1750770795:- |
| manual\_scaffold\_4a\_14108 | 0.4 | 0.75 ± 0.01 |  | 25 | 25 | 0 | 0 | yes |  |  |  | blast | aaucuauuggcuuugucugugau | aacagcaaagcuaauaaauagguggc | aacagcaaagcuaauaaauagguggcaacuaugcaaaaucuauuggcuuugucugugau | manual\_scaffold\_4a:115456751..115456810:- |
| manual\_scaffold\_8\_29538 | 0.4 | 0.75 ± 0.01 |  | 16 | 16 | 0 | 0 | yes |  |  |  | blast | uuccgugacuauauagaggaa | ccuuuuuaaggucaaguaaca | uuccgugacuauauagaggaaugaggcuaaaggcugaguuccuuuuuaaggucaaguaaca | manual\_scaffold\_8:1414639775..1414639836:+ |
| manual\_scaffold\_5\_19204 | 0.4 | 0.75 ± 0.01 |  | 270 | 270 | 0 | 0 | yes |  |  |  | blast | uucagucauuguuucuggu | ugaagaaaugcugcaaag | ugaagaaaugcugcaaagaaaucuaagagauuucuuucagucauuguuucuggu | manual\_scaffold\_5:91729593..91729647:- |
| manual\_scaffold\_2b\_6721 | 0.4 | 0.75 ± 0.01 |  | 9 | 9 | 0 | 0 | yes |  |  |  | blast | ggcggacggauaggucgc | ugccucuccgucccaccuc | ggcggacggauaggucgcacaaucaugccucuccgucccaccuc | manual\_scaffold\_2b:273467778..273467822:- |
| manual\_scaffold\_9\_33185 | 0.4 | 0.75 ± 0.01 |  | 9 | 9 | 0 | 0 | yes |  |  |  | blast | uggcuccuucuguauuacc | aagugcucuggagcucaca | uggcuccuucuguauuaccaaagugcucuggagcucaca | manual\_scaffold\_9:1120177530..1120177569:- |
| manual\_scaffold\_4b\_15355 | 0.4 | 0.75 ± 0.01 |  | 70 | 70 | 0 | 0 | yes |  |  |  | blast | uugcggucaugucaguccucacu | ugaggacuuacaggaaggcaaug | ugaggacuuacaggaaggcaauguaaucuucauugcggucaugucaguccucacu | manual\_scaffold\_4b:489580951..489581006:+ |
| manual\_scaffold\_8\_28851 | 0.4 | 0.75 ± 0.01 |  | 21 | 21 | 0 | 0 | yes |  |  |  | blast | ccauggacuuuguggaca | gccauuguuccaggcu | gccauuguuccaggcuccagacacccuugauucccuucuugggcagccauggacuuuguggaca | manual\_scaffold\_8:385898740..385898804:+ |
| manual\_scaffold\_10\_34983 | 0.4 | 0.75 ± 0.01 |  | 6045 | 6045 | 0 | 0 | yes |  |  |  | blast | uucuagaaguuguuggauacu | ggaccaaacuuuuucuagaaua | uucuagaaguuguuggauacuagaaguacuaucacuggaccaaacuuuuucuagaaua | manual\_scaffold\_10:325971425..325971483:- |
| manual\_scaffold\_3a\_9688 | 0.4 | 0.75 ± 0.01 |  | 43 | 43 | 0 | 0 | yes |  |  |  | blast | aagugcuuucuguugagcuu | gcucaagcuggggcaccugc | gcucaagcuggggcaccugcuauguuuagauuuguaagugcuuucuguugagcuu | manual\_scaffold\_3a:615982453..615982508:- |
| manual\_scaffold\_8\_30234 | 0.4 | 0.75 ± 0.01 |  | 8 | 8 | 0 | 0 | yes |  |  |  | blast | aguauaagagcagugcuuggau | ccuagcacugcucuuauacugc | ccuagcacugcucuuauacugcuugguauuuaauaaggaguauaagagcagugcuuggau | manual\_scaffold\_8:709490610..709490670:- |
| manual\_scaffold\_1b\_1876 | 0.4 | 0.75 ± 0.01 |  | 11 | 11 | 0 | 0 | yes |  |  |  | blast | uagagugguuauucucuguagu | caccaagaauacccauucuggcu | uagagugguuauucucuguaguucuuuccauuguaucaccaagaauacccauucuggcu | manual\_scaffold\_1b:89526270..89526329:+ |
| manual\_scaffold\_3b\_11626 | 0.4 | 0.75 ± 0.01 |  | 126 | 126 | 0 | 0 | yes |  |  |  | blast | ucucucuguaguugcaccucau | aaggugcaguugcacagaaac | aaggugcaguugcacagaaacaugcuuugacguucucucuguaguugcaccucau | manual\_scaffold\_3b:566279228..566279283:- |
| manual\_scaffold\_9\_32739 | 0.4 | 0.75 ± 0.01 |  | 110 | 110 | 0 | 0 | yes |  |  |  | blast | ucugaucucagcuuugucaccu | gugccauacuggaucugaaag | ucugaucucagcuuugucaccuuaugaauuucccuaggugccauacuggaucugaaag | manual\_scaffold\_9:672895064..672895122:- |
| manual\_scaffold\_6\_21902 | 0.4 | 0.75 ± 0.01 |  | 44 | 44 | 0 | 0 | yes |  |  |  | blast | aaugccaauauguagaacagu | uguacuuccucuuggcacuag | aaugccaauauguagaacagugagaauacgcuguacuuccucuuggcacuag | manual\_scaffold\_6:1133032014..1133032066:+ |
| manual\_scaffold\_2b\_6407 | 0.3 | 0.75 ± 0.01 |  | 239 | 239 | 0 | 0 | yes |  |  |  | blast | cugaaguguuucucugaga | ucugaggaauagag | ucugaggaauagaguuuuuguggguauuagagaguuuggaaugcucucuaauuuuacuaauuagucugaaguguuucucugaga | manual\_scaffold\_2b:672606283..672606367:+ |
| manual\_scaffold\_8\_29275 | 0.3 | 0.75 ± 0.01 |  | 13 | 13 | 0 | 0 | yes |  |  |  | blast | guuccccugguagaacacca | guggcccaccaacccuugagaauag | guggcccaccaacccuugagaauaguaaagaauaugaucucuguuccccugguagaacacca | manual\_scaffold\_8:1072113453..1072113515:+ |
| manual\_scaffold\_4b\_16018 | 0.3 | 0.75 ± 0.01 |  | 10 | 10 | 0 | 0 | yes |  |  |  | blast | ugccugugacucuugaaa | gcaagaacaugucgcugcgga | ugccugugacucuugaaagagcaagaacaugucgcugcgga | manual\_scaffold\_4b:1170007432..1170007473:+ |
| manual\_scaffold\_6\_24497 | 0.3 | 0.75 ± 0.01 |  | 14 | 14 | 0 | 0 | yes |  |  |  | blast | aggauaucccauucacuguauu | uauguggacaggauauccgga | aggauaucccauucacuguauuuugugcauuguagccuuuggcacuuguaguauguggacaggauauccgga | manual\_scaffold\_6:1704367358..1704367430:- |
| manual\_scaffold\_6\_21216 | 0.3 | 0.75 ± 0.01 |  | 8 | 7 | 0 | 1 | yes |  |  |  | blast | cacugggauaacuggcguaaacc | cggucagcccaguggaaaac | cacugggauaacuggcguaaaccuugguuuccgcgguuuccgccggucagcccaguggaaaac | manual\_scaffold\_6:368275601..368275664:+ |
| manual\_scaffold\_3b\_11851 | 0.3 | 0.75 ± 0.01 |  | 9 | 9 | 0 | 0 | yes |  |  |  | blast | uuguggacacuugucaggacu | ugccaacagguguccuggaa | uuguggacacuugucaggacuccuguauuacguuggcagugccaacagguguccuggaa | manual\_scaffold\_3b:802994314..802994373:- |
| manual\_scaffold\_10\_33967 | 0.3 | 0.75 ± 0.01 |  | 22 | 22 | 0 | 0 | yes |  |  |  | blast | ucgcagucauguuaguucucacu | ugaggacaugcgagacagcaaug | ugaggacaugcgagacagcaauguacucuucaucgcagucauguuaguucucacu | manual\_scaffold\_10:561316396..561316451:+ |
| manual\_scaffold\_5\_19565 | 0.3 | 0.75 ± 0.01 |  | 24 | 23 | 0 | 1 | yes |  |  |  | blast | ccuuggaugcaaacuugaaugu | ucaguuuugucucccaaagga | ccuuggaugcaaacuugaaugugaauucaaucaaauauucaguuuugucucccaaagga | manual\_scaffold\_5:546276309..546276368:- |
| manual\_scaffold\_6\_23824 | 0.3 | 0.75 ± 0.01 |  | 286 | 286 | 0 | 0 | yes |  |  |  | blast | uccacacucauauuucuacug | auggaaauaugcggaugcaca | auggaaauaugcggaugcacaguaguuaaguuuguccacacucauauuucuacug | manual\_scaffold\_6:889801199..889801254:- |
| manual\_scaffold\_3a\_8496 | 0.3 | 0.75 ± 0.01 |  | 10 | 10 | 0 | 0 | yes |  |  |  | blast | uuugcucaauuugagguuaaaccu | uguuaaccuuugugagcaguuu | uuugcucaauuugagguuaaaccuggguuuagaucaauguuaaccuuugugagcaguuu | manual\_scaffold\_3a:784336321..784336380:+ |
| manual\_scaffold\_8\_28830 | 0.3 | 0.75 ± 0.01 |  | 8 | 8 | 0 | 0 | yes |  |  |  | blast | cuuggucccagcagguugugua | cacaacuugcugggaccaaaca | cuuggucccagcagguuguguaaaaauguaauucuuacacaacuugcugggaccaaaca | manual\_scaffold\_8:357054380..357054439:+ |
| manual\_scaffold\_3a\_9037 | 0.3 | 0.75 ± 0.01 |  | 57380 | 31659 | 13 | 25708 | yes |  |  |  | blast | uugcaguaacaggugugaacauc | aucauguaugauacugcagaca | uugcaguaacaggugugaacaucuuauaagcaguuugaugaucauguaugauacugcagaca | manual\_scaffold\_3a:301645401..301645463:- |
| manual\_scaffold\_10\_33425 | 0.3 | 0.75 ± 0.01 |  | 176 | 176 | 0 | 0 | no |  | xtr-miR-17-5p |  | blast | gaaagugcuguucuguuggg | aaauaccagcacguccuuu | aaauaccagcacguccuuuauuuucaucgauaagaaagugcuguucuguuggg | manual\_scaffold\_10:48071004..48071057:+ |
| manual\_scaffold\_11\_36217 | 0.3 | 0.75 ± 0.01 |  | 8 | 8 | 0 | 0 | yes |  |  |  | blast | aguauaagagcagugcuuggau | ccuagcacugcucuuauguugc | ccuagcacugcucuuauguugcuuuguacuauaugcaguauaagagcagugcuuggau | manual\_scaffold\_11:459739711..459739769:+ |
| manual\_scaffold\_1a\_1528 | 0.3 | 0.75 ± 0.01 |  | 10 | 10 | 0 | 0 | yes |  |  |  | blast | gaagauauguauauguggag | ccauauauacgaaucuuuaa | ccauauauacgaaucuuuaagcuauauauauuuugaagauauguauauguggag | manual\_scaffold\_1a:862827434..862827488:- |
| manual\_scaffold\_7\_25078 | 0.3 | 0.75 ± 0.01 |  | 18 | 11 | 0 | 7 | yes |  |  |  | blast | ucgcaaagcuaacugcucagc | ugagagcuauuagcuuugccagu | ucgcaaagcuaacugcucagcacauacauuauaaaggugagagcuauuagcuuugccagu | manual\_scaffold\_7:426681487..426681547:+ |
| manual\_scaffold\_3b\_10825 | 0.3 | 0.75 ± 0.01 |  | 11 | 11 | 0 | 0 | yes |  |  |  | blast | uauaugcgugacucaaaccu | gcuugagccaugcuugcacc | gcuugagccaugcuugcaccgcgcaaugugguauaugcgugacucaaaccu | manual\_scaffold\_3b:918801279..918801330:+ |
| manual\_scaffold\_4a\_13380 | 0.3 | 0.75 ± 0.01 |  | 17 | 17 | 0 | 0 | yes |  |  |  | blast | uucuggcaccuguaguucugac | cagaacuacgaauggcagaugg | uucuggcaccuguaguucugacuucauaacggggcaaacagaacuacgaauggcagaugg | manual\_scaffold\_4a:709266554..709266614:+ |
| manual\_scaffold\_1a\_1328 | 0.2 | 0.75 ± 0.01 |  | 16 | 16 | 0 | 0 | yes |  |  |  | blast | uuacccucugacaaauucuagc | uagauuuugggagguaaag | uagauuuugggagguaaagugcaaggcugccuuuuacccucugacaaauucuagc | manual\_scaffold\_1a:570245613..570245668:- |
| manual\_scaffold\_5\_18001 | 0.2 | 0.75 ± 0.01 |  | 10 | 10 | 0 | 0 | yes |  |  |  | blast | ugacuggccucugagcgcauc | ugcguucagaggucacgcc | ugcguucagaggucacgcccuggauaauugcucacaguagugacuggccucugagcgcauc | manual\_scaffold\_5:478992577..478992638:+ |
| manual\_scaffold\_12\_38706 | 0.2 | 0.75 ± 0.01 |  | 10 | 10 | 0 | 0 | yes |  |  |  | blast | caccugugcuagacuguuggg | uaacggcuaucauggguc | caccugugcuagacuguugggcuacacaucuacuaaagagcguagcuaacggcuaucauggguc | manual\_scaffold\_12:672866238..672866302:+ |
| manual\_scaffold\_4a\_13955 | 0.2 | 0.75 ± 0.01 |  | 24 | 24 | 0 | 0 | yes |  |  |  | blast | uaucuugaauucaguugcac | guggcugauaaaaagauuuu | uaucuugaauucaguugcacagagugugguggcugauaaaaagauuuu | manual\_scaffold\_4a:92924226..92924274:- |
| manual\_scaffold\_12\_39421 | 0.2 | 0.75 ± 0.01 |  | 8 | 8 | 0 | 0 | yes |  |  |  | blast | ucggaccaaucacaacacugcu | cagcguugugauuggcuggga | ucggaccaaucacaacacugcugucaugcucguuacagcagcguugugauuggcuggga | manual\_scaffold\_12:515025674..515025733:- |
| manual\_scaffold\_9\_32527 | 0.2 | 0.75 ± 0.01 |  | 8 | 8 | 0 | 0 | yes |  |  |  | blast | cgcacucaugaugggaccuc | gguucugucaugaguguggg | cgcacucaugaugggaccucuggaaugaguaacugcagugcgaagguucugucaugaguguggg | manual\_scaffold\_9:358126609..358126673:- |
| manual\_scaffold\_6\_20627 | 0.2 | 0.75 ± 0.01 |  | 8 | 8 | 0 | 0 | yes |  |  |  | blast | uuuauuacuauucugagccc | gcucagagcaguaauaaaca | gcucagagcaguaauaaacauuugaaaggguccgauuguuuauuacuauucugagccc | manual\_scaffold\_6:7699997..7700055:+ |
| manual\_scaffold\_1b\_3247 | 0.2 | 0.75 ± 0.01 |  | 29 | 29 | 0 | 0 | yes |  |  |  | blast | agcuggcugagcaccucuguguu | caccauaggcccucauccauccug | agcuggcugagcaccucuguguucucuuuuacagcaccauaggcccucauccauccug | manual\_scaffold\_1b:441728000..441728058:- |
| manual\_scaffold\_2b\_6962 | 0.2 | 0.75 ± 0.01 |  | 182 | 182 | 0 | 0 | yes |  |  |  | blast | uagaccuguuggcauuguaaauu | uuugcaaugcaucaggucucac | uuugcaaugcaucaggucucacauuuaagcgaguuagaccuguuggcauuguaaauu | manual\_scaffold\_2b:554330310..554330367:- |
| manual\_scaffold\_5\_19578 | 0.2 | 0.75 ± 0.01 |  | 89 | 89 | 0 | 0 | yes |  |  |  | blast | acguaauugcauuguucggcu | ccaacaguauaauuaacuua | ccaacaguauaauuaacuuaauacacuuaacaauaacguaauugcauuguucggcu | manual\_scaffold\_5:564875861..564875917:- |
| manual\_scaffold\_2a\_4049 | 0.2 | 0.75 ± 0.01 |  | 7 | 7 | 0 | 0 | yes |  | xtr-miR-17-5p |  | blast | gaaagugcuguucuguug | gcggagacaugguaaguaagu | gcggagacaugguaaguaaguguauuuaguauuuggcauauacuguaggugcauuuugaaagugcuguucuguug | manual\_scaffold\_2a:304841988..304842063:+ |
| manual\_scaffold\_1a\_596 | 0.2 | 0.75 ± 0.01 |  | 28 | 28 | 0 | 0 | yes |  |  |  | blast | uccagcagugggucaggc | cugacucauaaggugguaa | uccagcagugggucaggcucugacucauaaggugguaa | manual\_scaffold\_1a:731739344..731739382:+ |
| manual\_scaffold\_6\_23281 | 0.2 | 0.75 ± 0.01 |  | 9 | 9 | 0 | 0 | yes |  |  |  | blast | ucgagcaccgggcugguuccgc | agagccuguccuaugcuuggug | ucgagcaccgggcugguuccgcaguguacaugccaucucagagccuguccuaugcuuggug | manual\_scaffold\_6:248992554..248992615:- |
| manual\_scaffold\_2a\_5719 | 0.2 | 0.75 ± 0.01 |  | 12 | 12 | 0 | 0 | yes |  |  |  | blast | cacuugccauucuuuaacucu | aguuuggaacugccaggaggu | cacuugccauucuuuaacucugugcuauaauagcaaguagaguuuggaacugccaggaggu | manual\_scaffold\_2a:1196211044..1196211105:- |
| manual\_scaffold\_4b\_15083 | 0.2 | 0.75 ± 0.01 |  | 9 | 9 | 0 | 0 | yes |  |  |  | blast | aacuugcaaaacgacggacagu | cgucugucgcauuacaaguucu | cgucugucgcauuacaaguucuauuauauucuauggaacuugcaaaacgacggacagu | manual\_scaffold\_4b:191121827..191121885:+ |
| manual\_scaffold\_4a\_14608 | 0.2 | 0.75 ± 0.01 |  | 14 | 14 | 0 | 0 | yes |  |  |  | blast | uagaccuguuggcauuguaaau | uugcaauguaaaaggucucgc | uugcaauguaaaaggucucgcauuagugagaguuagaccuguuggcauuguaaau | manual\_scaffold\_4a:714927524..714927579:- |
| manual\_scaffold\_3a\_7277 | 0.2 | 0.75 ± 0.01 |  | 191 | 191 | 0 | 0 | yes |  |  |  | blast | uaacgugaucaugaugccauca | auggcaucacccucaaguuaca | auggcaucacccucaaguuacagcgucaaugcaaagauguaacgugaucaugaugccauca | manual\_scaffold\_3a:59314092..59314153:+ |
| manual\_scaffold\_9\_31098 | 0.2 | 0.75 ± 0.01 |  | 11 | 11 | 0 | 0 | yes |  |  |  | blast | ucugcaugcagagacuugugagcu | ccuacaagucccagcaugcuacuu | ucugcaugcagagacuugugagcuguuuuucacaucacaggcaaggccuacaagucccagcaugcuacuu | manual\_scaffold\_9:218424075..218424145:+ |
| manual\_scaffold\_6\_20813 | 0.2 | 0.75 ± 0.01 |  | 10 | 8 | 0 | 2 | yes |  |  |  | blast | aacggccuugguuugcgggc | uccaugcaaauuaguugccguuu | uccaugcaaauuaguugccguuuaacucuccuuguuucaacggccuugguuugcgggc | manual\_scaffold\_6:129611342..129611400:+ |
| manual\_scaffold\_7\_25845 | 0.2 | 0.75 ± 0.01 |  | 22 | 21 | 0 | 1 | yes |  |  |  | blast | cuugaaggcccggagacagca | ugcuucucuguguuuucaugu | ugcuucucuguguuuucaugucugcuucaaccucuugaaggcccggagacagca | manual\_scaffold\_7:1175572944..1175572998:+ |
| manual\_scaffold\_5\_19563 | 0.2 | 0.75 ± 0.01 |  | 343 | 343 | 0 | 0 | yes |  |  |  | blast | ugaggacuugcgagacggca | cagucauaucaguucucacu | ugaggacuugcgagacggcaauguacucuucauugcagucauaucaguucucacu | manual\_scaffold\_5:543444884..543444939:- |
| manual\_scaffold\_10\_35043 | 0.2 | 0.75 ± 0.01 |  | 53 | 53 | 0 | 0 | yes |  |  |  | blast | uuucagaaguaaucucagacc | ucuagcauugcugcuggagcc | uuucagaaguaaucucagacccccaguguaauccuggagucuagcauugcugcuggagcc | manual\_scaffold\_10:346613508..346613568:- |
| manual\_scaffold\_6\_22050 | 0.1 | 0.75 ± 0.01 |  | 4812 | 4812 | 0 | 0 | no |  | xtr-let-7a |  | blast | ugagguaguagguugugu | aggacccugcccuguu | ugagguaguagguuguguagacuuaggugcaggacccugcccuguu | manual\_scaffold\_6:1242969056..1242969102:+ |
| manual\_scaffold\_4b\_16908 | 0.1 | 0.75 ± 0.01 |  | 17 | 17 | 0 | 0 | yes |  |  |  | blast | aacggugcaaaaugucuucagc | ugaaaacauuuuguacauuuuu | ugaaaacauuuuguacauuuuuuauuuuauuguaggcaacggugcaaaaugucuucagc | manual\_scaffold\_4b:547631116..547631175:- |
| manual\_scaffold\_4a\_12879 | 0.1 | 0.75 ± 0.01 |  | 10 | 10 | 0 | 0 | yes |  |  |  | blast | uagccaucucugaauguuuuu | aaccauucaacgauggcugcc | aaccauucaacgauggcugccuggcagaugcacaugcacagauagccaucucugaauguuuuu | manual\_scaffold\_4a:159721249..159721312:+ |
| manual\_scaffold\_4a\_14591 | 0.1 | 0.75 ± 0.01 |  | 10 | 10 | 0 | 0 | yes |  |  |  | blast | ggcaguugggcaguauuu | uagcuauccaaaaaguccu | ggcaguugggcaguauuuggcaacccacuccaugcccaucauggucuugacugugagguugugauagcuauccaaaaaguccu | manual\_scaffold\_4a:707747309..707747392:- |
| manual\_scaffold\_1b\_3749 | 0.1 | 0.75 ± 0.01 |  | 20 | 20 | 0 | 0 | yes |  |  |  | blast | uucuagaagucguuagauacu | gauccaacucuuucuagauuagg | uucuagaagucguuagauacuagcgguacuaucacuggauccaacucuuucuagauuagg | manual\_scaffold\_1b:1164831755..1164831815:- |
| manual\_scaffold\_1a\_315 | 0.1 | 0.75 ± 0.01 |  | 191 | 170 | 0 | 21 | yes |  |  |  | blast | uuuugaauaagugagugaau | uuuaacucccuuuaucaguagc | uuuaacucccuuuaucaguagcaguggucaaauucuguuuugaauaagugagugaau | manual\_scaffold\_1a:379954407..379954464:+ |
| manual\_scaffold\_7\_24880 | 0.1 | 0.75 ± 0.01 |  | 8 | 8 | 0 | 0 | yes |  |  |  | blast | uuggcacacuaguaaacuuuga | aaacuuuacuugugugccaaag | uuggcacacuaguaaacuuugagaacaucccgucaaacuuuacuugugugccaaag | manual\_scaffold\_7:156861113..156861169:+ |
| manual\_scaffold\_10\_35829 | 0.1 | 0.75 ± 0.01 |  | 8 | 8 | 0 | 0 | yes |  |  |  | blast | uucccgauauucgucucauacu | uaugagccugguauccggauug | uucccgauauucgucucauacucaguauuguaacacagaguaugagccugguauccggauug | manual\_scaffold\_10:1089779552..1089779614:- |
| manual\_scaffold\_10\_34848 | 0.1 | 0.75 ± 0.01 |  | 9 | 9 | 0 | 0 | yes |  |  |  | blast | ucagacuagugugugacuguc | gaguaacucgcuggucgacug | gaguaacucgcuggucgacuggagucugcacauucuccucagacuagugugugacuguc | manual\_scaffold\_10:236141910..236141969:- |
| manual\_scaffold\_5\_19894 | 0.1 | 0.75 ± 0.01 |  | 8 | 8 | 0 | 0 | yes |  |  |  | blast | cagaaauccgguagugcuc | gcgcuaccugacuuccacc | gcgcuaccugacuuccaccacaguaagggcagaaauccgguagugcuc | manual\_scaffold\_5:957319833..957319881:- |
| manual\_scaffold\_8\_29087 | 0.1 | 0.75 ± 0.01 |  | 13 | 13 | 0 | 0 | yes |  |  |  | blast | ugugugauaugaucauugaca | ucaaugauugugacacaccaa | ucaaugauugugacacaccaaugaaaugaaauucuuaguauacuuugugugauaugaucauugaca | manual\_scaffold\_8:757502889..757502955:+ |
| manual\_scaffold\_4a\_14434 | 0.1 | 0.75 ± 0.01 |  | 10 | 10 | 0 | 0 | yes |  |  |  | blast | ugugauguuagucagaacggcu | ugguucugauugacaugcaac | ugguucugauugacaugcaacugaaguuucacacuugucugugauguuagucagaacggcu | manual\_scaffold\_4a:483929696..483929757:- |
| manual\_scaffold\_7\_24679 | 0.1 | 0.75 ± 0.01 |  | 12 | 12 | 0 | 0 | yes |  |  |  | blast | aagcaagacugauugacauugc | augccagucaguuugauuugc | augccagucaguuugauuugccuuuacauacaagcaagacugauugacauugc | manual\_scaffold\_7:56840103..56840156:+ |
| manual\_scaffold\_2b\_7099 | 0.1 | 0.75 ± 0.01 |  | 9 | 9 | 0 | 0 | yes |  |  |  | blast | accggacucuguauauacuaua | uaguguguacagagucaguccu | accggacucuguauauacuauaccaaaaugcaguauaguguguacagagucaguccu | manual\_scaffold\_2b:713829418..713829475:- |
| manual\_scaffold\_9\_32585 | 0.1 | 0.75 ± 0.01 |  | 71 | 71 | 0 | 0 | yes |  |  |  | blast | acuuggcaacugaagaaa | auuucaguaagguau | auuucaguaagguaucagacuuggcaacugaagaaa | manual\_scaffold\_9:450850991..450851027:- |
| manual\_scaffold\_1b\_3345 | 0.1 | 0.75 ± 0.01 |  | 10 | 10 | 0 | 0 | yes |  |  |  | blast | uuaauguagacagccuagg | agggauugucaguguuuugg | uuaauguagacagccuaggcuuagagacuagccuggcgcaaguuagauucuucugggcaauagggauugucaguguuuugg | manual\_scaffold\_1b:600263804..600263885:- |
| manual\_scaffold\_9\_32581 | 0.1 | 0.75 ± 0.01 |  | 9 | 9 | 0 | 0 | yes |  |  |  | blast | ugcuugaagcuggacugugu | auauuucaguuccucaagcaca | ugcuugaagcuggacugugugcuuucacuguggugcauauuucaguuccucaagcaca | manual\_scaffold\_9:448306654..448306712:- |
| manual\_scaffold\_7\_25341 | 0.1 | 0.75 ± 0.01 |  | 14 | 14 | 0 | 0 | yes |  |  |  | blast | uuagacugacacuccugauagc | uuuugggaguauuagucuuaaau | uuuugggaguauuagucuuaaauugcuuucuuugaauuauuuagacugacacuccugauagc | manual\_scaffold\_7:684819497..684819559:+ |
| manual\_scaffold\_6\_23988 | 0.1 | 0.75 ± 0.01 |  | 8 | 8 | 0 | 0 | yes |  |  |  | blast | cagaaauccgguagugcuc | gcacugccugacuuccgcc | gcacugccugacuuccgccacaauaagggcagaaauccgguagugcuc | manual\_scaffold\_6:1062444498..1062444546:- |
| manual\_scaffold\_1b\_2431 | 0.1 | 0.75 ± 0.01 |  | 48 | 48 | 0 | 0 | yes |  |  |  | blast | ucgcgcucacguuaguccucacc | ugaagacuugcgagagggcaaug | ugaagacuugcgagagggcaaugcacucuucaucgcgcucacguuaguccucacc | manual\_scaffold\_1b:784802472..784802527:+ |
| manual\_scaffold\_5\_18154 | 0.1 | 0.75 ± 0.01 |  | 66 | 66 | 0 | 0 | yes |  |  |  | blast | uucuagaagucauuggauac | acccaacuucauuuagauua | uucuagaagucauuggauaccagaaguaauaucacuggacccaacuucauuuagauua | manual\_scaffold\_5:690737256..690737314:+ |
| manual\_scaffold\_3b\_10670 | 0.1 | 0.75 ± 0.01 |  | 344 | 344 | 0 | 0 | yes |  |  |  | blast | ugaggacuugcgagacggca | cggucacauuaguccucaac | ugaggacuugcgagacggcaauguagucugcaucucggucacauuaguccucaac | manual\_scaffold\_3b:758432290..758432345:+ |
| manual\_scaffold\_8\_28998 | 0.1 | 0.75 ± 0.01 |  | 13 | 13 | 0 | 0 | yes |  |  |  | blast | cacuuggccauacuuuaacucu | aguuuggagcugccaggagau | cacuuggccauacuuuaacucugugcuauaauagcaaguagaguuuggagcugccaggagau | manual\_scaffold\_8:596613348..596613410:+ |
| manual\_scaffold\_4a\_14158 | 0.1 | 0.75 ± 0.01 |  | 8 | 8 | 0 | 0 | yes |  |  |  | blast | acugaggacgagaucaucucgu | gauauggucuuguccucagcag | gauauggucuuguccucagcagcucuggagcugucugcacugaggacgagaucaucucgu | manual\_scaffold\_4a:142128666..142128726:- |
| manual\_scaffold\_1b\_2007 | 0 | 0.75 ± 0.01 |  | 9 | 9 | 0 | 0 | yes |  |  |  | blast | ucagugacugucugcugcu | caggggaccaagcacugauu | caggggaccaagcacugauucauuuaacuuaaucagugacugucugcugcu | manual\_scaffold\_1b:243533204..243533255:+ |
| manual\_scaffold\_4b\_17439 | 0 | 0.75 ± 0.01 |  | 126 | 126 | 0 | 0 | yes |  |  |  | blast | cuaucaacuuucgauggu | cauuguaaccaaugauagca | cuaucaacuuucgaugguaguguaugugccuaccauuguaaccaaugauagca | manual\_scaffold\_4b:1174200514..1174200567:- |
| manual\_scaffold\_2a\_4569 | 0 | 0.75 ± 0.01 |  | 3 | 3 | 0 | 0 | yes |  | xtr-miR-428a |  | blast | aaagugcuguuuuguugg | aacaaaacagcugcauga | aacaaaacagcugcaugacaauuccauguacauaugaugcaguuaaaugcaccauaggagugucagggaaagugcuguuuuguugg | manual\_scaffold\_2a:1000767980..1000768066:+ |
| manual\_scaffold\_10\_33765 | 0 | 0.75 ± 0.01 |  | 30 | 30 | 0 | 0 | yes |  |  |  | blast | uaaguguuuccuguugugguuu | gcuugaacugaggcaccugcu | gcuugaacugaggcaccugcuauguuuauauuuguaaguguuuccuguugugguuu | manual\_scaffold\_10:283992728..283992784:+ |
| manual\_scaffold\_5\_19344 | 0 | 0.75 ± 0.01 |  | 8 | 8 | 0 | 0 | yes |  |  |  | blast | uugucaggacgccucugcacacu | uugugcagaggcgaccuaacaauu | uugugcagaggcgaccuaacaauuuggaggucugcaaacuugucaggacgccucugcacacu | manual\_scaffold\_5:263863626..263863688:- |
| manual\_scaffold\_7\_26895 | 0 | 0.75 ± 0.01 |  | 11 | 11 | 0 | 0 | yes |  |  |  | blast | cugaggaucugcugucaugcug | gauguaguggcuggaucugggaa | cugaggaucugcugucaugcugucuguggguaagauagauguaguggcuggaucugggaa | manual\_scaffold\_7:328074092..328074152:- |
| manual\_scaffold\_7\_27185 | 0 | 0.75 ± 0.01 |  | 8 | 8 | 0 | 0 | yes |  |  |  | blast | acgccaauccugaugcgggcuu | gaagcgucaggauuggcuuaa | acgccaauccugaugcgggcuuucaugcugaugggagaagcgucaggauuggcuuaa | manual\_scaffold\_7:650253185..650253242:- |
| manual\_scaffold\_8\_29390 | 0 | 0.75 ± 0.01 |  | 8 | 7 | 0 | 1 | no |  |  |  | blast | cacuuggccacuguaagacucugc | agaauuggagcugccaggaggu | cacuuggccacuguaagacucugcgcuguuaaggcuuguagaauuggagcugccaggaggu | manual\_scaffold\_8:1238290816..1238290877:+ |
| manual\_scaffold\_9\_32376 | 0 | 0.75 ± 0.01 |  | 11 | 11 | 0 | 0 | yes |  |  |  | blast | uuuaaaagugagacaaggacc | ccuuugcaucucaguuacaacu | ccuuugcaucucaguuacaacuucuaggaugcugaaaguuuaaaagugagacaaggacc | manual\_scaffold\_9:180254567..180254626:- |
| manual\_scaffold\_10\_33321 | 0 | 0.75 ± 0.01 |  | 8 | 8 | 0 | 0 | yes |  |  |  | blast | uucccucgguccaacacuuccu | ggagugaugcagcgagggggag | ggagugaugcagcgagggggagacagucacuggaacacuccuucccucgguccaacacuuccu | manual\_scaffold\_10:14451909..14451972:+ |
| manual\_scaffold\_6\_23288 | 0 | 0.75 ± 0.01 |  | 8 | 8 | 0 | 0 | yes |  |  |  | blast | ugcuccgguggcguaggcccuc | ggccugcgccaccguagcuuc | ggccugcgccaccguagcuucacuuuuuaugaugcuccgguggcguaggcccuc | manual\_scaffold\_6:253343320..253343374:- |
| manual\_scaffold\_3a\_8779 | 0 | 0.75 ± 0.01 |  | 9 | 9 | 0 | 0 | yes |  |  |  | blast | caggaugcagaacaguugcuuc | aguggcuguuuugcuuccuacu | caggaugcagaacaguugcuucaaauguuuaaguggcuguuuugcuuccuacu | manual\_scaffold\_3a:108076954..108077007:- |
| manual\_scaffold\_4a\_14491 | 0 | 0.75 ± 0.01 |  | 9 | 9 | 0 | 0 | yes |  |  |  | blast | uuuuccccuuguaaauuucagc | ugaaauuuaucuggagaagcau | uuuuccccuuguaaauuucagcaugugugaccuacugaaauuuaucuggagaagcau | manual\_scaffold\_4a:568508537..568508594:- |
| manual\_scaffold\_1a\_1486 | 0 | 0.75 ± 0.01 |  | 9 | 9 | 0 | 0 | yes |  |  |  | blast | aagaaucuaguacugagc | uucuggugguaggagcagga | uucuggugguaggagcaggagucaucggaugcgagcugcugaagaaucuaguacugagc | manual\_scaffold\_1a:789246548..789246607:- |
| manual\_scaffold\_11\_36632 | 0 | 0.75 ± 0.01 |  | 8 | 8 | 0 | 0 | yes |  |  |  | blast | uugacaauacagggaugcacc | uguaucccuguguugugaga | uugacaauacagggaugcaccaaaauggguucaauaugguguaucccuguguugugaga | manual\_scaffold\_11:951164517..951164576:+ |
| manual\_scaffold\_2a\_4190 | 0 | 0.75 ± 0.01 |  | 7 | 6 | 0 | 1 | yes |  |  |  | blast | uuuggagggccacagaaucuug | caagauucuguggcccucca | uuuggagggccacagaaucuugguuggugcagaagaggauagugaaguguauuuuuacugcuccaaccaagauucuguggcccucca | manual\_scaffold\_2a:507510675..507510762:+ |
| manual\_scaffold\_12\_39099 | 0 | 0.75 ± 0.01 |  | 16 | 15 | 0 | 1 | yes |  |  |  | blast | uuugcauucugggacguauagu | uacaagcugcgaauacaaagc | uuugcauucugggacguauaguuuuauuuuuauaaugugauacaagcugcgaauacaaagc | manual\_scaffold\_12:113759720..113759781:- |
| manual\_scaffold\_6\_23542 | 0 | 0.75 ± 0.01 |  | 22 | 22 | 0 | 0 | yes |  |  |  | blast | ucgcagucauguuaguucucacu | ugggacuugcgagagagcaaug | ugggacuugcgagagagcaauguacuauucaucgcagucauguuaguucucacu | manual\_scaffold\_6:556183056..556183110:- |
| manual\_scaffold\_5\_19817 | 0 | 0.75 ± 0.01 |  | 54 | 54 | 0 | 0 | yes |  |  |  | blast | accuggauccucuggccc | cccagaucccuccugggucc | accuggauccucuggcccccucuuccuucucacacagaguugaggcccccagaucccuccugggucc | manual\_scaffold\_5:831576828..831576895:- |
| manual\_scaffold\_5\_19473 | 0 | 0.75 ± 0.01 |  | 10 | 10 | 0 | 0 | yes |  |  |  | blast | uggcacugugugaaaaaa | uuuucacuuuuugcuggu | uggcacugugugaaaaaacuggguugauugcagaggcccccuaacuuucugcccccauuuuucacuuuuugcuggu | manual\_scaffold\_5:459264243..459264319:- |
| manual\_scaffold\_1b\_3033 | 0 | 0.75 ± 0.01 |  | 8 | 8 | 0 | 0 | yes |  |  |  | blast | aacugcacgucugguuggcu | ccagccugacaugugcaguuca | aacugcacgucugguuggcuggcguguuugcuguggccagccugacaugugcaguuca | manual\_scaffold\_1b:207440078..207440136:- |
| manual\_scaffold\_3b\_10953 | 0 | 0.75 ± 0.01 |  | 10 | 10 | 0 | 0 | yes |  |  |  | blast | agcggccucuuuggaaugccu | gcaaugcaaggaggcugucgg | gcaaugcaaggaggcugucggguguauguaugcauagcggccucuuuggaaugccu | manual\_scaffold\_3b:1061440649..1061440705:+ |
| manual\_scaffold\_2a\_5912 | 0 | 0.75 ± 0.01 |  | 9 | 9 | 0 | 0 | yes |  |  |  | blast | ucagugacugucugcugcu | cagggaccaaguccugauu | cagggaccaaguccugauugguacaacuuaaucagugacugucugcugcu | manual\_scaffold\_2a:1507407816..1507407866:- |
| manual\_scaffold\_10\_35444 | 0 | 0.75 ± 0.01 |  | 8 | 8 | 0 | 0 | yes |  |  |  | blast | cagaaauccgguagugcuc | gcacggcaugacuuccgcc | gcacggcaugacuuccgccacaagaagggcagaaauccgguagugcuc | manual\_scaffold\_10:810495085..810495133:- |
| manual\_scaffold\_6\_24054 | 0 | 0.75 ± 0.01 |  | 16 | 16 | 0 | 0 | yes |  |  |  | blast | uuugcgacuacaaaaaaaccu | guuuuuuuguggucacaaaca | guuuuuuuguggucacaaacagcccaacucugcgcaucagacuguuugcgacuacaaaaaaaccu | manual\_scaffold\_6:1143952173..1143952238:- |
| manual\_scaffold\_8\_28600 | 0 | 0.75 ± 0.01 |  | 8 | 8 | 0 | 0 | yes |  |  |  | blast | ucaauggucuuguucucagcgc | gcugaggaugagaccaucuugu | ucaauggucuuguucucagcgcagccagcgccaauggugcugaggaugagaccaucuugu | manual\_scaffold\_8:120637049..120637109:+ |

  
  
  

## mature miRBase miRNAs detected by miRDeep2

  


tag idthis is a tag id assigned by miRDeep2. The first part of the id designates the chromosome or genome contig on which the miRNA gene is located. The second part is a running number that is added to avoid identical ids. The running number is incremented by one for each potential miRNA precursor that is excised from the genome. Clicking this field will display a pdf of the structure, read signature and score breakdown of the miRNA. | miRDeep2 scorethe log-odds score assigned to the hairpin by miRDeep2 | estimated probability that the miRNA is a true positivethe estimated probability that a predicted miRNA with a score of this or higher is a true positive. To see exactly how this probability is estimated, mouse over the 'novel miRNAs, true positives' in the table at the top of the webpage. For miRBase miRNAs, this reflects the support that the data at hand lends to the miRNA. | rfam alertthis field indicates if the miRNA hairpin has sequence similarity to reference rRNAs or tRNAs. Warnings in this field should overrule the estimated probability that a reported miRNA is a true positive (previous field). | predicted mature seq. in accordance with miRBase mature seq.If the predicted miRDeep2 sequence overlaps with the miRBase annotated mature sequence than this is indicated by 'TRUE'. If the predicted miRDeep2 star sequence overlaps with the miRBase annotated mature sequence this is inidicated by 'STAR'. | total read countthis is the sum of read counts for the mature, loop and star miRNAs. | mature read countthis is the number of reads that map to the miRNA hairpin and are contained in the sequence covered by the consensus mature miRNA, including 2 nts upstream and 5 nts downstream. | loop read countthis is the number of reads that map to the miRNA hairpin and are contained in the sequence covered by the consensus miRNA loop, including 2 nts upstream and 5 nts downstream. | star read countthis is the number of reads that map to the miRNA hairpin and are contained in the sequence covered by the consensus star miRNA, including 2 nts upstream and 5 nts downstream. | significant randfold p-valuethis field indicates if the estimated randfold p-value of the miRNA hairpin is equal to or lower than 0.05 (see Bonnet et al., Bioinformatics, 2004). | mature miRBase miRNAthis field displays the ids of any reference mature miRNAs for the species that map perfectly (full length, no mismatches) to the reported miRNA hairpin. If this is the case, the reported miRNA hairpin is assigned as a known miRNA. If not, it is assigned as a novel miRNA. If more than one reference mature miRNA maps to the miRNA hairpin, then only the id of the reference miRBase miRNA that matches the predicted mature sequence is output. | example miRBase miRNA with the same seedthis field displays the ids of any reference mature miRNAs from related species that have a seed sequence identical to that of the reported mature miRNA. The seed is here defined as nucleotides 2-8 from the 5' end of the mature miRNA. If more than one reference mature miRNA have identical seed, then only the id of the miRNA that occurs last in the input file of reference mature miRNAs from related species is displayed. | UCSC browserif a species name was input to miRDeep2, then clicking this field will initiate a UCSC blat search of the consensus precursor sequence against the reference genome. | NCBI blastnclicking this field will initiate a NCBI blastn search of the consensus precursor sequence against the nr/nt database (non-redundant collection of all NCBI nucleotide sequences). | consensus mature sequencethis is the consensus mature miRNA sequence as inferred from the deep sequencing reads. | consensus star sequencethis is the consensus star miRNA sequence as inferred from the deep sequencing reads. | consensus precursor sequencethis is the consensus precursor miRNA sequence as inferred from the deep sequencing reads. Note that this is the inferred Drosha hairpin product, and therefore does not include substantial flanking genomic sequence as does most miRBase precursors. | precursor coordinateThe given precursor coordinates refer do absolute position in the mapped reference sequence || manual\_scaffold\_6\_22875 | 2.9e+6 | 0.96 ± 0.01 |  | TRUE | 5692809 | 5692001 | 3 | 805 | yes | pwa-mir-10a | xtr-miR-10b |  | blast | uacccuguagauccgaauuugu | caaauucgugucuaggggaau | uacccuguagauccgaauuugugugaaaaaacgcagagucacaaauucgugucuaggggaau | manual\_scaffold\_6:160074527..160074589:- |
| manual\_scaffold\_7\_26410 | 2.1e+6 | 0.96 ± 0.01 |  | TRUE | 4301929 | 4288502 | 0 | 13427 | yes | pwa-mir-99b | xtr-miR-99 |  | blast | aacccguagauccgaacuugc | caagcucgagucugugggucu | aacccguagauccgaacuugcgguaugauucacacacaagcucgagucugugggucu | manual\_scaffold\_7:1559939164..1559939221:+ |
| manual\_scaffold\_6\_22594 | 2.0e+6 | 0.96 ± 0.01 |  | TRUE | 4024520 | 4012784 | 1 | 11735 | yes | pwa-mir-181a | xtr-miR-181b |  | blast | aacauucaacgcugucggugagu | accaucgaucguugacuguacc | aacauucaacgcugucggugaguuuuggacugagcagaaaccaucgaucguugacuguacc | manual\_scaffold\_6:1736408560..1736408621:+ |
| manual\_scaffold\_4a\_14635 | 2.0e+6 | 0.96 ± 0.01 |  | TRUE | 4023243 | 4011354 | 0 | 11889 | no | pwa-mir-181a | xtr-miR-181b |  | blast | aacauucaacgcugucggugagu | accaucgaccguugacuguacc | aacauucaacgcugucggugaguuugaaaauagaaagaaaaccaucgaccguugacuguacc | manual\_scaffold\_4a:737163527..737163589:- |
| manual\_scaffold\_4b\_16871 | 1.9e+6 | 0.96 ± 0.01 |  | TRUE | 3762589 | 3758842 | 1 | 3746 | yes | pwa-mir-182 | xtr-miR-182-5p |  | blast | uuuggcaaugguagaacucaca | ugguucuagacuugccaacu | uuuggcaaugguagaacucacacuggugagcuauaaggauccggugguucuagacuugccaacu | manual\_scaffold\_4b:479428005..479428069:- |
| manual\_scaffold\_4b\_16223 | 1.9e+6 | 0.96 ± 0.01 |  | TRUE | 3749000 | 3747869 | 1 | 1130 | yes | pwa-mir-26 | xtr-miR-26 |  | blast | uucaaguaauccaggauaggcu | ccuauucucgauuacuuguucc | uucaaguaauccaggauaggcuguuucuucgcagugcggccuauucucgauuacuuguucc | manual\_scaffold\_4b:72261067..72261128:- |
| manual\_scaffold\_5\_19489 | 1.7e+6 | 0.96 ± 0.01 |  | TRUE | 3464345 | 3464341 | 3 | 1 | yes | pwa-mir-30a | xtr-miR-30b |  | blast | uguaaacauccucgacugga | ucagucggauguuugcagc | uguaaacauccucgacuggaaguuguggagaugcugcuggagcuuucagucggauguuugcagc | manual\_scaffold\_5:467118432..467118496:- |
| manual\_scaffold\_10\_35440 | 1.4e+6 | 0.96 ± 0.01 |  | TRUE | 2913750 | 2888210 | 0 | 25540 | yes | pwa-mir-148a | xtr-miR-148a |  | blast | ucagugcacuacagaacuuugu | aaaguucugugacacuuagacu | aaaguucugugacacuuagacucgauauuaaagaagucagugcacuacagaacuuugu | manual\_scaffold\_10:804178128..804178186:- |
| manual\_scaffold\_4b\_17254 | 1.1e+6 | 0.96 ± 0.01 |  | TRUE | 2157858 | 2134944 | 1429 | 21485 | yes | pwa-let-7a | xtr-let-7a |  | blast | ugagguaguagguuguauaguu | uauacaaucuacugucuuuccu | ugagguaguagguuguauaguuuuaggggcacccccugucugucagauaacuauacaaucuacugucuuuccu | manual\_scaffold\_4b:986912735..986912808:- |
| manual\_scaffold\_6\_23589 | 1.0e+6 | 0.96 ± 0.01 |  | TRUE | 2130407 | 2130386 | 0 | 21 | yes | pwa-let-7m | xtr-let-7a |  | blast | ugagguaguagguuguauaguu | cuauacaguccauugccuuccu | ugagguaguagguuguauaguuuggcaggagguauuucauccaugucguggugauaacuauacaguccauugccuuccu | manual\_scaffold\_6:609756624..609756703:- |
| manual\_scaffold\_6\_23585 | 1.0e+6 | 0.96 ± 0.01 |  | TRUE | 2130407 | 2130386 | 0 | 21 | yes | pwa-let-7m | xtr-let-7a |  | blast | ugagguaguagguuguauaguu | cuauacaguccauugccuuccu | ugagguaguagguuguauaguuuggcaggagguauuucauccaugucguggugauaacuauacaguccauugccuuccu | manual\_scaffold\_6:609751108..609751187:- |
| manual\_scaffold\_1b\_2189 | 1.0e+6 | 0.96 ± 0.01 |  | TRUE | 2096880 | 2093840 | 3 | 3037 | yes | pwa-mir-27b | xtr-miR-27b |  | blast | uucacaguggcuaaguucugc | agagcuuagcugauuggugaac | agagcuuagcugauuggugaacagugauuacuuucucuuuuguucacaguggcuaaguucugc | manual\_scaffold\_1b:438512215..438512278:+ |
[truncated: 85,265 more chars]
