# Supplementary material for: Chromosome-scale genome assembly reveals how repeat elements shape non-coding RNA landscapes active during newt limb regeneration
Source: Cell Genom. 2025 Jan 27;5(2):100761. doi: 10.1016/j.xgen.2025.100761 (PMC11872487; doi:10.1016/j.xgen.2025.100761)
Supplement: Data S8. A pipeline for microsynteny analysis, related to STAR Methods: Protein-based synteny [file mmc24.pdf]

Finding a gene of interest

1. Download JBrowse2 Desktop (https://jbrowse.org/jb2/download/#jbrowse-2-desktop)
2. Download all the files from here for Pleurodeles (https://doi.org/10.17617/3.90C1ND) and put these into a folder of your choosing.
3. Prepare the gff3 file (aPleWal1.anno.20220803.gff3) using genometools like :

```
gt gff3 -sortlines -tidy -retainids aPleWal1.anno.20220803.gff3 >
aPleWal1.tidy.anno.20220803.gff3
```

4. Now use samtools to bgzip and index this gff3:

```
bgzip aPleWal1.tidy.anno.20220803.gff3
tabix --csi aPleWal1.tidy.anno.20220803.gff3.gz
```

5. Open JBrowse Desktop and click “Open Sequence File(s)”
6. Fill in the “Assembly Name” and “Assembly display name” fields, this can just be “Pleurodeles” for both.
7. Under the “Type” dropdown select “BgzipFastaAdapter”
8. Now you need to load the fasta genome files, click “CHOOSE FILE” under the fastaLocation and then navigate to where you stored the downloads from step 2. You should then select “aPleWal1.pri.20220803.fa.gz”.
9. Go over to the faiLocation and do the same, clicking “CHOOSE FILE” and then navigating to the same folder where you saved the downloads from step 2. Now select “aPleWal1.pri.20220803.fa.gz.fai”
10. Finally under gziLocation do the same as above, this time selecting “aPleWal1.pri.20220803.fa.gz.gzi”
11. Now you can press submit and you should see the following:

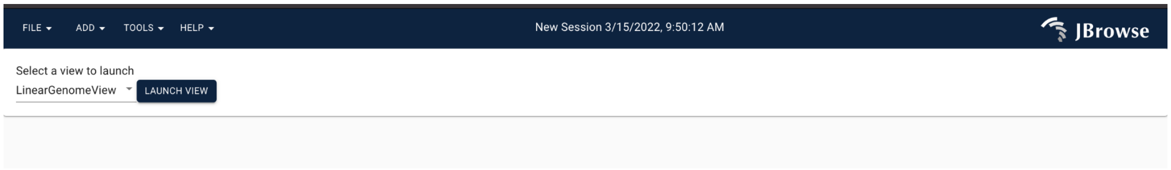

12. Click “Launch View” (you can also select other ways to view the genome with the dropdown)
13. Now click “Open”
14. Now you need to add the gff file which has the annotations in it. Go to “FILE” in the top left corner and select “Open track…”
15. This will open a sidebar on the right, you can find “Main file” where you will now click “FILE” and “CHOOSE FILE” again navigating to the folder you downloaded everything in and selecting “aPleWal1.tidy.anno.20220803.gff3.gz” and opening this file.
16. Now look below at “Index file” and do the same FILE→CHOOSE FILE followed by selecting “aPleWal1.tidy.anno.20220803.gff3.gzi” and opening this file.

17. Press “NEXT” and you should get: 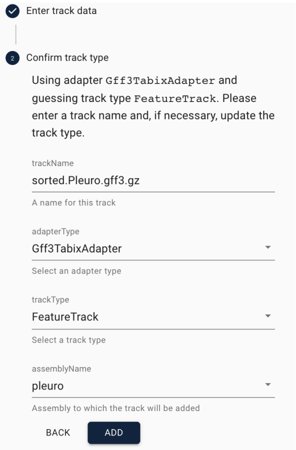

. The “assemblyName” changes based on what you put in for step 4. Now press “ADD”.
18. Now you have a genome with the gff (annotation file) loaded into JBrowse. The genome is really big, so when you’re zoomed out you may have to click “FORCE LOAD” (see below screenshot for an example of what force load looks like) to see the annotations.

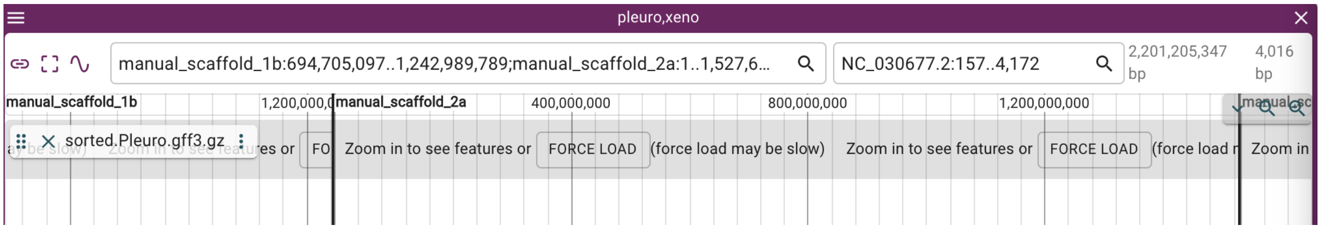

19. You can now navigate based on chromosome and location. You have to enable text searching based on gene_id to search for your gene of interest. To do this click the three dots to the right of the track name (e.g., above “sorted.Pleuro.gff3.gz) and then click “Settings”. Scroll down to “textSearching” and click “ADD ITEM”. Add in gene_id in the field. After you’ve done this click again on the three dots and select “Re-index track”. Now you should be able to search by gene name.
20. As an example you can search FGF20 and you see FGF20.1 comes up. In this annotation the gene was given the ID of its best UNIPROT homolog followed by an integer suffix (e.g., .1). If multiple genes shared the same UNIPROT ID, each additional gene will have a different integer suffix. In addition, transcript isoforms are identified with an additional integer suffix after the gene name (e.g., FGF20.1.1, FGF20.1.2, etc)
21. Once you select one of the FGF20.1 from the dropdown JBrowse will take you to (potentially) fgf20. If you click on the gene you will see a sidebar with more information and it should also let you get protein sequence and various other sequences of interest. I really like throwing the protein sequence into https://shoot.bio/ just to get a feel if the annotation makes sense. Confirming the annotation via a manual process can then improve genome annotation. This paired with the synteny analysis will be informative of whether the annotation is correct.
22. You can also select regions surrounding your gene of interest and take this sequence if desired.

Synteny

1. To begin looking at synteny between species you need to do a slight variation of the above steps (3-22) for your comparison genome of choice. You need to choose a species of interest and download the genome and gff3 file so that you can repeat steps 3-22. Some common species of interest are:

```
#one small note for the below files, JBrowse wants files ending in .fasta or .fa so you will want to rename these files with that ending before you get to JBrowse
#these are the genomes we used in the paper
#Gar, to download files you can do
wget
https://ftp.ncbi.nlm.nih.gov/genomes/all/GCF/000/242/695/GCF_000242695.1_LepOcu1/GCF_000242695.1_LepOcu1_genomic.fna.gz
wget
https://ftp.ncbi.nlm.nih.gov/genomes/all/GCF/000/242/695/GCF_000242695.1_LepOcu1/GCF_000242695.1_LepOcu1_genomic.gff.gz

#Axolotl
wget https://www.axolotl-omics.org/dl/AmexG_v6.0-DD.fa.gz
wget https://www.axolotl-omics.org/dl/AmexT_v47-AmexG_v6.0-DD.gtf.gz
#we converted this to gff3 format with AGAT
#(https://agat.readthedocs.io/en/latest/index.html) like so
agat_convert_sp_gxf2gxf.pl -g AmexT_v47-AmexG_v6.0-DD.gtf AmexT_v47-AmexG_v6.0-DD.gff3

#Human
wget
https://ftp.ncbi.nlm.nih.gov/genomes/all/GCA/000/001/405/GCA_000001405.15_GRCh38/seqs_for_alignment_pipelines.ucsc_ids/GCA_000001405.15_GRCh38_full_analysis_set.fna.gz

wget
https://ftp.ncbi.nlm.nih.gov/refseq/H_sapiens/annotation/GRCh38_latest/refseq_identifiers/GRCh38_latest_genomic.gff.gz

#xenopus
wget
https://ftp.ncbi.nlm.nih.gov/genomes/all/GCF/000/004/195/GCF_000004195.4_UCB_Xtro_10.0/GCF_000004195.4_UCB_Xtro_10.0_genomic.fna.gz
wget
https://ftp.ncbi.nlm.nih.gov/genomes/all/GCF/000/004/195/GCF_000004195.4_UCB_Xtro_10.0/GCF_000004195.4_UCB_Xtro_10.0_genomic.gff.gz
#lungfish
wget
https://ftp.ncbi.nlm.nih.gov/genomes/all/GCF/019/279/795/GCF_019279795.1_PAN1.0/GCF_019279795.1_PAN1.0_genomic.fna.gz

wget
https://ftp.ncbi.nlm.nih.gov/genomes/all/GCF/019/279/795/GCF_019279795.1_PAN1.0/GCF_019279795.1_PAN1.0_genomic.gff.gz
```

Keep in mind that this is a LOT of data, and depending on how much storage and RAM your computer has you may have some difficulties with these files. Also, realize that these files are from various sources and the annotation may differ, so if you are struggling to find a gene of interest it may take a bit more searching for the gene versus protein name. You can make a copy of the .gff.gz files and use samtools `bgzip -d` to make a version that can be looked at to see what the annotation looks like (I suggest something like `tail -n 20 file_name.gff3`.

2. To load your genome in, click on TOOLS→Open Assembly Manager. Then click “ADD NEW ASSEMBLY”, and go ahead and add another genome with “BgzipFastaAdapter” type selected. You’ll also need to add a new “track” (this is the gff annotation file). We will do this below.
3. Once you've loaded all your genomes, you can save your session (File→Save As) so that you can easily reload it in the future without going through all the above steps again (JBrowse will also autosave your session).
4. To look for syntenic blocks that are conserved between species go to Add→Linear Synteny view. This will then let you choose between two of the genomes that you are interested in comparing (as long as you’ve loaded these into JBrowse).
5. Once you open your Linear Synteny view you’ll see that for Pleurodeles there is already the track (sorted.Pleuro.gff3.gz) associated with the Pleurodeles genome. However, you haven’t added a track for your second genome of choice yet. Using Xenopus as an example, we will see this:

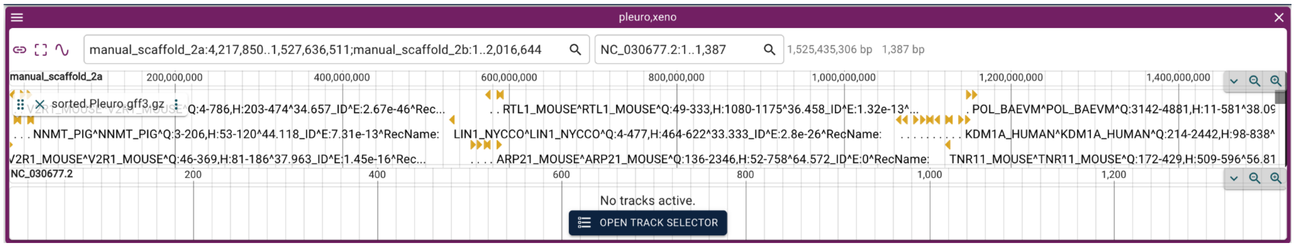

You now need to add the sorted.Xenopus.gff file as a track. Click “OPEN TRACK SELECTOR” and you’ll see the below come up on the right hand side of the screen. Click the three lines and select “add track”. Now you’ll add the “sorted.Xenopus.gff.gz” like you did above in step 14 for Pleurodeles.

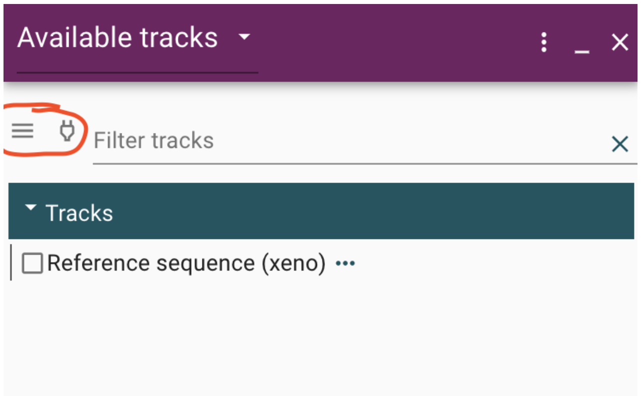

6. Now in this Linear Synteny view you can look at the two genomes and synteny between species. You will want to search for your gene of interest in both genomes separately and then look at the gene order between the species. Using our fgf20 example you would find the following:

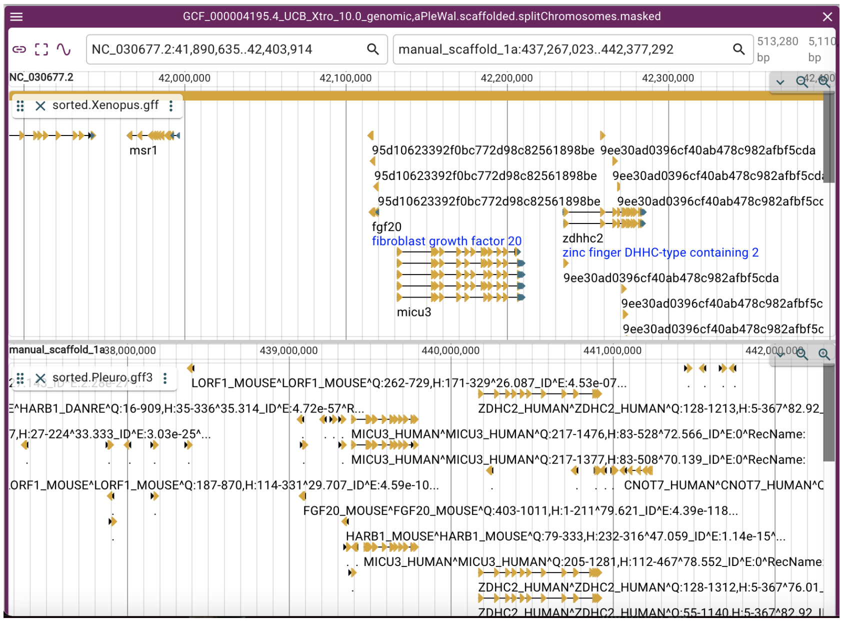

This is comparing Xenopus (top) to Pleurodeles (bottom). You can see fgf20 in both views, to the right of fgf20 you can see micu3 and zdhhc2 in both the Xenopus and Pleurodeles. To the left you see msr1 in Xenopus and nothing looking like that in Pleurodeles. However, if you scroll a bit further you can see a gene tusc3 present in both genomes to the left of fgf20 (not in the above picture). It is clear that this is a syntenic block that is conserved between these two species (also called collinearity). This is evidence that fgf20 that is annotated as such in both genomes are likely orthologs of one another. This is just comparing between Xenopus and Pleurodeles though, so you may want to extend your comparisons to the various other species available. Keep in mind we are still working on annotating the Pleurodeles genome and you will find quite a bit of annotations that are not currently insightful. These are typically “LORF1” and “HARB1”, you can see a few examples of this above.

7. If you take the time to do this analysis it is great if you save and share this information!
